# Safety, tolerability, and pharmacokinetics of long-acting injectable cabotegravir in low-risk HIV-uninfected individuals: HPTN 077, a phase 2a randomized controlled trial

**DOI:** 10.1371/journal.pmed.1002690

**Published:** 2018-11-08

**Authors:** Raphael J. Landovitz, Sue Li, Beatriz Grinsztejn, Halima Dawood, Albert Y. Liu, Manya Magnus, Mina C. Hosseinipour, Ravindre Panchia, Leslie Cottle, Gordon Chau, Paul Richardson, Mark A. Marzinke, Craig W. Hendrix, Susan H. Eshleman, Yinfeng Zhang, Elizabeth Tolley, Jeremy Sugarman, Ryan Kofron, Adeola Adeyeye, David Burns, Alex R. Rinehart, David Margolis, William R. Spreen, Myron S. Cohen, Marybeth McCauley, Joseph J. Eron

**Affiliations:** 1 UCLA Center for Clinical AIDS Research and Education, Los Angeles, California, United States of America; 2 Statistical Center for HIV/AIDS Research and Prevention, Fred Hutchinson Cancer Research Center, Seattle, Washington, United States of America; 3 Evandro Chagas National Institute of Infectious Diseases, Oswaldo Cruz Foundation, Rio de Janeiro, Brazil; 4 Centre for the AIDS Programme of Research in South Africa, University of KwaZulu Natal, Durban, South Africa; 5 Bridge HIV, Population Health Division, San Francisco Department of Health, San Francisco, California, United States of America; 6 Department of Epidemiology and Biostatistics, Milken Institute School of Public Health, George Washington University, Washington, District of Columbia, United States of America; 7 UNC Project–Malawi, Lilongwe, Malawi; 8 Perinatal HIV Research Unit, Chris Hani Baragwanath Hospital, Soweto, South Africa; 9 School of Medicine, Johns Hopkins University, Baltimore, Maryland, United States of America; 10 FHI360, Durham, North Carolina, United States of America; 11 Berman Institute of Bioethics, Johns Hopkins University, Baltimore, Maryland, United States of America; 12 Division of AIDS, National Institute of Allergy and Infectious Diseases, National Institutes of Health, Rockville, Maryland, United States of America; 13 ViiV Healthcare, Durham, North Carolina, United States of America; 14 University of North Carolina at Chapel Hill, Chapel Hill, North Carolina, United States of America; University of Southampton, UNITED KINGDOM

## Abstract

**Background:**

Cabotegravir (CAB) is a novel strand-transfer integrase inhibitor being developed for HIV treatment and prevention. CAB is formulated both as an immediate-release oral tablet for daily administration and as a long-acting injectable suspension (long-acting CAB [CAB LA]) for intramuscular (IM) administration, which delivers prolonged plasma exposure to the drug after IM injection. HIV Prevention Trials Network study 077 (HPTN 077) evaluated the safety, tolerability, and pharmacokinetics of CAB LA in HIV-uninfected males and females at 8 sites in Brazil, Malawi, South Africa, and the United States.

**Methods and findings:**

HPTN 077 was a double-blind, placebo-controlled phase 2a trial. Healthy individuals age 18–65 years at low HIV risk were randomized (3:1) to receive CAB or placebo (PBO). In the initial oral phase, participants received 1 daily oral tablet (CAB or PBO) for 4 weeks. Those without safety concerns in the oral phase continued and received injections in the injection phase (Cohort 1: 3 injections of CAB LA 800 mg or 0.9% saline as PBO IM every 12 weeks for 3 injection cycles; Cohort 2: CAB LA 600 mg or PBO IM for 5 injection cycles; the first 2 injections in Cohort 2 were separated by 4 weeks, the rest by 8 weeks). The primary analysis included weeks 5 to 41 of study participation, encompassing the injection phase. The cohorts were enrolled sequentially. Primary outcomes were safety and tolerability. Secondary outcomes included pharmacokinetics and events occurring during the oral and injection phases. Between February 9, 2015, and May 27, 2016, the study screened 443 individuals and enrolled 110 participants in Cohort 1 and 89 eligible participants in Cohort 2. Participant population characteristics were as follows: 66% female at birth; median age 31 years; 27% non-Hispanic white, 41% non-Hispanic black, 24% Hispanic/Latino, 3% Asian, and 6% mixed/other; and 6 transgender men and 1 transgender woman. Twenty-two (11%) participants discontinued the oral study product; 6 of these were for clinical or laboratory adverse events (AEs). Of those who received at least 1 CAB LA injection, 80% of Cohort 1 and 92% of Cohort 2 participants completed all injections; injection course completion rates were not different from those in the PBO arm. Injection site reactions (ISRs) were common (92% of Cohort 1 and 88% of Cohort 2 participants who received CAB LA reported any ISR). ISRs were mostly Grade 1 (mild) to Grade 2 (moderate), and 1 ISR event (Cohort 1) led to product discontinuation. Grade 2 or higher ISRs were the only AEs reported more commonly among CAB LA recipients than PBO recipients. Two Grade 3 (severe) ISRs occurred in CAB recipients, 1 in each cohort, but did not lead to product discontinuation in either case. Seven incident sexually transmitted infections were diagnosed in 6 participants. One HIV infection occurred in a participant 48 weeks after last injection of CAB LA: CAB was not detectable in plasma both at the time of first reactive HIV test and at the study visit 12 weeks prior to the first reactive test. Participants in Cohort 2 (unlike Cohort 1) consistently met prespecified pharmacokinetic targets of at least 95% of participants maintaining CAB trough concentrations above PA-IC_90_, and 80% maintaining trough concentrations above 4× PA-IC_90_. Study limitations include a modest sample size, a short course of injections, and a low-risk study population.

**Conclusions:**

In this study, CAB LA was well tolerated at the doses and dosing intervals used. ISRs were common, but infrequently led to product discontinuation. CAB LA 600 mg every 8 weeks met pharmacokinetic targets for both male and female study participants. The safety and pharmacokinetic results observed support the further development of CAB LA, and efficacy studies of CAB LA for HIV treatment and prevention are in progress.

**Trial registration:**

ClinicalTrials.gov Registry: ClinicalTrials.gov Trial number: NCT02178800.

## Abstract 2

In a phase 2a trial, Raphael Landovitz and colleagues investigate the safety of injectable cabotegravir in HIV-uninfected people

## Author summary

**Why was this study done?:**

- Daily oral pre-exposure prophylaxis (PrEP) with tenofovir disoproxil fumarate/emtricitabine is highly effective in preventing HIV infection when taken as prescribed, but adherence challenges have compromised full effectiveness in some populations.
- Cabotegravir (CAB) is a novel strand-transfer integrase inhibitor in development as a long-acting injectable preparation for HIV treatment and HIV prevention.
- HIV Prevention Trials Network study 077 (HPTN 077) aimed to establish the safety, tolerability, and pharmacokinetics of 2 dose/interval regimens of long-acting CAB in HIV-uninfected, low-risk individuals globally.

**What did the researchers do and find?:**

- Participants were randomized to active CAB or placebo. All participants received a 4-week daily oral lead-in of CAB or placebo, followed by a series of injections of CAB or placebo. Participants received either 800 mg as a split 2-ml injection every 12 weeks, or 600 mg as a single 3-ml injection every 8 weeks, after receiving 2 injections 4 weeks apart.
- We observed frequent injection site reactions (mostly injection site pain), and they were more common with CAB than placebo, but only led to discontinuation of injections in rare cases. No other safety concerns were noted.
- Pharmacokinetics, using target values from nonhuman primate simian/human immunodeficiency virus challenge prevention studies, support the 600 mg every 8 weeks dose, meeting prespecified targets.

**What do these findings mean?:**

- We found that CAB was generally well tolerated in HIV-uninfected males and females in diverse geographic locations.
- Injection site reactions, although frequent, did not deter continued dosing, albeit with short courses.
- Studies are ongoing to evaluate efficacy in at-risk populations and to further evaluate the safety of the 600 mg every 8 weeks dose.

## Introduction

Oral tenofovir disoproxil fumarate (TDF)–based pre-exposure prophylaxis (PrEP) is highly effective for HIV prevention when taken as prescribed [1–5]. However, achieving and maintaining adherence rates sufficient for high-level protection against HIV is challenging for some individuals and populations [6–8]. Impediments to adherence with daily tablets may be as simple as forgetfulness but could involve more complicated reasons such as concerns about safety, stigma related to use, and potential harms if PrEP use is disclosed to sexual partners. Lessons learned from contraceptive technology suggest that an increased variety of product types for PrEP will increase the probability that at least 1 product will fit a given individual’s needs at a particular time [9]. For these reasons, there is interest in development and evaluation of HIV prevention agents that do not require daily adherence.

Cabotegravir (CAB) is an investigational strand-transfer integrase inhibitor with potent activity against HIV in vitro and in vivo [10,11]. CAB is formulated both as an oral tablet for daily administration and as a long-acting injectable suspension (long-acting CAB [CAB LA]) [12]. CAB LA is currently under development for HIV treatment in combination with long-acting injectable rilpivirine (NCT02951052 and NCT02938520) [13,14] and as a stand-alone antiviral agent for HIV prevention (NCT02720094 and NCT03164564). Nonhuman primate (NHP) models have demonstrated that CAB LA can protect against rectal, vaginal, parenteral, and penile simian immunodeficiency virus and simian/human immunodeficiency virus (SHIV) challenges [15–20]. In those studies, high levels of protection were seen against repeated exposures when CAB plasma concentrations were above 4 times the protein-adjusted IC_90_ (PA-IC_90_) (0.664 μg/ml), and were generally maintained at drug concentrations above the PA-IC_90_ (0.166 μg/ml).

In humans, the safety and pharmacokinetics of CAB LA were evaluated in a phase 2a trial, the ECLAIR trial, that enrolled HIV-uninfected low-risk men in the US [21]. This trial investigated CAB LA at a dose of 800 mg IM every 12 weeks. The ECLAIR study demonstrated that CAB LA was safe and well tolerated, but prespecified pharmacokinetic targets established from the NHP models for preventive efficacy were not consistently met [21]. In vitro data and data from NHP studies were used to set pharmacokinetic targets prior to study inception. The target median trough concentration was set at 1.35 μg/ml, which, if achieved, was predicted to attain trough concentrations of greater than or equal to 4× the PA-IC_90_ (0.664 μg/ml) in 80% of participants and greater than or equal to the PA-IC_90_ (0.166 μg/ml) in 95% of participants. In NHP models, no transmissions were observed with rectal or vaginal challenge when the concentration of CAB remained above 4× the PA-IC_90_, and most animals were protected when concentrations remained above the PA-IC_90_. No major integrase resistance mutations were found in SHIV isolates from NHP models of rectal and vaginal challenges where infections occurred during the pharmacokinetic tail [18,20].

Subsequent to the ECLAIR results, modeling based on datasets from both HIV-infected and HIV-uninfected individuals in ongoing studies suggested that a dose of 600 mg IM every 8 weeks, following an initial 4-week interval between first and second injections, is more likely to meet these pharmacokinetic targets. Accordingly, HIV Prevention Trials Network study 077 (HPTN 077) was undertaken to evaluate the safety, tolerability, and pharmacokinetics of CAB LA at 2 doses and intervals (800 mg every 12 weeks and 600 mg every 8 weeks) in HIV-negative, low-risk males and females in the US and resource-constrained countries as a requisite step in the development of CAB LA for PrEP for HIV.

## Methods

### Study design and participants

HPTN 077 is a randomized, double-blind placebo-controlled phase 2a trial conducted at 8 sites in Brazil, Malawi, South Africa, and the US. Participants were 18–65 years old, HIV uninfected at screening (determined using a US Food and Drug Administration [FDA]–cleared HIV rapid test, instrumented fourth generation antigen/antibody testing, and an HIV RNA test within 2 weeks of study entry), and at low risk for HIV infection (low infection risk was defined as no condomless anal or vaginal intercourse with HIV-infected or serostatus-unknown partners; no stimulant, inhaled nitrate, or injection drug use; no diagnosis of a sexually transmitted infection [STI]; and having fewer than 5 sexual partners—all in the past 12 months by self-report). Participants were generally in good health, with hemoglobin > 11 g/dl, absolute neutrophil count > 750 cells/mm^3^, platelets > 100,000/mm^3^, creatinine clearance ≥ 70 ml/min, normal aspartate aminotransferase and alanine aminotransferase values, and negative tests for hepatitis B and C virus infection. Females who were of reproductive potential were required to use an effective contraception method. Individuals were not eligible for enrollment if they had significant cardiovascular disease; had used post-exposure prophylaxis or PrEP in the 90 days prior to study entry; had known liver disease, coagulopathy, or seizures (added to exclusionary conditions mid-study); were pregnant or breastfeeding; or had a score of ≥8 on the Alcohol Use Disorders Identification Test (AUDIT) [22]. The study was carried out according to the study protocol (S1 Text) except where specifically noted, and is reported according to CONSORT (S1 CONSORT Checklist). The institutional review board or ethics committee at each participating site approved the protocol, and all participants provided written informed consent. The trial was registered as NCT02178800 at ClinicalTrials.gov.

### Randomization and masking

Participants were randomly assigned 3:1 by a computer-generated algorithm stratified by sex at birth and region (US or non-US) to CAB or placebo (PBO). CAB LA and 0.9% saline PBO injections were further masked with semi-opaque syringe overlays. Site pharmacists were not blinded to treatment assignments in order to provide appropriate study product; other study personnel and study participants remained blinded to treatment assignments from study entry until all participants completed the week 41 study visit and all data for the primary endpoint (i.e., the week 41 time point) had been collected. One laboratory investigator was unblinded for the purpose of selecting samples for CAB concentration testing; the unblinding information was not shared with any other study staff.

### Procedures

Participants were enrolled in 2 sequential cohorts with different dosing regimens. In the initial oral phase, randomized participants in both cohorts received 30 mg oral CAB or a matching oral PBO tablet daily for 4 weeks. HIV infection, safety, and tolerability were assessed after 2 and 4 weeks on study products. Participants proceeded to the injection phase of the study if they had adequate safety and tolerability measures and if they had at least 75% adherence to the oral study product (assessed by pill count) at the week 4 visit. The safety requirements for progression to the injection phase of the trial were stringent at the suggestion of the FDA (S1 Text).

**Fig 1:**
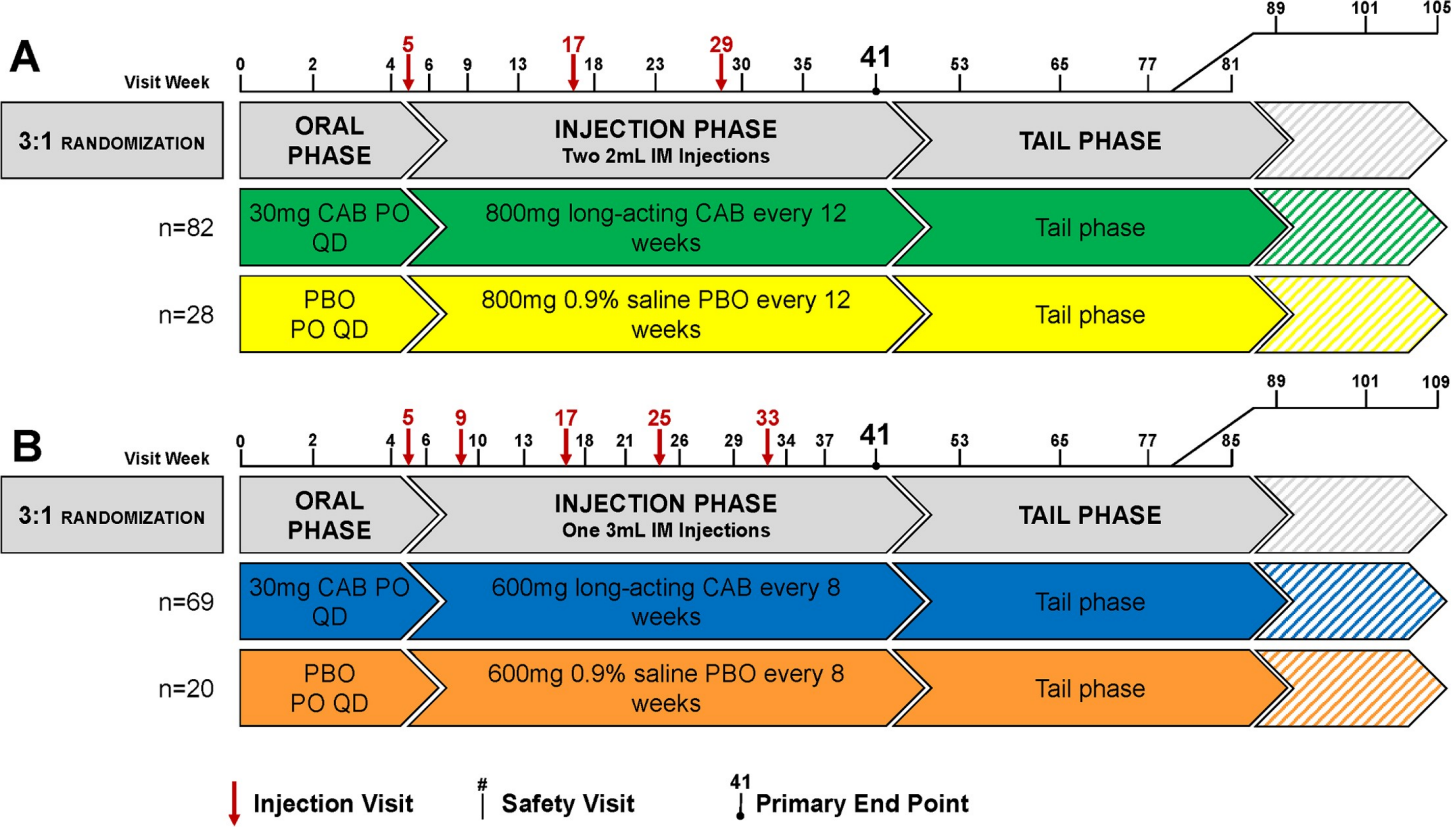
Study design. (A) Cohort 1 (CAB LA 800 mg or 0.9% saline PBO IM every 12 weeks). (B) Cohort 2 (CAB LA 600 mg or 0.9% saline PBO IM every 8 weeks after an initial 4-week interval). CAB, cabotegravir; CAB LA, long-acting cabotegravir; IM, intramuscular; PBO, placebo; PO, by mouth; QD, once daily.

Cohort 1 participants received 800 mg CAB LA (administered as two 400-mg [2 ml] IM injections) or 0.9% saline PBO. Injections of CAB LA and PBO were administered in the gluteal muscle, at weeks 5, 17, and 29 (every 12 weeks); this was the same dosing regimen used in the ECLAIR trial in men from the US. The primary endpoint was reached at week 41 (12 weeks after the final injection). HIV testing, laboratory and clinical safety assessments, and CAB plasma concentration measurements occurred at injection visits and at weeks 6, 9, 13, 18, 23, 30, 35, and 41 (Fig 1A).

Cohort 2 was added by protocol amendment following the results from the ECLAIR study in order to provide data on an alternative dosing scheme predicted to more consistently achieve pharmacokinetic targets. In Cohort 2, participants received 600 mg CAB LA or 0.9% saline PBO, administered as a single (3 ml) IM injection in the gluteal muscle. Injections were administered at weeks 5, 9, 17, 25, and 33 (every 8 weeks after a 4-week interval between the first and second injections). As in Cohort 1, the primary endpoint was reached at week 41 (8 weeks after the final injection). HIV testing, laboratory and clinical safety assessments, and CAB plasma concentration measurements occurred at injection visits and at weeks 6, 10, 13, 18, 21, 26, 29, 34, 37, and 41 (Fig 1B).

In both cohorts, PBO-treated participants were discontinued from long-term follow-up at the time of unblinding. Participants in both cohorts who received active CAB continued on quarterly follow-up for 52–76 weeks after their final injection. Participants who enrolled near the start of the study had only 52 weeks of follow-up, as designated by the protocol; follow-up was extended to 76 weeks for participants enrolled later in the study, when results from long-term follow-up in the ECLAIR study became available.

### Outcome assessments

The primary analysis included weeks 5 to 41 of study participation, encompassing the injection phase. The cohorts were enrolled sequentially. Primary outcomes were safety and tolerability. Secondary outcomes included pharmacokinetics and events occurring during the oral and injection phases. Additional clinical events of interest (HIV seroconversion and pregnancy in the tail-phase of the study) are also reported. All injection and safety visits included clinical and laboratory assessments for adverse events (AEs), HIV testing (using an FDA-approved HIV rapid test and instrumented antigen/antibody testing at all visits), injection site reaction (ISR) evaluation, vital signs, clinical examination, and review of concomitant medications. AEs were graded according to the Division of AIDS Table for Grading the Severity of Adult and Pediatric Adverse Events, Version 2.0 [23]. The satisfaction with study products and preferences were assessed at enrollment and 1 week after each injection; sexual risk behavior was assessed at enrollment, at injection visits, and at 12-week intervals during follow-up. Testing for STIs included urine and rectal *Neisseria gonorrhoeae* and *Chlamydia trachomatis* nucleic acid amplification testing and serologic syphilis testing at screening, and at approximately 6-month intervals throughout the study to monitor for changes in risk for HIV acquisition. For females of reproductive potential, pregnancy testing was performed in both cohorts at screening, enrollment, weeks 2 and 4, injection visits prior to each injection, the week 41 primary endpoint visit, and each quarterly follow-up visit. Electrocardiograms were performed at screening, enrollment, and weeks 4, 6, 9, 23, and 35 in Cohort 1 and weeks 4, 6, 13, 21, 29, and 37 in Cohort 2.

Plasma CAB concentrations were measured using a validated liquid chromatographic–mass spectrometric (LC-MS/MS) assay with a lower limit of quantification (LLOQ) of 0.025 μg/ml. On visits at which injections were administered, CAB concentrations were measured using samples collected prior to injection.

For the participant who acquired HIV infection, HIV drug resistance testing was retrospectively performed using the ViroSeq HIV-1 Genotyping System, version 2.0, and the ViroSeq HIV-1 Integrase Genotyping Kit (Abbott Molecular, Des Plaines, IL). HIV drug resistance testing was also performed using a research assay based on next generation sequencing (NGS) (see S2 Text).

Primary study outcomes were (1) safety, as determined by the proportion of participants experiencing any Grade 2 or higher clinical AE or any laboratory abnormality occurring from the initial injection at week 5 to the primary endpoint visit at week 41 among participants who received at least 1 injection, and (2) tolerability, as determined by the proportion of participants who discontinued injections before receiving the full course of injections for reasons of intolerability of injection (including but not limited to ISRs, injection burden or frequency, or any clinical or laboratory AE).

### Statistical analysis

The primary analysis is limited to data collected through the primary endpoint (week 41) and included all participants who met eligibility criteria and were exposed to any study product. Comparisons of baseline demographics between CAB and PBO treatment arms were done using the chi-squared test for categorical variables and Wilcoxon rank sum test for continuous variables. For the primary endpoint, a modified intention-to-treat analysis was conducted that included participants who received at least 1 injection. Fisher’s exact test was used to compare the proportion of participants in the CAB versus PBO treatment arm who experienced any Grade 2 or higher clinical AE or laboratory abnormality between the first injection at week 5 and the primary endpoint visit at week 41. Participants in the CAB treatment arm who received at least 1 injection were included in the analysis of plasma CAB concentrations. The means and the 90% prediction intervals at visits were estimated using linear mixed models. Plasma CAB concentrations were log-transformed before they were fitted to a linear mixed model. The geometric means of CAB concentrations were compared between participants who were males and females at birth using the Wald test in the linear mixed model.

Pharmacokinetic parameters were calculated for each participant who received active CAB. Non-compartmental analysis was used to estimate the area under the plasma concentration–time curve over the dosing interval (AUC_0–τ_), maximum plasma concentration (*C*_max_), and trough concentration at the end of the dosing interval (*C*_τ_). Concentrations below the LLOQ (0.025 μg/ml) were imputed as LLOQ/2 prior to the estimations. Plasma CAB concentrations were summarized from samples collected within defined post-injection sampling windows by sex at birth and cohort. The geometric means of pharmacokinetic parameters were estimated using the linear mixed model and compared between males and females and by body mass index (BMI) continuously and by BMI group (≥median versus <median, ≥25 versus <25 kg/m^2^, and ≥30 versus <30 kg/m^2^) by the Wald test. Other than comparisons of pharmacokinetic parameters, all comparisons between sexes were post hoc analyses. Tests with *p*-values ≤ 0.05 were considered to be statistically significant.

Safety was evaluated for CAB LA overall (Cohort 1 and Cohort 2 combined) and for each individual cohort. The ability of the study to detect serious adverse events (SAEs) was expressed by the true event rate above which at least 1 SAE would likely be observed and the true event rate below which no events would likely be observed. Specifically, for the CAB arm overall (*n* = 134), there was a 90% chance of observing no events if the true rate was 0.1% or less. Similarly, for the CAB arm in individual cohorts (*n =* 74 for Cohort 1; *n =* 60 for Cohort 2), there was a 90% chance of observing at least 1 event if the true rate of such an event was 3.1% or more for Cohort 1 and 3.8% or more for Cohort 2, and there was a 90% chance of observing no events if the true rate was 0.1% or less for both cohorts. The study had over 80% power to detect a 13%–26% difference in the proportion of participants experiencing a Grade 2 or higher clinical AE or laboratory abnormality between the CAB (*n =* 134) and PBO (*n =* 43) treatment arms if the proportion of participants with a Grade 2 or higher clinical AE or laboratory abnormality in the PBO arm ranged from 0% to 50%.

## Results

**Fig 2:**
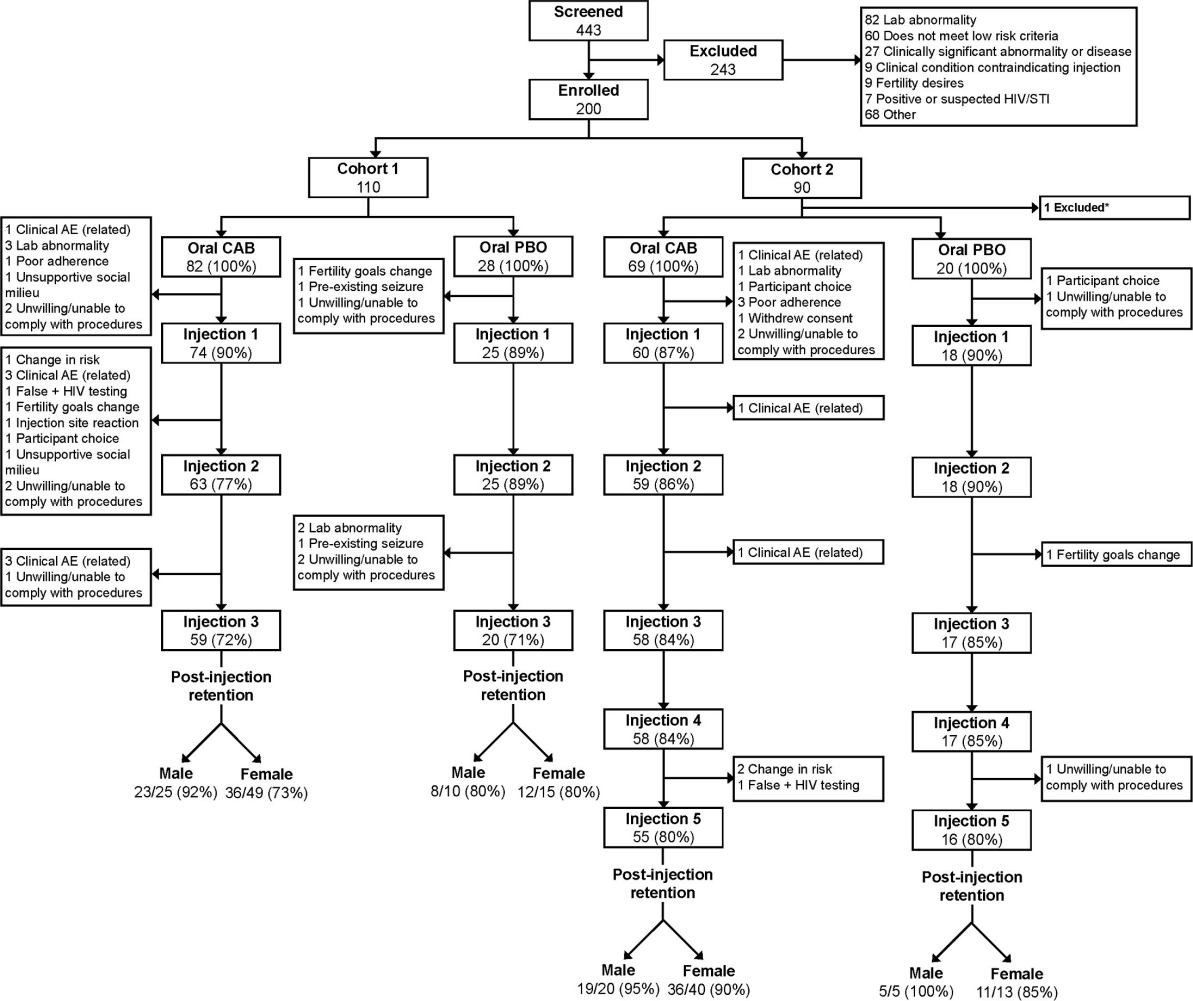
Study consort diagram. *One participant excluded due to exclusionary condition at enrollment. AE, adverse event; CAB, cabotegravir; PBO, placebo; STI, sexually transmitted infection.

A total of 443 individuals were screened, and 200 participants were enrolled (Fig 2). The most frequent reasons for screening failure were not meeting the protocol’s low-risk or clinical inclusion criteria. One hundred ten participants were enrolled in Cohort 1 and were randomized 3:1 CAB to PBO (82 CAB and 28 PBO) from February 9, 2015, to September 16, 2015. Cohort 2 enrolled an additional 89 eligible participants (69 CAB and 20 PBO) from December 18, 2015, to May 27, 2016. Overall, the median age was 31 years (interquartile range [IQR] 24, 40); 132 (66%) were female at birth, with most female participants enrolled in Malawi, South Africa, and the US. The study population included 6 transgender men and 1 transgender woman. For analysis purposes, participants were identified by sex at birth. Participants were 27% non-Hispanic white, 41% non-Hispanic black, 24% Hispanic/Latino, 3% Asian, and 6% mixed/other. The demographics were similar between the 2 cohorts and were not significantly different between Cohort 1 and Cohort 2 (Table 1).

**Table 1:** Demographics by cohort and treatment arm.

Characteristic	Cohort 1			Cohort 2			*p*-Value*
	Overall	CAB	PBO	Overall	CAB	PBO	
Age, median (IQR)	33 (25, 42)	30 (24, 41)	35 (27, 42)	31 (24, 37)	30 (23, 36)	33 (25, 39)	0.211
BMI, median (IQR)	27 (24, 32)	27 (24, 33)	26 (24, 32)	26 (23, 33)	26 (22, 32)	28 (24, 34)	0.256
Weight, median (IQR)	76 (67, 91)	78 (68, 94)	75 (66, 87)	73 (61, 93)	72 (60, 86)	74 (62, 100)	0.165
Sex at birth, *n* (%)							
Female	72 (65%)	54 (66%)	18 (64%)	60 (67%)	46 (67%)	14 (70%)	0.771
Male	38 (35%)	28 (34%)	10 (36%)	29 (33%)	23 (33%)	6 (30%)	
Sex at birth and region, *n* (%)							
Female							
US	32 (44%)	24 (44%)	8 (44%)	25 (42%)	19 (41%)	6 (43%)	0.637
Brazil	11 (15%)	8 (15%)	3 (17%)	13 (22%)	8 (17%)	5 (36%)	
Sub-Saharan Africa	29 (40%)	22 (41%)	7 (39%)	22 (37%)	19 (41%)	3 (21%)	
Male							
US	31 (82%)	23 (82%)	8 (80%)	18 (62%)	14 (61%)	4 (67%)	0.115
Brazil	5 (13%)	4 (14%)	1 (10%)	5 (17%)	4 (17%)	1 (17%)	
Sub-Saharan Africa	2 (5%)	1 (4%)	1 (10%)	6 (21%)	5 (22%)	1 (17%)	
Transgender, *n* (%)							
Female	0 (0%)	0 (0%)	0 (0%)	1 (1.7%)	1 (2.2%)	0 (0%)	1.000
Male	3 (7.9%)	3 (10.7%)	0 (0%)	3 (10.3%)	1 (4.3%)	2 (33.3%)	
Race/ethnicity, *n* (%)							
Non-Hispanic white	36 (33%)	29 (35%)	7 (25%)	18 (20%)	13 (19%)	5 (25%)	0.162
Non-Hispanic black	42 (38%)	31 (38%)	11 (39%)	40 (45%)	33 (48%)	7 (35%)	
Hispanic/Latino	24 (22%)	19 (23%)	5 (18%)	23 (26%)	17 (25%)	6 (30%)	
Asian	1 (1%)	0 (0%)	1 (4%)	4 (4%)	3 (4%)	1 (5%)	
Mixed/other	7 (6%)	3 (4%)	4 (14%)	4 (4%)	3 (4%)	1 (5%)	

*p-Value for the comparison between Cohort 1 and Cohort 2 participant characteristics.

CAB, cabotegravir; PBO, placebo.

Fig 2 shows participant disposition during the study. In Cohort 1, 90% of participants randomized to the CAB arm completed the oral phase and received at least 1 injection; 72% completed all 3 injections. In Cohort 2, 87% of participants in the CAB arm completed the oral phase; 80% completed the oral phase and received all 5 injections. Of those receiving at least 1 active CAB injection, 80% of Cohort 1 and 92% of Cohort 2 participants completed the full course of injections. There were no statistically significant differences between the CAB and PBO arms in the proportions of participants who entered the injection phase or completed all injection visits. Although injection course completion rates were numerically higher for males than for females in both cohorts (Fig 2; S1 Table), these differences were not statistically significant. Most germane to the phase 3 development program of CAB LA for HIV prevention, 95% of males and 90% of females in Cohort 2 who began the injection phase completed all 5 injections.

**Fig 3:**
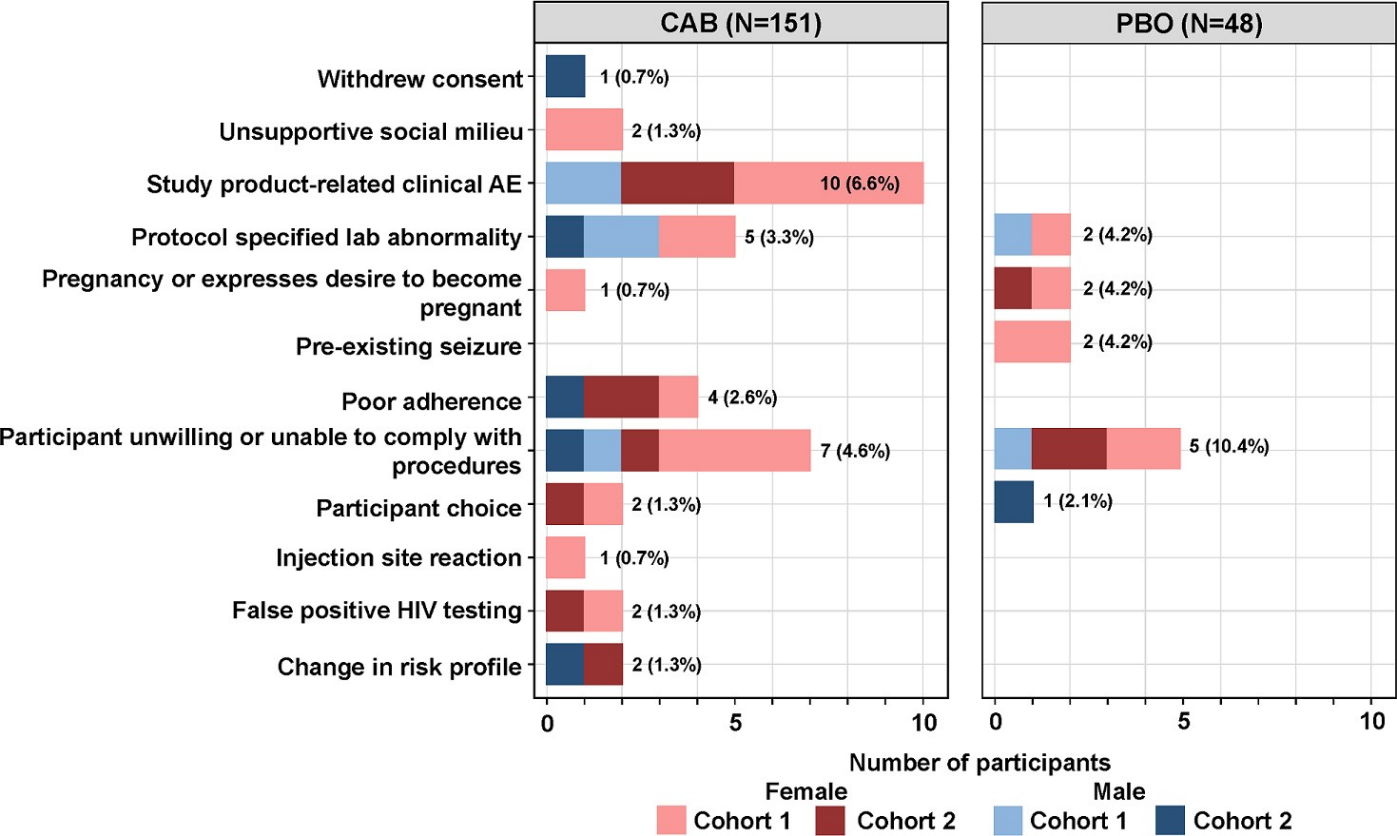
Study product discontinuation by cohort and arm (oral and injection phase). *n* = 199 (Cohort 1 CAB = 82; Cohort 1 PBO = 28; Cohort 2 CAB = 69; Cohort 2 PBO = 20). AE, adverse event; CAB, cabotegravir; PBO, placebo.

Forty-nine participants discontinued the injectable study product. The most common reasons for the discontinuation of study product (CAB and PBO) were unwillingness or inability to comply with study procedures (12 events), protocol-specified study-product-related clinical AEs (10 events), and protocol-specified lab abnormalities (7 events). Rates of study product discontinuation due to clinical or laboratory abnormalities were not different for males and females; product discontinuation rates were also not different between participants in the CAB and PBO arms (Fig 3; S2 Table). One participant in the CAB arm of Cohort 1 discontinued study product due to a Grade 2 ISR.

The 10 study product discontinuations (Fig 3; S2 Table) for clinical AEs among active CAB recipients included 1 gastrointestinal event (constipation), 3 rash/urticaria events, 6 neurologic events, including headaches (2), neuropathy (1), transient weakness (1), papilledema (1), and a seizure event in a participant with a preexisting history of seizure disorder (1). Four SAEs occurred in participants in CAB arms, including vertigo, transient weakness, laryngitis, and acute kidney injury; the latter 2 events were assessed by study site investigators and determined to be unrelated to study products. Two SAEs occurred in participants in the PBO arms, including 1 episode of cholelithiasis and 1 spontaneous loss of pregnancy at 11 weeks of gestation.

**Fig 4:**
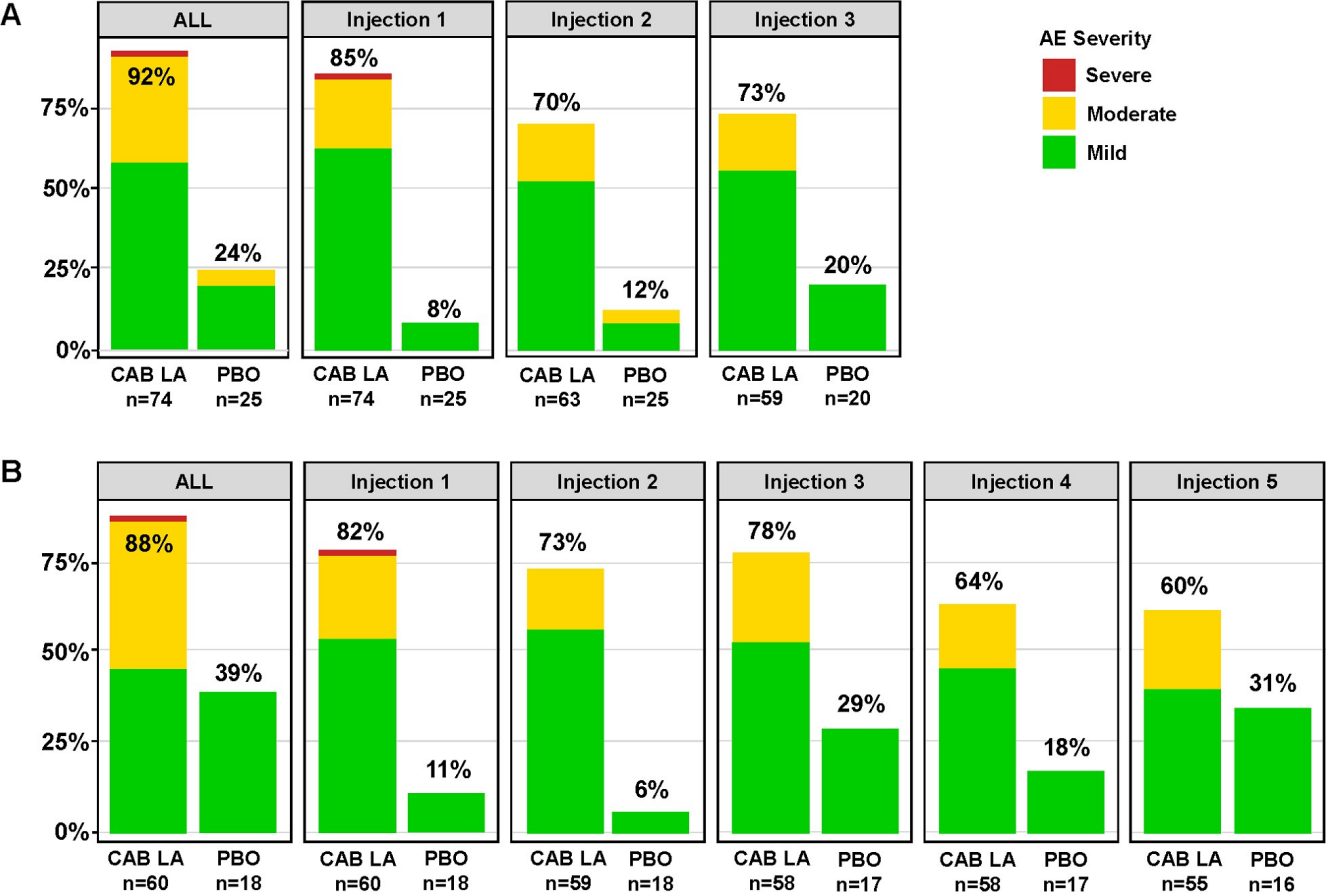
Proportion of participants reporting an injection site reaction resulting from each injection visit. (A) Proportion of Cohort 1 participants reporting any injection site reaction. (B) Proportion of Cohort 2 participants reporting any injection site reaction. AE, adverse event; CAB LA, long-acting cabotegravir; PBO, placebo.

Overall, 122 (91%) participants in the CAB arm reported at least 1 Grade 2 or higher AE during the injection phase, compared with 38 (88%) of participants in the PBO arm (*p =* 0.56; Table 2). The most commonly reported Grade 2 or higher AEs were decreased creatinine clearance by Cockcroft–Gault calculation, at similar rates in each arm (63 [47%] in the CAB arm and 20 [46.5%] in the PBO arm, *p =* 1.0; Table 2). The only Grade 2 or higher clinical AEs that were more common in the CAB arm compared to the PBO arm were ISRs (51 [38%] in the CAB arm and 1 [2%] in the PBO arm, *p* < 0.001; Table 2). ISRs of any grade, mostly Grade 1 (mild) to Grade 2 (moderate), were reported in the CAB arm by 92% of participants in Cohort 1 (Fig 4A) and 88% of participants in Cohort 2 (Fig 4B); ISRs of any grade were less frequent in the PBO arm (24% in Cohort 1 and 39% in Cohort 2). For the CAB arm overall, ISR events declined with subsequent injections; ISR event rates were similar for male and female participants receiving CAB across both cohorts (91% versus 90%, *p =* 1.0) (S1 Fig). One Grade 3 (severe) ISR occurred in a CAB recipient in each cohort; neither resulted in discontinuation of study products. No participant discontinued study products due to protocol-specified electrocardiographic changes.

**Table 2:** Grade 2 and higher AEs experienced by at least 5% of participants during the injection phase.

AE	CAB LA (*n* = 134)		PBO (*n* = 43)		*p*-Value*	
	*n* (%)	95% CI	*n* (%)	95% CI		
AE ≥ Grade 2	122 (91.0%)	85.0%, 94.8%	38 (88.4%)	75.5%, 94.9%	0.565	
Serious adverse event	4 (3.0%)	1.2%, 7.4%	2 (4.7%)	1.3%, 15.5%	0.634	
Creatinine renal clearance decreased^†^	63 (47.0%)	38.8%, 55.4%	20 (46.5%)	32.5%, 61.1%	1.000	
Injection site reaction^‡^	51 (38.1%)	30.3%, 46.5%	1 (2.3%)	0.4%, 12.1%	<0.001	
Musculoskeletal discomfort	34 (25.4%)	18.8%, 33.4%	6 (14.0%)	6.6%, 27.3%	0.145	
Upper respiratory infection	31 (23.1%)	16.8%, 31.0%	10 (23.3%)	13.0%, 37.7%	1.000	
Headache	22 (16.4%)	11.7%, 24.4%	4 (9.3%)	3.7%, 21.6%	0.326	
Hypoglycemia	15 (11.2%)	6.9%, 17.6%	3 (7.0%)	2.4%, 18.6%	0.568	
Influenza	14 (10.4%)	6.3%, 16.8%	3 (7.0%)	2.4%, 18.6%	0.766	
Blood creatinine increased	13 (9.7%)	5.8%, 15.9%	3 (7.0%)	2.4%, 18.6%	0.764	
Nasopharyngitis	14 (10.4%)	6.3%, 16.8%	2 (4.7%)	1.3%, 15.5%	0.364	
Lipase increased	10 (7.5%)	4.1%, 13.2%	4 (9.3%)	3.7%, 21.6%	0.747	
Conjunctivitis	11 (8.2%)	4.6%, 14.1%	1 (2.3%)	0.4%, 12.1%	0.298	
Gastroenteritis	10 (7.5%)	4.1%, 13.2%	2 (4.7%)	1.3%, 15.5%	0.733	
Urinary tract infection	11 (8.2%)	4.6%, 14.1%	1 (2.3%)	0.4%, 12.1%	0.298	
Blood creatine phosphokinase increased	6 (4.5%)	2.1%, 9.4%	5 (12%)	5.1%, 24.5%	0.139	
Rash	9 (6.7%)	4.1%, 13.2%	1 (2.3%)	0.4%, 12.1%	0.455	
Dermatitis	8 (6.0%)	3.1%, 11.3%	2 (4.7%)	1.3%, 15.5%	1.000	
Weight decreased	6 (4.5%)	2.1%, 9.4%	3 (7.0%)	2.4%, 18.6%	0.455	
Genital candidiasis	7 (5.2%)	2.6%, 10.4%	1 (2.3%)	0.4%, 12.1%	0.682	
Sinusitis	7 (5.2%)	2.6%, 10.4%	1 (2.3%)	0.4%, 12.1%	0.682	
Depression	0 (0.0%)	0.0%, 2.8%	3 (7.0%)	2.4%, 18.6%	0.014	

*p-Value for comparing the cabotegravir and PBO arms.

^†^Grade 2 AEs for creatinine clearance are determined by a result of <90 to 60 ml/min or a 10% to <30% decrease from baseline. Grade 3 AEs for creatinine clearance are determined by a result of <60 to 30 ml/min or a 30% to <50% decrease from baseline. Of these participants, 42 (51%) had a decrease from baseline but maintained a creatinine clearance ≥90 ml/min during the entire injection phase; 26 (31%) experienced a treatment-emergent decline in creatinine clearance to <90 ml/min. Creatinine clearance returned to >90 ml/min during the injection phase for 20 (80%) of the 26 participants without interruption of study products. Fourteen participants began the study with a Grade 2 creatinine clearance (<90 mm/min), which improved to >90 ml/min on study products, and then returned to <90 ml/min creatinine clearance. One participant in the PBO arm experienced a transient decline in creatinine clearance from 70 ml/min to 57 ml/min.

^‡^Injection site pain in 47/52 (90%).

CAB LA, long-acting cabotegravir; PBO, placebo.

Two pregnancies occurred in participants in the PBO arm of the study. One resulted in a miscarriage at 11 weeks, likely due to Zika virus infection, and 1 led to a full-term, healthy infant without apparent congenital anomalies. A third female participant became pregnant during the tail-phase of the study (after the period of primary analysis covered in this paper), 8 months after her fifth and final injection of active CAB. Her pregnancy was complicated by preeclampsia; however, she delivered a healthy, early-term (38 5/7 weeks) [24] child without birth defects.

One participant acquired HIV during the study: a female participant at a site in sub-Saharan Africa in Cohort 1, 48 weeks after the final injection of active CAB. The participant’s plasma CAB concentration was below the LLOQ (0.025 μg/ml) at the visit at which HIV infection was first detected, and also at a visit 12 weeks prior, when the participant had non-reactive/negative HIV tests with undetectable HIV RNA. HIV from this participant had no mutations associated with drug resistance in HIV reverse transcriptase, protease, or integrase assessed using an assay based on population sequencing. Results obtained using a research assay based on NGS are shown in S2 Text; no integrase mutations were detected. Seven incident STIs were diagnosed in 6 participants during the primary analysis period: rectal chlamydia (2 cases), urinary chlamydia (1 case), urinary gonorrhea (1 case), dual rectal and urinary gonorrhea (1 case), and early syphilis (1 case). All STIs except for syphilis were in female participants.

**Fig 5:**
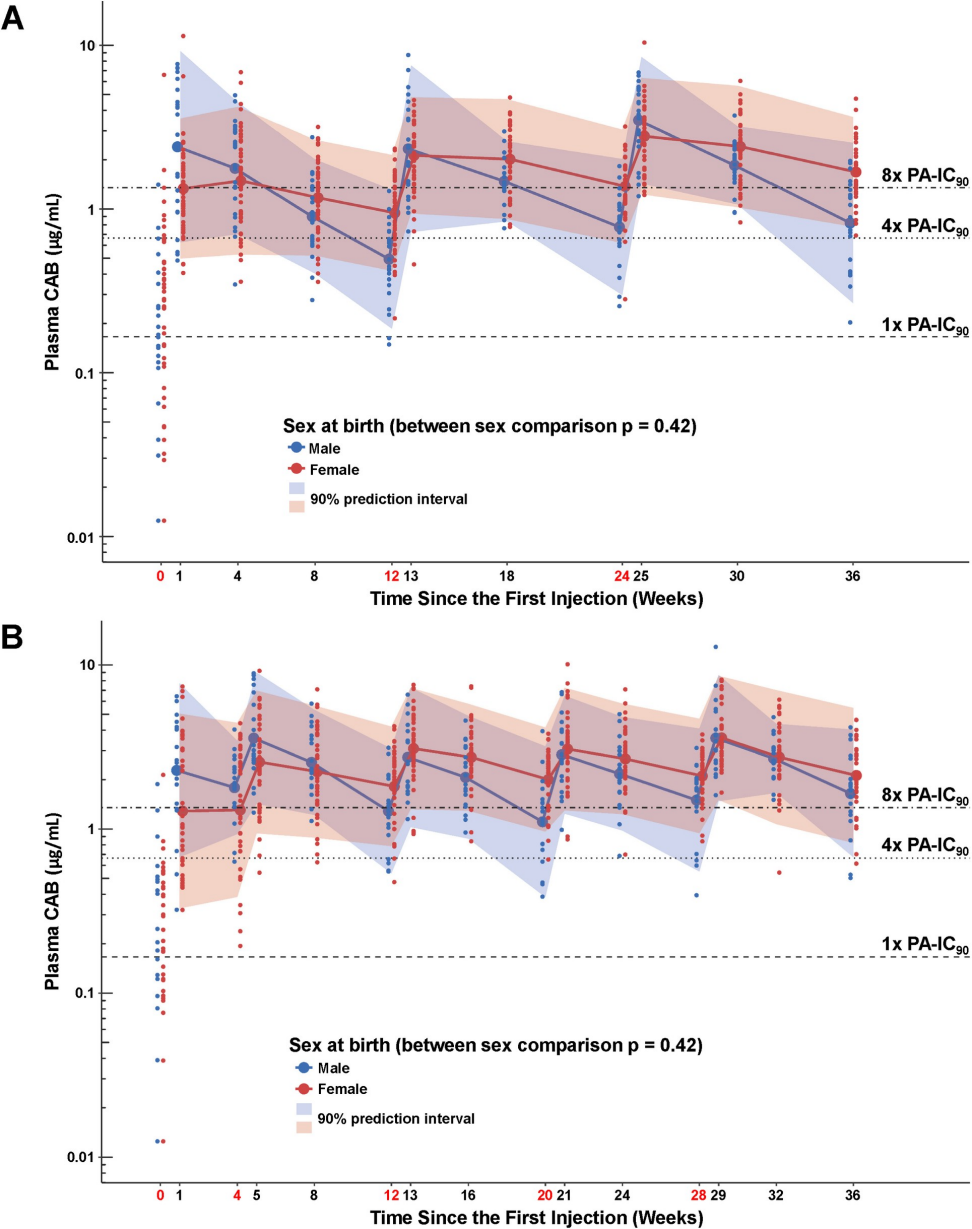
CAB concentrations by sex at birth and cohort. (A) Cohort 1 plasma CAB concentrations by sex at birth. (B) Cohort 2 plasma CAB concentrations by sex at birth. Shaded areas represent the 90% prediction interval for the model. Time points indicated in red denote visits at which injections were administered. Time 0 values represent plasma levels 1 week after last oral CAB dosing. PA-IC_90_ is the protein-adjusted concentration at which 90% inhibition of viral replication is achieved. CAB, cabotegravir.

CAB concentrations at all study visits, and the geometric means and their 90% prediction intervals by sex at birth and cohort, are shown in Fig 5 for visits during the injection phase of the study.

**Fig 6:**
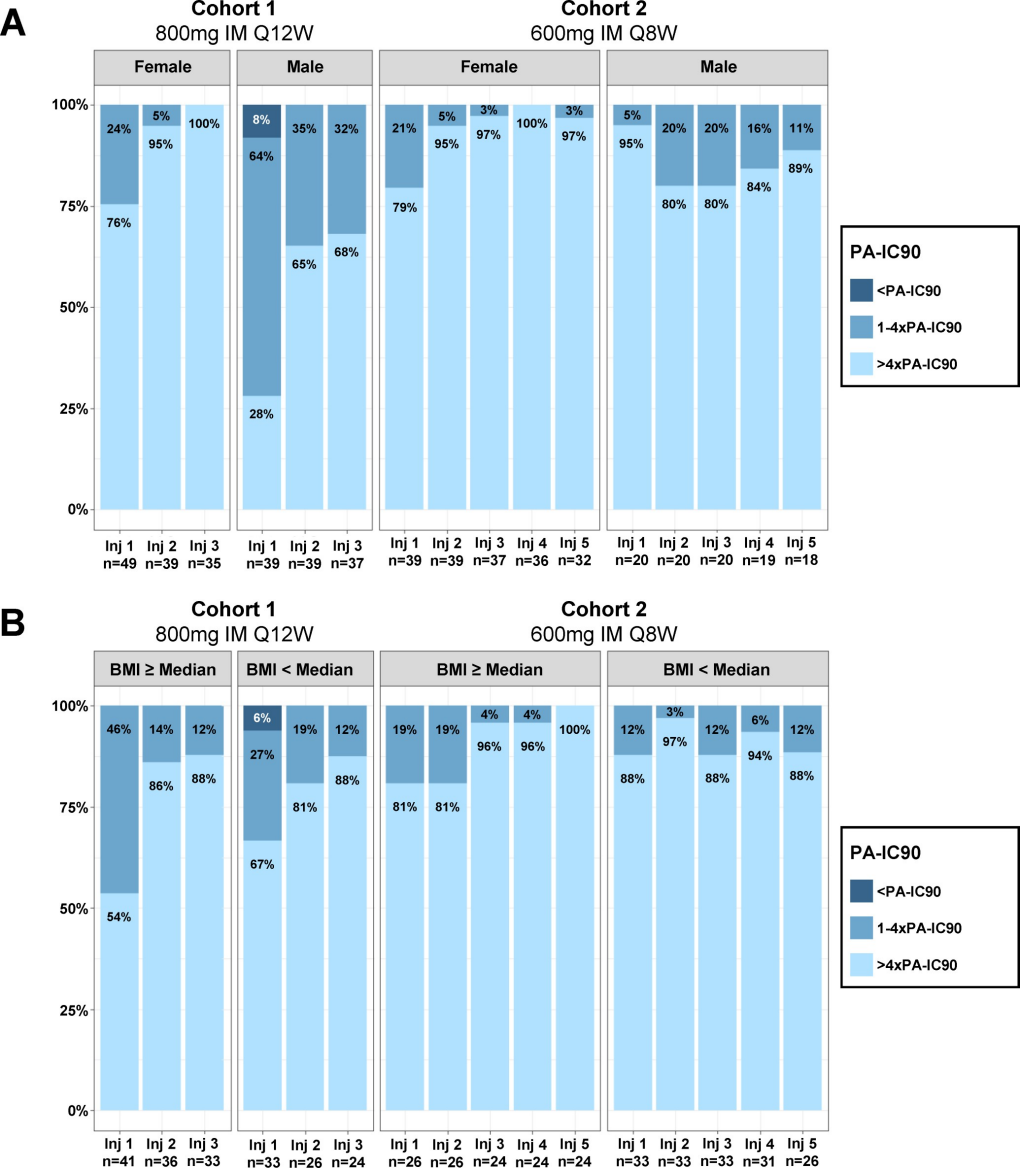
Distribution of *C*_τ_ CAB concentrations relative to PA-IC_90_ by cohort and sex at birth and cohort and BMI (≥median versus <median). (A) Distribution of *C*_τ_ CAB concentrations by cohort and sex at birth. (B) Distribution of *C*_τ_ CAB concentrations by cohort and BMI. PA-IC_90_ is the protein-adjusted concentration at which 90% inhibition of viral replication is achieved. BMI, body mass index; IM, intramuscular; Inj, injection; Q12W, every 12 weeks; Q8W, every 8 weeks.

In Cohort 1 (800 mg every 12 weeks), geometric mean *C*_max_ CAB concentrations were significantly higher in male than in female participants following the first injection, and *C*_τ_ concentrations were significantly lower in male than in female participants following all 3 injections (Table 3). AUC_0–τ_ was not significantly different between males and females. For males in Cohort 1, 72%, 35%, and 32% of participants had *C*_τ_ concentrations that fell below 4× PA-IC_90_ (0.166 μg/ml) at post-injection troughs 1, 2, and 3, respectively (Fig 6A, weeks 17, 29, and 41). For females in Cohort 1, 24%, 5%, and 0% of participants’ *C*_τ_ concentrations fell below 4× PA-IC_90_ at troughs 1, 2, and 3, respectively. Female participants’ *C*_τ_ CAB concentrations were uniformly above the PA-IC_90_ after the third injection.

**Table 3:** Geometric means of pharmacokinetic parameters (*C*_max_, *C*_τ_, and AUC_0–τ_) by sex at birth.

Injection	Parameter	Cohort 1			Cohort 2		
		Female	Male	*p*-Value	Female	Male	*p*-Value
1	*n*	49	25		39	20	
	*C*_max_ (μg/ml)	1.89	2.67	0.019	1.58	2.51	0.003
	*C*_τ_ (μg/ml)	0.95	0.49	<0.001	1.33	1.79	0.052
	AUC_0–τ_ (d*μg/ml)	108.86	118.06	0.470	32.16	51.15	0.001
2	*n*	39	23		39	20	
	*C*_max_ (μg/ml)	2.29	2.57	0.451	2.96	3.90	0.074
	*C*_τ_ (μg/ml)	1.35	0.78	<0.001	1.82	1.29	0.024
	AUC_0–τ_ (d*μg/ml)	152.86	133.24	0.254	125.05	146.59	0.264
3	*n*	35	22		37	20	
	*C*_max_ (μg/ml)	3.01	3.39	0.461	3.46	2.96	0.325
	*C*_τ_ (μg/ml)	1.65	0.82	<0.001	2.04	1.11	<0.001
	AUC_0–τ_ (d*μg/ml)	208.19	184.51	0.332	156.29	124.76	0.117
4	*n*				36	19	
	*C*_max_ (μg/ml)				3.33	2.96	0.460
	*C*_τ_ (μg/ml)				2.06	1.46	0.032
	AUC_0–τ_ (d*μg/ml)				149.04	121.57	0.166
5	*n*				32	18	
	*C*_max_ (μg/ml)				3.66	3.82	0.800
	*C*_τ_ (μg/ml)				2.03	1.68	0.259
	AUC_0–τ_ (d*μg/ml)				167.83	163.04	0.849

Includes plasma cabotegravir concentrations obtained from samples collected within defined post-injection sampling windows.

AUC_0–τ_, area under the plasma concentration–time curve over the dosing interval; *C*_max_, maximum plasma concentration; *C*_τ_, trough concentration at the end of the dosing interval.

In Cohort 2 (600 mg every 8 weeks after an initial 4-week interval), injections occurred at weeks 5, 9, 17, 25, and 33. With the first injection, male participants had significantly higher *C*_max_, *C*_τ_, and AUC_0–τ_ CAB concentrations than female participants (Table 3). However, *C*_τ_ CAB concentrations following the second, third, and fourth injections were significantly lower in male participants than in female participants, while *C*_max_ and AUC_0–τ_ values did not significantly differ between male and female participants. After the fifth injection, there were no significant differences between *C*_max_, *C*_τ_, and AUC_0–τ_ values in male and female participants. In Cohort 2, 79% or more of both male and female participants maintained *C*_τ_ CAB concentrations above 4× PA-IC_90_ after all injections (Fig 6A).

When BMI was dichotomized at or above versus below median by sex at birth, Cohort 1 participants with lower BMI had numerically higher *C*_max_ and AUC_0–τ_ concentrations than participants with higher BMI, which was statistically significant for the first injection only; *C*_τ_ concentrations were not different for any injection. In Cohort 2, participants with lower BMI also had higher *C*_max_ and AUC_0–τ_ concentrations with injections 1, 3 and 4; *C*_τ_ was also higher in those with lower BMI after injection 1 (Table 4; Fig 6B). The distribution of *C*_τ_ relative to PA-IC_90_ by BMI stratified by sex at birth is presented in S2 Fig. Results were similar when BMI was dichotomized using a cutoff of 25 or 30 kg/m^2^; a nonlinear inverse relationship appears to exist between BMI and pharmacokinetic parameters. This relationship is being explored further in ongoing analyses.

**Table 4:** Geometric means of pharmacokinetic parameters (*C*_max_, *C*_τ_, and AUC_0–τ_) by BMI (≥median versus <median).

Injection	Parameter	Cohort 1			Cohort 2		
		≥Median BMI*	<Median BMI*	*p*-Value	≥Median BMI*	<Median BMI*	*p*-Value
1	*n*	41	33		26	33	
	*C*_max_ (μg/ml)	1.82	2.56	0.014	1.35	2.37	<0.001
	*C*_τ_ (μg/ml)	0.73	0.81	0.454	1.12	1.82	0.001
	AUC_0–τ_ (d*μg/ml)	99.09	130.12	0.010	27.47	48.24	<0.001
2	*n*	36	26		26	33	
	*C*_max_ (μg/ml)	2.14	2.78	0.086	2.82	3.63	0.075
	*C*_τ_ (μg/ml)	1.10	1.09	0.946	1.40	1.82	0.085
	AUC_0–τ_ (d*μg/ml)	132.21	165.50	0.051	111.83	150.36	0.025
3	*n*	33	24		24	33	
	*C*_max_ (μg/ml)	2.93	3.48	0.272	2.57	3.90	0.005
	*C*_τ_ (μg/ml)	1.17	1.39	0.289	1.53	1.74	0.394
	AUC_0–τ_ (d*μg/ml)	181.07	225.81	0.065	113.68	171.86	0.002
4	*n*				24	31	
	*C*_max_ (μg/ml)				2.69	3.65	0.040
	*C*_τ_ (μg/ml)				1.85	1.81	0.887
	AUC_0–τ_ (d*μg/ml)				123.14	152.50	0.116
5	*n*				24	26	
	*C*_max_ (μg/ml)				3.27	4.18	0.112
	*C*_τ_ (μg/ml)				1.92	1.87	0.881
	AUC_0–τ_ (d*μg/ml)				153.49	178.63	0.284

Includes plasma cabotegravir concentrations obtained from samples collected within defined post-injection sampling windows.

*Median BMI is 27.2 kg/m^2^ for females and 25.0 kg/m^2^ for males. AUC_0–τ_, area under the plasma concentration–time curve over the dosing interval; BMI, body mass index; *C*_max_, maximum plasma concentration; *C*_τ_, trough concentration at the end of the dosing interval.

## Discussion

In HPTN 077, we administered 800 mg of CAB LA every 12 weeks (Cohort 1) or 600 mg of CAB LA every 8 weeks (after an initial 4-week injection interval; Cohort 2) or PBO to HIV-uninfected males and females in diverse geographic locations. This study builds on safety and pharmacokinetic findings of a previous phase 2a study of CAB LA in men in the US, using the 800 mg every 12 weeks dose only. We found that both doses/intervals were well tolerated. ISRs were very common, but infrequently led to injection discontinuation—at least with short-term injection sequences over 36 weeks. No other safety concerns were identified. Pharmacokinetics show that the 600 mg every 8 weeks dose used in Cohort 2 consistently met prespecified pharmacokinetic targets based on macaque models using a rectal SHIV challenge.

Long-acting antiretroviral drugs for HIV treatment and prevention are of tremendous interest, since they have the potential to minimize or eliminate some of the barriers associated with adherence to daily oral-tablet-based regimens. If proven safe and effective, the ability to use long-acting injectable drugs discretely and without daily dosing has the potential to significantly increase the proportion of at-risk individuals protected by antiretroviral-based prevention strategies. HPTN 077 characterized the safety, tolerability, and pharmacokinetics of CAB LA in males and females from regions disproportionally affected by the HIV epidemic. Importantly, we had the opportunity to test a 600 mg dose every 8 weeks, to study CAB LA in women, and to study CAB LA in people in sub-Saharan Africa and Brazil. CAB LA is currently in clinical development for both HIV treatment (in combination with long-acting rilpivirine for maintenance of virologic suppression) and HIV prevention (as a single agent). In HPTN 077, we defined the pharmacokinetic parameters of CAB LA in both males and females at 8- and 12-week dosing intervals while demonstrating the tolerability and safety of this agent in a low-risk, HIV-uninfected population.

In the current study, CAB LA was generally well tolerated, with limited laboratory abnormalities. The only statistically significant difference in AEs between CAB and PBO was for injection site pain. Although ISRs were common in participants receiving active CAB (approximately 90% of participants in both cohorts), only 1 participant discontinued injections because of an ISR. Two participants (1 in each cohort) who experienced severe (Grade 3) ISRs with their first injection did not discontinue injections; both participants completed the full series of injections. Although not reaching statistical significance, there appeared to be a numerical decrease in reports of ISRs with repeated injections in both dosing cohorts. While the difference in rates of ISRs between CAB LA and PBO recipients is striking, the majority of these reactions were mild or moderate and not treatment-limiting. Injection site tolerability will be important to monitor carefully in phase 3 studies and, if CAB LA is shown to be safe and effective for HIV prevention, in future open-label demonstration activities and research. While it is notable that 20% of all Cohort 1 participants and 8% of all Cohort 2 participants who began the injection phase did not complete the relatively short follow-up (Fig 2), the majority of the discontinuations were due to rigorous mandated clinical and laboratory stopping rules operationalized at regulatory behest, in order to be cautious with an investigational long-acting, novel chemical entity under evaluation in low-risk, HIV-uninfected individuals.

One HIV infection occurred, 48 weeks after the third and final injection of 800 mg of CAB LA. In this participant, CAB concentrations were below the limit of quantitation both at the seroconversion visit and at the visit 12 weeks prior to seroconversion, when HIV tests indicated that the participant was not HIV infected. This suggests that the exposure and HIV infection occurred in the setting of undetectable CAB concentrations. HIV resistance testing using both population sequencing and NGS did not demonstrate the selection of integrase-associated mutations. The participant’s CAB concentrations during the injection period were consistent with those of other participants in the Cohort 1 study population.

HPTN 077 was designed to supplement data from the previous phase 2a study of CAB LA in US-based males [21] by providing multi-dose pharmacokinetic data for CAB LA in females. During the conduct of HPTN 077 Cohort 1, it became clear from the earlier study that males who received 800 mg every 12 weeks did not consistently achieve the PA-IC_90_ goals. Cohort 2 was therefore enrolled to provide data on the 600 mg every 8 weeks dose in both males and females. Follow-up of participants in Cohorts 1 and 2 was also extended to better characterize CAB LA tail-phase pharmacokinetics.

HPTN 077 provides confirmatory data that males who receive CAB LA at a dose of 800 mg every 12 weeks do not consistently achieve target trough concentrations. Females who received the 800 mg quarterly dose fared better, only failing to meet pharmacokinetic targets at the first trough time point (79% of trough concentrations were greater than 4× PA-IC_90_). In contrast, all participants dosed with 600 mg every 8 weeks met the targets of 80% and 95% of participants with trough concentrations above 4× and 1× PA-IC_90_, respectively. Participants with lower BMI were found to generally exhibit higher pharmacokinetic peak concentrations after injection, as well as increased AUC concentrations. These BMI-related higher pharmacokinetic peaks were similar to findings in the previous male-only phase 2a study of CAB LA [21], but here were noted to be recapitulated for female participants. The mechanism and significance of these differences with respect to preventive efficacy will be evaluated in phase 3 clinical trials. It is possible that local injection site fat content and distribution may be contributing to altered pharmacokinetics, which will have important implications for other large-muscle injection options. Currently, CAB LA is only recommended for injection in the buttock region (gluteus medius or gluteus maximus). Should regulatory approvals be successful, there is interest in expanding injection site options, including the potential for anterior thigh self-injection; however, the suggestion of muscle fat-content related pharmacokinetic differences will obligate additional clinical evaluation before the use of alternative sites can be sanctioned. It is reassuring that macaque studies of SHIV challenge breakthrough infections did not result in resistant virus.

In an earlier phase 2a study in US males, CAB levels were still detectable at 52 weeks post-injection in 17% of participants [21]. HPTN 077 tail-phase pharmacokinetic data are not yet available. Pharmacokinetic data from the long-term follow-up of study participants were collected for up to 76 weeks after final injection. The description of these tail-phase kinetics in males and females (for whom the terminal decay rate is anticipated to be 50% slower than for males) will be important to the discussion of the risk for seroconversion and subsequent selection of CAB-resistant virus. This is of particular concern should HIV seroconversion occur during the period of CAB persistence below some critical, as yet to be determined threshold.

This study has several limitations. The sample size in each cohort may have limited our ability to detect rare AEs and to find smaller safety differences between cohorts and between CAB and PBO study arms. Assessment of ISRs between safety visits was limited to self-report and may have been influenced by recall bias when participants presented to the clinic. Sexual risk and adherence to oral study product were assessed by self-report and pill count, which are subject to social desirability bias and manipulation, respectively. Stringent stopping criteria in both the oral and injection phases of the study likely mandated more discontinuations than would be undertaken in either a phase 3 study or clinical practice. The limited number of transgender participants enrolled precludes robust subset analyses in this population; appropriately powered studies for transgender individuals are needed. Additionally, this low-risk population is not the intended target population for PrEP; some findings, such as tolerability and acceptability, may differ in meaningful ways from findings in those who are at higher risk of HIV infection and will be examined in phase 3 studies. Finally, the safety of CAB in women of childbearing potential will be of critical importance when considering the scalability of CAB LA PrEP for global use, even if proven effective in clinical studies. An ongoing phase 3 clinical trial enrolling women of childbearing potential will further define its safety profile in this population.

Other long-acting or extended release agents under investigation for HIV prevention include a nanosuspension of rilpivirine [25], broadly neutralizing monoclonal antibodies [26], subdermal implants that would elute agents from a removable or degradable depot (similar to implantable contraception) [27], and vaginal rings impregnated with dapivirine [28,29].

HPTN 077 demonstrated the safety and favorable pharmacokinetics for CAB LA at a dose of 600 mg intramuscularly administered every 8 weeks after an initial 4-week dosing interval in a global population of males and females. These results support the CAB LA dose selected for the current designs of the phase 3 studies HPTN 083 and HPTN 084 (NCT02720094 and NCT03164564 [https://www.hptn.org/research/studies]). These fully powered studies in high-risk populations are directly comparing the efficacy and safety of long-acting injectable CAB to standard of care daily oral TDF/emtricitabine in North and South America, Africa, and Asia.

## Supporting information

S1 CONSORT Checklist (DOCX)

S1 Data Datasets. (ZIP)

S1 Fig Pattern of ISR reports over injection sequences categorized by initial severity. Cohort 1 (800 mg IM every 12 weeks) (A) and Cohort 2 (600 mg IM every 8 weeks after a 4-week initial interval) (B).
(TIF)

S2 Fig Distribution of *C*_τ_ relative to PA-IC_90_ by BMI stratified by sex at birth. (TIF)

S1 Table All Grade 2 and higher AEs experienced by participants during the injection phase. (DOCX)

S2 Table Study product discontinuation by cohort and arm (oral and injection phase). (DOCX)

S1 Text Protocol. (PDF)

S2 Text NGS methods. (DOCX)

## Abbreviations

AE: adverse event
BMI: body mass index
CAB: cabotegravir
CAB LA: long-acting cabotegravir
FDA: US Food and Drug Administration
HPTN 077: HIV Prevention Trials Network study 077
IM: intramuscular
IQR: interquartile range
ISR: injection site reaction
LLOQ: lower limit of quantification
NGS: next generation sequencing
NHP: nonhuman primate
PBO: placebo
PrEP: pre-exposure prophylaxis
SAE: serious adverse event
SHIV: simian/human immunodeficiency virus
STI: sexually transmitted infection
TDF: tenofovir disoproxil fumarate
