# Supplementary material for: Safety, tolerability, and pharmacokinetics of long-acting injectable cabotegravir in low-risk HIV-uninfected individuals: HPTN 077, a phase 2a randomized controlled trial
Source: PLoS Med. 2018 Nov 8;15(11):e1002690. doi: 10.1371/journal.pmed.1002690 (PMC6224042; doi:10.1371/journal.pmed.1002690)
Supplement: S1 Data — (ZIP) [file pmed.1002690.s002.zip › d_aeinjgr2_data_dictionary.docx]

| **Variable Names** | **Format** | **Description** |
| --- | --- | --- |
| Uid |  | Participant id |
| Revised_AEMDRA |  | AE MedDRA code (adjudicated) |
| AE Page Number |  | AE Page Number on CRF |
| 2.Onset Date |  |  |
| 3.Severity | 1=”Mild”  2=”Moderate”  3=”Severe”  4=”Potentially life-threatening”  5=”Death” | AE severity grade |
| 8.SAE according to ICH? | 1=”Yes”  2=”No” |  |
| 11.Reprtd rslt site reac | 1=”Yes”  2=”No” | Is this an injection site reaction? |
| Arm | 1=”CAB”  2=”Placebo” |  |
| Cohort | 1=”Cohort 1”  2=”Cohort 2” |  |
