# Supplementary material for: Safety, tolerability, and pharmacokinetics of long-acting injectable cabotegravir in low-risk HIV-uninfected individuals: HPTN 077, a phase 2a randomized controlled trial
Source: PLoS Med. 2018 Nov 8;15(11):e1002690. doi: 10.1371/journal.pmed.1002690 (PMC6224042; doi:10.1371/journal.pmed.1002690)
Supplement: S1 Data — (ZIP) [file pmed.1002690.s002.zip › d_demo_data_dictionary.docx]

| **Variable Names** | **Format** | **Description** |  |
| --- | --- | --- | --- |
| Uid |  | Participant id | |
| race | 1="Latino"  2="Non-hispanic Asian"  3="Non-hispanic Black"  4="Non-hispanic White"  5="Non-hispanic mixed/other"  ; | Race | |
| Age at Enrollment |  | Age | |
| cohort | 1="Cohort 1"  2="Cohort 2"  ; | 077 study cohort | |
| bmi |  | BMI (kg/m^2^) |  |
| arm | 1="CAB"  2="Placebo"  ; | Study Arm |  |
| Latino/a or Hispanic |  | If participant is Hispanic or not. |  |
| Sex at birth | 1= “Male”  2= “Female” | Sex at birth | |
| Self-ID gender | 1=”male”  2=”female”  3=”other” | Self-identified gender |  |
| Weight |  | Weight (kg) |  |
| Region | 1= “US”  2= “Non-US” | US vs. non-US region |  |
| transgender | 1=”Male”  2=”Female” | Derived variable. If self-identified gender is different than sex at birth, then transgender is the opposite of sex at birth. |  |
