# Supplementary material for: Safety, tolerability, and pharmacokinetics of long-acting injectable cabotegravir in low-risk HIV-uninfected individuals: HPTN 077, a phase 2a randomized controlled trial
Source: PLoS Med. 2018 Nov 8;15(11):e1002690. doi: 10.1371/journal.pmed.1002690 (PMC6224042; doi:10.1371/journal.pmed.1002690)
Supplement: S1 Data — (ZIP) [file pmed.1002690.s002.zip › d_isr_inj_data_dictionary.docx]

| **Variable Names** | **Format** | **Description** |
| --- | --- | --- |
| Uid |  |  |
| cohort | 1=”Cohort 1”  2=”Cohort 2” |  |
| arm | 1=”CAB”  2=”Placebo” |  |
| gender | 1=”Male”  2=”Female |  |
| 3.Severity | 1=”Mild”  2=”Moderate”  3=”Severe”  4=”Potentially life-threatening”  5=”Death” |  |
| isr_byinj |  | Number of Injections received during the time around the injection site reaction observed |
| MedDRA text Preferred |  | AE MedDRA code |
|  |  |  |
|  |  |  |
|  |  |  |
|  |  |  |
|  |  |  |
|  |  |  |
|  |  |  |
