# Supplementary material for: Safety, tolerability, and pharmacokinetics of long-acting injectable cabotegravir in low-risk HIV-uninfected individuals: HPTN 077, a phase 2a randomized controlled trial
Source: PLoS Med. 2018 Nov 8;15(11):e1002690. doi: 10.1371/journal.pmed.1002690 (PMC6224042; doi:10.1371/journal.pmed.1002690)
Supplement: S1 Data — (ZIP) [file pmed.1002690.s002.zip › d_pk_data_dictionary.docx]

| **Variable Names** | **Format** | **Description** |
| --- | --- | --- |
| Uid |  |  |
| cohort |  |  |
| Visit Code |  | Numeric visit code |
| visitc |  | Weeks corresponding to visit code |
| Route of dosing |  |  |
| 8.SAE according to ICH? |  |  |
| Sex at birth | 1=”Male”  2=”Female” |  |
| BMI at enrollment |  |  |
| Drug |  |  |
| Sample Volume from LDMS |  |  |
| Drug concentration |  |  |
| Drug concentration units |  |  |
| LDMS censor code | (B) or B means non-detectable | Indicates if a variable is non-detectable |
| Lower limit of detection (NG/ML) |  |  |
|  |  |  |
