# Supplementary figures and images for: Safety, tolerability, and pharmacokinetics of long-acting injectable cabotegravir in low-risk HIV-uninfected individuals: HPTN 077, a phase 2a randomized controlled trial
Source: PLoS Med. 2018 Nov 8;15(11):e1002690. doi: 10.1371/journal.pmed.1002690 (PMC6224042; doi:10.1371/journal.pmed.1002690)

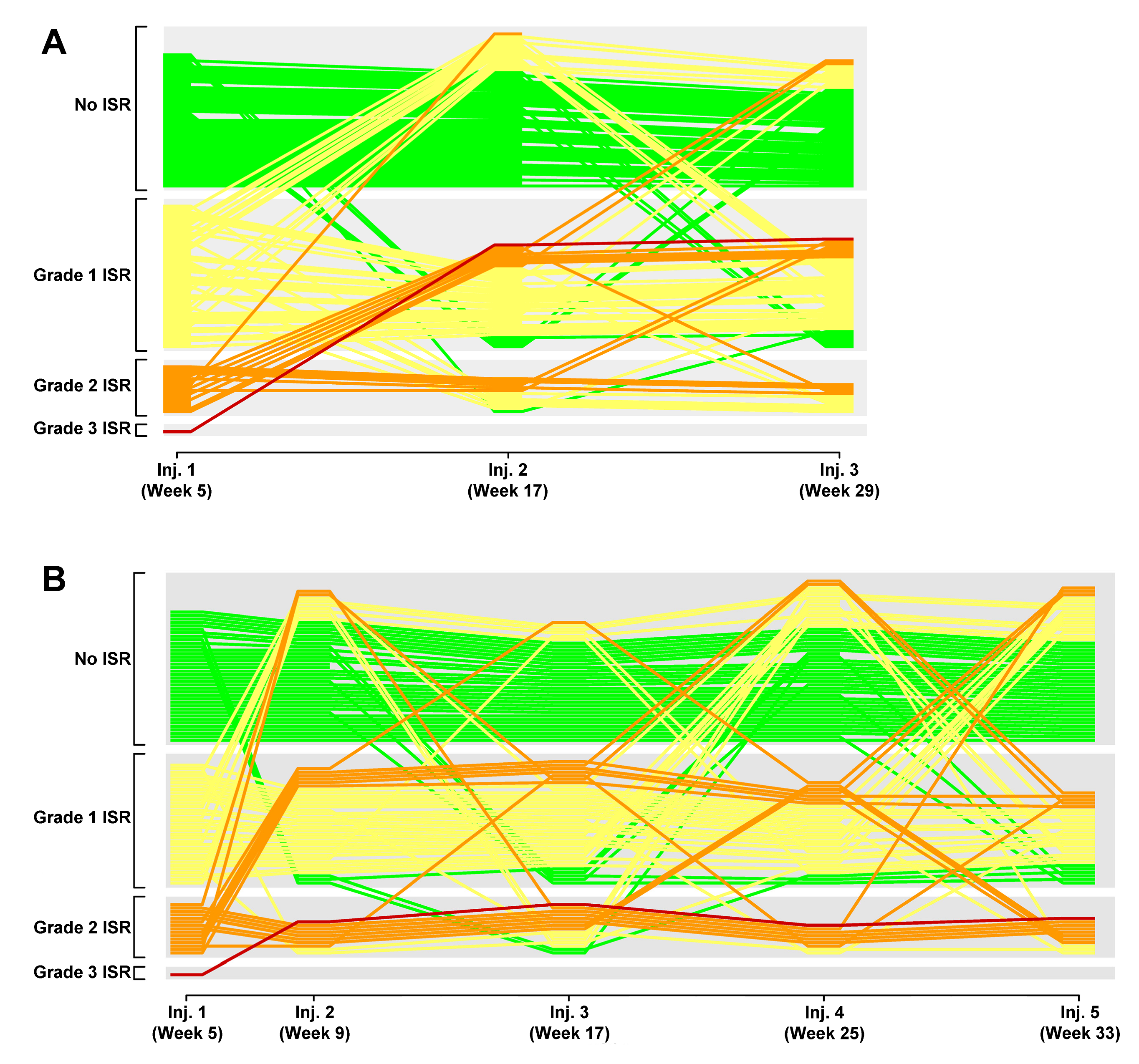

Supplement: S1 Fig — Cohort 1 (800 mg IM every 12 weeks) (A) and Cohort 2 (600 mg IM every 8 weeks after a 4-week initial interval) (B). (TIF) [file pmed.1002690.s003.tif]

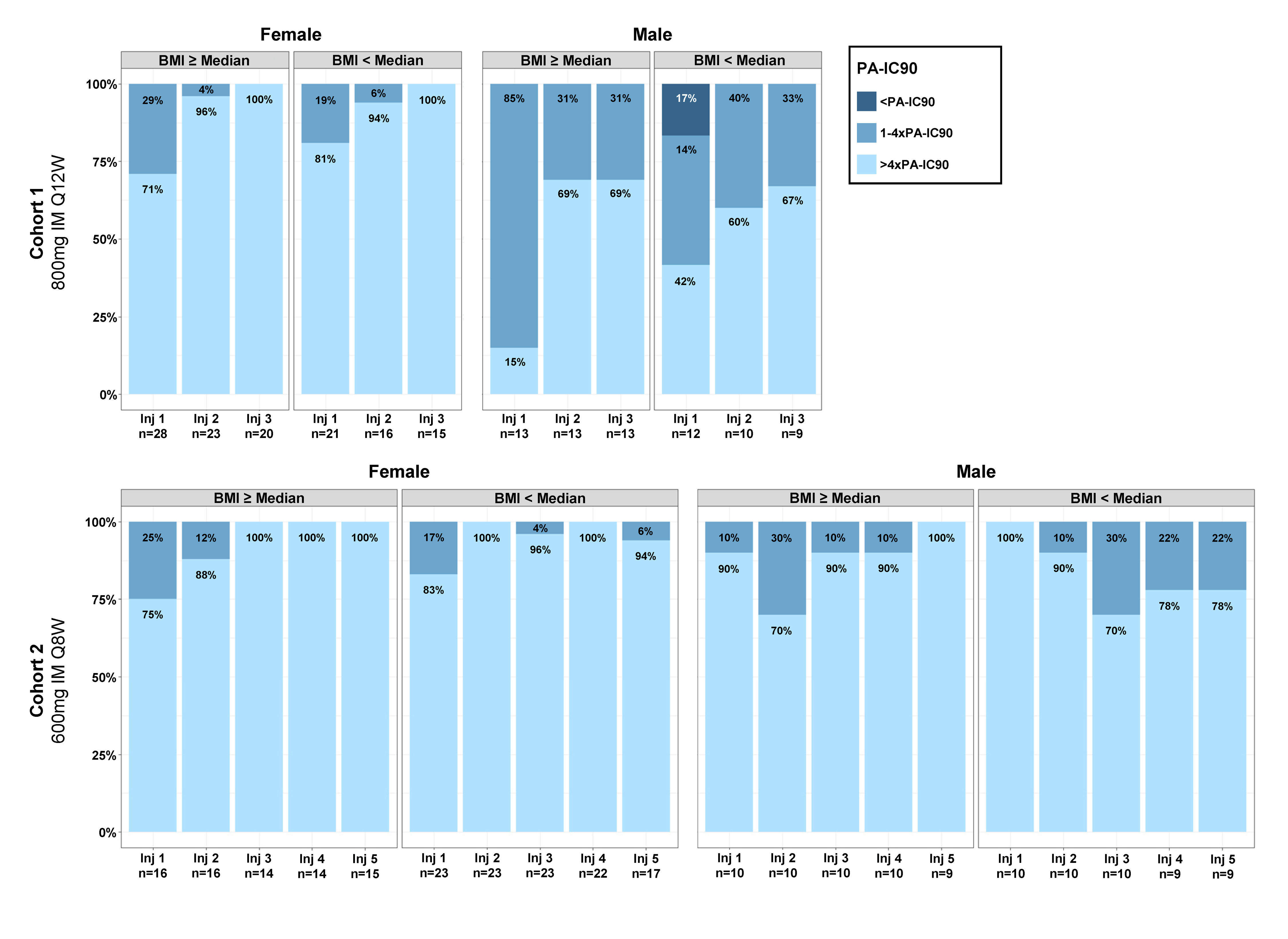

Supplement: S2 Fig — (TIF) [file pmed.1002690.s004.tif]
