# Supplementary material for: Safety, tolerability, and pharmacokinetics of long-acting injectable cabotegravir in low-risk HIV-uninfected individuals: HPTN 077, a phase 2a randomized controlled trial
Source: PLoS Med. 2018 Nov 8;15(11):e1002690. doi: 10.1371/journal.pmed.1002690 (PMC6224042; doi:10.1371/journal.pmed.1002690)
Supplement: S1 Table — (DOCX) [file pmed.1002690.s005.docx]

**S1 Table. All grade 2 and higher adverse events experienced by participants during the injection phase**

|  | **CAB LA (N=134)** | | **Placebo (N=43)** | |  |
| --- | --- | --- | --- | --- | --- |
|  | **N (%)** | **95% CI** | **N (%)** | **95% CI** | **P-value** |
| Adverse Event ≥ Grade 2 | **122 (91.0%)** | **(85.0%, 94.8%)** | **38 (88.4%)** | **(75.5%, 94.9%)** | **0.565** |
| Any Serious Adverse Event | 4 (3.0%) | (1.2%, 7.4%) | 2 (4.7%) | (1.3%, 15.5%) | 0.634 |
|  | | | | | |
| Creatinine renal clearance decreased | 63 (47.0%) | (38.8%, 55.4%) | 20 (46.5%) | (32.5%, 61.1%) | 1.0000 |
| Injection Site Reaction | 51 (38.1%) | (30.3%, 46.5%) | 1 (2.3%) | (0.4%, 12.1%) | <.0001 |
| Upper Respiratory Infection | 31 (23.1%) | (16.8%, 31.0%) | 10 (23.3%) | (13.2%, 37.7%) | 1.000 |
| Musculoskeletal discomfort | 34 (25.4%) | (18.8%, 33.4%) | 6 (14.0%) | (6.6%, 27.3%) | 0.145 |
| Headache | 22 (16.4%) | (11.1%, 23.6%) | 4 (9.3%) | (3.7%, 21.6%) | 0.326 |
| Hypoglycaemia | 15 (11.2%) | (6.9%, 17.6%) | 3 (7.0%) | (2.4%, 18.6%) | 0.568 |
| Influenza | 14 (10.4%) | (6.3%, 16.8%) | 3 (7.0%) | (2.4%, 18.6%) | 0.766 |
| Blood creatinine increased | 13 (9.7%) | (5.8%, 15.9%) | 3 (7.0%) | (2.4%, 18.6%) | 0.764 |
| Nasopharyngitis | 14 (10.4%) | (6.3%, 16.8%) | 2 (4.7%) | (1.3%, 15.5%) | 0.364 |
| Lipase increased | 10 (7.5%) | (4.1%, 13.2%) | 4 (9.3%) | (3.7%, 21.6%) | 0.747 |
| Conjunctivitis | 11 (8.2%) | (4.6%, 14.1%) | 1 (2.3%) | (0.4%, 12.1%) | 0.298 |
| Gastroenteritis | 10 (7.5%) | (4.1%, 13.2%) | 2 (4.7%) | (1.3%, 15.5%) | 0.733 |
| UTI | 11 (8.2%) | (4.6%, 14.1%) | 1 (2.3%) | (0.4%, 12.1%) | 0.298 |
| Blood creatine phosphokinase increased | 6 (4.5%) | (2.1%, 9.4%) | 5 (11.6%) | (5.1%, 24.5%) | 0.139 |
| Dermatitis | 8 (6.0%) | (3.1%, 11.3%) | 2 (4.7%) | (1.3%, 15.5%) | 1.000 |
| Rash | 9 (6.7%) | (3.6%, 12.3%) | 1 (2.3%) | (0.4%, 12.1%) | 0.455 |
| Weight Decreased | 6 (4.5%) | (2.1%, 9.4%) | 3 (7.0%) | (2.4%, 18.6%) | 0.455 |
| Diarrhea | 6 (4.5%) | (2.1%, 9.4%) | 2 (4.7%) | (1.3%, 15.5%) | 1.000 |
| Genital candidiasis | 7 (5.2%) | (2.6%, 10.4%) | 1 (2.3%) | (0.4%, 12.1%) | 0.682 |
| Hyperglycaemia | 6 (4.5%) | (2.1%, 9.4%) | 2 (4.7%) | (1.3%, 15.5%) | 1.000 |
| Sinusitis | 7 (5.2%) | (2.6%, 10.4%) | 1 (2.3%) | (0.4%, 12.1%) | 0.682 |
| Abdominal pain | 6 (4.5%) | (2.1%, 9.4%) | 0 (0.0%) | (0.0%, 8.2%) | 0.338 |
| Bilirubin conjugated increased | 6 (4.5%) | (2.1%, 9.4%) | 0 (0.0%) | (0.0%, 8.2%) | 0.338 |
| Allergic rhinitis | 3 (2.2%) | (0.8%, 6.4%) | 2 (4.7%) | (1.3%, 15.5%) | 0.596 |
| Acne | 3 (2.2%) | (0.8%, 6.4%) | 1 (2.3%) | (0.4%, 12.1%) | 1.000 |
| Dysfunctional uterine bleeding | 4 (3.0%) | (1.2%, 7.4%) | 0 (0.0%) | (0.0%, 8.2%) | 0.573 |
| Fatigue | 4 (3.0%) | (1.2%, 7.4%) | 0 (0.0%) | (0.0%, 8.2%) | 0.573 |
| Injury | 4 (3.0%) | (1.2%, 7.4%) | 0 (0.0%) | (0.0%, 8.2%) | 0.573 |
| Insomnia | 2 (1.5%) | (0.4%, 5.3%) | 2 (4.7%) | (1.3%, 15.5%) | 0.249 |
| Pruritus | 4 (3.0%) | (1.2%, 7.4%) | 0 (0.0%) | (0.0%, 8.2%) | 0.573 |
| Pyrexia | 4 (3.0%) | (1.2%, 7.4%) | 0 (0.0%) | (0.0%, 8.2%) | 0.573 |
| Reflux | 2 (1.5%) | (0.4%, 5.3%) | 2 (4.7%) | (1.3%, 15.5%) | 0.249 |
| Urticaria | 3 (2.2%) | (0.8%, 6.4%) | 1 (2.3%) | (0.4%, 12.1%) | 1.000 |
| Vaginal discharge | 4 (3.0%) | (1.2%, 7.4%) | 0 (0.0%) | (0.0%, 8.2%) | 0.573 |
| Anxiety | 2 (1.5%) | (0.4%, 5.3%) | 1 (2.3%) | (0.4%, 12.1%) | 0.569 |
| Aspartate aminotransferase increased | 2 (1.5%) | (0.4%, 5.3%) | 1 (2.3%) | (0.4%, 12.1%) | 0.569 |
| Blood magnesium decreased | 3 (2.2%) | (0.8%, 6.4%) | 0 (0.0%) | (0.0%, 8.2%) | 1.000 |
| Constipation | 2 (1.5%) | (0.4%, 5.3%) | 1 (2.3%) | (0.4%, 12.1%) | 0.569 |
| Depression | 0 (0.0%) | (0.0%, 2.8%) | 3 (7.0%) | (2.4%, 18.6%) | 0.014 |
| Dizziness/Vertigo | 3 (2.2%) | (0.8%, 6.4%) | 0 (0.0%) | (0.0%, 8.2%) | 1.000 |
| Elevated Blood Pressure | 2 (1.5%) | (0.4%, 5.3%) | 1 (2.3%) | (0.4%, 12.1%) | 0.569 |
| Hypophosphataemia | 2 (1.5%) | (0.4%, 5.3%) | 1 (2.3%) | (0.4%, 12.1%) | 0.569 |
| Alanine aminotransferase increased | 1 (0.7%) | (0.1%, 4.1%) | 1 (2.3%) | (0.4%, 12.1%) | 0.428 |
| Amylase increased | 2 (1.5%) | (0.4%, 5.3%) | 0 (0.0%) | (0.0%, 8.2%) | 1.000 |
| Angioedema | 2 (1.5%) | (0.4%, 5.3%) | 0 (0.0%) | (0.0%, 8.2%) | 1.000 |
| Bacterial vaginosis | 1 (0.7%) | (0.1%, 4.1%) | 1 (2.3%) | (0.4%, 12.1%) | 0.428 |
| Blood bilirubin increased | 1 (0.7%) | (0.1%, 4.1%) | 1 (2.3%) | (0.4%, 12.1%) | 0.428 |
| Blood calcium decreased | 2 (1.5%) | (0.4%, 5.3%) | 0 (0.0%) | (0.0%, 8.2%) | 1.000 |
| Cellulitis | 2 (1.5%) | (0.4%, 5.3%) | 0 (0.0%) | (0.0%, 8.2%) | 1.000 |
| Chest pain | 2 (1.5%) | (0.4%, 5.3%) | 0 (0.0%) | (0.0%, 8.2%) | 1.000 |
| Chlamydial infection | 2 (1.5%) | (0.4%, 5.3%) | 0 (0.0%) | (0.0%, 8.2%) | 1.000 |
| Electrocardiogram QT prolonged | 2 (1.5%) | (0.4%, 5.3%) | 0 (0.0%) | (0.0%, 8.2%) | 1.000 |
| Genitourinary tract gonococcal infection | 2 (1.5%) | (0.4%, 5.3%) | 0 (0.0%) | (0.0%, 8.2%) | 1.000 |
| Gingivitis | 1 (0.7%) | (0.1%, 4.1%) | 1 (2.3%) | (0.4%, 12.1%) | 0.428 |
| Haemoglobin decreased | 1 (0.7%) | (0.1%, 4.1%) | 1 (2.3%) | (0.4%, 12.1%) | 0.428 |
| Infected bite | 2 (1.5%) | (0.4%, 5.3%) | 0 (0.0%) | (0.0%, 8.2%) | 1.000 |
| Infection parasitic | 2 (1.5%) | (0.4%, 5.3%) | 0 (0.0%) | (0.0%, 8.2%) | 1.000 |
| Low density lipoprotein increased | 1 (0.7%) | (0.1%, 4.1%) | 1 (2.3%) | (0.4%, 12.1%) | 0.428 |
| Nausea | 2 (1.5%) | (0.4%, 5.3%) | 0 (0.0%) | (0.0%, 8.2%) | 1.000 |
| Oral herpes | 2 (1.5%) | (0.4%, 5.3%) | 0 (0.0%) | (0.0%, 8.2%) | 1.000 |
| Oropharyngeal pain | 2 (1.5%) | (0.4%, 5.3%) | 0 (0.0%) | (0.0%, 8.2%) | 1.000 |
| Pelvic inflammatory disease | 2 (1.5%) | (0.4%, 5.3%) | 0 (0.0%) | (0.0%, 8.2%) | 1.000 |
| Vomiting | 1 (0.7%) | (0.1%, 4.1%) | 1 (2.3%) | (0.4%, 12.1%) | 0.428 |
| Abortion spontaneous | 0 (0.0%) | (0.0%, 2.8%) | 1 (2.3%) | (0.4%, 12.1%) | 0.243 |
| Acute Kidney Injury | 1 (0.7%) | (0.1%, 4.1%) | 0 (0.0%) | (0.0%, 8.2%) | 1.000 |
| Blood calcium increased | 1 (0.7%) | (0.1%, 4.1%) | 0 (0.0%) | (0.0%, 8.2%) | 1.000 |
| Blood cholesterol increased | 1 (0.7%) | (0.1%, 4.1%) | 0 (0.0%) | (0.0%, 8.2%) | 1.000 |
| Blood potassium decreased | 1 (0.7%) | (0.1%, 4.1%) | 0 (0.0%) | (0.0%, 8.2%) | 1.000 |
| Blood triglycerides increased | 1 (0.7%) | (0.1%, 4.1%) | 0 (0.0%) | (0.0%, 8.2%) | 1.000 |
| Cervical dysplasia | 0 (0.0%) | (0.0%, 2.8%) | 1 (2.3%) | (0.4%, 12.1%) | 0.243 |
| Cervicitis | 0 (0.0%) | (0.0%, 2.8%) | 1 (2.3%) | (0.4%, 12.1%) | 0.243 |
| Chikungunya virus infection | 1 (0.7%) | (0.1%, 4.1%) | 0 (0.0%) | (0.0%, 8.2%) | 1.000 |
| Cholelithiasis | 0 (0.0%) | (0.0%, 2.8%) | 1 (2.3%) | (0.4%, 12.1%) | 0.243 |
| Complication associated with device | 1 (0.7%) | (0.1%, 4.1%) | 0 (0.0%) | (0.0%, 8.2%) | 1.000 |
| Complication of device insertion | 1 (0.7%) | (0.1%, 4.1%) | 0 (0.0%) | (0.0%, 8.2%) | 1.000 |
| Device breakage | 1 (0.7%) | (0.1%, 4.1%) | 0 (0.0%) | (0.0%, 8.2%) | 1.000 |
| Diabetes mellitus | 1 (0.7%) | (0.1%, 4.1%) | 0 (0.0%) | (0.0%, 8.2%) | 1.000 |
| Dyspareunia | 1 (0.7%) | (0.1%, 4.1%) | 0 (0.0%) | (0.0%, 8.2%) | 1.000 |
| Ear pain | 1 (0.7%) | (0.1%, 4.1%) | 0 (0.0%) | (0.0%, 8.2%) | 1.000 |
| Electrocardiogram PR prolongation | 1 (0.7%) | (0.1%, 4.1%) | 0 (0.0%) | (0.0%, 8.2%) | 1.000 |
| External ear inflammation | 0 (0.0%) | (0.0%, 2.8%) | 1 (2.3%) | (0.4%, 12.1%) | 0.243 |
| Eye pruritus | 1 (0.7%) | (0.1%, 4.1%) | 0 (0.0%) | (0.0%, 8.2%) | 1.000 |
| Femoroacetabular impingement | 1 (0.7%) | (0.1%, 4.1%) | 0 (0.0%) | (0.0%, 8.2%) | 1.000 |
| Flatulence | 1 (0.7%) | (0.1%, 4.1%) | 0 (0.0%) | (0.0%, 8.2%) | 1.000 |
| Fungal infection | 1 (0.7%) | (0.1%, 4.1%) | 0 (0.0%) | (0.0%, 8.2%) | 1.000 |
| Furuncle | 0 (0.0%) | (0.0%, 2.8%) | 1 (2.3%) | (0.4%, 12.1%) | 0.243 |
| Groin abscess | 1 (0.7%) | (0.1%, 4.1%) | 0 (0.0%) | (0.0%, 8.2%) | 1.000 |
| Herpes zoster | 0 (0.0%) | (0.0%, 2.8%) | 1 (2.3%) | (0.4%, 12.1%) | 0.243 |
| Hypersensitivity | 1 (0.7%) | (0.1%, 4.1%) | 0 (0.0%) | (0.0%, 8.2%) | 1.000 |
| Hypoaesthesia | 1 (0.7%) | (0.1%, 4.1%) | 0 (0.0%) | (0.0%, 8.2%) | 1.000 |
| Inflammation | 1 (0.7%) | (0.1%, 4.1%) | 0 (0.0%) | (0.0%, 8.2%) | 1.000 |
| Intracranial pressure increased | 1 (0.7%) | (0.1%, 4.1%) | 0 (0.0%) | (0.0%, 8.2%) | 1.000 |
| Iron deficiency | 1 (0.7%) | (0.1%, 4.1%) | 0 (0.0%) | (0.0%, 8.2%) | 1.000 |
| Laceration | 1 (0.7%) | (0.1%, 4.1%) | 0 (0.0%) | (0.0%, 8.2%) | 1.000 |
| Laryngitis bacterial | 1 (0.7%) | (0.1%, 4.1%) | 0 (0.0%) | (0.0%, 8.2%) | 1.000 |
| Malaria | 0 (0.0%) | (0.0%, 2.8%) | 1 (2.3%) | (0.4%, 12.1%) | 0.243 |
| Mastoiditis | 1 (0.7%) | (0.1%, 4.1%) | 0 (0.0%) | (0.0%, 8.2%) | 1.000 |
| Neutrophil count decreased | 1 (0.7%) | (0.1%, 4.1%) | 0 (0.0%) | (0.0%, 8.2%) | 1.000 |
| Night blindness | 1 (0.7%) | (0.1%, 4.1%) | 0 (0.0%) | (0.0%, 8.2%) | 1.000 |
| Oropharyngeal gonococcal infection | 1 (0.7%) | (0.1%, 4.1%) | 0 (0.0%) | (0.0%, 8.2%) | 1.000 |
| Otitis media bacterial | 0 (0.0%) | (0.0%, 2.8%) | 1 (2.3%) | (0.4%, 12.1%) | 0.243 |
| Ovarian cyst | 1 (0.7%) | (0.1%, 4.1%) | 0 (0.0%) | (0.0%, 8.2%) | 1.000 |
| Paronychia | 0 (0.0%) | (0.0%, 2.8%) | 1 (2.3%) | (0.4%, 12.1%) | 0.243 |
| Periorbital cellulitis | 1 (0.7%) | (0.1%, 4.1%) | 0 (0.0%) | (0.0%, 8.2%) | 1.000 |
| Plantar fasciitis | 1 (0.7%) | (0.1%, 4.1%) | 0 (0.0%) | (0.0%, 8.2%) | 1.000 |
| Platelet count decreased | 1 (0.7%) | (0.1%, 4.1%) | 0 (0.0%) | (0.0%, 8.2%) | 1.000 |
| Pneumonia bacterial | 0 (0.0%) | (0.0%, 2.8%) | 1 (2.3%) | (0.4%, 12.1%) | 0.243 |
| Post lumbar puncture syndrome | 1 (0.7%) | (0.1%, 4.1%) | 0 (0.0%) | (0.0%, 8.2%) | 1.000 |
| Puncture site pain | 1 (0.7%) | (0.1%, 4.1%) | 0 (0.0%) | (0.0%, 8.2%) | 1.000 |
| Schistosomiasis | 1 (0.7%) | (0.1%, 4.1%) | 0 (0.0%) | (0.0%, 8.2%) | 1.000 |
| Seizure | 1 (0.7%) | (0.1%, 4.1%) | 0 (0.0%) | (0.0%, 8.2%) | 1.000 |
| Sensory loss | 1 (0.7%) | (0.1%, 4.1%) | 0 (0.0%) | (0.0%, 8.2%) | 1.000 |
| Somnolence | 0 (0.0%) | (0.0%, 2.8%) | 1 (2.3%) | (0.4%, 12.1%) | 0.243 |
| Subcutaneous abscess | 1 (0.7%) | (0.1%, 4.1%) | 0 (0.0%) | (0.0%, 8.2%) | 1.000 |
| Tooth abscess | 1 (0.7%) | (0.1%, 4.1%) | 0 (0.0%) | (0.0%, 8.2%) | 1.000 |
| Toothache | 1 (0.7%) | (0.1%, 4.1%) | 0 (0.0%) | (0.0%, 8.2%) | 1.000 |
| Toxicity to various agents | 1 (0.7%) | (0.1%, 4.1%) | 0 (0.0%) | (0.0%, 8.2%) | 1.000 |
| Umbilical hernia | 1 (0.7%) | (0.1%, 4.1%) | 0 (0.0%) | (0.0%, 8.2%) | 1.000 |
| Urethritis gonococcal | 1 (0.7%) | (0.1%, 4.1%) | 0 (0.0%) | (0.0%, 8.2%) | 1.000 |
| Uterine haemorrhage | 0 (0.0%) | (0.0%, 2.8%) | 1 (2.3%) | (0.4%, 12.1%) | 0.243 |
| Vitamin D deficiency | 1 (0.7%) | (0.1%, 4.1%) | 0 (0.0%) | (0.0%, 8.2%) | 1.000 |
| Xerosis | 1 (0.7%) | (0.1%, 4.1%) | 0 (0.0%) | (0.0%, 8.2%) | 1.000 |
| Zika virus infection | 1 (0.7%) | (0.1%, 4.1%) | 0 (0.0%) | (0.0%, 8.2%) | 1.000 |
|  | | | | | |
