# Supplementary material for: Safety, tolerability, and pharmacokinetics of long-acting injectable cabotegravir in low-risk HIV-uninfected individuals: HPTN 077, a phase 2a randomized controlled trial
Source: PLoS Med. 2018 Nov 8;15(11):e1002690. doi: 10.1371/journal.pmed.1002690 (PMC6224042; doi:10.1371/journal.pmed.1002690)
Supplement: S2 Table — (DOCX) [file pmed.1002690.s006.docx]

S2 Table. Study product discontinuation by cohort and arm (oral and injection phase)

| **Product discontinuation reason** | **CAB** | | | | **PBO** | | | |
| --- | --- | --- | --- | --- | --- | --- | --- | --- |
|  | **COHORT 1** | | **COHORT 2** | | **COHORT 1** | | **COHORT 2** | |
|  | **Males**  **N=28** | **Females**  **N=54** | **Males**  **N=23** | **Females**  **N=46** | **Males**  **N=10** | **Females**  **N=18** | **Males**  **N=6** | **Females**  **N=14** |
| Withdrew consent | 0 (0%) | 0 (0%) | 1 (4%) | 0 (0%) | 0 (0%) | 0 (0%) | 0 (0%) | 0 (0%) |
| Unsupportive social milieu | 0 (0%) | 2 (4%) | 0 (0%) | 0 (0%) | 0 (0%) | 0 (0%) | 0 (0%) | 0 (0%) |
| Study product related clinical AE | 2 (7%) | 5 (9%) | 0 (0%) | 3 (7%) | 0 (0%) | 0 (0%) | 0 (0%) | 0 (0%) |
| Protocol specified lab abnormality | 2 (7%) | 2 (4%) | 1 (4%) | 0 (0%) | 1 (10%) | 1 (6%) | 0 (0%) | 0 (0%) |
| Pregnancy or expresses desire to become pregnant | 0 (0%) | 1 (2%) | 0 (0%) | 0 (0%) | 0 (0%) | 1 (6%) | 0 (0%) | 1 (7%) |
| Pre-existing seizure | 0 (0%) | 0 (0%) | 0 (0%) | 0 (0%) | 0 (0%) | 2 (11%) | 0 (0%) | 0 (0%) |
| Poor adherence | 0 (0%) | 1 (2%) | 1 (4%) | 2 (4%) | 0 (0%) | 0 (0%) | 0 (0%) | 0 (0%) |
| Participant unwilling or unable to comply with procedures | 1 (4%) | 4 (7%) | 1 (4%) | 1 (2%) | 1 (10%) | 2 (11%) | 0 (0%) | 2 (14%) |
| Participant choice | 0 (0%) | 1 (2%) | 0 (0%) | 1 (2%) | 0 (0%) | 0 (0%) | 1 (17%) | 0 (0%) |
| Injection site reaction | 0 (0%) | 1 (2%) | 0 (0%) | 0 (0%) | 0 (0%) | 0 (0%) | 0 (0%) | 0 (0%) |
| False positive HIV testing | 0 (0%) | 1 (2%) | 0 (0%) | 1 (2%) | 0 (0%) | 0 (0%) | 0 (0%) | 0 (0%) |
| Change in risk profile | 0 (0%) | 0 (0%) | 1 (4%) | 1 (2%) | 0 (0%) | 0 (0%) | 0 (0%) | 0 (0%) |

AE = adverse event; CAB = cabotegravir; PBO = placebo
