# Supplementary material for: Safety, tolerability, and pharmacokinetics of long-acting injectable cabotegravir in low-risk HIV-uninfected individuals: HPTN 077, a phase 2a randomized controlled trial
Source: PLoS Med. 2018 Nov 8;15(11):e1002690. doi: 10.1371/journal.pmed.1002690 (PMC6224042; doi:10.1371/journal.pmed.1002690)
Supplement: S1 Text — (PDF) [file pmed.1002690.s007.pdf]

HPTN 077

A Phase IIa Study to Evaluate the Safety, Tolerability and Pharmacokinetics of the Investigational Injectable HIV Integrase Inhibitor, GSK1265744, in HIV-uninfected Men and Women

DAIDS Document ID: 11964

A Study by the HIV Prevention Trials Network

Sponsored by:
Division of AIDS, US National Institute of Allergy and Infectious Diseases

Pharmaceutical Support Provided by:
ViiV Healthcare

IND # 122,744

Protocol Chair:
Raphael J. Landovitz, M.D., M.Sc.

Final Version 3.0
October 13, 2015

HPTN 077
A Phase IIa Study to Evaluate the Safety, Tolerability, and Pharmacokinetics of the Investigational Injectable HIV Integrase Inhibitor, GSK1265744, in HIV-uninfected Men and Women

TABLE OF CONTENTS

LIST OF ABBREVIATIONS AND ACRONYMS 5

PROTOCOL TEAM ROSTER 8

TERMINOLOGY FOR GSK1265744 FORMULATIONS 11

SCHEMA 12

OVERVIEW OF STUDY DESIGN AND RANDOMIZATION SCHEME 15

1.0 INTRODUCTION 16

1.1 Background and Rationale 16
1.2 Overview of GSK1265744 oral (oral 744) and long acting injectable (744LA) 17
1.3 Dose Rationale 20
1.4 Clinical Experience to Date – GSK1265744 26
1.5 Risk Assessment for GSK1265744 30
1.6 Acceptability 31
1.7 Sexual Risk Behavior 31
1.8 Rationale for Placebo-Controlled Study Design 31

2.0 STUDY OBJECTIVES AND DESIGN 33

2.1 Primary Objective 33
2.2 Secondary Objectives 33
2.3 Exploratory Objectives 33
2.4 Study Design and Overview 34

3.0 STUDY POPULATION 35

3.1 Inclusion Criteria 35
3.2 Exclusion Criteria 36
3.3 Recruitment Process 38
3.4 Co-Enrollment Guidelines 38
3.5 Participant Retention 38
3.6 Participant Withdrawal 38

4.0 STUDY PRODUCT CONSIDERATIONS 40

4.1 Study Product Regimens/Administration/Formulation Content 40
4.2 Study Product Acquisition and Accountability 41
4.3 Toxicity Management 41
4.4 Concomitant, Prohibited, and Precautionary Medications 41

5.0 STUDY PROCEDURES 42

5.1 Screening: Both Cohorts 42
5.2 Week 0: Enrollment – Both Cohorts 43
5.3 Weeks 2 and 4: Oral Safety Visits (Week 4 is Post-Oral Safety Visit) – Both Cohorts 44
5.4 First Injection Visit: Both Cohorts - Week 5 45
5.5 Safety Visits: Cohort 1 - Week 6, 9, 13; Cohort 2 - Week 6 only 45
5.6 Second Injection Visit: Cohort 1 – Week 17; Cohort 2 – Week 9 46

5.7 Safety Visits: Cohort 1 - Week 18 and 23; Cohort 2 – Week 10 and 13 46
5.8 Third Injection Visit: Cohort 1 – Week 29; Cohort 2 – Week 17 47
5.9 Safety Visits: Cohort 1 – Week 30 and 35; Cohort 2 – Week 18 and 21 48
5.10 Fourth Injection Visit: Cohort 2 only - Week 25 48
5.11 Safety Visits: Cohort 2 only – Week 26 and 29 49
5.12 Fifth Injection Visit: Cohort 2 only – Week 33 49
5.13 Safety Visits: Cohort 2 only – Weeks 34 and 37 50
5.14 Primary Endpoint Visit: Both Cohorts - Week 41 50
5.15 Tail Phase Visits: Both Cohorts – Week 53, 65, 77 51
5.16 Final Visit: Cohort 1 – Week 81; COHORT 2 – Week 85 51
5.17 Injection Visit Windows 52
5.18 Procedures for Continued Oral and Injectable Dosing 52
5.19 Procedures for Participants Who Do Not Complete the Full Course of Injections 52
5.20 Planned Unblinding of Study Participants 52
5.21 Participants with Suspected or Confirmed HIV Infection, at Screening, Enrollment, or Follow-Up 52
5.22 Sexually Transmitted Infections 54
5.23 Pregnancy 54
5.24 Changes in Reported HIV/STI Risk Status During Follow-Up 55
5.25 Hepatitis B and Hepatitis C 55
5.26 Interim Contacts and Visits 55
5.27 Criteria for Early Termination of Study Participation 55
5.28 Pharmacokinetics 56

6.0 SAFETY MONITORING AND ADVERSE EVENT (AE) REPORTING 57

6.1 Safety Monitoring 57
6.2 Clinical Data Review 57
6.3 Adverse Event Definition and Reporting 57
6.4 Expedited Adverse Event Reporting 58
6.5 Social Impact Reporting 59

7.0 STATISTICAL CONSIDERATIONS 60

7.1 Review of Study Design 60
7.2 Sample Size 61
7.3 Accrual, Follow-up, and Retention 65
7.4 Random Assignment 65
7.5 Blinding 65
7.6 Data and Safety Monitoring Analysis 66

8.0 HUMAN SUBJECTS CONSIDERATIONS 70

8.1 Ethical Review 70
8.2 Informed Consent 70
8.3 Incentives 70
8.4 Confidentiality 70
8.5 Communicable Disease Reporting Requirements 71
8.6 Study Discontinuation 71

9.0 LABORATORY SPECIMENS AND BIOHAZARD CONTAINMENT 72

9.1 Local Laboratory Specimens 72
9.2 Stored Specimens 73
9.3 Quality Control and Quality Assurance Procedures 74
9.4 Specimen Storage and Possible Future Research Testing 75
9.5 Biohazard Containment 75

10.0 ADMINISTRATIVE PROCEDURES....76

10.1 PROTOCOL REGISTRATION 76
10.2 STUDY ACTIVATION....76
10.3 STUDY COORDINATION 76
10.4 STUDY MONITORING....77
10.5 PROTOCOL COMPLIANCE....77
10.6 INVESTIGATOR'S RECORDS....77
10.7 USE OF INFORMATION AND PUBLICATIONS 77

11.0 REFERENCES....78

APPENDICES I-XIII 82

APPENDIX I: SCHEDULE OF PROCEDURES AND EVALUATIONS – FOR PARTICIPANTS WHO COMPLETE ALL THREE INJECTIONS IN COHORT 1....83

APPENDIX II: SCHEDULE OF PROCEDURES AND EVALUATIONS – FOR PARTICIPANTS WHO COMPLETE TWO INJECTIONS ONLY IN COHORT 1....86

APPENDIX III: SCHEDULE OF PROCEDURES AND EVALUATIONS – FOR PARTICIPANTS WHO COMPLETE ONE INJECTION ONLY IN COHORT 1 88

APPENDIX IV: SCHEDULE OF PROCEDURES AND EVALUATIONS – FOR PARTICIPANTS WHO COMPLETE ALL FIVE INJECTIONS IN COHORT 2 90

APPENDIX V: SCHEDULE OF PROCEDURES AND EVALUATIONS – FOR PARTICIPANTS WHO COMPLETE FOUR INJECTIONS ONLY IN COHORT 2 93

APPENDIX VI: SCHEDULE OF PROCEDURES AND EVALUATIONS – FOR PARTICIPANTS WHO COMPLETE THREE INJECTIONS ONLY IN COHORT 2....95

APPENDIX VII: SCHEDULE OF PROCEDURES AND EVALUATIONS – FOR PARTICIPANTS WHO COMPLETE TWO INJECTIONS ONLY IN COHORT 2....97

APPENDIX VIII: SCHEDULE OF PROCEDURES AND EVALUATIONS – FOR PARTICIPANTS WHO COMPLETE ONE INJECTION ONLY IN COHORT 2 99

APPENDIX IX: SCHEDULE FOR ADDITIONAL LABORATORY PROCEDURES FOR ENROLLED PARTICIPANTS WHO HAVE A REACTIVE POSITIVE HIV TEST RESULT….. 101

APPENDIX X: RISK ASSESSMENT AND MITIGATION STRATEGY....102

APPENDIX XI: TOXICITY MANAGEMENT....105

APPENDIX XII: SAMPLE SCREENING AND ENROLLMENT INFORMED CONSENT FORM FOR PARTICIPANTS ENROLLING UNDER VERSION 3.0....114

APPENDIX XIII: SAMPLE HPTN 077 PARTICIPANT LETTER/INFORMATION SHEET FOR PARTICIPANTS ENROLLED UNDER VERSION 2.0 133

LIST OF ABBREVIATIONS AND ACRONYMS

3TC	lamivudine
Oral 744	oral formulation of GSK1265744
744LA	long-acting injectable formulation of GSK1265744
ABC/3TC	abacavir/lamivudine
AE	adverse event
AIDS	acquired immunodeficiency syndrome
ALT	alanine aminotransferase
ART	antiretroviral therapy
ARV	antiretroviral
AST	aspartate aminotransferase
AUC	area under the curve
AUDIT	Alcohol Use Disorders Identification Test
AV	atrioventricular
BCRP	breast cancer resistance protein
CAB	Community Advisory Board
CABG	coronary artery bypass grafting
CBC	complete blood count
CDC	(US) Centers for Disease Control and Prevention
CFR	(US) Code of Federal Regulations
CI	confidence interval
CLIA	Clinical Laboratory Improvement Amendments
CMC	Clinical Management Committee
C_{max}	maximum or “peak” concentration of a drug observed after its administration
C_{min}	minimum or “trough” concentration of a drug observed after its administration and just prior to the administration of a subsequent dose
CPK	creatine phosphokinase
CPQA	Clinical Pharmacology Quality Assurance Committee
CRF	Case Report Form
CROI	Conference on Retroviruses and Opportunistic Infections
CRPMC	(NIAID) Clinical Research Products Management Center
DAERS	DAIDS Adverse Event Reporting System
DAIDS	Division of AIDS
DAIDS ES	DAIDS Enterprise System
DAIDS PRO	DAIDS Protocol Registration Office
DDI	drug-drug interaction
DNA	deoxyribonucleic acid
EAE	expedited adverse event
EC	Ethics Committee
ECG	electrocardiogram
EQA	external quality assurance

ETR	etravirine
FDA	(US) Food and Drug Administration
FEM-PrEP	Phase 3, double-blind, randomized, placebo-controlled effectiveness and safety study among 3900 women at high risk of HIV infection at 6 sites in 4 African countries
FSH	follicle-stimulating hormone
GI	gastrointestinal
GSK	GlaxoSmithKline
HBV	hepatitis B virus
HCV	hepatitis C virus
HDPE	high density polyethylene
HIV	human immunodeficiency virus
HIV-1 IIIB	human immunodeficiency virus type 1 IIIB strain
HIV-1 NL-432	human immunodeficiency virus type 1 NL-432 strain
LC	HIV Prevention Trials Network (HPTN) Laboratory Center
LDMS	Laboratory Data Management System
LOC	(HPTN) Leadership and Operations Center
MOP	(HPTN) Manual of Operations
SDMC	(HPTN) Statistical and Data Management Center
HSR	hypersensitivity reaction
IATA	International Air Transport Association
ICAAC	Interscience Conference on Antimicrobial Agents and Chemotherapy
ID	identification
IM	intramuscular
IND	investigational new drug
iPrEx	Phase 3, safety and effectiveness trial of once-daily TDF/FTC in gay men, transgender women and other men who have sex with men in six countries
IQA	(DAIDS) Immunology Quality Assurance
IRB	Institutional Review Board
ISR	injection site reaction
IUD	intrauterine device
IUS	intrauterine system
LA	long-acting (injectable)
LAP	long-acting parenteral
LC	Laboratory Center
MT-2	lymphocyte cells
NIAID	National Institute of Allergy and Infectious Diseases
NIH	National Institutes of Health
NOAEL	no observed adverse effect level
NRTI	nucleoside/nucleotide reverse transcriptase inhibitors
OHRP	Office for Human Research Protections
PA-IC_{50}	protein-adjusted 50% inhibitory concentration
PA-IC_{90}	protein-adjusted 90% inhibitory concentration
PBMC	peripheral blood mononuclear cells
PCTA	percutaneous transluminal coronary angioplasty

PEG	polyethylene glycol 3350
PEP	post-exposure prophylaxis
Pgp	permeability glycoprotein
PK	pharmacokinetic
PO	by mouth/orally
PrEP	pre-exposure prophylaxis
QA	quality assurance
QC	quality control
qd	once daily dose
QRS	interval from the beginning of the Q wave to the end of the S wave
QTc	corrected QT
RE	relative effect; Regulatory Entity
RNA	ribonucleic acid
RPV	rilpivirine
RSC	(DAIDS) Regulatory Support Center
SAE	serious adverse event
SC	subcutaneous
SCHARP-FHCRC	Statistical Center for HIV/AIDS Research and Prevention – Fred Hutchinson Cancer Research Center
SD	standard deviation
SHIV	simian human immunodeficiency virus
SMC	Study Monitoring Committee
SSA	sub-Saharan Africa
SSP	Study Specific Procedures Manual
TDF	tenofovir disoproxil fumarate
TDF/FTC	tenofovir disoproxil fumarate/emtricitabine
TDF	tenofovir
ULN	upper limit of normal
UKNEQAS	United Kingdom National External Quality Assessment Service
US	United States of America
VQA	(DAIDS) Virology Quality Assurance
WHO	World Health Organization
WPW	Wolf Parkinson White syndrome

HPTN 077

A Phase IIa Study to Evaluate the Safety, Tolerability, and Pharmacokinetics of the Investigational Injectable HIV Integrase Inhibitor, GSK1265744, in HIV-uninfected Men and Women

PROTOCOL TEAM ROSTER

Namandje Bumpus, PhD
HPTN Laboratory Center Pharmacologist
Department of Pharmacology and Molecular Sciences
Johns Hopkins University School of Medicine
725 N. Wolfe Street, Biophysics 307A
Baltimore, MD 21205
Phone: 410-955-0562
Fax: 410-955-3023
Email: nbumpus1@jhmi.edu

Mike Cohen, MD
HPTN Co-PI
University of North Carolina School of Medicine
Department of Medicine
Division of Infectious Diseases
130 Mason Farm Road
Bioinformatics Bldg.
Chapel Hill, NC 27599
Phone: 919-966-2536
Fax: 919-966-6714
Email: myron_cohen@med.unc.edu

Cheryl Cokley, BS
HPTN Leadership and Operations Center Community Program Manager
FHI 360
359 Blackwell Street, Suite 200
Durham, NC 27701
Phone: 919-544-7040, ext. 11515
Fax: 919-544-0207
Email: ccokley@fhi360.org

Leslie Cottle, BA
HPTN SDMC Protocol Manager
SCHARP-FHCRC
1100 Fairview Avenue N, LE-400
PO Box 19024
Seattle, WA 98109
Phone: 206-667-7405
Fax: 206-667-4812
Email: leslie@scharp.org

Vanessa Elharrar, MD, MPH
DAIDS Medical Officer
Prevention Science Program
DAIDS/NIAID/NIH
5601 Fishers Lane, Rm 8B39, MSC 9830
Bethesda, MD 20892
Phone: 240-292-4787
Email: elharrarva@niaid.nih.gov

Joseph Eron, MD
Site Investigator
Professor of Medicine
University of North Carolina at Chapel Hill
Division of Infectious Diseases
130 Mason Farm Rd; CB 7215
Chapel Hill, NC 27599-7215
Phone: 919-843-2722
Fax: 919-966-6714
Email: jeron@med.unc.edu

Susan Eshleman, MD, PhD
HPTN Laboratory Center Virologist
Johns Hopkins Univ. School of Medicine
720 Rutland Avenue
Ross Bldg., Room 646
Baltimore, MD 21205
Phone: 410-614-4734
Fax: 410-502-9244
Email: seshlem@jhmi.edu

Beatriz Grinsztejn, MD, PhD
Site Investigator
Instituto de Pesquisa Clinica Evandro Chagas-Fiocruz
Avenida Brasil 4365
Rio de Janeiro, Brasil
Phone: 55-21-2270-7064
Fax: 55-21-25644933
Mobile: 55-21-981584631
Email: gbeatriz@ipec.fiocruz.br

Mina Hosseinipour, MD, MPH
Site Investigator
Clinical Director
UNC Project
Tidziwe Center
Kamuzu Central Hospital
100 Mzimba Road
Private Bag A/104
Lilongwe, Malawi
Phone: 265-1-755-056
Fax: 265-1-755-954
Email: minach@med.unc.edu

Quarraisha Abdool Karim, PhD
Site Investigator
CAPRISA
2nd Floor Doris Duke Medical Research Institute
Nelson R Mandela School of Medicine
University of KwaZuu-Natal
Private Bag 7
Congella 4013
South Africa
Phone: 27-31-2604550
Fax: 27-31-2604566
Email: abdoolq2@ukzn.ac.za

Raphael J. Landovitz, MD, MSc
Protocol Chair
UCLA Center for Clinical AIDS Research & Education
9911 W. Pico Blvd, Suite 980
Los Angeles, CA 90035
Phone: 310-557-1894
Fax: 310-557-1899
Email: rlandovitz@mednet.ucla.edu

Sue Li, PhD
HPTN SDMC Protocol Statistician
Fred Hutchinson Cancer Research Center
1100 Fairview Avenue N, M2-C200
PO Box 19024
Seattle, WA 98109
Phone: 206-667-7066
Fax: 206-667-6352
Email: sli@scharp.org

Albert Liu, MD, MPH
Site Investigator
Director, HIV Prevention Intervention Studies
Bridge HIV, San Francisco Department of Public Health
25 Van Ness Avenue, Suite 100
San Francisco, CA 94102
Phone: 415-437-7408
Fax: 415-431-7029
Email: albert.liu@sfdph.org

Manya Magnus, PhD, MPH
Site Investigator
George Washington University
School of Public Health and Health Services
Department of Epidemiology and Biostatistics
950 New Hampshire Avenue
Washington, DC 20052
Phone: 202-994-3024
Fax: 202-994 0082
Email: manyadm@gwu.edu

David Margolis, MD, MPH
GlaxoSmithKline
5 Moore Drive
Durham NC, 27709
Phone: 919-483-7869
Fax: 919-315-6359
Email: david.a.margolis@gsk.com

Marybeth McCauley, MPH
HPTN Leadership and Operations Center
Senior Clinical Program Manager
FHI 360
1825 Connecticut Avenue, NW
Washington, DC 20009
Phone: 202-884-8340
Fax: 202-884-8400
Email: mmccauley@fhi360.org

Ravindre Panchia, MBBCh
Site Investigator
Soweto HPTN CRS
Chris Hani Baragwanath Hospital
Soweto, South Africa
Phone: 27-11-9899711
Fax: 27-11-9899877
Email: panchiar@phru.co.za

Paul Richardson, MSc
HPTN Laboratory Center QA/QC Coordinator
Johns Hopkins Univ. Dept. of Pathology
Pathology Building 306
600 N. Wolfe St.
Baltimore, MD 21287
Phone: 410-502-0435
Fax: 410-614-0430
Email: pricha18@jhmi.edu

Alex Rinehart, PhD
ViiV Healthcare
5 Moore Drive
Durham, NC 27709
Phone: 919-483-2085 (office)
Phone: 919-599-7551 (mobile)
Email: alex.r.rinehart@viivhealthcare.com

William Spreen, PhD
GlaxoSmithKline
5 Moore Drive
Phone: 919-483-1393
Fax: 919-315-5263
Email: william.r.spreen@gsk.com
Durham, NC 27709

Britt Stancil, BS
GlaxoSmithKline
5 Moore Drive
Durham, NC 27709
Phone: 919-483-5561
Fax: 919-483-0272
Email: Britt.n.shahan@gsk.com

Jeremy Sugarman, MD, MPH
HPTN Ethics Working Group Representative
Harvey M. Meyerhoff Professor of Bioethics and Medicine
Johns Hopkins Berman Institute of Bioethics
1809 Ashland Ave.
Deering Hall, Room 203
Baltimore, MD 21205
Phone: 410-614-5634
Fax: 410-614-5360
Email: jsugarman@jhu.edu

Betsy Tolley, MA, PhD
FHI 360
359 Blackwell Street, Suite 200
Durham, NC 27701, USA
Phone: 919-544-7040, Ext. 11334
Fax: 919-405-1402
Email: btolley@fhi360.org

Phaedrea Watkins, MBA
HPTN Leadership and Operations Center
Clinical Research Manager
FHI 360
359 Blackwell St, Suite 200
Durham, NC 27701
Phone: 919-544-7040, ext. 11596
Fax: 919-405-1402
Email: pwatkins@fhi360.org

Michelle Wildman, Pharm D
Protocol Pharmacist
Division of AIDS/NIAID/NIH
5601 Fishers Lane, Room 9E15
Bethesda, MD 20892
Phone: 240-627-3058
Email: michelle.wildman@nih.gov

HPTN 077
A Phase IIa Study to Evaluate the Safety, Tolerability and Pharmacokinetics of the Investigational Injectable HIV Integrase Inhibitor, GSK1265744, in HIV-uninfected Men and Women

TERMINOLOGY FOR GSK1265744 FORMULATIONS

Compound Name or Abbreviation	Comments
**GSK1265744**	When written as shown, this is the ViiV Healthcare compound under study, and refers to the oral or long-acting (LA) formulation. Also when written as shown, it refers to plasma concentrations following both oral and LA formulations.
**Oral 744**	When written as shown, this refers to the oral formulation of GSK1265744.
**744LA**	When written as shown, this refers to the long-acting injectable formulation of GSK1265744.

HPTN 077

A Phase IIa Study to Evaluate the Safety, Tolerability and Pharmacokinetics of the Investigational Injectable HIV Integrase Inhibitor, GSK1265744, in HIV-uninfected Men and Women

SCHEMA

Purpose: To evaluate the safety, tolerability, pharmacokinetics and acceptability of the injectable agent, GSK1265744 long-acting injectable (744LA), in healthy, HIV-uninfected men and women.

Design: Multi-site, double-blind, two-arm, randomized, placebo-controlled trial of the safety, tolerability, and acceptability of 744LA.

Population: HIV-uninfected men and women at low to minimal risk for acquiring HIV infection, ages 18 to 65.

Study Size: Approximately 194 men and women, randomized 3:1, with approximately 145 in the active drug arm, and approximately 49 in the placebo arm. It is anticipated that approximately 60% of the enrolled participants will be women. Two cohorts will be enrolled (Cohort 1 and Cohort 2). Cohort 1 will include approximately 106 participants (approximately 40 men and 66 women), and Cohort 2 will include approximately 88 participants (approximately 35 men and 53 women).

Study Regimens: Participants will be randomized to receive daily oral GSK1265744 (30 mg tablets) or daily oral placebo for 4 weeks, followed by a one-week washout, to assess safety and tolerability before they receive injections. After safety and tolerability assessments from the oral phase have been completed (if no concerns are identified), participants will enter the injection phase of the study. Cohort 1 will receive two intra-muscular (IM) gluteal injections of 744LA (800 mg, administered as two 400 mg injections) or placebo (matching vehicle control) at three study visits performed at 12-week intervals (Week 5, 17, and 29). Cohort 2 will receive a single IM gluteal injection of 744LA (600 mg or placebo) at five study visits, with the first two occurring 4 weeks apart (Weeks 5 and 9), and the remaining three at 8-week intervals (Weeks 17, 25, and 33).

Study Duration: Approximately 3 years. Accrual will require approximately 16 to 24 weeks (4 – 6 months) for each cohort. Participants in both cohorts will receive oral tablets for 4 weeks, followed by a 1 week wash out period. Cohort 1 will then receive intra-muscular (IM) injections at 3 time points over 24 weeks (6 months); Cohort 2 will receive IM injections at 5 time points over 28 weeks (7 months). Participants in the active study product arm will be followed for 52 weeks (12 months) after their last injection. Participants in the placebo arm will be followed until 52 weeks after their last injection or until the last participant in the active study product arm of Cohort 2 completes their Week 41 visit, whichever comes first, after which the cohorts will be unblinded to assess the primary endpoint. When all procedures related to unblinding are completed, participants in the placebo arms of both cohorts will no longer be followed.

Study Sites: HIV Prevention Trials Network (HPTN)-affiliated Clinical Trial Units (CTUs) and/or Clinical Research Sites (CRSs) participating in this study will be listed in the SSP Manual, and will include sites in Brazil, sub-Saharan Africa (SSA), and the United States (US).

SCHEMA (Continued)

Primary Objectives:

- Evaluate the safety and tolerability of the injectable agent GSK1265744 long acting (744LA) injectable (800 mg dose administered at three time points at 12 week intervals), through Week 41 in HIV-uninfected men and women.
- Evaluate the safety and tolerability of the injectable agent GSK1265744 long acting (744LA) injectable (600 mg dose administered at two time points at 4 week intervals, followed by three time points at 8 week intervals), through Week 41 in HIV-uninfected men and women.

Secondary Objectives:

- Evaluate the safety and tolerability of GSK1265744 (daily oral 744 + 744LA) for 52 weeks of follow-up after final injection (each cohort will be analyzed separately)
- Evaluate the safety and tolerability of oral 744 from Week 0 to Week 5
- Evaluate the pharmacokinetics of 744LA administered at three time points as 800 mg IM every 12 weeks for 52 weeks of follow-up after final injection
- Evaluate the pharmacokinetics of 744LA administered at five time points as 600 mg IM at two time points every 4 weeks followed by three time points every 8 weeks for 52 weeks of follow-up after final injection
- Evaluate differential pharmacokinetics of 744LA in participants by age, gender, race, ethnicity, weight, body mass index (BMI), and smoking status
- Evaluate the acceptability of 744LA injections
- Evaluate the effect of 744LA on sexual risk behavior by change from enrollment over time during the study period
- Evaluate HIV incidence and antiretroviral drug resistance, in participants who acquire HIV infection during the study
- Evaluate the safety, tolerability and pharmacokinetics of 744LA in the subset of women who use a hormone-based contraceptive

Exploratory Objectives:

- Explore relationships between safety parameters and GSK1265744 concentrations and/or participant demographic factors
- Explore genetic factors that may impact the pharmacokinetics of 744LA

- Perform secondary laboratory assessments which may include evaluation of factors related to HIV infection, hepatitis infection, or sexually transmitted infections (STIs); antiretroviral (ARV) drug use; pharmacogenomics; characterization of HIV in infected participants; and evaluation of laboratory assays related to the study objectives

HPTN 077
A Phase IIa Study to Evaluate the Safety and Pharmacokinetics of the Investigational Injectable HIV Integrase Inhibitor, GSK1265744, in HIV-uninfected Men and Women

OVERVIEW OF STUDY DESIGN AND RANDOMIZATION SCHEME

COHORT 1

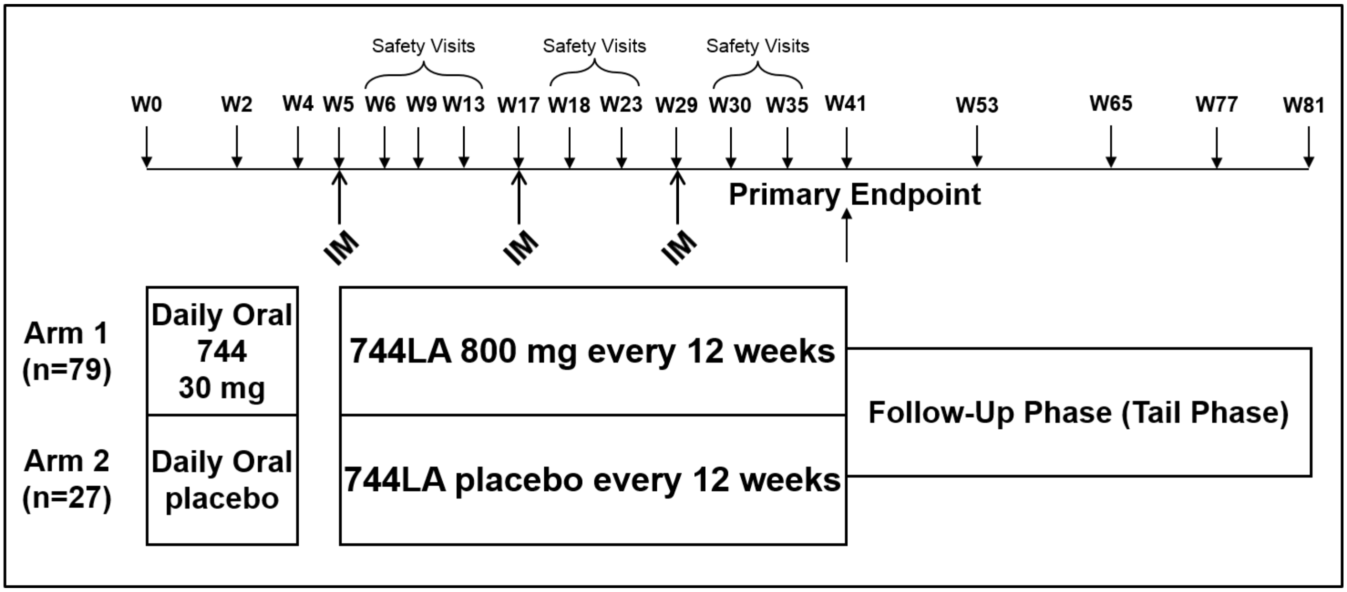

W = Week

COHORT 2

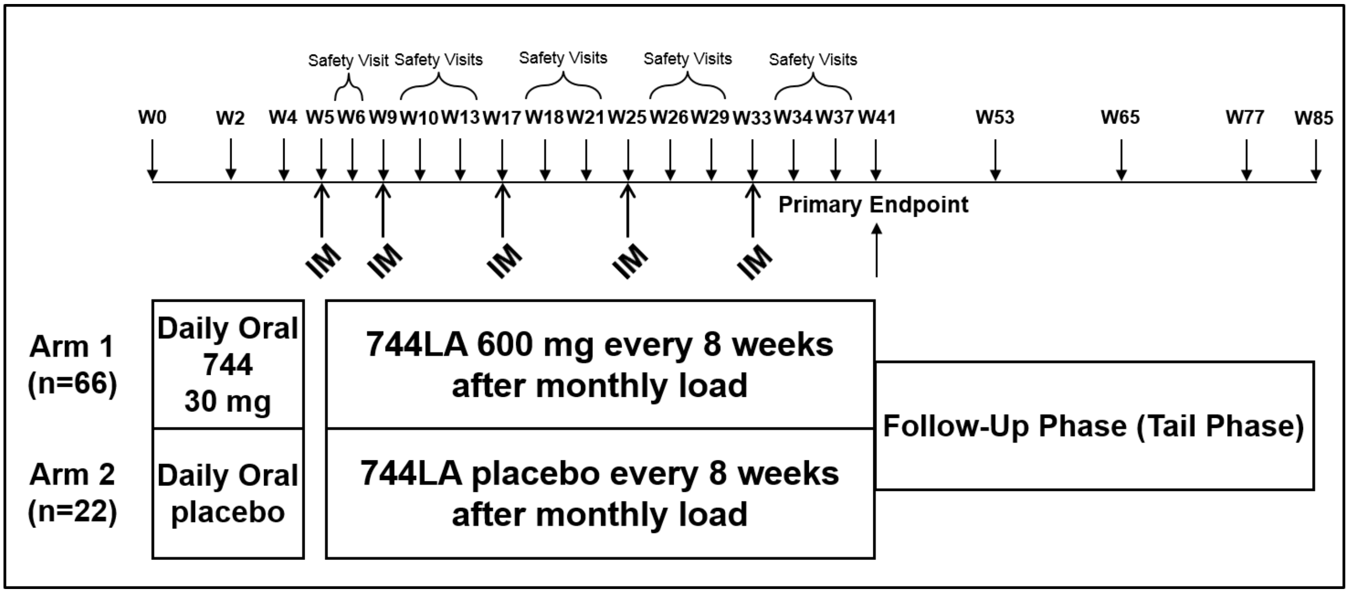

W = Week

1.0 INTRODUCTION

1.1 Background and Rationale

HPTN 077 is a Phase IIa study designed to establish the safety, tolerability, and multiple-dose pharmacokinetics of GSK1265744 in low-risk HIV-uninfected men and women. Following small single-dose and multiple-dose studies of a long-acting nanosuspension formulation (744LA), this study is the next developmental investigation of the 744LA nanosuspension in healthy, HIV negative, low-risk for HIV acquisition men and women. While preventive and therapeutic efficacy have yet to be definitively established, the goal of the product development pathway (by way of planned future Phase 3 studies) to establish the efficacy, safety, and tolerability of 744LA for HIV prevention. A parallel developmental pathway is being pursued for HIV therapeutic use.

The study of systemic antiretroviral therapy (ART) for both HIV treatment and prevention has provided consistent and compelling evidence of efficacy[1-8]. A challenge in the use of oral formulations for each indication is user dependence on adherence to the dosing strategy[9.10]. In healthy uninfected individuals and infected individuals with therapeutic success, sustaining adherence with time becomes increasingly challenging[11]. In addition to treatment failure, one of the concerning sequellae of treatment non-adherence is the development of resistant HIV quasispecies[12-15]. HIV seroconversion in the context of ART use for prevention has been a rare occurrence, with the overwhelming majority of seroconversions occurring in the context of subtherapeutic drug levels belying non-adherence. Resistance has therefore not been frequently seen in such prevention failures, absent selective drug pressure – and almost exclusively in cases in which the therapeutic agent was begun during occult primary HIV infection [2,3].

New ART drugs continue to be developed, and an exciting advance is the formulation of new drugs in long-acting slow release injectable formulations[16]. This has significant implications for patients who have achieved therapeutic success on oral medication and need to maintain this over many years or for HIV uninfected persons who are at risk of acquiring HIV.

Despite these advances in both treatment and prevention, the HIV epidemic persists worldwide[17]. Oral tenofovir disoproxil fumarate/emtricitabine (TDF/FTC) was recently approved by the United States (US) Food and Drug Administration (FDA) for PrEP and the US Centers for Disease Control and Prevention (CDC) and the World Health Organization (WHO) have offered interim guidance about its use[18,19]. The safety and efficacy of TDF/FTC in combination with other antiretroviral (ARV) drugs for antiretroviral therapy (ART)[20-24,] and results from TDF/FTC PrEP in a rhesus macaque rectal simian-HIV (SHIV) exposure model[25,26], supported the development and evaluation of TDF/FTC for PrEP. Six randomized clinical trials (RCTs) have evaluated oral TDF or TDF/FTC as PrEP[2,3,27-30]. These six trials differ in several ways, but suboptimal adherence[31,32] with a daily pill regimen was noted in the FEM-PrEP[28], iPrEx[2], and VOICE[30] trials. In fact, the FEM-PrEP and VOICE trials, both conducted in sexually active, young women in sub-Saharan Africa and requiring adherence to a daily regimen, were either stopped for futility or showed no efficacy on completion. Both, on further investigation, confirmed very poor adherence by trial participants to study product. Analyses of the available randomized controlled trial data suggests a strong correlation between adherence to study product and observed efficacy. While the TDF-based therapy provides an exciting new prevention tool, there are several reasons why alternative PrEP agents are needed, including: 1) TDF/FTC causes side effects that may impede adherence[2;] 2) TDF/FTC has been associated with increased creatinine levels and decreased bone density[33-35]; 3) Tenofovir (TDF) does not concentrate in the cervix or vagina after a single dose, and this may reduce "forgiveness" in the protection of women if doses are missed;[36] and 4) TDF/FTC is a key component of many first-line ART regimens, so resistance that might emerge with its use for PrEP is highly undesirable.[37]

PrEP may only reach its full potential with agents that do not depend on daily pill-taking. Therefore, development of alternative agents for PrEP, including long-acting injectable agents, is

desirable. Such agents have the potential to prevent HIV acquisition without relying on adherence to a daily oral regimen. Injectable agents are being used by women for contraception. In the future, an effective PrEP agent has the potential to be combined with an effective contraceptive for prevention of both pregnancy and HIV infection, and would be a valuable tool for HIV prevention in women in resource-limited settings, including sub-Saharan Africa, which bear the majority of prevalent and incident HIV/AIDS cases worldwide.

The evaluation of the GSK1265744 long-acting injectable integrase inhibitor (744LA) for PrEP during this relatively early stage of clinical development addresses an important issue. It is imperative that HIV-uninfected individuals who are offered PrEP receive a safe and well-tolerated agent; a significant number of persons who are at high risk of acquiring HIV infection are currently unable to effectively use oral agents or condoms to prevent transmission[30, 38]. Developing alternative options for PrEP remains a high priority. PrEP development has to date been restricted to agents that have already been shown to be well-tolerated and safe in subjects with HIV infection who benefit directly from the proven antiviral activity of these agents. The parallel development plan of 744LA for the treatment of HIV infection will provide expedited safety and tolerability data to support development of this drug as a PrEP agent. Further, the therapeutic utility of long-acting agents for treatment of HIV is dependent on availability and co-administration of the agent with other long-acting formulated agents to create a fully-suppressive long-acting ART combination to realize and exploit the potential of the improved pharmacokinetics (PK).

1.2 Overview of GSK1265744 oral (oral 744) and long acting injectable (744LA)

The majority of information contained in this section is a summary of information contained in the GSK1265744 Investigator's Brochure, Effective Date 01 December 2014, unless otherwise noted. [39]

GSK1265744 is an investigational HIV-1 integrase strand transfer inhibitor that possesses attributes favorable for both HIV treatment and prevention indications. Currently in Phase 2 clinical trials, it was initially selected for development based on its potential for a high genetic barrier to resistance and a pharmacokinetic (PK) profile that allows low-dose, once-daily oral dosing or monthly to quarterly parenteral dosing using a nanosuspension formulation. An oral tablet version of GSK1265744 (oral 744) is also under development as lead-in therapy to establish acute safety and tolerability in individual subjects prior to switching to the long-acting formulation. 744LA has a plasma half-life of 21 to 50 days in healthy HIV-uninfected adults, supporting a quarterly dosing interval when used for PrEP.

744LA has demonstrated activity in preventing SHIV infection in non-human primate models[40]. In a preclinical study evaluating the potential of 744LA for PrEP, eight male rhesus macaques were injected with 744LA (50 mg/kg intramuscularly [IM]) at two time points, 1 week prior to the first virus exposure and 4 weeks later. An additional eight male macaques were untreated and served as placebo controls. All animals were challenged intra-rectally each week with SHIV162p3 (50 tissue culture infective dose $[TCID]_{50}$) for up to eight exposures. All eight placebo macaques became infected after a median of two rectal exposures (range 1 to 7). Of the eight 744LA-treated macaques, none had detectable systemic viremia 10 weeks after the last virus challenge. In these protected animals, the plasma concentrations of 744LA throughout the period of virus challenges were comparable to clinically relevant concentrations in humans.

After acquisition of these proof-of-concept results, a follow-up study was performed to confirm the initial findings and determine plasma levels of GSK1265744 at which protection is no longer maintained during repeated low-dose intrarectal SHIV challenge in male macaques. Twelve male macaques were injected with 744LA 50 mg/kg IM one week prior to the first viral challenge. Four animals remained untreated as placebo controls, with one placebo animal beginning viral challenge every 4 weeks. All animals were challenged rectally each week with SHIV162p3 (50 $TCID_{50}$) until infection was established. Of the 12 male macaques treated with 744LA, none had

detectable systemic viremia following the first 4 weekly challenges confirming the previous observation that monthly administration of 744LA protects male macaques against repeated intrarectal low-dose SHIV challenge. Thereafter, 744LA-treated animals became infected after 6 to 17 challenges compared with 1 to 7 challenges for the 12 placebo controls (4 in-study and 8 historical from initial study). One dose of 744LA delayed infection by 5 to 10 (median 8) challenges compared with untreated controls. The percent of challenges resulting in infection were calculated relative to the plasma GSK1265744 protein-adjusted inhibitory concentration (PA-IC_{90}) value. None of 59 challenges resulted in infection when plasma levels were greater than 3 times the PA-IC_{90}, compared with 1 out of 22 challenges resulting in infection when plasma levels were between 1 to 3 times the PA-IC_{90} and 11 out of 43 challenges resulting in infection when plasma levels were less than 1 times the PA-IC_{90}. Twelve out of 26 viral challenges resulted in infection in control animals; rectal tissue levels of 744 were approximately 20% of plasma levels[41,42].

Intravaginal challenge studies with GSK1265744 in pigtail macaques demonstrated that 3 monthly injections of 50 mg/kg of 744LA, protected against infection by 12 cycles of weekly vaginal SHIV challenges. Protection was durably demonstrated out to 28 weeks; vaginal fluid 744 concentrations were 16-26% of plasma concentrations. Placebo injected animals became infected after identical vaginal challenges after an average of 2-4 challenges[43].

1.2.1 In Vitro Studies

GSK1265744, is a potent inhibitor of HIV integrase *in vitro*. It inhibits integrase-catalyzed viral deoxyribonucleic acid (DNA) strand transfer with half maximal inhibitory concentration (IC_{50}) values in the nanomolar range (3.0 to 13.0 nM). GSK1265744 is a potent antiviral agent when tested in a variety of *in vitro* assays. Additionally, the IC_{50} values of GSK1265744 for viral replication were also in the low nanomolar concentration range for National Institutes of Health (NIH) reference strains (24 HIV-1 strains and 3 HIV-2 strains in peripheral blood mononuclear cell (PBMC) assays; 3 HIV-1 strains in monocyte-derived macrophage assays). The protein adjusted IC_{50} (PA-IC_{50}) was estimated to be 102 nM in HIV-1 IIIB infected MT-4 cells. Exposure of MT-2 cells infected with HIV-1 IIIB to GSK1265744 for up to 112 days did not produce any highly resistant mutants. No amino acid substitutions in the integrase (IN) region were selected when passaging the wild type HIV-1 NL-432 in the presence of 6.4 nM GSK1265744 for 56 days.

1.2.2 Metabolism

The protein binding of GSK1265744 in rat, dog, monkey and human plasma and serum was high (>99%). GSK1265744 is a substrate for permeability glycoprotein (Pgp) but because of its high permeability, no alteration in absorption would be expected by co-administration of either Pgp or breast cancer resistance protein (BCRP) inhibitors. After oral administration of GSK1265744 to rats, radioactivity was slowly absorbed, widely distributed to tissues and minimally associated with blood cellular components. Elimination of radioactivity was slow with most tissues containing low but quantifiable radioactivity at 28 days. Following oral administration of GSK1265744 to mice and rats, GSK1265744 was the principal component in plasma of both species, representing up to 99.4% and 92.3% of the plasma radioactivity, respectively. Elimination of drug-related material occurred predominantly via the feces, with the majority of the absorbed radioactivity secreted into the bile. Renal excretion was minimal, with less than 1% of the dose eliminated in the urine. The primary route of biotransformation in mice and rats was conjugation with glucuronic acid (M1), similar to its metabolism in human hepatocytes. Overall, these findings suggest that metabolism represents a significant route of clearance for absorbed GSK1265744 in the mouse and rat.

1.2.3 Preclinical Studies

The GSK1265744 toxicology package supports the careful conduct of clinical studies with GSK1265744 up to the no observed adverse effect level (NOAEL) exposure in the 39-week monkey toxicity study (Week 39 gender mean area under the curve (AUC_{0-24}) and C_{max} of 547 μg·h/mL and 34.6 μg/mL, respectively. The results of the multiple dose rat subcutaneous (SC) and IM toxicity study, along with data from the oral toxicity program, support the careful conduct of clinical studies with GSK1265744 injectable suspension up to the mean C_{max} plasma concentration observed at the NOAEL in the 39-week monkey oral GSK1265744 toxicity study (34.6 μg/mL) or the mean $AUC_{1,440-2,160h}$ following once monthly IM dosing at the NOAEL (75 mg/kg/dose) in the 3-month rat 744LA toxicity study (92,566 μg·h/mL).

In a rat pre- and postnatal development (PPN) study, female pregnant rats were administered 0.5, 5 or 1000 mg/kg of oral 744 daily from gestation day 6 to postnatal day 21 (without dosing to the offspring directly). In the high-dose (1000 mg/kg/day) group, there was an increased number of rat offspring dead at birth (2.9% stillborn vs. 0.7% in control) and offspring that died during the early postnantal period (10.2% dead or missing on post-natal day 2-4 vs. 0.7% in control). This resulted in a decrease in rat offspring viability during post-natal day 1-4 (87.4% vs. 98.9%) and a corresponding decrease in live litter size on post-natal day 4 (10 offspring/litter vs. 11.5 in control on post-natal day 4). There were no treatment-related findings in the 0.5 or 5 mg/kg/day groups. Clinical dosing in HPTN 077 is approximately 7 fold below the NOAEL in 10-day-old rat offspring. Importantly, an early fetal development (EFD) study in rats was negative.

1.2.4 Pharmacokinetics

The PK of GSK1265744 in both healthy HIV-uninfected subjects and HIV-infected subjects supports once daily administration with oral dosing and once monthly or longer dosing with the LA formulation.

Oral 744 dosing:

Following oral administration of single doses to healthy subjects, oral 744 was readily absorbed with the maximum concentration achieved between 0.5 and 2.0 hours when dosed as solution and between 1.5 and 3.5 hours when dosed as tablet in the fasted state. Following repeat dose administration for 14 days, GSK1265744 AUC $_{(0-\tau tau)}$, C_{max}, C_{τ}, and C_{min} increased proportionally to increase in dose. Available data support time invariant PK. The effective half-life was estimated to be 40 hours. GSK1265744 has a low potential for drug interactions. Co-administration of oral 744 and etravirine (ETR) had no effect on the PK of oral 744. A designated cardiac safety study is on-going to evaluate QT prolongation.

744LA dosing:

Following a single IM or SC injection of 744LA, plasma drug concentrations increased rapidly over the first week, followed by a general trend to plateau for the remainder of the 12-week follow-up period. GSK1265744 was detected in plasma up to 48 weeks. When the dose was administered as two equally divided injections, total drug release was increased so that C_{max} was greater than dose proportional and there was a more pronounced decay in drug concentrations over time. However, overall extent of exposure ($AUC_{0-\infty tau}$) was similar. Following single dose 744LA administration, mean absorption-limited apparent terminal phase half-life ranged from 21 to 50 days, reflecting elimination from the depot site (absorption from depot site) rather than the systemic circulation.

744LA PK has been evaluated in Phase 1 studies following repeat administration in healthy HIV-uninfected subjects. Following a loading dose of 800 mg IM and three monthly (every 28 days) maintenance doses, the third monthly 200 mg SC, 200 mg IM and 400 mg IM injections

achieved geometric mean plasma Cτ of .61μg/mL, 1.72 μg/mL, and 3.22 μg/mL – 9.7-, 10-, and 19-fold above the PA-IC_{90} (0.166 μg/mL), respectively. Following two quarterly 800 mg IM doses, geometric mean plasma GSK1265744 Cτ was 1.11 μg/mL, 6.7-fold above the PA-IC_{90}.

The phase 2a ÉCLAIR study evaluated 800mg IM quarterly x 3 doses in N=127 healthy male study participants. Preliminary evaluation of PK data from that study showed that only 30 to 37% of reportable CAB trough concentrations were ≥4x PA-IC90 following each of the three injections, and 15 to 31% were below 1x PA-IC90. Graphical evaluation of the CAB concentration-time profiles suggests that drug absorption from the depot injection site was more rapid in ÉCLAIR than observed in prior studies, resulting in higher peaks and lower troughs (Figure 3).

The rationale for extended follow-up in this study (52 weeks after last injection) is two-fold: The first is to assess safety events during the prolonged interval during which drug levels have been demonstrated to be detectable in blood plasma in order to best characterize the safety profile of 744LA. The second is to characterize in detail any HIV infections that might occur during a period of declining blood plasma drug levels, including viral resistance profiles and response to antiretroviral therapy treatment.

Plans for suppressive ART treatment for participants who acquire HIV infection during the study are detailed in Section 5.21.

1.2.5 Pharmacogenomics

The purpose of the pharmacogenomic analysis will be to determine whether genetic polymorphisms play a role in governing the variability among participants in the PK and pharmacodynamics (PD) of GSK1265744 when potentially used for HIV prevention in the future. Pharmacogenomics will be an exploratory end point in this study. Genome wide association studies will be performed and compared to PK parameters. The genes to be analyzed will include those that encode the UDP-glucuronosyltransferase family of enzymes since these proteins play a role in the clearance of GSK1265744 via the conjugation of glucuronic acid.

1.3 Dose Rationale

Rationale for Cohort 1:

The Cohort 1 744LA dose rationale is based on the objective to deliver adequate drug concentrations to prevent sexual transmission of HIV. The proposed dose schedule for evaluation in humans is based on maintaining 744LA plasma concentrations well above the PA-IC_{90} value of 0.166 μg/mL, a concentration range shown to have significant antiviral activity. In a short-term monotherapy study seven HIV-infected subjects received oral 744 5 mg once daily for 10 days[44, 45]. Geometric mean Day 10 C_τ was 0.57 μg/mL, 3.4-fold above the PA-IC_{90} value, and was associated with a mean Day 11 HIV ribonucleic acid (RNA) change from baseline of -2.2 log_{10} c/mL. In LAI116482, the Phase IIb study in HIV-1 infected, antiretroviral therapy naïve adult subjects[46], suppression of HIV infection was accomplished with oral 744 at doses of 10 mg to 60 mg once daily in combination with 2 nucleotide reverse transcriptase inhibitors (NRTIs), and suppression of HIV has been maintained through 96 weeks using a dual regimen of oral 744 and rilpivirine (RPV) at oral 744 10 mg and higher. The geometric mean individual average predose concentration following oral 744 10 mg once daily was 1.35 μg/mL. Following two quarterly doses (12-weeks apart) of 744LA 800 mg IM in healthy participants (n=10), geometric mean (trough concentration) C_τ was approximately 1.11 μg/mL, approximately 6.7-fold above the PA-IC_{90} and between the 5 mg and 10 mg oral doses (Table 1). Furthermore, based on population PK modeling and simulation, 744LA 800 mg IM every 12 weeks achieves a mean concentration above the 1.35 μg/mL target based on 10 mg daily oral dosing and the lower bound of the 90% confidence interval (CI) is at 4-fold PA-IC_{90} (Figure 1). The overall range in

predicted 744LA C_τ values following 744LA 800 mg IM is similar to that following once daily dosing of oral 744 10 mg (Figure 2).

Lastly, a one week delay in dosing at steady state for 800 mg IM every 12 weeks is predicted to result in a geometric mean C_τ that is 10% lower than for dosing that is administered on schedule while remaining above the 1.35 µg/mL target (Table 1). Thus, the 800 mg IM every 12 weekly dosing schedule was expected to maintain geometric mean plasma concentrations well above the PA-IC_{90} value throughout the dosing interval and in a range of 5-10 mg oral doses with demonstrated antiviral activity.

Table 1: Summary of GSK1265744 Pharmacokinetic Parameters Following Oral Administration in HIV- Infected Subjects and IM Administration in HIV-Uninfected Subjects

Route Study Population N	GSK 1265744 Regimen	GSK1265744 PK Parameter[a]			
		C_τ or C_0 (µg/mL)	C_{max} (µg/mL)	AUC(0-τ) (µg• h/mL)	Geomean IQ C_0 or C_τ:PA-IC-90
Oral ITZ111451 HIV N=7	5 mg PO qd[b]	0.57 [33%]	1.02 [25%]	17.7 [31%]	3.4
Oral LAI116482 HIV N=14	10 mg PO qd[b]	1.35 [45%] (n=57)	2.77 [33]	45.7 [32]	8.1
IM LAI115428 HVs[c] N=9	800 mg IM every 12weeks x 2 (2nd dose interval)	1.11 [139%]	3.35 [56%]	4,417 [53%]	6.7
IM PopPK simulation HVs	800 mg IM every 12 weeks x 5 (last dose)	1.57 [56%]	ND[d]	ND[d]	9.5
IM PopPK simulation HVs	800 mg IM every 12 weeks x 5 (last dose with 1w delay)	1.41 [60%]	ND[d]	ND[d]	8.5

a. Data presented as geometric mean, [CVb%]
b. By mouth, once daily
c. Healthy volunteers
d. Not Determined

Figure 1: Predicted Mean (90% CI) 744LA Concentration-Time Profile Following 800 mg IM Every 12 Weeks x 5 in HIV-Uninfected Subjects Compared to Oral Target (1.35 µg/ml) based on 10 mg Oral Daily Dosing in HIV-Infected Subjects and to 4 times the PA-IC_{90} (0.664 µg/ml)

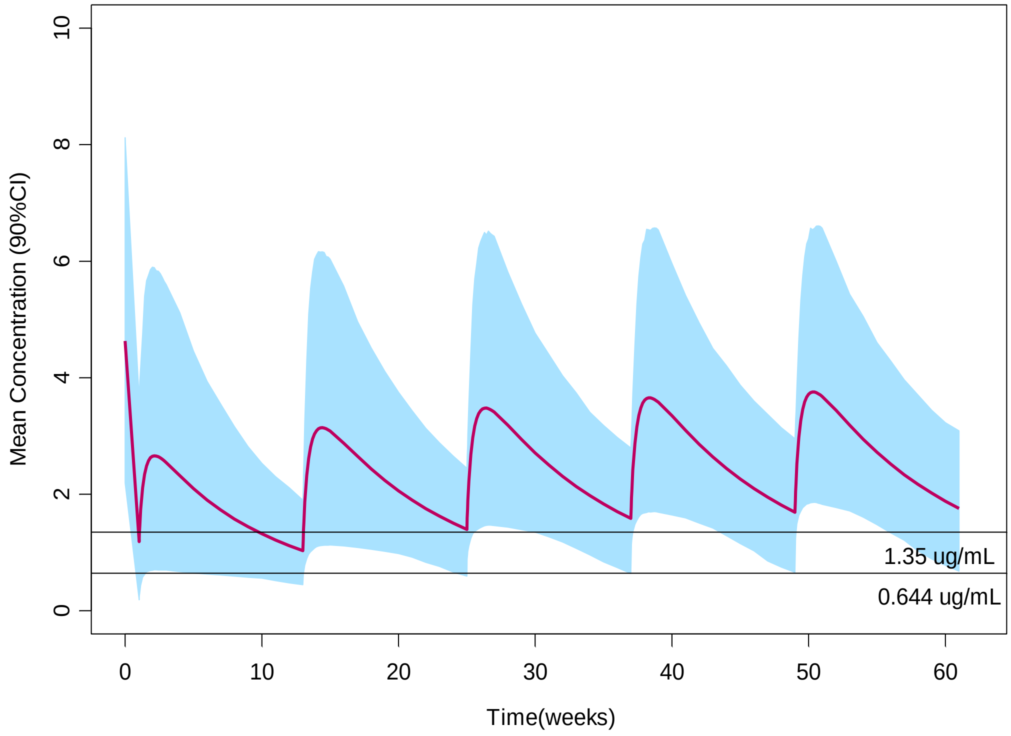

Figure 2: Predicted 744LA Trough Concentrations Following 800 mg IM Every 12 Weeks in HIV-Uninfected Subjects Compared to Oral Daily Dosing of 10mg

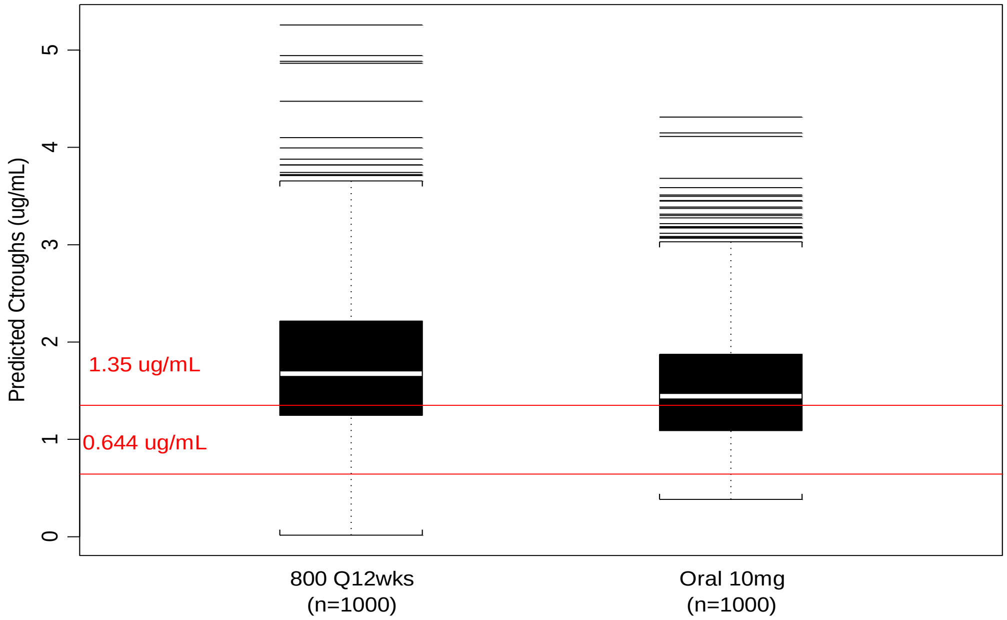

White line: median; Solid box: interquartile range (IQR); Whiskers: ±1.5 IQR; Horizontal lines: outliers

Rationale for Cohort 2:

An analogous study to HPTN 077 (201120, ÉCLAIR) was undertaken to evaluate 744LA 800mg quarterly at three time points in healthy male participants, with results becoming available during the conduct of the HPTN 077 study. Preliminary evaluation of PK data from that study showed only 30 to 37% of reportable 744 trough plasma concentrations were ≥4x PA-IC90 following each of the three injections, and 15 to 31% were below 1x PA-IC90. Graphical evaluation of the 744 concentration-time profiles suggests that absorption was more rapid among participants in the ÉCLAIR study than observed in prior studies and the related Phase 2 population PK model, resulting in higher peak and lower trough concentrations (Figure 3). Of note, the 744LA nanosuspension formulation has remained essentially unchanged throughout the clinical development program, indicating that other factors are contributing to the observed PK differences. Given this information, a regimen of 744LA 800mg every 12 weeks, particularly in male subjects, may not maintain sufficient exposures in all participants, and an amendment to the HPTN 077 dosing schedule in the protocol will enable evaluation of a second regimen (Cohort 2) in both males and females at low risk of acquiring HIV infection.

The 744 Phase 2 population PK model has been updated with ÉCLAIR PK data, significantly increasing the dataset in the model, and preliminary results indicate absorption from the injection site (Ka LA) has increased ~50% overall and ~2-fold for ÉCLAIR participants, specifically, relative to the original model. Further modeling efforts to understand which covariates may be influencing this increase in absorption are ongoing. Simulations of the 744LA 800mg every 12 weeks regimen have been re-run with the updated PK model and now reflect the predicted troughs below targets, particularly in males as observed in the ÉCLAIR study (Figure 4). The proposed regimen for Cohort 2 in this study is 744LA 600mg at Week 5, four weeks later at Week 9, and then every 8 weeks thereafter at three more time points (Week 5, 9, 17, 25 and 33). Simulations of this regimen, based on the updated PK model, predict that steady state trough concentrations will achieve targets in both males and females (Figure 5). This regimen will enable evaluation of a 600mg LA dose, which has not been studied previously without an 800mg LA loading dose, with the understanding that a higher loading dose may be required in future studies of participants at higher risk of acquiring HIV infection, particularly females who on average demonstrate slower 744LA absorption rates.

Lastly, a similar regimen has been administered to HIV infected participants in 200056 (LATTE-2). In that study, 744LA 800mg was administered as a loading dose, and two subsequent monthly doses of 744 LA 600mg were administered prior to initiating the every 8 week dosing starting at Week 8. Preliminary data for this every 8 week regimen in LATTE-2 were not available at the time of the population PK update but have been evaluated graphically by gender (Figure 6). On average, this regimen has achieved clinical targets in both males and females, further supporting the selection of an every 8 week regimen, for inclusion in the HPTN 077 study.

Figure 3. Mean (SD) Plasma 744 (CAB) following 744LA 800mg IM Q12W in ÉCLAIR and in original Phase 2 Dose Rationale Model Predictions (Sparse Time Points)

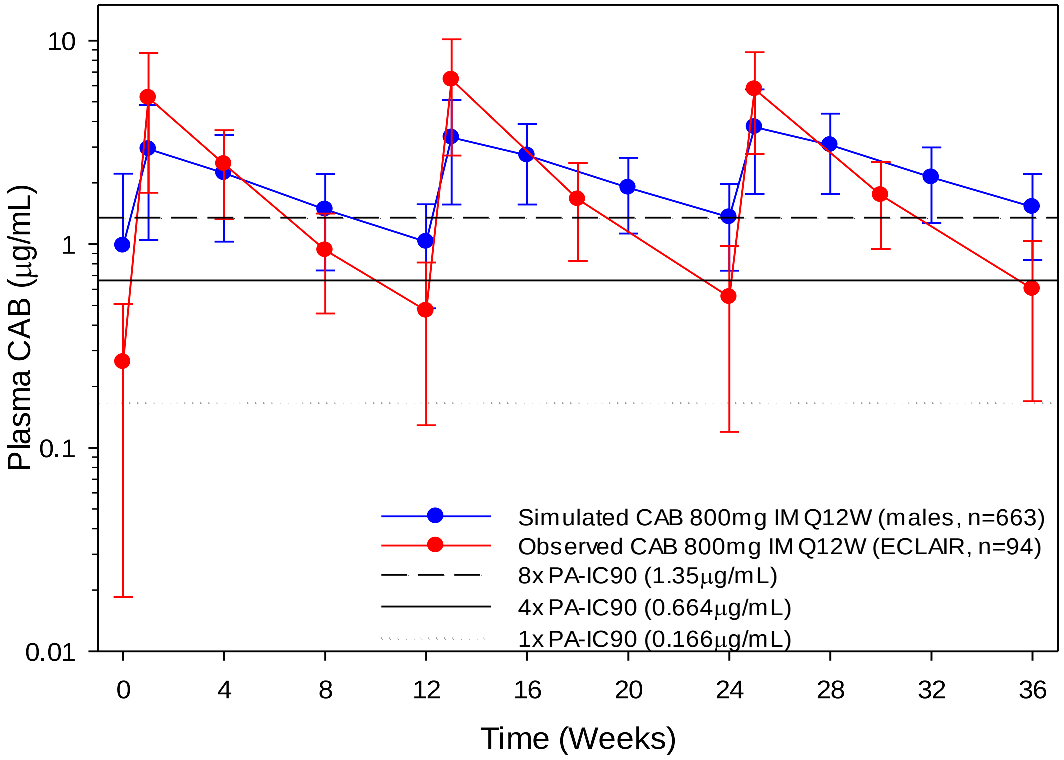

Figure 4. Simulated Concentration-Time profiles following 744 (CAB) LA 800 IM Q12W in Males and Females based on updated PopPK Model

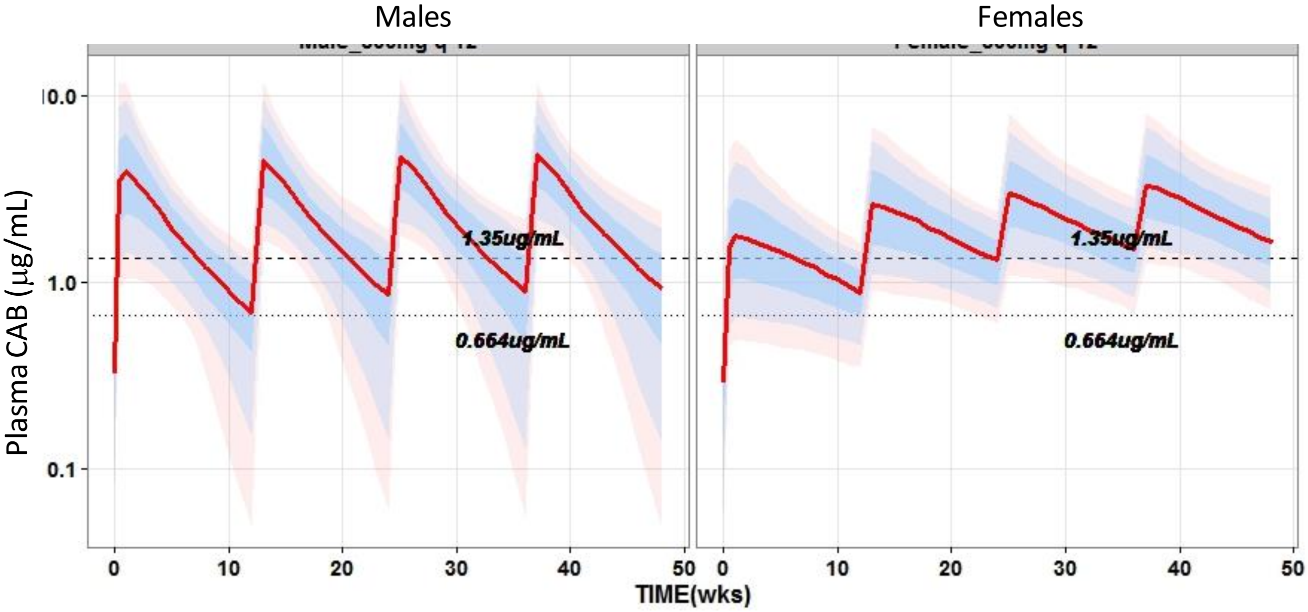

Legend: Red Line = Median, Blue band = IQR ($25^{th}/75^{th}$ percentiles), Light blue band = $10^{th}/90^{th}$ percentiles, Pink band =$5^{th}/95^{th}$ percentiles

Figure 5. Simulated Concentration-Time profiles following 744 (CAB) LA 600mg IM at Day 1, Week 4 and Q8W thereafter in Males and Females based on updated PopPK Model

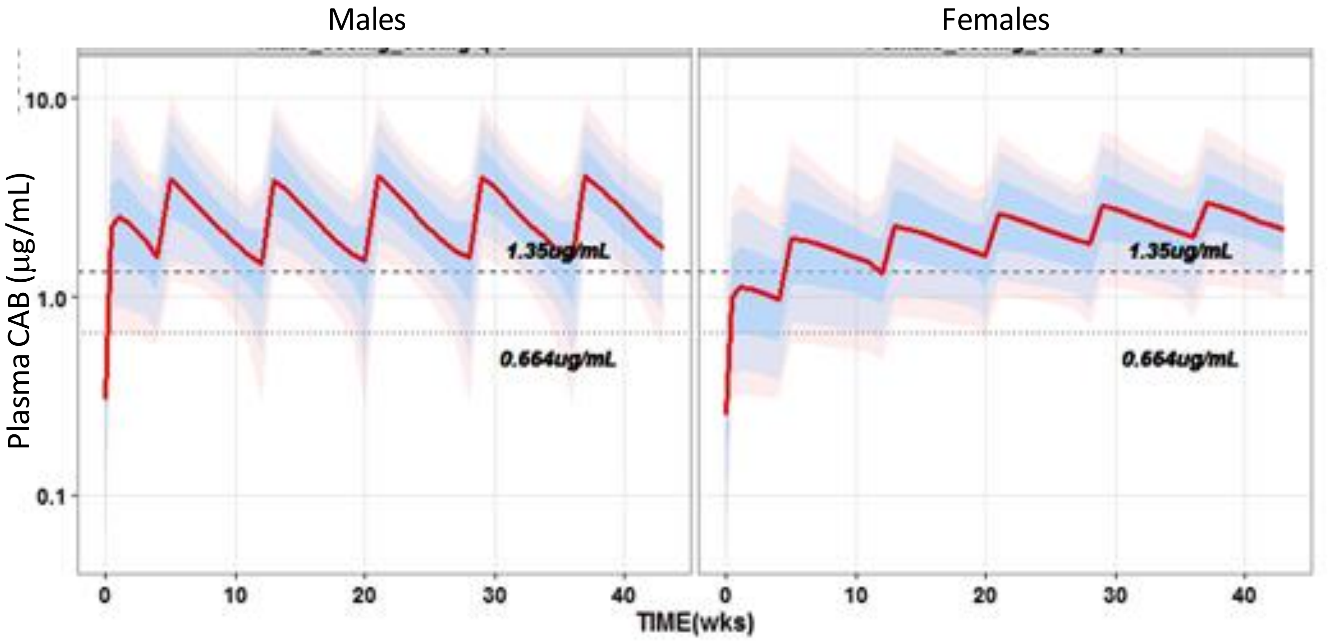

Legend: Red Line = Median, Blue band = IQR (25th/75th percentiles), Light blue band = 10th/90th percentiles, Pink band =5th/95th percentiles

Figure 6. Preliminary Concentration-Time Profiles following the 744 (CAB) LA Q8W Regimen in Males and Females (LATTE-2)

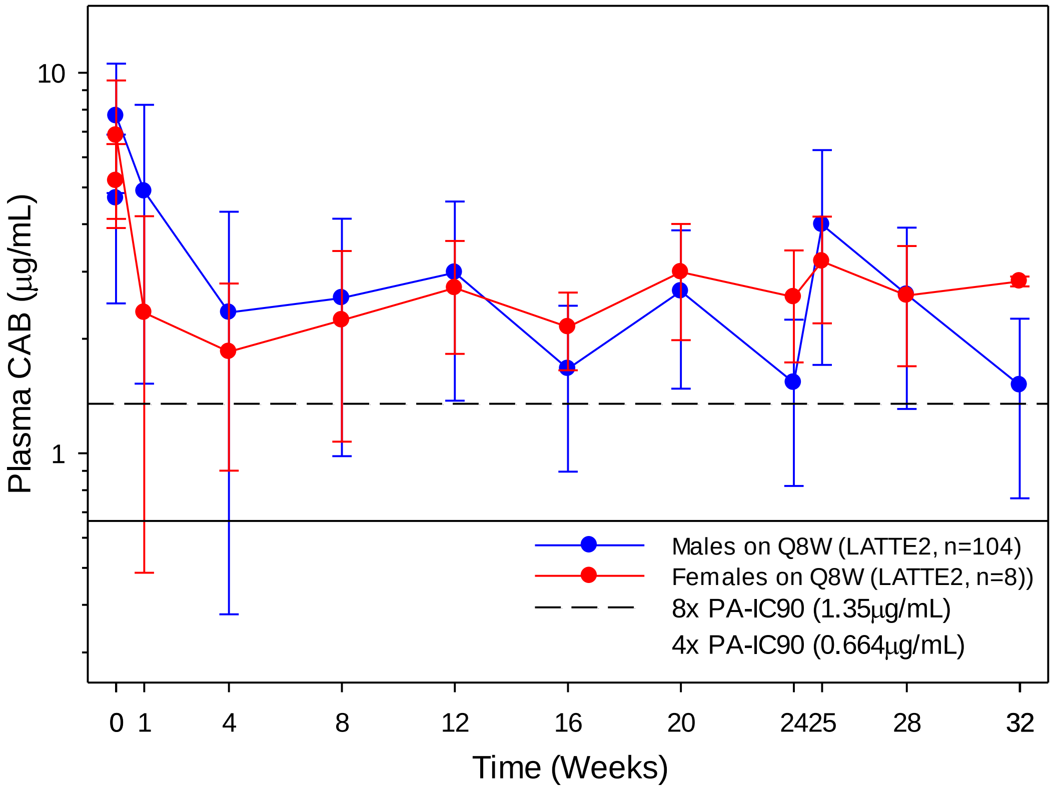

1.4 Clinical Experience to Date – GSK1265744

Through 1 July 2015, approximately 1056 adult subjects have been exposed to at least one dose of CAB (oral and/or long acting) across 18 completed or ongoing Phase 1 and Phase 2 clinical trials.

Oral 744 has been studied at doses between 5 mg - 150 mg in HIV-uninfected and HIV-infected adults. The oral formulation of GSK 1265744 has been generally well-tolerated as single or repeated doses in clinical studies of HIV-uninfected adults. Among the HIV-uninfected and HIV-infected participants who received oral formulations ranging from 5 mg-150 mg in Phase 1 and 2a studies, eleven participants receiving oral 744 were withdrawn due to potentially drug-related adverse events (AEs), including dizziness, leucopenia, and aspartate aminotransferase and alanine aminotransferase [AST/ALT]/gamma-glutamyltransferase increases, CPK increases and fatigue. No drug-related Grade 3 or Grade 4 clinical AEs or serious adverse events (SAEs) have been reported to date in Phase 1 and 2a studies. [46] One death from anoxic brain injury as a result of status epilepticus was reported in the HIV-infected Phase 2b treatment study; the event was in the clinical context of recreational drug use, but contribution from oral 744 could not be ruled out.

One hundred thirty six (136) healthy participants from completed studies have received single or repeat doses of 744 LA in Phase 1 studies at doses ranging from 100-800 mg administered as single or split IM or SC injections. To date there have been no drug-related Grade 3 or Grade 4 clinical AEs and only one participant has been withdrawn from dosing due to mild and transient

rash. Five non-drug-related SAEs have been reported: foot osteomyelitis, uterine fibroids, appendicitis, elective hysterectomy, and seizure. An additional 94 participants have received 744 LA as 800 mg IM injections every 12 weeks in the ongoing 201120 (ÉCLAIR) study. One participant was withdrawn from 744 LA therapy due to a pre-existing prostate cancer diagnosis, a non-drug-related adverse event.

Injection site reactions (ISR) occurred in the majority of subjects following IM (76% with any ISR) dosing, however, the reactions were mild and moderate (overall ISR Grade 2: 14% in IM without any Grade 3 or 4 ISRs).[46] ISRs related to 744LA injection were common but generally mild (IM: 86%, SC: 99%) with no Grade 3 ISR AEs in Phase 1 studies. The most frequent ISRs for IM dosing were pain (71%), erythema (9%) and nodules (7%)C 2013). Median IM ISR durations were approximately 5 days for pain and erythema, and approximately 22 days for nodules[46].

A Phase 2a safety and acceptability study (ÉCLAIR), designed similarly to HPTN 077, is currently in the follow-up phase of the study at 10 US-based sites. In this study, 205 individuals were screened in order to enroll and randomize 127 HIV-uninfected low-risk men. Participants received daily oral 744 30 mg or daily oral placebo in a randomized 5:1 ratio during the 4-week oral lead-in phase. One participant randomized to active study product (oral 744 and 744LA) withdrew prior to the oral lead-in due to being incarcerated. During the oral lead-in, eleven participants withdrew prior to their first injection, all of whom were randomized to 744LA, seven for adverse events and four for other reasons. Ninety-four (94) participants received at least one injection of 744LA 800 mg and 21 participants received at least one injection of placebo. Of those that started injections 95% (20 of 21) of those randomized to placebo and 93% (87 of 94) randomized to 744LA completed all three of their injections. The participant on the placebo arm that did not complete all three injections reached a protocol-defined stopping criteria, subject acquired HIV, after his second injection. Four participants on the 744LA arm withdrew after their second injection, citing injection tolerability as a primary reason. Three other participants discontinued study participation after receiving injections, for non-AE and non injection-related reasons.

Fifteen participants experienced a Grade 1 or higher abnormality of ALT and 14 experienced a Grade 1 or higher AST. There were no Grade 3 or 4 ALT elevations. AE events leading to withdrawal included transient neutropenia (three participants), transiently elevated CPK (three participants), and fatigue (one participant). Two SAEs were reported, one deep vein thrombosis on placebo that was considered possibly drug-related and one appendicitis on 744LA that was not considered drug related. Eighteen participants reported Grade 3 ISR pain.

Grade 4 treatment emergent CPK elevations with concomitant AST and/or ALT elevations were noted in four participants at the Week 4 visit, leading to early withdrawal in three participants. One of the four participants described a new rigorous exercise regimen prior to the Week 4 study visit; a second of these events resolved despite ongoing exposure to study product. All four Grade 4 abnormalities were resolving at one week follow-up visits and have subsequently returned to normal off study product.

Two Phase 2b clinical trials are in progress (GSK protocol LAI116482 [LATTE] and 200056 [LATTE-2]). In the LAI116482 study 181 participants were randomized to receive oral 744 (10, 30, or 60 mg once-daily, blinded doses) in combination with either TDF/FTC or abacavir-lamivudine.[47] (ABC/3TC). An additional 62 participants were randomized to a control arm of open-label efavirenz (EFV) 600 mg once daily in combination with one of the two NRTIs. A week 24 interim analysis demonstrated good initial efficacy and safety of 744 in combination with NRTIs. The overall response rate across the three dosing arms of oral 744 were 87% <50 c/mL (FDA snapshot analysis) with minimal differences between oral 744 doses; the control arm response rate was 74% <50 c/mL. In the “maintenance” phase, participants randomized to any of the 744 doses who had viral loads < 50c/mL prior to week 24 were transitioned to a regimen

maintaining their 744 dosing but substituting oral rilpivirine 25 mg daily for the nucleosides. Efavirenz-treated participants were kept on their “induction” regimen of dual nucleosides with efavirenz. 96-week data (representing 72 weeks of maintenance dosing)) showed virologic suppression (<50 c/mL) rates via snapshot analysis to be 79%, 85% and 93% for 744 10mg, 30mg, and 60mg daily, and 83% for the efavirenz control participants. One participant randomized to 744 10mg who successfully transitioned to rilpivirine plus 744 10 mg daily experienced virologic failure at week 48 in the context of subtherapeutic (<50% expected) 744 and rilpirivine plasma levels (partially confounded by an extreme calorie-restricted diet during weeks 40-48), and developed treatment-emergent high level integrase (Q148R) and NNRTI (E138Q) resistance[48].

Safety results through week 96 support continuation of all three oral 744 dosing arms. There have been no deaths, oral 744 drug-related SAEs or clinically significant AE trends identified to date in LAI116482. The most common clinical drug related AEs to date have been headache (15%), nausea (17%) and diarrhea (10%) with few oral 744 AEs leading to withdrawal from the study (744 - 4% vs EFV - 15%). Two HIV-infected participants receiving oral 744 60 mg + ABC/3TC with pre-existing steatohepatitis developed an ALT >10x upper limit of normal (ULN) 4 weeks and 8 weeks after study initiation. Both participants remained asymptomatic with normal bilirubin levels and hepatic function, and ALT levels normalized after drug discontinuation. No other participants have required dose adjustment or discontinuation due to a change in transaminases through week 96. One participant receiving oral 744 and rilpivirine 25 mg developed ALT values >10x ULN at Week 96 likely due to acute Hepatitis C infection.

Plasma exposures after administration of 744LA are expected to remain between the oral 744 10 mg and 30 mg exposures. At this stage of development, a lead-in of oral 744 is being employed to determine safety and tolerability in individual participants, prior to the transition to 744LA. The accumulated efficacy and safety data with oral 744 and 744 LAHIV-infected and HIV-uninfected participants supports continued clinical development for HIV treatment and PrEP.

A second Phase 2b study in HIV-infected, antiretroviral naïve adults is currently underway (LATTE-2), with 310 participants enrolled to receive oral 744 30mg + ABC/3TC for 20 weeks followed by a long acting regimen containing 744 LA + TMC278LA or continuation of oral medications. Data from this study is limited and preliminary. One participant with Hepatitis C infection and underlying stage 3/4liver fibrosis received oral 744 30mg + ABC/3TC and developed possible drug induced liver injury and a second subject on oral 744 30 mg + ABC/3TC, with steatohepatitis, developed possible drug induced liver injury approximately 6 months after initiating oral 744. See details below under Hepatic Safety. Two deaths have occurred on study, one due to a motor vehicle accident (unrelated to study products), and as noted above, one due to anoxic brain injury from status epilepticus in the setting of recreational drug use; contribution from oral 744 could not be ruled out.

Cumulative exposures of GSK1265744, through 01 July 2015, are shown in Table 2.

Table 2: Cumulative GSK1265744 Exposure Estimates from Phase 1 through Phase 2b Clinical Studies Up To 01 July 2015

Treatment Population/Dose	Duration	Completed	Ongoing/ Concluded[a]	Total
**HIV uninfected healthy subjects**		**351**	**200**	**551**
5 to 150 mg	Single dose	115	37	152
10 to 30 mg once daily	10 to 28 days	138	163[b]	301
150 mg every 12 hours	3 doses	40	0	40
100 – 800 mg IM/SC LA	Max 252 days	136[c]	114[d]	250
**HIV-infected subjects**		**15**	**490**	**505**
5 to 30 mg once daily (Ph IIa)	10 days	15	0	15
10 to 60 mg once daily (Ph IIb)	Max 1022 days[i]	0	181	181
30 mg once daily (Ph IIb)	Max 394 days	0	309	309
Up to 800 mg IM LA[e]	Max 232 days[h]		229[f]	229
**Total**		**366**	**690[b]**	**1056**
Single dose oral (5 to 150 mg)		115	37	152
Repeat dose once daily oral (10 to 60 mg)	-	153	653	806
150 mg oral every 12 hours x 3		40	0	40
Single or repeat dose LA (100 – 800 mg)	-	136[c]	343[g]	479

a. Concluded studies: study completed through follow-up; and/or clinical study report is in preparation
b. Approximately 10 are on placebo in study 201103
c. 78 received both oral and LA dosing
d. 114 received both oral and LA dosing and approximately 5 are on placebo from study 201103
e. Includes 400 mg Q4W and 600 mg Q8W dosing
f. Subset that received 30 mg once daily in study 200056
g. 343 received both oral and LA dosing and approximately 5 are on placebo from study 201103
h. Detectable CAB concentrations can remain for up to 52 weeks following the last CAB injection
i. As of 28 Dec 2014, all participants had transitioned to oral 744 30 mg in the Open-Label phase of study LAI116482 (LATTE), therefore, the longer durations apply to the 30 mg dose only

Hepatic Safety

As part of the early phase development of GSK 1265744, two participants in the HIV Treatment Phase IIb LAI116482 (LATTE) study developed Grade 4 transaminase elevations. Both were HIV-infected, had pre-existing steatohepatitis, and had Grade 1 ALT/AST elevations at screening/entry. Both were treated with ABC/3TC + 744 60 mg PO QD as part of "induction therapy" in the LATTE-1 study. Laboratory assessments at week 2, and again at week 4 (for one of the two) were normal. One participant at Week 4, and the other at Week 8 (day 57) demonstrated ALT elevations 12x ULN and 8x ULN respectively. Bilirubin remained normal, as did coagulation parameters, and the participants were clinically asymptomatic. Oral 744 was withdrawn and ALT/AST returned to normal 5 and 8 weeks thereafter, respectively.

A third participant in the HIV Treatment Phase 2b 200056 (LATTE-2) study developed Grade 4 transaminase elevations, and also > Grade 2 bilirubin elevation. This participant was HIV/HCV co-infected, with portal hypertension and Grade 3-4 fibrosis on Fibroscan but normal screening

transaminase levels. He was begun on ABC/3TC (600 mg/300 mg) + 744 30 mg PO QD. On Day 35 of treatment the participant developed ALT 5 x ULN, which peaked one week later at 7 x ULN. Drug was withdrawn and the ALT/AST and bilirubin have normalized. The participant was clinically asymptomatic. A fourth participant receiving 744 30 mg PO + ABC/3TC developed an ALT >5x ULN after approximately one year on therapy which lead to discontinuation of oral therapy, with resolution of ALT values. Initial ALT elevations were detected approximately 6 months after initiating therapy. This participant was noted to have steatohepatitis on ultrasound imaging and did not develop evidence of hepatic dysfunction. Oral 744 could not be ruled out as a contributing factor to the observed liver injury.

Central Nervous System Safety

In a single-dose study, a healthy male volunteer with a history of prior seizure activity (one episode 14 years prior), on no anti-epileptic medication, experienced an unwitnessed seizure event 270 days after a single injection of CAB LA 400 mg IM. Plasma levels of CAB were undetectable for 4 months prior to the seizure event. The event occurred in the context of consumption of approximately 10 cans of beer, and an MRI was unrevealing for pathology. An EEG was consistent with benign juvenile epilepsy; the participant recovered without additional events.

A second participant in the ongoing Phase 2b LATTE-2 trial of HIV-infected individuals received oral ABC/3TC with CAB 30mg daily for 20 weeks, and received ABC/3TC, CAB and oral RPV for 4 weeks, all without incident or clinically significant AEs or laboratory abnormalities. He was randomized to continue CAB LA 400 mg IM + RPV LA 600 mg IM Q4W. On day 349 of overall CAB treatment (139 days of oral CAB + 210 days of CAB LA exposure), the participant was found in his apartment by emergency personnel unresponsive, unconscious, and having generalized tonic-clonic seizures. The participant was hospitalized in the ICU, had recurrent seizures, and was found to have anoxic brain injury resulting in death. The participant had no prior history of seizures, and clinical history and toxicity screens suggested possible recreational substance use.

A third participant in the healthy volunteer HPTN 077 study, with a prior history of seizures treated with Dilantin had been taken off Dilantin 2 years prior to study participation, and had been seizure-free. A “spell” 1 month prior to study enrollment did not prompt re-initiation of anti-epileptics. Oral CAB 30mg (or placebo) was administered for 4 weeks without incident, and Day 62 after administration of CAB LA 800 mg IM x 1 (or placebo), the participant had an unwitnessed seizure event; he was subsequently hospitalized for transient dizziness and hemiparesis, for which work-up was unrevealing, and resolved with meclizine treatment. The participant recovered without additional events and is not planned for additional IP dosing.

1.5 Risk Assessment for GSK1265744

Since GSK1265744 is still in clinical development and exposure in humans with or without HIV infection is limited, the clinical safety profile in humans has yet to be fully elucidated. Refer to Appendix X, which outlines the risks that have primarily been identified during routine preclinical testing and/or in the clinical trial experience to date and are considered of potential relevance to clinical usage in the context of this protocol. Summaries of findings from both clinical and non-clinical studies conducted with GSK1265744 also can be found in the Investigator’s Brochure.

1.6 Acceptability

Acceptability has been conceptualized as individuals' willingness and ability to use a product over time and, when available, in the context of a range of alternative choices.[49-51] In both the fields of HIV prevention and contraception, individuals' decisions to initiate use of a product, and their ability to adhere to and sustain use of that product is strongly influenced by their knowledge and beliefs about the product, attitudes towards and experiences of product attributes and how product use fits their social/sexual context – especially in comparison to other prevention methods. As apparent from recent microbicide and PrEP clinical trials, product-related acceptability is also intertwined with the clinical trial context, including a person's understanding of the study product efficacy (which may be a placebo) and attitudes towards other trial requirements. Such factors may lead to differences between within-trial acceptability and likelihood of using a product outside of a trial context.

While assessing acceptability in a low-risk population may not be representative of the target population for PrEP, given the novelty of this investigational approach, it is believed that capturing limited and focused information on acceptability is warranted. Acceptability will be assessed through a questionnaire, including questions related to health outcomes [52,53], assessed after initiation of each injection. Semi-structured interviews may be conducted at select sites regarding their experience with study treatment. These would be conducted under a separate IRB/EC approved consent. Participation in such interviews would be optional.

1.7 Sexual Risk Behavior

Concern for "risk compensation" – a compensatory increase in numbers of sexual partners or unprotected sex acts engendered by perceived protection of a biomedical agent – has been a major concern regarding all ART-based, microbicide, and vaccine agents, including male circumcision. In the Phase III placebo-controlled trials of oral and vaginal microbicide products, such risk compensation was not observed; however, placebo-controlled studies do not provide the optimal method for evaluating effects of investigational products on such behavior.[54] Open-label demonstration projects are currently ongoing to better inform the field. The open-label continuation of the Partners-PrEP study provided one of the first opportunities to assess behavior change after study unblinding; in this study of serodiscordant heterosexual couples in Kenya and Uganda, there was an increase in rates of non-primary sexual partnering post-unblinding[55]. Mathematical modeling has suggested that even small increases in sexual risk behavior have the potential to abrogate the prophylactic efficacy of even a potent PrEP agent[56]. Assessment of sexual risk behavior change in response to a long-acting prophylactic agent would be particularly important to the field, especially with regard to cost-efficacy, risk-benefit, and implementation issues.

Sexual risk behavior will be assessed at baseline (prior to initiation of oral 744 and again prior to initiation of 744LA), and throughout the injection phase of the study and follow-up at quarterly intervals.

1.8 Rationale for Placebo-Controlled Study Design

The placebo-controlled trial (PCT) study design is a rigorous method of evaluating therapeutic options for safety and efficacy, but is ethically controversial when effective alternatives to the study agent exist. International debate has resulted in guidance to help inform such clinical trial designs[57].

In the case of 744LA for HIV prevention, the effective comparator that could be considered is daily TDF/FTC. Although daily oral TDF/FTC has been demonstrated to be effective in MSM and transgender women globally and heterosexual men and women in Africa, clinical trial efficacy has not translated into effectiveness due to poor adherence to daily dosing [28, 30]. The lack of understanding of the likely many socio-cultural, economic, and contextual correlates of

adherence to daily oral PrEP is, in large part, the genesis for the push to develop novel PrEP agents and delivery systems such as the one being investigated in the current protocol.

A PCT study design may be appropriate in specific scenarios. One of these scenarios is when withholding treatment poses negligible risk to participants.[58]

In this trial, although there is a proven effective strategy for ART-based PrEP as noted above, participants in the current protocol will be specifically selected for being at low to minimal risk of HIV acquisition. Participants will be particularly screened through the lens that randomization to the placebo arm would be acceptable to both the Investigator of Record (IoR) and participant – that is, for the well-being of the participant, lack of access to oral PrEP that is known to be safe and effective would not be expected to be problematic during the course of the trial.

Careful selection of the study population, for whom it will be determined by both the IoR at the site, study staff, and the participant that the participant's HIV risk-mitigation strategies and partner profile abrogate the need for further HIV prevention support from TDF/FTC-based PrEP will be employed. Additionally, condoms and sexual health counseling will be provided to participants in both arms. In this way, risk associated with assignment to the placebo arm is minimized.

While meeting such criteria justifies the use of placebo control, it is insufficient rationale alone; there must also be sound methodological reasons for PCT. GSK1265744 has to date had relatively limited HIV-uninfected human exposure [39], and there is need to establish the safety, tolerability, acceptability, and impact on sexual risk behavior of the injectable preparation. As the alternative comparator (if active comparator were to be employed) is an oral daily preparation, this would not only confound the acceptability and sexual risk behavior outcomes of the study, it would be providing an intervention to individuals who do not carry an indication for its use; that is, those not deemed at "significant" risk for HIV acquisition do not meet the US FDA or CDC guidance for TDF/FTC PrEP use.

2.0 STUDY OBJECTIVES AND DESIGN

2.1 Primary Objective

- Evaluate the safety and tolerability of the injectable agent GSK1265744 long acting (744LA) injectable (800 mg dose administered at three time points at 12 week intervals), through Week 41 in HIV-uninfected men and women.

- Evaluate the safety and tolerability of the injectable agent GSK1265744 long acting (744LA) injectable (600 mg dose administered at two time points at 4 week intervals, followed by three time points at 8 week intervals), through Week 41 in HIV-uninfected men and women.

2.2 Secondary Objectives

- Evaluate the safety and tolerability of GSK1265744 (daily oral 744 + 744LA) for 52 weeks of follow-up after final injection (each cohort will be analyzed separately)

- Evaluate the safety and tolerability of oral 744 from Week 0 to Week 5

- Evaluate the pharmacokinetics of 744LA administered at three time points as 800 mg IM every 12 weeks for 52 weeks of follow-up after final injection

- Evaluate the pharmacokinetics of 744LA administered at five time points as 600 mg IM at two time points every 4 weeks followed by three time points every 8 weeks for 52 weeks of follow-up after final injection

- Evaluate differential pharmacokinetics of 744LA in participants by age, gender, race, ethnicity, weight, body mass index (BMI), and smoking status

- Evaluate the acceptability of 744LA injections

- Evaluate the effect of 744LA on sexual risk behavior by change from enrollment over time during the study period

- Evaluate HIV incidence and antiretroviral drug resistance, in participants who acquire HIV infection during the study

- Evaluate the safety, tolerability and pharmacokinetics of 744LA in the subset of women who use a hormone-based contraceptive

2.3 Exploratory Objectives

- Explore relationships between safety parameters and GSK1265744 concentrations and/or participant demographic factors

- Explore genetic factors that may impact the pharmacokinetics of 744LA

- Perform secondary laboratory assessments which may include evaluation of factors related to HIV infection, hepatitis infection, or sexually transmitted infections (STIs); antiretroviral (ARV) drug use; pharmacogenomics; characterization of HIV in infected participants; and evaluation of laboratory assays related to the study objectives

2.4 Study Design and Overview

This is a Phase 2a, randomized, multi-site, two-arm, double-blind study of the safety, tolerability, pharmacokinetics, and acceptability of 744LA. Eligible participants will be enrolled into two cohorts, and will begin an oral lead-in phase in which they will be randomized to receive daily oral 744 (30 mg tablets) or matching placebo for 4 weeks, followed by a one week washout period, to assess safety prior to receiving 744LA. Following final safety lab assessments from the oral regimen phase, participants will enter the injection phase and receive IM injections of 744LA or placebo at three time points at 12 week intervals in Cohort 1, and five time points at 4 and 8 week intervals in Cohort 2.

Approximately 194 participants will be divided into two dose cohorts and will be randomized to two arms in a ratio of 3:1 as follows:

Cohort 1

Arm 1: Approximately 79 participants will receive daily oral 744 (30 mg tablets) for 4 weeks, followed by a one week washout period, to establish safety and tolerability, followed by intra-muscular (IM) gluteal injections of 800 mg of 744LA (administered as two sequential 400 mg gluteal injections) at three time points at 12 week intervals as: Week 5, Week 17, and Week 29.

Arm 2: Approximately 27 participants will receive daily oral matching placebo and IM injections of vehicle-matched placebo on the same schedule as Arm 1.

Cohort 2

Arm 1: Approximately 66 participants will receive daily oral 744 (30 mg tablets) for 4 weeks, followed by a one week washout period, to establish safety and tolerability, followed by intra-muscular (IM) gluteal injections of 600 mg of 744LA (administered as one 600 mg gluteal injection) at five time points at 4 and 8 week intervals as: Week 5, Week 9, Week 17, Week 25, and Week 33.

Arm 2: Approximately 22 participants will receive daily oral matching placebo and IM injections of vehicle-matched placebo on the same schedule as Arm 1.

All participants will receive HIV testing with pre- and post-test counseling, in addition to risk-reduction counseling and condoms and lubricant. All participants will be followed according to the Schedule of Evaluations and Procedures provided in Appendices I-IX.

2.4.1 Participating Sites/Institutions

Participating sites are listed in the SSP Manual, and are located in Brazil, SSA, and the US.

2.4.2 Study Duration

This study will be approximately 3 years in length. Accrual will require approximately 16-24 weeks (4-6 months) for each cohort. Study participants in both cohorts will receive oral tablets for 4 weeks, followed by a one-week washout period. Cohort 1 will then receive injections at three points over 24 weeks (6 months); Cohort 2 will receive injections at five time points over 28 weeks (7 months). Participants in the active study product arms will be followed for 52 weeks following their last injection. Participants in the placebo arm will be followed until 52 weeks after their last injection or until the last participant in the active study product arm in Cohort 2 completes their Week 41 visit, whichever comes first, after which the cohorts will be unblinded to assess the primary endpoint. When all procedures related to unblinding are completed, participants in the placebo arm of the respective cohort will no longer be followed.

3.0 STUDY POPULATION

Approximately 194 hundred HIV-uninfected men and women will be included in this study. A parallel study is being conducted in the US in men who have sex with men; as such, this study will enroll a higher proportion of women (60%). Each site will be asked to work with their community advisory boards (CABs) and outreach, education and recruitment teams to develop a recruitment plan appropriate for their local population. Participants will be selected for the study according to the criteria in Sections 3.1 and 3.2. Study participants will be recruited as described in Section 3.3 and assigned to a study arm as described in Section 7.5. Requirements related to participant retention and withdrawal from the study are described in Sections 3.5 and 3.6, respectively. Individual sites will be given enrollment targets and gender distribution targets commensurate with study population needs, adjusted for local epidemiology, such that overall cross-site enrollment meets overall protocol goals. Enrollment targets may be substantially different in terms of gender distribution across sites.

3.1 Inclusion Criteria

Participants who meet all of the following criteria are eligible for inclusion in this study:

- Men and women, 18-65 years old at the time of screening
- Willing to provide informed consent for the study
- In the last 12 months (at the time of screening):
    - No self-reported unprotected anal or vaginal intercourse with someone known to be HIV-infected or of unknown HIV infection status
    - No self-reported stimulant use (cocaine [including crack], methamphetamine, or non-physician prescribed pharmaceutical-grade stimulants) or inhaled nitrate
    - No self-reported illicit injection drug use of any kind
    - No self-reported diagnosis of GC, CT, incident syphilis, bacterial vaginosis, or trichomoniasis
    - Not reporting greater than or equal to 5 different sexual partners, regardless of use of protection or knowledge of HIV status

  Note: "Unknown HIV status" is defined as a partner not known to be HIV infected or HIV uninfected by the potential participant (as assessed by the Investigator of Record or designee). The Clinical Management Committee (CMC) – refer to Section 6.1 for further information regarding the CMC – should be contacted regarding any questions about how to apply or interpret the risk eligibility criteria. The SSP Manual should also be referred to for additional guidance regarding appropriate categorization of participants as low risk.

- In general good health, as evidenced by the following laboratory values, which must be from specimens obtained within 45 days prior to study enrollment:
    - Non-reactive / negative HIV test results*
    - Hemoglobin > 11 g/dL,
    - Absolute neutrophil count > 750 cells/mm^3
    - Platelet count ≥ 100,000/mm^3
    - Calculated creatinine clearance ≥ 70 mL/minute using the Cockcroft-Gault equation
    - Alanine aminotransferase (ALT) and aspartate aminotransferase (AST) ≤ upper limit of normal (ULN)
    - Total bilirubin ≤ Grade 1 and direct bilirubin ≤ ULN
    - Hepatitis B surface antigen (HBsAg) negative
    - Hepatitis C Ab negative

Note: Chemistry and hematology parameters which do not meet the inclusion criteria above may be repeated once during screening.

*HIV uninfected, based on HIV test results obtained at Screening and just prior to randomization at the Enrollment visit. All HIV test results from the Screening visit must be obtained and must all be negative/non-reactive. This includes testing for acute HIV infection, which must be performed within 14 days of Enrollment. In addition, at least one HIV test result obtained at the Enrollment visit must be obtained prior to randomization in to the study and must be negative/non-reactive. Individuals who have one or more reactive or positive HIV test results will not be enrolled, even if subsequent confirmatory testing indicates that they are not HIV-infected. Refer to SSP Manual.

- No alcohol or substance use that, in the opinion of the study investigator, would interfere with the conduct of the study (e.g., provided by self-report, or found upon medical history and examination or in available medical records). See Section 3.2 for additional exclusion criteria related to substance use.
- No medical condition that, in the opinion of the study investigator, would interfere with the conduct of the study (e.g., provided by self-report, or found upon medical history and examination or in available medical records)
- Willing to undergo all required study procedures

Additional requirements for all women:

- If of reproductive potential (defined as pre-menopausal women who have not had a sterilization procedure per self-report, such as hysterectomy, bilateral oophorectomy, tubal ligation or salpingectomy), **must** have a negative urine pregnancy test performed (and results known) within 48 hours before initiating the protocol-specified medication(s) at enrollment. Women are considered menopausal if they have not had a menses for at least 12 months and have a follicle stimulating hormone (FSH) level of greater than 40 IU/L; if FSH testing is not available, they must have had amenorrhea for 24 or more consecutive months. (FSH testing is not a protocol requirement.)
- If of reproductive potential and participating in sexual activity that could lead to pregnancy, women must agree to use a form of contraception during the trial and for 30 days after stopping the oral study medication or for 52 weeks after stopping the long acting injectable from the list below:
  - Intrauterine device (IUD) or intrauterine system (IUS) that meets <1% failure rate as stated in the product label
  - Hormone-based contraceptive

3.2 Exclusion Criteria

Participants who meet any of the following criteria will be excluded from this study:

- One or more reactive or positive HIV test result at Screening or Enrollment, even if HIV infection is not confirmed
- Any active sexually transmitted infection detected by laboratory testing at Screening
- Co-enrollment in any other HIV interventional research study or other concurrent studies which may interfere with this study (as provided by self-report or other available documentation. Exceptions may be made if appropriate after consultation with the CMC.)
- Past or current participation in HIV vaccine trial. An exception will be made for participants that can provide documentation of receipt of placebo (not active arm).

- Use of ART (e.g., for non-occupational post-exposure prophylaxis [PEP] or PrEP) in the 90 days prior to study entry
- Clinically significant cardiovascular disease, including:
    - ECG (one repeat ECG is allowed during screening; may be performed on the same day) with:
        - heart rate < 45 or > 100 beats per minute for men, and <50 or >100 beats per minute for women
        - QRS duration >120 msec
        - QTc interval (B or F) > 450 msec
        - evidence of previous myocardial infarction (pathologic Q waves, S-T segment changes (except early repolarization))
        - any clinically significant conduction abnormality (including but not specific to left or right complete bundle branch block, AV block [2nd degree (type II) or higher], Wolf Parkinson White [WPW] syndrome) (Any question of clinical significance should be referred to the CMC for adjudication)
        - sinus pauses > 3 seconds
        - any clinically significant arrhythmia which, in the opinion of the Investigator of Record or designee, will interfere with the safety for the individual participant (any question of clinical significance should be referred to the CMC for adjudication)
        - or history of non-sustained (≥3 consecutive ventricular ectopic beats on ECG at screening or entry) or sustained ventricular tachycardia
    - History/evidence of symptomatic arrhythmia, angina/ischemia, coronary artery bypass grafting (CABG) surgery or percutaneous transluminal coronary angioplasty (PTCA) or any clinically significant cardiac disease
    - Systolic blood pressure at screening outside the range of 90-140 mmHg or diastolic blood pressure is outside the range of 45-90 mmHg (confirmed on repeat measurement)
- Underlying skin disease or currently active skin disorder (e.g., infection, inflammation, dermatitis, eczema, psoriasis, urticaria). Mild cases of localized acne or folliculitis or other mild skin condition may not be exclusionary at the discretion of the Investigator of Record or designee in consultation with the CMC
- Has a tattoo or other dermatological condition overlying the buttock region which in the opinion of the IoR or designee, in consultation with the CMC, may interfere with interpretation of injection site reactions
- Current or chronic history of liver disease (e.g., non-alcoholic or alcoholic steatohepatitis) or known hepatic or biliary abnormalities (with the exception of Gilbert's syndrome, asymptomatic gallstones, or cholecystectomy)
- Coagulopathy (primary or iatrogenic) which would contraindicate IM injection (concomitant anticoagulant or anti-platelet therapy use should be discussed with the CMC)
- Active or planned use of prohibited medications as described in the Investigator’s Brochure or listed in the SSP Manual (provided by self-report, or obtained from medical history or medical records)
- A score of ≥ 8 on the WHO Alcohol Use Disorders Identification Test (AUDIT) at screening. Note: A score of ≥ 8 indicates a medium to high level of problem alcohol use.
- For women, pregnant or currently breastfeeding, or intends to become pregnant and/or breastfeed during the study
- A history of seizure disorder, by self-report

3.3 Recruitment Process

Sites will be responsible for developing appropriate recruitment processes that are geared toward their respective local communities. All advertising materials must undergo approval by each participating site's Institutional Review Board (IRB)/Ethics Committee (EC).

3.4 Co-Enrollment Guidelines

Participants in this study will not be allowed to take part in other concurrent research studies during their participation in the study. This is due in part to concerns about participant study burden, American Red Cross-mandated limitations on per-unit-time phlebotomized blood volumes, to avoid potential unblinding of this or other studies, and to avoid confounding in the interpretation of the study data. Any requests for co-enrollment in observational studies should be directed to the CMC.

3.5 Participant Retention

Once a participant enrolls in this study, the study site will make every effort to retain him or her for the entire follow-up period. Optimally, participant retention procedures will be established such that loss rates do not exceed the range that allow the incidence rate of the primary study outcome to be reliably estimated (i.e., a maximum of 10% as assumed in the sample size calculation). Study site staff are responsible for developing and implementing local standard operating procedures to target this goal. Components of such procedures may include:

- Thorough explanation of the study visit schedule and procedural requirements during the informed consent process and re-emphasis at each study visit.
- Thorough explanation of the importance of both arms to the overall success of the study.
- Collection of detailed locator information at the study Screening Visit, and active review and updating of this information at each subsequent visit, including where the participant lives and other locator venues.
- Use of appropriate and timely visit-reminder mechanisms.
- Immediate and multifaceted follow-up on missed visits.
- Mobilization of trained staff to complete in-person contact with participants at their homes and/or other community locations.
- Regular communication with the study community at large to increase awareness about HIV/AIDS and explain the purpose of HIV prevention research and the importance of completing research study visits.

3.6 Participant Withdrawal

Regardless of the participant retention methods described in Section 3.5, participants may voluntarily withdraw from the study for any reason at any time. The Investigator also may withdraw participants from the study in order to protect their safety and/or if they are unwilling or unable to comply with required study procedures after consultation with the Protocol Chair, Division of AIDS (DAIDS) Medical Officer, Statistical and Data Management Center (SDMC) Protocol Statistician, representatives from the Laboratory Center (LC), the Leadership and Operations Center (LOC) Clinical Research Manager (CRM), and others.

Participants also may be withdrawn if the study sponsor, government or regulatory authorities (including Office for Human Research Protections [OHRP] and the FDA) or site IRBs terminate the study prior to its planned end date.

Every reasonable effort will be made to complete a final evaluation of participants who terminate from the study prior to the final protocol-dictated study week, and study staff will record the reason(s) for all withdrawals from the study in participants' study records. In such cases, the Investigator of Record or designee must contact the CMC for guidance regarding final evaluation procedures.

4.0 STUDY PRODUCT CONSIDERATIONS

4.1 Study Product Regimens/Administration/Formulation Content

Study Product Regimens

Both Cohorts:

Oral lead-in phase: Participants will be randomized 3:1 to one of two study arms:

Arm 1: GSK1265744 tablets 30 mg – one tablet orally daily for 4 weeks, with or without food

Arm 2: Placebo for GSK1265744 – one tablet orally daily for 4 weeks, with or without food

Injectable phase: After a one week washout period to assess safety, participants will begin injections (with the same randomization as the oral lead-in phase) as:

Cohort 1:

Arm 1: GSK1265744 (744 LA) 800 mg administered as two 2 mL (400 mg) IM injections in the gluteal muscle at Weeks 5, 17 and 29

Arm 2: Placebo for GSK1265744 (744 LA Placebo) administered as two 2 mL (400 mg) injections in the gluteal muscle at Weeks 5, 17 and 29

Cohort 2:

Arm 1: GSK1265744 (744 LA) 600 mg administered as one 3mL (600 mg) IM injections in the gluteal muscle at Weeks 5, 9, 17, 25 and 33

Arm 2: Placebo for GSK1265744 (744 LA Placebo) administered as one 3 mL (600 mg) injections in the gluteal muscle at Weeks 5, 9, 17, 25 and 33

Study Product Formulations

Oral product:

GSK1265744 tablets 30 mg are formulated as white to almost white oval-shaped coated tablets for oral administration. The tablets are packaged in high density polyethylene (HDPE) bottles with child-resistant closure that include an induction seal. The bottles contain a desiccant. The bottles should be stored up to 25 degrees Celsius (25° C) and protected from moisture.

Placebo tablets for GSK1265744 are formulated as white to almost white oval-shaped coated tablets to visually match the active GSK1265744 tablets. The tablets are packaged in high density polyethylene (HDPE) bottles with child-resistant closure that include an induction seal. The bottles contain a desiccant. The bottles should be stored up to 25 degrees Celsius (25° C) and protected from moisture.

Injectable Suspension:

The GSK1265744LA is formulated as a sterile white to slightly colored suspension containing 400mg/2mL of GSK1265744 for administration by intramuscular (IM). The product is packaged in a 3 mL vials. Each vial is for single use containing a nominal fill of 2mL (400 mg), and does not require dilution prior to administration. The GSK1265744LA injectable suspension is to be stored at 2 degrees celsius to 30 degrees celsius (2° C – 30° C), do not freeze.

Placebo for GSK1265744 Injectable Suspension will be Sodium Chloride for Injection USP, 0.9%.

Syringes containing active and placebo study product will be covered with an overlay by the study Pharmacist of Record or designee prior to dispensing in order to maintain the blind, as per the SSP Manual.

The study product being tested in this study is investigational and not yet approved by the US FDA for the treatment or prevention of HIV-1 infection. Further information on the study product is available in the Investigator's Brochure, which will be provided by the DAIDS Regulatory Support Contract (RSC).

4.2 Study Product Acquisition and Accountability

Study product is being provided by ViiV Healthcare.

Study Product Acquisition

All study products (active drug and placebo) will be supplied by the National Institute of Allergy and Infectious Diseases (NIAID) Clinical Research Products Management Center (CRPMC). The study site pharmacist can obtain all the study products through the CRPMC by following the instructions in the *Pharmacy Guidelines and Instructions for DAIDS Clinical Trial Networks*, and instructions in the SSP Manual.

Study Product Accountability

The site pharmacist is required to maintain complete records of all study products received from the CRPMC and subsequently dispensed to study participants. All study products must be stored in the pharmacy. All unused study products must be returned to the CRPMC after the study is completed, terminated or otherwise instructed by the study sponsor. The procedures to be followed are provided in the *Pharmacy Guidelines and Instructions for DAIDS Clinical Trials Networks.*

4.3 Toxicity Management

Toxicity management guidelines can be found in Appendix XI.

4.4 Concomitant, Prohibited, and Precautionary Medications

Information regarding prohibited and precautionary concomitant medications can be found in the SSP Manual. The SSP Manual will be revised (as a whole or as a Memorandum of Changes) and re-issued when changes are made.

All concomitant medications/preparations (prescription and non-prescription) including alternative/complementary medications/preparations (e.g., herbs, vitamins, etc.) taken within 30 days prior to enrollment and anytime thereafter during study participation will be collected in the study participant's chart and on study case report forms (CRFs). Alcohol and recreational or street drug use reported by a participant during the study will be recorded in the participant's study chart only (and <u>not</u> captured on the concomitant medication log for inclusion in the study database).

5.0 STUDY PROCEDURES

Overviews of the study visit and procedures schedules for both cohorts are presented in Appendices I-VIII (Cohort 1, Appendices I-III; Cohort 2, Appendices IV-VIII). Presented below is additional information on visit-specific study procedures. Detailed instructions to guide and standardize all study procedures across sites are included in the SSP Manual.

5.1 Screening: Both Cohorts

It is the responsibility of the local site to determine the best approach to screening. For each participant, independent written informed consent will be obtained before any study procedures are initiated. Screening procedures may occur over one or more visits. The SSP Manual provides additional information regarding the procedures outlined below, including clinical and laboratory procedures and requirements. Enrollment must occur within 45 days of specimen collection (except for HIV RNA, which must be collected and results obtained 14 days before enrollment) for the clinical and laboratory evaluation and procedures outlined below. The following evaluations and procedures will occur as part of screening:

Administrative, Behavioral, and Regulatory Procedures

- Informed consent
- Locator information
- HIV counseling
- Administer AUDIT questionnaire
- Offer condoms and lubricant

Clinical Procedures

- Target medical history (including bleeding history) and targeted physical exam for ascertainment of eligibility, and concomitant medications
- ECG
- Blood collection
- Urine collection for GC/CT testing (men and women)
- Urine pregnancy testing for women of reproductive potential
- Vaginal swab collection for GC/CT testing (optional alternate collection for women if urine not used)

Laboratory Evaluations

- HIV testing (see SSP Manual)., including testing for acute HIV infection within 14 days prior to enrollment
- Hepatitis testing: HBsAg, HBsAb, HBcAb, HCAb
- CBC with differential
- Chemistry testing (see Appendix I or IV)
- Liver function testing (AST, ALT, total and direct bilirubin, and alkaline phosphatase)
- Syphilis testing
- GC/CT testing (urine for men and either urine or vaginal swab for women)
- Plasma storage
- Urine pregnancy testing (for women of reproductive potential)

Sites will follow the HIV testing algorithm for Screening included in the SSP Manual. If a reactive/positive result is obtained for any HIV test, the person is not eligible for the study. Additional testing to confirm suspected HIV infection during Screening will be performed in accordance with local guidelines. If HIV infection is confirmed, participants will receive counseling and be referred for appropriate care, as necessary.

Participants who have a positive test result for a sexually transmitted infection (STI) at Screening are not eligible for enrollment; all positive results will be reported per local legal reporting

guidance and referred for appropriate care and treatment. STI treatment will not be provided by the study protocol.

If a participant is deemed eligible after Screening, they will be asked to return to the site for Enrollment. Those who are not eligible will be informed that they do not meet the elibility criteria for the study and will be referred for appropriate medical care, if necessary.

Potential participants may be rescreened once at the discretion of the IoR or their designee. However, potential participants with clinically significant cardiovascular disease as outlined in the exclusion criteria in Section 3.2, or having symptoms consistent with acute HIV infection (per IoR or designee) or a reactive HIV test may not be re-screened.

Eligible participants should be reminded that the use of anticoagulant and/or antiplatelet medications as outlined in the SSP Manual are prohibited within 7 days before and 7 days after injections. Reminders should be built in to the concomitant medication history.

5.2 Week 0: Enrollment – Both Cohorts

Administrative, Behavioral, and Regulatory Procedures

- Locator information
- Demographic information
- Randomization
- HIV counseling
- Offer condoms and lubricant
- Adherence counseling
- Behavioral Assessment
- Acceptability Assessment

Clinical Procedures

- Complete medical history and complete physical exam, including concomitant medications (may be performed during screening at the discretion of the Investigator of Record or their designee)
- ECG
- Blood collection (collect prior to administration of study product)
- Urine collection for urinalysis
- Urine pregnancy testing for women of reproductive potential
- Rectal swab for GC/CT testing (men and women) (at US sites, may be performed during Screening at the discretion of the Investigator of Record or his/her designee)
- Provide oral drug

Laboratory Evaluations

- HIV testing (see SSP Manual)
- CBC with differential
- Chemistry testing
- Liver function testing (AST, ALT, total bilirubin, alkaline phosphatase)
- Fasting lipid profile (participants should be fasting for at least 8 [preferably 12] hours prior to sample collection)
- Urinalysis (protein and glucose)
- GC/CT testing (rectal swab for men and women); for non-US sites, to be batched and shipped to the HPTN Laboratory Center for testing (see SSP for details). For US sites, to be performed locally.
- Plasma storage
- Sample storage for Pharmacogenomic testing (at the discretion of the study site). For sites that store samples for this testing, participants must provide specific consent).

NOTE: All HIV test results including testing for acute HIV testing from Screening and at least one HIV test result from Enrollment must be available and confirmed to be negative/non-reactive PRIOR to provision of study product. In addition, for women of reproductive potential, a pregnancy test result from the Enrollment visit must be available and confirmed to be negative PRIOR to the provision of study product. Results from the chemistry testing, LFTs, lipid profile, hematology testing, and urinalysis from this visit are NOT required prior to the issue of study product.

5.3 Weeks 2 and 4: Oral Safety Visits (Week 4 is Post-Oral Safety Visit) – Both Cohorts

Administrative, Behavioral, and Regulatory Procedures

- Locator information
- HIV counseling
- Offer condoms and lubricant
- Adherence counseling (Week 2 only)
- Returned pill count

Clinical Procedures

- Targeted medical history and targeted physical exam, including concomitant medications and central nervous system (CNS) symptoms assessment
- ECG (Week 4 only)
- Blood collection
- Urine collection for urine pregnancy testing for women of reproductive potential

Laboratory Evaluations

- HIV testing (see SSP Manual)
- CBC with differential
- Chemistry testing
- Liver function testing (AST, ALT, total bilirubin, alkaline phosphatase)
- Plasma storage

NOTES for Week 2:

- All HIV test results from previous visits and at least one HIV test result from the Week 2 visit must be available and reviewed at this visit. If any of these tests is reactive/positive, study drug should be discontinued (see also Section 5.21). In addition, for women of reproductive potential, a pregnancy test result from the Week 2 visit must be available and confirmed to be negative PRIOR to the provision of study product. Results from other laboratory tests (e.g., chemistry, LFTs, hematology) from the Week 2 visit are NOT required prior to the issue of study product.

- A Grade 1 AST/ALT abnormality reported at Week 2, regardless of relatedness to the study product, should be confirmed within one week. Study drug may continue until the confirmatory results are available. If the repeat value is < Grade 1, study drug may continue to Week 4. If the repeat value is Grade 1 or higher, study product should be stopped, and the participant will be prohibited from entering the injection phase of the study, and will be terminated from the study. All such cases must be reported to the CMC. In addition, participants will be followed with weekly AST/ALT assessments until they return to < Grade 1.

- A Grade 2 or higher AST/ALT abnormality reported at Week 2 of the oral phase, regardless of relatedness to the study product, will prohibit a participant from entering the injection phase of the study, and the participant will be terminated from the study. All such cases must be reported to the CMC. In addition, participants will be followed with weekly AST/ALT assessments until they return to < Grade 1.

NOTE for Week 4: Results from all Week 4 clinical and laboratory evaluations (e.g., chemistry, LFTs, hematology) must be available and be reviewed by the IoR or their designee prior to the first injection at Week 5. A Grade 1 (unconfirmed) or higher AST/ALT abnormality reported at Week 4 during the oral phase, regardless of relatedness to the study product, will prohibit a participant from entering the injection phase of the study, and the participant will be terminated from the study. All such cases must be reported to the CMC. In addition, participants will be followed with weekly AST/ALT assessments until they return to < Grade 1.

NOTE for Weeks 2 and 4: The CMC should be contacted for guidance regarding pill counts resulting in less than 75% adherence prior to the Week 5 First Injection Visit, or any other concerns regarding adherence.

5.4 First Injection Visit: Both Cohorts - Week 5

Administrative, Behavioral, and Regulatory Procedures

- Locator information
- HIV counseling
- Offer condoms and lubricant
- Behavioral assessment

Clinical Procedures

- Targeted medical history and targeted physical exam, including concomitant medications and CNS symptoms assessment
- Blood collection
- Urine pregnancy testing for women of reproductive potential
- Administer injection (with counseling)

Laboratory Evaluations

- HIV testing (see SSP Manual)
- CBC with differential
- Chemistry testing
- Liver function testing (AST, ALT, total bilirubin, alkaline phosphatase)
- Plasma storage (must be drawn PRIOR to the injection)

NOTE: All HIV test results from previous visits and at least one HIV test result from the Week 5 visit must be available and confirmed to be negative/non-reactive PRIOR to injection of study product. The injection must not be given if any HIV test is reactive/positive.

For women of reproductive potential, a pregnancy test result from the Week 5 visit must be available and confirmed to be negative PRIOR to injection of study product. The injection must not be given if the pregnancy test is positive.

Results from the other laboratory evaluations (e.g., chemistry, LFTs, hematology) from the Week 5 visit are NOT required prior to injection.

Results from all Week 4 clinical and laboratory evaluations (e.g., chemistry, LFTs, hematology) must be available and be reviewed by the IoR or their designee prior to injection.

5.5 Safety Visits: Cohort 1 - Week 6, 9, 13; Cohort 2 - Week 6 only

Administrative, Behavioral, and Regulatory Procedures

- Locator information
- HIV counseling
- Offer condoms and lubricant
- Acceptability assessment (Week 6 only)

Clinical Procedures
- Targeted medical history and targeted physical exam, and concomitant medications and CNS symptoms assessment
- ECG (Week 6 and 9 only for Cohort 1, and Week 6 only for Cohort 2)
- Blood collection
- ISR evaluation

Laboratory Evaluations
- HIV testing (see SSP Manual)
- CBC with differential
- Chemistry testing
- Liver function testing (AST, ALT, total bilirubin, alkaline phosphatase)
- Plasma storage

5.6 Second Injection Visit: Cohort 1 – Week 17; Cohort 2 – Week 9

Administrative, Behavioral, and Regulatory Procedures
- Locator information
- HIV counseling
- Offer condoms and lubricant
- Behavioral assessment

Clinical Procedures
- Targeted medical history and targeted physical exam, including concomitant medications and CNS symptoms assessment
- Blood collection
- Urine pregnancy testing for women of reproductive potential
- Administer injection (with counseling)

Laboratory Evaluations
- HIV testing (see SSP Manual)
- CBC with differential
- Chemistry testing
- Liver function testing (AST, ALT, total bilirubin, alkaline phosphatase)
- Plasma storage (must be drawn PRIOR to the injection)

NOTE: All HIV test results from previous visits and at least one HIV test result from the current visit must be available and confirmed to be negative/non-reactive PRIOR to injection of study product. The injection must not be given if any HIV test is reactive/positive.

For women of reproductive potential, a pregnancy test result from the current visit must be available and confirmed to be negative PRIOR to injection of study product. The injection must not be given if the pregnancy test is positive.

Results from the other laboratory evaluations (e.g., chemistry, LFTs, hematology) from the current visit are NOT required prior to injection.

Results from all the previous safety visit clinical and laboratory evaluations (e.g., chemistry, LFTs, hematology) must be available and be reviewed by the IoR or designee prior to injection.

5.7 Safety Visits: Cohort 1 - Week 18 and 23; Cohort 2 – Week 10 and 13

Administrative, Behavioral, and Regulatory Procedures
- Locator information
- HIV counseling
- Offer condoms and lubricant

- Tolerability/Acceptability assessment (Cohort 1 – Week 18 only; Cohort 2 – Week 10 only)

Clinical Procedures
- Targeted medical history and targeted physical exam, including concomitant medications and CNS symptoms assessment
- ECG (Cohort 1 - Week 23 only; Cohort 2 – Week 13 only)
- Blood collection
- ISR evaluation

Laboratory Evaluations
- HIV testing (see SSP Manual)
- CBC with differential
- Chemistry testing
- Liver function testing (AST, ALT, total bilirubin, alkaline phosphatase)
- Plasma storage

5.8 Third Injection Visit: Cohort 1 – Week 29; Cohort 2 – Week 17

Administrative, Behavioral, and Regulatory Procedures
- Locator information
- HIV counseling
- Offer condoms and lubricant
- Behavioral assessment

Clinical Procedures
- Targeted medical history and targeted physical exam, including concomitant medications and CNS symptoms assessment
- Blood collection
- Urine collection for GC/CT testing (men and women) (Cohort 1 only)
- Urine pregnancy testing for women of reproductive potential
- Rectal swab collection for GC/CT testing (men and women) (Cohort 1 only)
- Vaginal swab collection for GC/CT testing (optional alternate collection for women if urine is not used) (Cohort 1 only)
- Administer injection (with counseling)

Laboratory Evaluations
- HIV testing (see SSP Manual)
- CBC with differential
- Chemistry testing (see Appendix I or IV).
- Liver function testing (AST, ALT, total bilirubin, alkaline phosphatase)
- Syphilis testing (Cohort 1 only)
- GC/CT testing: Urine for men and either urine or vaginal swab for women. For rectal swab GC/CT testing for men and women, non-US sites will batch and ship to the HPTN Laboratory Center for testing (see SSP for details); US sites will perform testing locally. (Cohort 1 only)
- Plasma storage (must be drawn PRIOR to the injection)

NOTE: All HIV test results from previous visits and at least one HIV test result from the current visit must be available and confirmed to be negative/non-reactive PRIOR to injection of study product. The injection must not be given if any HIV test is reactive/positive.

For women of reproductive potential, a pregnancy test result from the current visit must be available and confirmed to be negative PRIOR to injection of study product. The injection must not be given if the pregnancy test is positive.

Results from the other laboratory evaluations (e.g., chemistry, LFTs, hematology) from the current visit are NOT required prior to the injections.

Results from the previous safety visit clinical and laboratory evaluations (e.g., chemistry, LFTs, hematology) must be available and be reviewed by the IoR or their designee prior to the injection.

5.9 Safety Visits: Cohort 1 – Week 30 and 35; Cohort 2 – Week 18 and 21

Administrative, Behavioral, and Regulatory Procedures
- Locator information
- HIV counseling
- Offer condoms and lubricant
- Tolerability/Acceptability assessments (Cohort 1 -Week 30 only, Cohort 2 -Week 18 only)

Clinical Procedures
- Targeted medical history and targeted physical exam, including concomitant medications and CNS symptoms assessment
- ECG (Cohort 1 - Week 35 only; Cohort 2 – Week 21 only)
- Blood collection
- ISR evaluation

Laboratory Evaluation:
- HIV testing (see SSP Manual)
- CBC with differential
- Chemistry testing
- Liver function testing (AST, ALT, total bilirubin, alkaline phosphatase)
- Plasma storage

5.10 Fourth Injection Visit: Cohort 2 only - Week 25

Administrative, Behavioral, and Regulatory Procedures
- Locator information
- HIV counseling
- Offer condoms and lubricant
- Behavioral assessment

Clinical Procedures
- Targeted medical history and targeted physical exam, including concomitant medications and CNS symptoms assessment
- Blood collection
- Urine collection for GC/CT testing (men and women)
- Urine pregnancy testing for women of reproductive potential
- Rectal swab collection for GC/CT testing (men and women)
- Vaginal swab collection for GC/CT testing (optional alternate collection for women if urine is not used)
- Administer injection (with counseling)

Laboratory Evaluations
- HIV testing (see SSP Manual)
- CBC with differential
- Chemistry testing
- Liver function testing (AST, ALT, total bilirubin, alkaline phosphatase)
- Syphilis testing

- GC/CT testing: Urine for men and either urine or vaginal swab for women. For rectal swab GC/CT testing for men and women, non-US sites will batch and ship to the HPTN Laboratory Center for testing (see SSP for details); US sites will perform testing locally.
- Plasma storage (must be drawn PRIOR to the injection)

NOTE: All HIV test results from previous visits and at least one HIV test result from the current visit must be available and confirmed to be negative/non-reactive PRIOR to injection of study product. The injection must not be given if any HIV test is reactive/positive.

For women of reproductive potential, a pregnancy test result from the current visit must be available and confirmed to be negative PRIOR to injection of study product. The injection must not be given if the pregnancy test is positive.

Results from the other laboratory evaluations (e.g., chemistry, LFTs, hematology) from the current visit are NOT required prior injection.

Results from the previous safety visit clinical and laboratory evaluations (e.g., chemistry, LFTs, hematology) must be available and be reviewed by the IoR or their designee prior to the injection.

5.11 Safety Visits: Cohort 2 only – Week 26 and 29

Administrative, Behavioral, and Regulatory Procedures

- Locator information
- HIV counseling
- Offer condoms and lubricant
- Tolerability/Acceptability assessments (Week 26 only)

Clinical Procedures

- Targeted medical history and targeted physical exam, including concomitant medications and CNS symptoms assessment
- ECG (Week 29 only)
- Blood collection
- ISR evaluation

Laboratory Evaluation:

- HIV testing (see SSP Manual)
- CBC with differential
- Chemistry testing
- Liver function testing (AST, ALT, total bilirubin, alkaline phosphatase)
- Plasma storage

5.12 Fifth Injection Visit: Cohort 2 only – Week 33

Administrative, Behavioral, and Regulatory Procedures

- Locator information
- HIV counseling
- Offer condoms and lubricant
- Behavioral assessment

Clinical Procedures

- Targeted medical history and targeted physical exam, including concomitant medications and CNS symptoms assessment
- Blood collection
- Urine pregnancy testing for women of reproductive potential
- Administer injection (with counseling)

Laboratory Evaluations

- HIV testing (see SSP Manual)
- CBC with differential
- Chemistry testing
- Liver function testing (AST, ALT, total bilirubin, alkaline phosphatase)
- Plasma storage (must be drawn PRIOR to the injection)

NOTE: All HIV test results from previous visits and at least one HIV test result from the current visit must be available and confirmed to be negative/non-reactive PRIOR to injection of study product. The injection must not be given if any HIV test is reactive/positive.

For women of reproductive potential, a pregnancy test result from the current visit must be available and confirmed to be negative PRIOR to injection of study product. The injection must not be given if the pregnancy test is positive.

Results from the other laboratory evaluations (e.g., chemistry, LFTs, hematology) from the current visit are NOT required prior injection.

Results from the previous safety visit clinical and laboratory evaluations (e.g., chemistry, LFTs, hematology) must be available and be reviewed by the IoR or their designee prior to the injection.

5.13 Safety Visits: Cohort 2 only – Weeks 34 and 37

Administrative, Behavioral, and Regulatory Procedures

- Locator information
- HIV counseling
- Offer condoms and lubricant
- Tolerability/Acceptability assessments (Week 34 only)

Clinical Procedures

- Targeted medical history and targeted physical exam, including concomitant medications and CNS symptoms assessment
- ECG (Week 37 only)
- Blood collection
- ISR evaluation

Laboratory Evaluation:

- HIV testing (see SSP Manual)
- CBC with differential
- Chemistry testing
- Liver function testing (AST, ALT, total bilirubin, alkaline phosphatase)
- Plasma storage

5.14 Primary Endpoint Visit: Both Cohorts - Week 41

Administrative, Behavioral, and Regulatory Procedures

- Locator information
- HIV counseling
- Offer condoms and lubricant
- Behavioral assessment

Clinical Procedures

- Targeted medical history and targeted physical exam, including concomitant medications and CNS symptoms assessment

- Blood collection
- Urine collection for urinalysis
- Urine pregnancy testing for women of reproductive potential

Laboratory Evaluations
- HIV testing (see SSP Manual)
- CBC with differential
- Chemistry testing
- Liver function testing (AST, ALT, total bilirubin, alkaline phosphatase)
- Fasting lipid profile (Participants should be fasting for at least 8 [preferably 12] hours prior to sample collection)
- Urinalysis (protein and glucose)
- Plasma storage

5.15 Tail Phase Visits: Both Cohorts – Week 53, 65, 77

Administrative, Behavioral, and Regulatory Procedures
- Locator information
- HIV counseling
- Offer condoms and lubricant
- Behavioral assessment

Clinical Procedures
- Targeted medical history and targeted physical exam, including concomitant medications and CNS symptoms assessment
- Blood collection
- Urine collection for GC/CT testing (men and women at week 53 only)
- Urine pregnancy testing for women of reproductive potential
- Rectal swab collection for GC/CT testing (men and women at week 53 only)
- Vaginal swab collection for GC/CT testing (optional alternate collection for women if urine is not used; Week 53 only)

Laboratory Evaluations
- HIV testing (see SSP Manual)
- Liver function testing (AST, ALT, total bilirubin, alkaline phosphatase)
- Syphilis testing (Week 53 only)
- GC/CT testing (Week 53 only): Urine for men and either urine or vaginal swab for women. For rectal swab GC/CT testing for men and women, non-US sites will batch and ship to the HPTN Laboratory Center for testing (see SSP for details); US sites will perform testing locally.
- Plasma storage

5.16 Final Visit: Cohort 1 – Week 81; Cohort 2 – Week 85

Administrative, Behavioral, and Regulatory Procedures
- HIV counseling
- Offer condoms and lubricant

Clinical Procedures
- Targeted medical history and targeted physical exam, including concomitant medications and CNS symptoms assessment
- Blood collection
- Urine pregnancy testing for women of reproductive potential

Laboratory Evaluations

- HIV testing (see SSP Manual)
- Liver function testing (AST, ALT, total bilirubin, alkaline phosphatase)
- Plasma storage

5.17 Injection Visit Windows

The visit windows for all visits, including injection visits, are outlined in the SSP Manual. In brief, the visit window for injection visits is +/- 3 days. If a participant is unable to report to the visit during this time frame, or if the participant misses their appointment within this time frame, the CMC must be contacted for consultation regarding whether rescheduling outside of the visit window is allowable.

5.18 Procedures for Continued Oral and Injectable Dosing

Appendix XI, Toxicity Management, must be referred to regarding general toxicity management, as well as specific clinical and laboratory toxicity management guidelines, including directions regarding temporary and permanent study product holds. Study product guidelines in the event of HIV infection and pregnancy are outlined in Sections 5.21 and 5.23, respectively.

5.19 Procedures for Participants Who Do Not Complete the Full Course of Injections

Participants who do not receive the full course of injections in either cohort will be followed per the relevant Schedule of Evaluations and Procedures (Appendices II and III for Cohort 1; Appendices V-VIII for Cohort 2).

Participants who are unable to receive the first injection for any reason will be terminated from the study. If the reason is due to HIV infection or pregnancy, refer to Section 5.21 and 5.23, respectively.

5.20 Planned Unblinding of Study Participants

When the last participant on the active study product arm in Cohort 2 completes his or her Week 41 visit (Cohort 2), and when key study procedures at the HPTN SDMC, LC, and LOC have been completed, both cohorts will be unblinded. Participants will be unblinded at their next study visit following final confirmation from the HPTN SDMC that all corresponding unblinding procedures have been completed. Participants in the placebo arm will no longer be required to complete any further follow-up visits once they have been unblinded. Active study product arm participants will complete their follow-up per the appropriate Schedule of Evaluations and Procedures.

As previously noted, due to differences in timing of study initiation across the participating sites, as well as the ratio of active product arm vs. placebo arm participants being enrolled, it is likely that some placebo participants will complete their full follow-up visit schedule.

Additional information will be outlined in the SSP Manual.

5.21 Participants with Suspected or Confirmed HIV Infection, at Screening, Enrollment, or Follow-Up

The CMC must be notified of any possible HIV exposures as reported by the participant during the study, as well as any reactive or positive HIV test result identified at Enrollment or follow-up.

Screening and Enrollment

HIV testing will be performed to identify participants with HIV infection, and to identify participants whose HIV results may be equivocal (which could complicate HIV diagnosis at follow-up visits). Individuals who have one or more reactive or positive HIV tests at Screening or Enrollment are not eligible to participate in this study. Furthermore, at the Screening and Enrollment (at Enrollment, prior to randomization), individuals with any signs or symptoms consistent with acute (pre-seroconversion) HIV infection will not be enrolled. Signs and symptoms consistent with acute HIV infection will be included in the SSP Manual.

Follow-up (after study Enrollment)

Frequent testing for HIV acquisition during the study period will help prevent dosing with the investigational product in a participant who may have acquired HIV infection, minimizing the risk that resistant virus will emerge. Therefore, HIV testing will be performed at all scheduled study visits. In addition, if a participant has signs or symptoms consistent with acute HIV infection (see above), or expresses a concern about recent HIV acquisition, HIV testing will be performed using an ribonucleic acid (RNA) test that, in the opinion of the Investigator of Record or designee, is able to detect early HIV infection. If possible, an assay that is US FDA-cleared for early HIV diagnosis such as the Aptima HIV-1 RNA Qualitative Assay should be used.

Regardless of whether HIV RNA testing is used for diagnostic testing, HIV acquisition after study enrollment must be confirmed in all cases using two independent samples collected on different days.

Participants who have any reactive or positive HIV test result during follow-up visits will have further testing to confirm infection, as described in the SSP Manual and Appendix IX. Samples from participants with confirmed HIV infection may be sent to a local laboratory for resistance testing to assist with clinical management; results from resistance testing performed in local laboratories will not be reported to the HPTN Statistical and Data Management Center (SDMC). The participant will not receive additional doses of study drug if they have a reactive or positive HIV test, even if further testing indicates that they do not have HIV infection.

In the event that a participant has a reactive or positive HIV test at Week 2, 4, or 5, study product will be discontinued and the participant will be followed for an additional two visits - Weeks 17 and 29 - and will be terminated from the study at the Week 29 visit. See the SSP Manual for details regarding the Week 17 and 29 visit procedures and specimens to be collected in the event of HIV infection during the oral phase.

Participants who have a reactive or positive HIV test after Week 5 (that is, have had at least one injection) will not receive additional injections and will be followed per the appropriate Schedule of Evaluations and Procedures, on study, but off study product.

For participants with confirmed HIV infection during follow-up, the treatment assignment will be provided to a subset of the CMC as outlined in the SSP Manual. This is necessary due to the limited information available regarding the study product considering it is investigative in nature, and this subset of the CMC may assist the site with the appropriate follow-up care, including HIV treatment recommendations.

Sites will have a standard operating procedure (SOP) that outlines a plan in the event that a participant becomes HIV infected during the study, to include the participant's facilitation into immediate suppressive ART treatment to prevent persistent-monotherapy-related resistance complications for a minimum of 52 weeks post-final 744LA injection. At the end of the 52 weeks, participants will be transitioned to a local HIV care clinic where ART provision could be continued (if the participant desires to stay on ART).

Administration of PEP in cases of possible HIV exposure should be expedited at the site level. Such action should be simultaneously and immediately reported to the CMC for assistance with additional clinical and behavioral management plans, as well as decisions about further administration of study medication and follow-up schedules.

5.22 Sexually Transmitted Infections

Testing for GC/ CT in men (urine) and women (urine or vaginal swab), and syphilis will occur at Screening and approximately every 6 months – see Scheduled of Procedures and Evaluations. Testing will be performed at the local laboratory. Testing for GC/ CT in men and women (rectal swab), will occur at Enrollment and approximately every 6 months – see Schedule of Procedures and Evaluations. At US sites, testing will be performed at the local laboratory. At non US sites, samples will be stored for non-real-time, batched testing at the HPTN LC or designated laboratory.

Treatment for STIs will be referred for treatment as per local guidelines. Symptomatic screening for STIs beyond what is required by the protocol will be at a site's discretion.

5.23 Pregnancy

Because this is an investigational agent, receipt of study product by female study participants of reproductive potential requires use of an effective method of contraception as outlined in Section 3.1. All participants should also use male or female condoms for prevention of HIV and other sexually transmitted infections (STIs). As needed, study staff will provide contraceptive counseling to enrolled participants throughout the duration of study participation and will facilitate access to contraceptive services through direct service delivery and/or active referrals to local service providers. Study staff also will provide participants with male and/or female condoms and lubricant and counseling on use of condoms.

Female participants of reproductive potential will have pregnancy testing performed as outlined in the Schedule of Evaluations and Procedures. Participants will be encouraged to report all signs or symptoms of pregnancy to study staff.

Any pregnancy that occurs during the course of a female participant's participation in the study should be reported to the CMC upon site awareness (either upon confirming via urine pregnancy testing during a study visit or as reported by the participant between study visits). In the event that a female participant has a positive pregnancy test at Weeks 2, 4, or 5, study product will be discontinued and the participant will be followed approximately every 12 weeks starting at the Week 17 visit until pregnancy outcome is reached. Once pregnancy outcome is reached, the participant will be terminated from the study. The CMC should be contacted regarding visit procedures and specimens to be collected at follow-up visits in the event of pregnancy during the oral phase.

Participants who have a positive pregnancy test or report a desire to become pregnant at any other time point after Week 5 (that is, have had at least one injection) will not receive additional injections (regardless of pregnancy outcome) and will be followed per the appropriate Schedule of Evaluations and Procedures, on-study but off study product.

The site IoR or designee will counsel any participants who become pregnant regarding possible risks to the fetus according to site-specific SOPs. Participants may not enroll if they are currently breastfeeding and study product should be discontinued if any participant identifies that she is breastfeeding after enrollment. The site IoR or designee also will refer the participant to

all applicable services; however, sites will not be responsible for paying for pregnancy-related care.

Participants who are pregnant at their last study visit will continue to be followed (if they agree) until the pregnancy outcome is ascertained or it is determined that the pregnancy outcome cannot be ascertained. All pregnancy outcomes will be reported on relevant Case Report Forms (CRFs). Outcomes meeting criteria for expedited adverse event (EAE) reporting also will be reported.

5.24 Changes in Reported HIV/STI Risk Status During Follow-Up

Changes in HIV and STI risk status (i.e., increase in risk) during the oral or injection phases of the study as reported by the participant upon interviewer-administered questionnaires or as deemed by the IoR upon history and physical exam, should be brought to the attention of the CMC in order to adjudicate the appropriateness of a) further dosing b) risk reduction counseling and/or c) referral for appropriate services (substance use, mental health, intimate partner violence). No further study product should be administered from the time the site is made aware of the change in risk status until the CMC has provided guidance on appropriate management.

5.25 Hepatitis B and Hepatitis C

Testing for HBV will be performed at Screening (HBsAg, HBsAb, and HBcAb). Persons with a positive HBsAg test will be excluded from the study and will be referred to their primary provider for management. Participants who do not have evidence of immunity to HBV (e.g., negative HBsAb) will be referred for HBV vaccination. For participants who do not have evidence of HBV immunity at Screening, HBV testing should be repeated at the discretion of the IoR or designee during the study if clinically indicated, if the participant has elevated AST/ALT results (elevated level at discretion of IoR or designee), or if the participant expresses a concern about having acquired HBV infection after enrollment.

HCV antibody testing will be performed at Screening. Persons with a positive HCV antibody test will be excluded from the study. Refer to the SSP Manual for persons who are isolated HBCAb positive.

5.26 Interim Contacts and Visits

Interim contacts and visits (those between regularly scheduled follow up visits) may be performed at participant request or as deemed necessary by the investigator or designee at any time during the study. All interim contacts and visits will be documented in participants' study records and on applicable CRFs.

Some interim visits may occur for administrative reasons. For example, the participant may have questions for study staff. Interim visits at which no data are collected are not documented on case report forms. Other interim contacts and visits may occur in response to AEs experienced by study participants. When interim contacts or visits are completed in response to participant reports of AEs, study staff will assess the reported event clinically, record the event on the case report form, and provide or refer the participant to appropriate medical care.

5.27 Criteria for Early Termination of Study Participation

Participants may voluntarily withdraw from the study for any reason at any time. Site IoRs may, with the approval of the CMC, withdraw participants before their scheduled termination visit to protect their safety, and/or if participants are unable or unwilling to comply with study procedures. Participants also may be withdrawn if the study sponsors, government or regulatory authorities (including the OHRP and US FDA), or site IRBs terminate the study prior to its

planned end date. Study staff will record the reason(s) for all withdrawals in participants' study records.

5.28 Pharmacokinetics

Cohort 1: Blood samples will be collected during the oral lead-in phase of the study for determination of plasma concentrations of GSK1265744 following oral dosing. Blood samples for PK analysis will also be collected starting on the first day of the first injection phase prior to the injection and every visit thereafter. At each injection visit a blood sample will be collected prior to the injections at Weeks 5, 17 and 29, and one week post-injection at Weeks 6, 18, and 30, and at week 41. The sample collected 12 weeks following each injection will serve as the pre-dose sample for the subsequent dosing interval (except for after the 3rd injection visit, i.e., the sample will be collected, but will not serve as a pre-dose sample because injections will be completed after the 3rd injection). Two additional samples will be collected 4 and 8 weeks after the first injection (Weeks 9 and 13), and one additional sample will be collected 6 weeks following the second and third injections (Weeks 23 and 35). PK samples will be collected during the oral phase at Week 2 and 4, and every 12 weeks at Weeks 35, 53, 65, 77 and 81 until participants reach 52 weeks post-third injection (these collections are modified for participants who receive less than three injections [see Appendices II and III]).

Cohort 2: Blood samples will be collected during the oral lead-in phase of the study for determination of plasma concentrations of GSK1265744 following oral dosing. Blood samples for PK analysis will also be collected starting on the first day of the first injection phase prior to the injection and every visit thereafter, as indicated in Appendices IV-VIII. At each injection visit a blood sample will be collected prior to the injections at Weeks 5, 9, 17, 25, and 33. The sample collected 4 or 8 weeks following each injection will serve as the pre-dose sample for the subsequent dosing interval (except for after the 5rh injection visit, i.e., the sample will be collected, but will not serve as a pre-dose sample because injections will be completed after the 5th injection). Two additional samples will be collected 1 and 4 weeks after each of the four injections, beginning with the Week 9 injection (Weeks 10, 13, 18, 21, 26, 29, 34, 37). Additionally, a single PK sample will be obtained 1 week after the Week 5 loading dose (Week 6). PK samples also will be collected during the oral phase at Week 2 and 4, and at follow-up phase visits (week 41, 53, 65, 77, and 85) until participants reach 52 weeks post-fifth injection (these collections are modified for participants who receive less than five injections [see Appendices V-VIII).

The actual date and time of each blood sample collection will be recorded, as well as the time of each injection. The timing of PK samples may be altered and/or PK samples may be obtained at additional time points to ensure thorough PK monitoring. Details of PK blood sample collection (including volume to be collected), processing, storage and shipping procedures are provided in the SSP Manual.

Concentrations of GSK1265744 will be determined in plasma using an approved, validated analytical methodology.

6.0 SAFETY MONITORING AND ADVERSE EVENT (AE) REPORTING

6.1 Safety Monitoring

Close cooperation between the Protocol Chair, study site Investigator(s), DAIDS Medical Officer, LOC Clinical Research Manager, SDMC Biostatistician, SDMC Clinical Affairs Staff, HPTN Laboratory Center (LC), and other study team members will be necessary in order to monitor participant safety and to respond to occurrences of toxicity in a timely manner. The team will have regularly scheduled conference calls during the period of study implementation, and additional ad hoc calls will be convened if required.

The study site Investigators are responsible for continuous close monitoring and management of AEs in conjunction with IoRs. Sites are required to have detailed SOPs describing methods for AE reporting and toxicity management to ensure that AEs are reported and managed in accordance with the protocol and for alerting the Clinical Management Committee (CMC – outlined below) if unexpected concerns arise.

A sub-group of the Protocol Team, including the Protocol Chair, DAIDS Medical Officer, site clinicians, and the SDMC Clinical Affairs Safety Associate will serve as members of the CMC. The CMC provides support to site clinicians regarding individual participant clinical management (e.g., questions related to eligibility, toxicity management, clinical holds of study drug, permanent discontinuations, etc.). In addition, for trials such as this with no DSMB oversight, the HPTN Study Monitoring Committee (SMC) may also review safety data in aggregate.

6.2 Clinical Data Review

A multi-tiered safety review process will be followed for the duration of this study. The study site investigators are responsible for the initial evaluation and reporting of safety information at the participant level, and for alerting the CMC if unexpected concerns arise.

Participant safety is also monitored by the SDMC Clinical Affairs staff (SMC reviews), who review incoming safety data for completeness and consistency on an ongoing basis. Events identified as questionable, inconsistent, or unexplained will be queried for verification.

AE reports submitted in an expedited manner to the DAIDS Safety Office will be forwarded to the DAIDS Medical Officer for review.

The SDMC will prepare routine study conduct and safety reports for the SMC, which will meet by conference call approximately every 6 months and will review safety data by study arm (unblinded) during a closed meeting. More frequent or *ad hoc* reviews of safety reports may be conducted by the SMC as needed. A recommendation to stop the trial may be made by the SMC at any such time that the team agrees an unacceptable type and/or frequency of AEs has been observed. If at any time a decision is made to discontinue the study product in all participants, DAIDS will notify the US FDA, as well as the site IoRs, who will notify the responsible IRBs expeditiously.

6.3 Adverse Event Definition and Reporting

An adverse event (AE) is defined as any untoward medical occurrence in a clinical research participant administered an investigational product and which does not necessarily have a causal relationship with the investigational product. As such, an AE can be an unfavorable or unintended sign (including an abnormal laboratory finding, for example), symptom or disease temporally associated with the use of an investigational product, whether or not considered related to the product.

Study participants will be provided a 24-hour telephone number and contact information and instructed to contact the study clinician to report any AEs they may experience. For life-threatening events, they will also be instructed to seek immediate emergency care. Where feasible and medically appropriate, participants will be encouraged to seek evaluation where the study clinician is based, and to request that the clinician be contacted upon their arrival. With appropriate permission of the participant, whenever possible, records from all non-study medical providers related to AEs will be obtained and required data elements will be recorded on study CRFs. All participants reporting an AE will be followed clinically, until the AE resolves (returns to baseline) or stabilizes.

Study site staff will document in source documents and the appropriate CRF all AEs (Grade 1 and higher) reported by or observed in enrolled study participants regardless of severity and presumed relationship to study product. AE severity will be graded per the DAIDS Table for Grading Adult and Pediatric Adverse Events, Version 2.0, November 2014. This version will be used for the entire duration of the study.

Relatedness is an assessment made by a study clinician of whether or not the event is related to the study agent. The relationship of all AE to study product will be assessed as specified in Version 2.0, January 2010 (or most current version) of the DAIDS Expedited Adverse Event (EAE) Reporting Manual.

6.4 Expedited Adverse Event Reporting

Requirements, definitions, and methods for expedited reporting of adverse events are outlined in Version 2.0 (or latest version) of the DAIDS EAE Manual, which is available on the DAIDS Regulatory Support Center (RSC) website at http://rsc.tech-res.com/safetyandpharmacovigilance.

6.4.1 Reporting to DAIDS

The DAIDS Adverse Event Reporting System (DAERS), an internet-based reporting system, must be used for EAE reporting to DAIDS. In the event of system outages or technical difficulties, EAEs may be submitted via the DAIDS EAE form. For questions about DAERS, please contact DAIDS-ES at DAIDS-ESSupport@niaid.nih.gov. Site queries may also be sent with the DAERS application itself.

Where DAERS has not been implemented, sites will submit expedited AEs by documenting the information on the current DAIDS EAE form. This form is available on the RSC website: http://rsc.tech-res.com/safetyandpharmacovigilance/. For questions about EAE reporting, please contact the RSC at DAIDSRSCSafetyOffice@tech-res.com.

6.4.2 Reporting Requirements for this Study

The Serious Adverse Event (SAE) Reporting Category, as defined in Version 2.0 of the DAIDS EAE manual, will be used for this study (the definition of an SAE is also included in the manual).

In addition to SAEs, sites will report in an expedited manner the following:

- ALT≥3xULN AND total bilirubin≥2xULN (.must be both in order to require expedited reporting)
- Any seizure event

These reporting requirements are required for each study participant from enrollment (week 0) until their follow-up in the study ends. After this time, sites must report serious, unexpected, clinical suspected adverse drug reactions if the study site becomes aware of the event on a passive basis, i.e., from publicly available information.

The study agents for the purposes of EAE reporting are GSK1265744 30 mg oral tablet or placebo and 744LA injectable suspension (200mg/mL) or placebo (also outlined in Section 4.1.).

Grading Severity of Events

The Division of AIDS Table for Grading the Severity of Adult and Pediatric Adverse Events, Version 2.0, November 2014, will be used for the entire duration of the study for determining and reporting the severity of adverse events. The DAIDS grading table is available on the DAIDS RSC website at http://rsc.tech-res.com/safetyandpharmacovigilance.

In addition to submitting EAE information to the DAIDS Safety Office via DAERS, the site investigator may be required to submit AE and EAE information to local regulatory agencies or other local authorities.

Information on Grade 1 and higher AEs will be included in reports to the US FDA, and other government and regulatory authorities as applicable. Site staff will report information regarding AEs to their IRB in accordance with all applicable regulations and local IRB requirements.

6.5 Social Impact Reporting

It is possible that participants' involvement in the study could become known to others, and that a social impact may result (i.e., because participants could be perceived as being HIV-infected or at risk or "high risk" for HIV infection). For example, participants could be treated unfairly, or could have problems being accepted by their families and/or communities. A social impact that is reported by the participant and judged by the IoR/designee to be serious or unexpected will be reported to the responsible site's IRBs at least annually, or according to their individual requirements. Social impacts will be collected and reported on CRFs during regular visits. In the event that a participant reports a social impact, every effort will be made by study staff to provide appropriate care and counseling to the participant as necessary, and/or referral to appropriate resources for the safety of the participant. Each site will provide such care and counseling in accordance with standardized guidance in the SSP Manual. While maintaining participant confidentiality, study sites may engage their CAB in exploring the social context surrounding instances of social impacts, to minimize the potential occurrence of such an impact.

7.0 STATISTICAL CONSIDERATIONS

7.1 Review of Study Design

This is a Phase 2a, randomized, multi-site, two-arm, double-blinded study of the safety, tolerability, pharmacokinetics and acceptability of 744LA. Eligible participants will be randomized 3:1, stratified by gender and region (US and non US sites), to receive GSK1265744 or matching placebo, and placed into one of two cohorts. Participants will receive daily 30 mg oral 744 tablets or matching placebo for 4 weeks, followed by a one-week washout period, to assess for safety and tolerability prior to receiving injections. Following clinical review of safety lab assessments from the oral regimen phase, participants will enter the injection phase and receive IM injections of 744LA or placebo at three time points at 12 week intervals (Cohort 1), or five time points at 4 and 8 week intervals (Cohort 2). Participants in the active drug arms will be followed for 52 weeks following their last injection. Participants in the placebo arms will be followed until 52 weeks after their last injection or until the last participant in the active study product arm of Cohort 2 completes their Week 41 visit, whichever comes first, at which time the cohorts will be unblinded to assess the primary endpoint. When all procedures related to unblinding are completed, participants in the placebo arm will no longer be followed.

Approximately 194 participants will be placed into two dose cohorts and will be randomized to one of the two study arms with an active GSK 744 to placebo ratio of 3:1, stratified by gender and region (US and non US), as follows:

Cohort 1

Arm 1: Approximately 79 participants will receive daily oral 744 (30 mg tablets) for 4 weeks, followed by a one week washout period, to establish safety and tolerability, followed by intra-muscular (IM) gluteal injections of 800 mg of 744LA (administered as two sequential 400 mg gluteal injections) at three time points at 12 week intervals as: Week 5, Week 17, and Week 29.

Arm 2: Approximately 27 participants will receive daily oral matching placebo and IM injections of vehicle-matched placebo on the same schedule as Arm 1.

Cohort 2

Arm 1: Approximately 66 participants will receive daily oral 744 (30 mg tablets) for 4 weeks, followed by a one week washout period, to establish safety and tolerability, followed by intra-muscular (IM) gluteal injections of 600 mg of 744LA (administered as one 600 mg gluteal injection) at five time points at 4 and 8 week intervals as: Week 5, Week 9, Week 17, Week 25, and Week 33.

Arm 2: Approximately 22 participants will receive daily oral matching placebo and IM injections of vehicle-matched placebo on the same schedule as Arm 1.

7.1.1 Primary Endpoint

Safety endpoint: Proportion of participants experiencing any Grade 2 or higher clinical AEs and laboratory abnormalities that occur from the initial injection to Week 41 among participants who receive at least one injection (injectable phase only)

Tolerability endpoint: Proportion of participants who receive at least 1 injection and who discontinue receiving injections prior to the full course of injections due to intolerability of injection (including but not limited to ISR), frequency of injections, burden of study procedures, or any AE.

These cohorts may be aggregated for an overall analysis if appropriate.

7.1.2 Secondary Endpoints

The following endpoints will be analyzed by cohort and in aggregate, unless otherwise noted:

- Proportion of participants who discontinue either oral or injectable study product for reasons of toxicity, tolerability, or acceptability prior to completion of the full oral and injectable phases
- Proportion of participants experiencing Grade 2 or higher clinical AEs and laboratory abnormalities during 52 weeks following final injection (safety) and any AE that leads to discontinuation (tolerability) during the aggregate oral and injectable phases
- Proportion of participants experiencing Grade 2 or higher clinical AEs and laboratory abnormalities (safety) and any AE that leads to discontinuation (tolerability) in the oral phase and washout period
- Plasma drug levels of GSK1265744 at designated time points after each injection of 744LA (by cohort only)
- Plasma drug levels of GSK1265744 at designated time points after each injection of 744LA stratified by age, gender, race, ethnicity, weight, BMI, and smoking status (by cohort only)
- Proportion of participants willing to use an injectable agent such as the study product for HIV prevention in the future
- Change from enrollment of self-reported sexual behavior (number of sexual partners, episodes of unprotected anal and/or vaginal intercourse) during the study period using a standardized assessment tool (in aggregate only)
- Number of incident HIV infections through the study period, including number with treatment emergent resistance
- Proportion of injectable hormonal-contraception-using female participants who reach a safety or tolerability endpoint as defined above

7.1.3 Exploratory Endpoints

- Relationships between observed AEs and pharmacokinetic parameters (including but not limited to GSK1265744 C_{max} and C_{min} and AUC) and participant demographic data (individual and in aggregate)
- Relationships between pharmacogenetics (evaluated by Genome Wide Association Study [GWAS], which will inform subsequent targeted analyses) and pharmacokinetic parameters or incidence of AEs (any and by organ class and individual AE)

7.2 Sample Size

It is expected that at least 168 (124 in 744LA and 44 in placebo) out of 194 participants (106 in Cohort 1 and 88 in Cohort 2) will complete the primary endpoint visit assuming that the annual retention rate is approximately 90% and the percentage of the participants who will not be advanced to the injection phase due to their elevated AST/ALT (grade ≥1) during the oral phase is 5% (at most according to ÉCLAIR data). The following power calculations are based on the sample size of 168 (124 in 744LA and 44 in placebo) for the combined two cohorts as well as the sample size of 67 in 744LA for Cohort 1 and 57 in 744LA for Cohort 2.

The goal of the safety evaluation for this study is to identify safety concerns associated with 744LA overall (combined Cohort 1 and Cohort 2) and 744LA in individual cohort (Cohort 1 or Cohort 2). The ability of the study to detect SAEs (See Section 6.3) can be expressed by the true event rate above which at least 1 SAE would likely be observed and the true event rate below

which no events would likely be observed. Specifically, for the 744LA arm of the study (n =124), there is a 90% chance of observing at least 1 event if the true rate of such an event is 1.9% or more, and there is a 90% chance of observing no events if the true rate is <0.01%. For the placebo arm (n=44), there is a 90% chance of observing at least 1 event if the true rate of such an event is 5.1% or more, and there is a 90% chance of observing no events if the true rate is 0.2% or less.

Probabilities of observing 0, 1 or more, and 2 or more events among the arms (N=168; 124 in 744LA arm, and 44 in placebo arm) are presented in Table 3 for a range of true adverse event rates. These calculations provide a more complete picture of the sensitivity of this study design to identify potential safety concerns with 744LA.

Table 3: Probability of observing 0 events, 1 or more events, and 2 or more events, among both arms in the combined cohorts: the 744LA arm (n=124) and the placebo arm (n=44), for different true event rates.

True event rate (%)	Pr(0/124)	Pr(1+/124)	Pr(2+/124)	Pr(0/44)	Pr(1+/44)	Pr(2+/44)
0.5	53.7	46.3	12.8	80.2	19.8	2.1
1	28.8	71.2	35.2	64.3	35.7	7.2
2.5	4.3	95.7	81.9	32.8	67.2	30.1
5	0.2	99.8	98.7	10.5	89.5	65.3
10	<0.1	>99.9	>99.9	1	99	94.3
15	<0.1	>99.9	>99.9	0.1	99.9	99.3
20	<0.1	>99.9	>99.9	<0.1	>99.9	99.9

For the 744LA arm in Cohort 1 (n=67), there is a 90% chance of observing at least 1 event if the true rate of such an event is 3.4% or more, and there is a 90% chance of observing no events if the true rate is 0.1% or less. For the 744LA arm in Cohort 2 (n=57), there is a 90% chance of observing at least 1 event if the true rate of such an event is 4% or more, and there is a 90% chance of observing no events if the true rate is 0.1% or less.

Probabilities of observing 0, 1 or more, and 2 or more events among the 744LA arm in two cohorts (n_1=67 and n_2=57) are presented in Table 3a for a range of true adverse event rates. These calculations provide a more complete picture of the sensitivity of this study design to identify potential safety concerns with 744LA (in 800mg or 600mg dose).

Table 3a: Probability of observing 0 events, 1 or more events, and 2 or more events, among the 744LA arm in two cohorts (n_1=67 and n_2=57)

True event rate (%)	Pr(0/67)	Pr(1+/67)	Pr(2+/67)	Pr(0/57)	Pr(1+/57)	Pr(2+/57)
0.5	71.5	28.5	4.5	75.1	24.9	3.3
1	51	49	14.5	56.4	43.6	11.1
2.5	18.3	81.7	50.2	23.6	76.4	41.9
5	3.2	96.8	85.4	5.4	94.6	78.5
10	0.1	99.9	99.3	0.2	99.8	98.2
15	<0.1	>99.9	>99.9	0	>99.9	99.9
20	<0.1	>99.9	>99.9	<0.1	>99.9	99.9

The primary safety endpoint is the proportion of participants experiencing any Grade 2 or higher clinical AEs and laboratory abnormalities that occur from the initial injection to 41 weeks among participants who receive at least one injection. The precision with which the true event rate can be estimated from the observed data depends on the underlying true event rate and the sample size. Table 4 shows the two-sided 95% confidence intervals for the true event rate based on various values for the observed event rates, given the sample size of 124 in the 744LA arm and 44 in the placebo arm in the combined cohorts. Calculations are done using the score test method.[59]

Table 4. Two-sided 95% confidence intervals based on observing a particular rate of event for the 744LA arm (n=124) and the placebo arm(n=44) in the combined cohorts

Observed Event Rate (%)	95% CI (744LA arm)	95% CI (Placebo arm)
0	(0, 3)	(0, 8)
2.5	(0.8, 6.9)	(0.4, 11.8)
5	(2.2, 10.2)	(1.3, 15.1)
10	(5.6, 16.2)	(3.6, 21.2)
15	(10, 22.7)	(7.9, 29.4)
20	(14, 28.1)	(11.2, 34.5)
25	(18.2, 33.3)	(14.6, 39.4)

Table 4a shows the two-sided 95% confidence intervals for the true event rate based on various values for the observed event rates, given the sample size of 67 in 744LA arm in Cohort 1 and 57 in 744LA arm in Cohort 2.

Table 4a. Two-sided 95% confidence intervals based on observing a particular rate of event for the 744LA arm in Cohort 1 (n=67) and Cohort 2 (n=57)

Observed Event Rate (%)	95% CI (744LA arm in Cohort 1)	95% CI (744 LA arm in Cohort 2)
0	(0, 5.4)	(0, 6.3)
2.5	(0.8, 10.2)	(0.3, 9.3)
5	(1.5, 12.4)	(1.8, 14.4)
10	(5.2, 20)	(4.9, 21.1)
15	(8.3, 25.3)	(8.5, 27.4)
20	(11.7, 30.4)	(11.1, 31.3)
25	(16.5, 36.9)	(15.2, 37.1)

The safety and toxicity rates of participants using 744LA and the rates for those using placebo will not be formally compared. A sample size of 168 participants (124 in 744LA and 44 in placebo) in the combined cohorts assuming 90% retention rate per year will assure with 83% power that a 95% confidence interval for the difference between the study drug and placebo safety and toxicity rates has an upper limit no more than 10% when the true toxicity rates for 744LA and placebo are both 5%. Table 5 displays two-sided 95% confidence interval for different assumptions on the safety and toxicity rates in the active product and placebo arms for a study of this sample size combined from the two cohorts.

Table 5: Two-sided 95% Confidence Intervals for the Difference between 744LA and Placebo Safety Rates (n=124 in the 744LA arm and n=44 in the placebo arm)

Assumed rate in placebo arm	Assumed rate in 744LA arm					
	**1%**	**5%**	**7.5%**	**10.0%**	**12.5%**	**15.0%**
**1%**	(-3.4, 3.4)	(-0.8, 8.8)	(1, 12)	(3, 15)	(5, 18)	(7.1, 20.9)
**5%**	-	(-7.5, 7.5)	(-5.4, 10.4)	(-3.3, 13.3)	(-1.2, 16.2)	(1, 19)
**7.5%**	-	-	(-9.1, 9.1)	(-6.9, 11.9)	(-4.7, 14.7)	(-2.5, 17.5)
**10.0%**	-	-	-	(-10.3, 10.3)	(-8.1, 13.1)	(-5.9, 15.9)
**12.5%**	-	-	-	-	(-11.4, 11.4)	(-9.1, 14.1)
**15.0%**	-	-	-	-	-	(-12.3, 12.3)

Tables 5a and 5b shows the two-sided 95% confidence interval for different assumptions on the safety and toxicity rates in the active product arm in each of two cohorts and the placebo arm combined from the two cohorts.

Table 5a: Two-sided 95% Confidence Intervals for the Difference of Safety Rates between the 744LA arm in Cohort 1 (n=67) and the Placebo arm (combined from the placebo arms of both cohorts) (n=44)

Assumed rate in placebo arm	Assumed rate in 744LA arm					
	**1%**	**5%**	**7.5%**	**10.0%**	**12.5%**	**15.0%**
**1%**	(-3.8, 3.8)	(-2, 10)	(-0.5, 13.5)	(1.2, 16.8)	(3.1, 19.9)	(5, 23)
**5%**	-	(-8.3, 8.3)	(-6.5, 11.5)	(-4.6, 14.6)	(-2.7, 17.7)	(-0.7, 20.7)
**7.5%**	-	-	(-10, 10)	(-8.1, 13.1)	(-6.1, 16.1)	(-4.1, 19.1)
**10.0%**	-	-	-	(-11.4, 11.4)	(-9.4, 14.4)	(-7.3, 17.3)
**12.5%**	-	-	-	-	(-12.6, 12.6)	(-10.5, 15.5)
**15.0%**	-	-	-	-	-	(-13.6, 13.6)

Table 5b Two-sided 95% Confidence Intervals for the Difference of Safety Rates between 744LA arm in Cohort 2 (n=57) and the Placebo arm (combined from the placebo arms from both cohort) (n=44)

Assumed rate in placebo arm	Assumed rate in 744LA arm					
	**1%**	**5%**	**7.5%**	**10.0%**	**12.5%**	**15.0%**
**1%**	(-3.9, 3.9)	(-2.4, 10.4)	(-0.9, 13.9)	(0.7, 17.3)	(2.4, 20.6)	(4.3, 23.7)
**5%**	-	(-8.6, 8.6)	(-6.9, 11.9)	(-5.1, 15.1)	(-3.2, 18.2)	(-1.3, 21.3)
**7.5%**	-	-	(-10.4, 10.4)	(-8.5, 13.5)	(-6.6, 16.6)	(-4.6, 19.6)
**10.0%**	-	-	-	(-11.8, 11.8)	(-9.8, 14.8)	(-7.8, 17.8)
**12.5%**	-	-	-	-	(-13, 13)	(-11, 16)
**15.0%**	-	-	-	-	-	(-14, 14)

The tolerability of 744LA will be measured by the proportion (with 95% CI) of participants who terminate from receiving injections prior to the full course due to AE, intolerability of injection, frequency of injections, or burden of procedures related to injections out of those participants that received at least one injection by treatment arm. The values of Table 4 show (in addition to

confidence intervals for safety) two-sided 95% confidence intervals for the true tolerability rate based on various possible observed rates, given the sample size of 124 participants in the 744LA arm. Table 4a similarly also shows two-sided 95% confidence intervals for true tolerability rate based on various possible observed rates, given the sample size of 67 (57) participants in the 744LA arm in Cohort 1 (Cohort 2).

The acceptability of 744LA will be measured by the proportion (with 95% CI for the 744 LA arm) of participants who would consider using 744LA for HIV prevention in the future. This endpoint will be measured at the end of the study on every enrolled participant (including those who terminated product use during the trial). Table 6 shows two-sided 95% confidence intervals for true acceptance rate based on various possible observed rates, given the sample size of 124 participants in the active 744LA arm from the two cohorts and the sample size of 67 and 57 participants in the active 744LA arm in Cohort 1 and 2, respectively.

Table 6: 95% Confidence Intervals for the True Acceptance Rate Given Possibly Observed Rates in the 744LA arm overall (n=124) and by cohort (n=67, 57)

Observed Event Rate	95% CI for True Event Rate (n=124)	95% CI for True Event Rate (n=67)	95% CI for True Event Rate (n=57)
50%	(41.3, 58.7)	(39.1, 62.3)	(36.6, 61.7)
70%	(61.6, 77.5)	(58.3, 79.8)	(57.3, 80.5)
80%	(71.9, 86)	(69.6, 88.3)	(68.7, 88.9)
90%	(83.8, 94.4)	(80, 94.8)	(78.9, 95.1)
95%	(89.8, 97.8)	(87.6, 98.5)	(85.6, 98.2)

7.3 Accrual, Follow-up, and Retention

A total of approximately 194 participants will be enrolled in approximately 16 to 24 weeks for each cohort. Study participants will receive oral tablets for 4 weeks, followed by a 1 week wash out period, followed by receipt of injections at 3 time points (Cohort 1) or 5 time points (Cohort 2) over 24 weeks. Participants in the active drug arm will be followed for 52 weeks (12 months) after their last injection. Participants in the placebo arm will be followed until 52 weeks after their last injection or until the last participant in the active study product arm completes their primary endpoint visit, whichever comes first, at which time the cohorts will be unblinded to assess the primary endpoint. From that point on, placebo participants will no longer be followed.

The protocol team will be targeting an average annual retention rate of 85-90 percent.

7.4 Random Assignment

Enrolled participants will be randomized to one of two study arms with an active GSK1265744 to placebo ratio of 3:1 in two cohorts. The arm randomization will be stratified by gender and region (US and non-US sites) and done in blocks to ensure balanced treatment assignments within each gender and region in each cohort. The randomization scheme will be generated, operationalized and maintained by the HPTN SDMC. Additional details regarding the process of randomization will be included in the SSP Manual.

7.5 Blinding

Study site staff, with the exception of the site Pharmacist of Record or their designee, and participants will be blinded to the random assignments. Blinding will be maintained until the last participant enrolled in to the active drug arm in both cohorts has completed their primary endpoint visit, and when all data related to the primary endpoint visit has been entered into the database, as well as cleaned and verified. At a specified time directed by the HPTN SDMC,

participants will be unblinded to their treatment assignment, and participants in the placebo arm will no longer need to be followed. Participants in the active study product arm will continued to be followed for 52 weeks after their last injection. In addition, as noted in Section 5.21, an Investigator can request unblinding to the CMC in the event that a participant becomes infected with HIV during the study, and the CMC will assist in the treatment of the participant's HIV infection.

NOTE: Samples may be unblinded in the HPTN Pharmacology Laboratory (only), so the relevant assays are only performed on participants who received the study product.

7.6 Data and Safety Monitoring Analysis

7.6.1 Study Monitoring Committee

Data and Safety Monitoring Board oversight is not planned for this study. The HPTN SMC will conduct interim reviews of study progress, including rates of participant accrual, visit retention, completion of primary and main secondary endpoint collection, and, in a closed report, safety data by arm. The frequency and content of SMC reviews will be determined prior to the start of the study and outlined in the SSP Manual.

7.6.2 Primary Analyses

All participants who receive at least one injection will contribute to the primary analyses. The safety and tolerability will be analyzed by cohort and in aggregate.

When the use of descriptive statistics to assess group characteristics or differences is required, the following methods will be used: for categorical variables, the number and percent in each category; for continuous variables, the mean, median, standard deviation, quartiles and range (minimum, maximum). Within-treatment group assessment of the change from the baseline measurement to a follow-up measurement will be analyzed using McNemar test (for categorical response variables) or the paired t-test or Wilcoxon signed-ranks test (for continuous variables). When use of formal testing to assess differences between users of the placebo arm and users of 744LA is required, the following methods will be used: for binomial response variables, chi-square tests and logistic regression (or exact testing methods); for continuous variables, t-tests and linear regression or nonparametric methods if data are non-normal.

To assess the adequacy of the randomization, participants in each of the two arms will be compared for baseline characteristics including demographics and laboratory measurements using descriptive statistics only.

Safety Endpoints

The primary safety analysis will include Grade 2 or higher clinical and laboratory events that occur from the initial injection to 12 weeks (Cohort 1) or 8 weeks (Cohort 2) after the last injection among participants who receive at least one injection. Secondary safety analyses will include the same definition applied over the oral phase only (week 0 to week 5) and the aggregate oral+injectable+follow-up period.

To assess safety, the number and the percent of participants experiencing each safety endpoint will be tabulated by study arm. Each participant will contribute once in each category (for example, only for the highest severity AE for each participant) for the calculation of event rates. Exact binomial confidence intervals will be calculated for each safety endpoint for each arm and Fisher's exact test used to test for differences in event rates between the two arms. No formal treatment comparisons will be performed in this study, so any p-values that are calculated should

only be used as a descriptive statistic. No formal multiple comparison adjustments will be employed for safety endpoints.

Injection Site Reaction (ISR)

The number and percentage of participants experiencing each type of injection site reaction sign or symptom will be tabulated by severity. For a given sign or symptom, each participant's ISR will be counted once under the maximum severity for all injection visits as well as by each successive injection.

AEs

AEs will be tabulated using MedDRA preferred terms. The number and percentage of participants experiencing each specific AE (All AEs, Grade 2 or higher, and SAEs) will be tabulated by severity and by relationship to study product. For the calculations in these tables, each participant's AEs will be counted once as the maximum severity and relationship to study product. AEs leading to temporarily or permanently stopping drug will also be summarized by treatment. AEs will be summarized for those that are treatment emergent during LA dosing separately from those that are treatment emergent during oral dosing and also for those that are treatment emergent across the entire treatment phase of the study (combining both LA and oral dosing).

A listing of EAEs reported to the DAIDS RSC Safety Office will provide details of the events including severity, relationship to study product, time between onset and last injection, number of injections received, and a summary of the event.

Tolerability

To assess tolerability, the number and the percent of participants who receive at least one injection and discontinue receiving injections prior to the full course due to intolerability of injection (including but limited to ISR), frequency of injections, or burden of procedures or any AE will be tabulated by study arm. Chi-square test will be used to compare the proportion of participants who terminated from receiving the full course of injections due to any of the above reasons between the two study arms.

7.6.3 Secondary Analyses

Acceptability

To assess acceptability of the 744LA, the proportion (with 95% CI for the 744LA arm) of subjects who would consider using 744LA for HIV prevention in the future will be calculated among all enrolled participants (including those who terminated product use during the trial) and summarized by treatment arm. Acceptability will be assessed by cohort and in aggregate.

A questionnaire modeled after the HIV Treatment Satisfaction Questionnaire (HIVTSQ) will also be used to assess participant acceptability and satisfaction to the treatment. For each question the responses will be summarized by the proportion of participants reporting the response on the Likert scale out of all those that answered the question by visit and treatment arm. An overall treatment satisfaction score will be calculated for each participant by visit. These total scores will be summarized by visit and treatment arm.

Sexual Risk Behaviors

Change in sexual risk behavior (number of sexual partners, episodes of unprotected anal and/or vaginal intercourse) during the injection phase will be measured by summarizing the change from baseline by visit and treatment arm. Sexual risk behavior will be analyzed in aggregate.

HIV Incidence

HIV incidence rate will be calculated as the total number of participants with confirmed HIV infection during study follow-up divided by the person-years accumulated in each arm. 95% CIs will be calculated based on Poisson distribution assumptions. HIV incidence will be assessed by cohort and in aggregate.

Pharmacokinetics

Plasma GSK1265744 concentration-time data will be analyzed by noncompartmental methods using WinNonlin Professional 5.2 or higher, Phoenix (Pharsight Corporation) or comparable software. Individual plasma PK parameters for each injection interval will be determined, including: area under the plasma concentration time curve over the dosing interval ($AUC(0\text{-}\tau)$), maximum observed concentration (C_{max}), time to maximum observed concentration (tmax), concentration at the end of the dosing interval (C_{τ}), apparent terminal phase half-life for 744LA administration ($t_{½}$) and lambda z as a measure of absorption rate constant (λ_z) if data allow. Pharmacokinetics will be analyzed by cohort only.

Descriptive statistics will be used to summarize GSK1265744 concentration by time and plasma PK parameters. Unless stated otherwise, descriptive summaries will include n, mean, standard deviation (SD), coefficient of variation (%CV), median, minimum, and maximum for continuous variables, n and percent for categorical variables, and geometric mean, 95% confidence interval (CI), and the between-subject CV (%CVb) for the $\log_e$-transformed PK parameters.

Accumulation of GSK1265744 will determined by using point estimates and confidence intervals for difference in least squares means for the comparisons of $AUC_{(0\text{-}\tau)}$, C_{max}, and C_{τ} as follows:

Table 7: Summary of Comparisons of $AUC_{(0\text{-}\tau)}$, C_{max}, and C

Comparison	Test	Reference
Accumulation	$AUC(0\text{-}\tau)$, the last injection (Week 29-41 in Cohort 1, or Week 33-41 in Cohort 2)	$AUC(0\text{-}\tau)$, the first injection (Week<br>5-17 in Cohort 1) and the second injection (Week 9-17 in Cohort 2)
	C_{max}, last injection	C_{max}, first injection
	C_{τ}, the injection (Week 41 for both cohorts)	C_{τ}, the first injection (in Cohort 1) or the second injection (in Cohort 2) (Week 17)

The difference in pharmacokinetic parameters, $AUC_{(0\text{-}\tau)}$, C_{max}, and C_{τ}, between the two different time points will be tested using paired t-test if the data or log transferred data appear to be normally distributed and using Wilcoxon signed rank test if the data or log transferred data are not normally distributed.

Time to steady state will be assessed by comparing plasma concentration at Week 41 (C_{τ} following the last injection) to previous C_{τ} concentrations at Weeks 17 and 29 in Cohort 1, or previous C_{τ} concentrations at Week 17, 25, and 33 in Cohort 2. This may be accomplished by visual inspection of graphical data or by calculating the point estimate and confidence interval for the slope for $\log(C_{\tau})$ by injection visit and cohort.

Injectable Hormonal Contraception Usage

Safety and Tolerability analyses described above will be performed on the subset of female participants who self-report the use of injectable hormonal-based contraception at study entry or at any time during the study period. Proportions of such women meeting safety or tolerability endpoints will be compared a) across study arms b) to women in the active study arm who are do not report the use of injectable hormonal contraception c) to the overall study population randomized to active GSK1265744.

7.6.4 Exploratory Analyses

Pharmacokinetics/Pharmacodynamics

The relationship between GSK1265744 PK parameters and demography (age, gender, race, ethnicity, weight, BMI, smoking status) or PD endpoints (conversion to HIV-infected status, safety parameters) may be explored.

Pharmacogenomic Analysis

Specific genes may be studied that encode the drug targets, drug mechanism of action pathways, drug metabolizing enzymes, or drug transporters or which may underpin AEs, disease risk, or pharmacokinetics or drug response. These candidate genes may include a common set of absorption, distribution, metabolism, and excretion genes that are studied to determine the relationship between gene variants and pharmacokinetics or safety parameters. In addition, future research may identify other enzymes, transporters, proteins, or receptors that may be involved in response to GSK1265744. The genes that may code for these proteins may also be studied. Results of any pharmacogenomics investigations performed will be reported either as part of the main clinical study report or as a separate report. A detailed description of any analysis elected to be performed will be documented in the study statistical analysis plan or in a separate pharmacogenomics statistical analysis plan, as appropriate.

7.6.5 Interim Analysis/Interim Meta-Analysis

An interim analysis will be performed when 50% of the participants have completed their primary endpoint visit (Week 41). All data available at that time will be included in the analysis. Pharmacokinetic data will be analyzed when approximately 50% of Cohort 1 reaches Week 29 (two troughs) for Cohort 1 participants, and when approximately 50% of Cohort 2 reaches Week 17 (two troughs) for Cohort 2. For the interim analysis, unblinded summaries from the analysis will be provided to others as pre-specified by the HPTN SMC and to the US FDA. All members of the protocol team, including GSK and ViiV Healthcare, as well as study participants will remain blinded to individual participant treatment assignment. The data from this analysis may also be combined with the interim data from a similar study being conducted by GSK, if needed and appropriate. Pooled summaries will also be provided to the study team to share with regulators. Specifics regarding study monitoring by the SMC and provision of data summaries, their review, and the estimated timing and guidance that will be used to determine regimen safety will be determined by the HPTN SMC, Protocol Chair, and ViiV Healthcare. Additional ad-hoc pharmacokinetic analyses, as required to inform regulatory interactions, may be conducted.

8.0 HUMAN SUBJECTS CONSIDERATIONS

8.1 Ethical Review

This protocol and the template informed consent form contained in Appendix XII and XIII — and any subsequent modifications — will be reviewed and approved by the HPTN Scientific Review Committee and DAIDS Prevention Science Review Committee with respect to scientific content and compliance with applicable research and human subjects regulations.

The protocol, site-specific informed consent form, participant education and recruitment materials, and other requested documents — and any subsequent modifications — also will be reviewed and approved by the ethical review bodies responsible for oversight of research conducted at the study site.

Subsequent to initial review and approval, the responsible IRBs/ECs will review the protocol at least annually. The Investigator will make safety and progress reports to the IRBs/ECs at least annually, and within three months of study termination or completion. These reports will include the total number of participants enrolled in the study, the number of participants who completed the study, all changes in the research activity, and all unanticipated problems involving risks to human subjects or others. Study sites are responsible for the submission of continuing review to the DAIDS Protocol Registration Office, in accordance with the current DAIDS Protocol Registration Policy and Procedure Manual.

8.2 Informed Consent

Written informed consent will be obtained from each study participant. Each study site is responsible for developing study informed consent forms for local use, based on the template in Appendix XII and XIII that describes the purpose of the study, the procedures to be followed, and the risks and benefits of participation, in accordance with all applicable regulations. If applicable, the study site also is responsible for translating the template form into local languages, and verifying the accuracy of the translation by performing an independent back-translation.

Participants will document their provision of informed consent by signing their informed consent forms. (Further details regarding DAIDS requirements for documenting the informed consent process can be found in the DAIDS Standard Operating Procedure for Source Documentation.)

All participants will be offered a copy of their informed consent form.

8.3 Incentives

Pending IRB approval, participants will be compensated for their time and effort in this study, and/or be reimbursed for travel to study visits and time away from work. Site-specific reimbursement amounts will be specified in the study informed consent forms.

8.4 Confidentiality

All study-related information will be stored securely at the study site. All participant information will be stored in locked file cabinets in areas with access limited to study staff. All laboratory specimens, reports, study data collection, process, and administrative forms will be identified by a coded number only to maintain participant confidentiality. All records that contain names or other personal identifiers, such as locator forms and informed consent forms, will be stored separately from study records identified by code number. All local databases will be secured with password-protected access systems. Forms, lists, logbooks, appointment books, and any other listings that link participant ID numbers to other identifying information will be stored in a separate, locked file in an area with limited access.

Participant's study information will not be released without the written permission of the participant, except as necessary for monitoring by the NIAID and/or its contractors; GSK and ViiV Healthcare; representatives of the HPTN LOC, SDMC, and/or LC; the US FDA, OHRP, other government and regulatory authorities, and/or site IRBs.

For sites located in the US, the HPTN will obtain a Certificate of Confidentiality from the US Department of Health and Human Services that will be applicable for this study. Sites may register under the Certificate through the HPTN LOC once they have obtained local IRB approvals for the study. This Certificate protects study staff from being compelled to disclose study-related information by any Federal, State or local civil, criminal, administrative, legislative, or other body.

8.5 Communicable Disease Reporting Requirements

Study staff will comply with all applicable local requirements to report communicable diseases identified among study participants to local health authorities. Participants will be made aware of all reporting requirements during the study informed consent process.

8.6 Study Discontinuation

The study also may be discontinued at any time by NIAID, the HPTN, the pharmaceutical sponsors, the US FDA, other government or regulatory authorities (OHRP), or site IRBs.

9.0 LABORATORY SPECIMENS AND BIOHAZARD CONTAINMENT

Laboratory procedures are described below, Appendices I-VIII, and Section 5.0; additional tests to be performed at a subsequent visit for participants who have a reactive or positive HIV test result are described in Appendix IX.

9.1 Local Laboratory Specimens

The following types of tests will be performed at the local laboratory:

- HIV testing (see SSP Manual)
- HBV and HCV testing to include hepatitis B surface antigen (HBsAg), hepatitis B surface antibody (HBsAb), hepatitis B core antibody (HBcAb) and hepatitis C antibody (HCV) tests
- CBC with differential and platelets
- Chemistry testing (BUN/urea, Na, K, Cl, CO_2, CPK, creatinine, total protein, glucose, calcium, phosphorous, amylase, lipase, magnesium)
- LFTs (AST, ALT, total and direct bilirubin, alkaline phosphatase)
- Fasting lipid profile (total cholesterol, HDL, triglycerides, LDL – calculated or measured (participants should be fasting for at least 8 [preferably 12] hours prior to sample collection)
- Syphilis serologic testing (men and women)
- Urine for GC/CT testing for men and women. (may be replaced with a vaginal swab in women if desired)
- Urinalysis (protein and glucose)
- Urine test for pregnancy*
- Rectal swabs for GC/CT for men and women (for non-US sites, batch and ship to the HPTN LC; for US sites, testing will be performed locally)
- Plasma storage
- Plasma storage for Pharmacology testing
- Sample storage for Pharmacogenomic testing (sites may choose to opt out of this testing if is not acceptable to local regulatory bodies or for other reasons approved by the protocol team)
- Following a positive or reactive HIV test result: HIV viral load, CD4 cell count, HIV resistance testing at a local laboratory (real-time resistance testing for clinical management is optional, at the site's discretion), plasma storage.

If the HIV testing algorithm includes HIV rapid testing, that testing may be performed in the clinic or laboratory. Urine pregnancy testing may be performed in the clinic or the laboratory.

*All women of reproductive potential will have a urine βHCG test for pregnancy (sensitivity of $\leq$ 25 mIU/mL) at each visit. Pregnancy testing is not required at subsequent visits if a woman had a positive pregnancy test at a previous visit and is still pregnant.

Each study site will adhere to standards of good laboratory practice, the HPTN LC Manual, the SSP and local SOPs for proper collection, processing, labeling, transport, and storage of specimens to the local laboratory. Specimen collection, testing, and storage at the local laboratory will be documented using the HPTN Laboratory Data Management System (LDMS) as described in the SSP Manual.

All specimens will be shipped in accordance with International Air Transport Association (IATA) specimen shipping regulations. All shipments will be documented using the HPTN LDMS as described in the SSP Manual.

9.2 Stored Specimens

As described in Section 5.0, and indicated in Appendices I-VIII, plasma will be stored at the local site at every visit. In addition, blood samples will be stored at the Enrollment visit for participants who consent to Pharmacogenomic testing. A subset of the stored samples will be shipped to the HPTN LC (located in the US) for Quality Assurance (QA) and other assessments. As indicated below, testing on stored samples will be performed by the HPTN LC or another US laboratory designated by the HPTN LC. Rectal swab samples may also be shipped to the HPTN LC for testing. The HPTN LC may ask sites to store other specimen types for STI testing at the HPTN LC if the site is not able to perform the testing.

Virology

The HPTN LC will perform QA testing, including testing to determine HIV infection status in selected cases. Additional assays may be performed at the HPTN LC or a laboratory designated by the HPTN LC. This testing may include the following tests for participants who acquire HIV infection: HIV viral load, HIV resistance testing, HIV subtyping, and other tests to characterize HIV viruses and/or the host response to HIV infection. Results will not be returned to the sites or study participants, with the exception of HIV testing (if results obtained at the HPTN LC do not agree with site results) and the exception for resistance test results, noted below.

Resistance testing will be performed at the HPTN LC or a laboratory designated by the HPTN LC. This testing will be performed retrospectively at the end of the study. If real-time resistance testing is needed for clinical management, that testing should be arranged by the site outside of the study; separate specimens should be collected for that testing. For sites that do not have the capacity for local resistance testing for clinical care, results from resistance testing may be provided at the end of the study at the request of the site IoR, with approval of the HPTN LC and Protocol Chair. Results from specialized resistance testing (e.g., minority variants analysis, if performed) will not be returned to study sites.

Pharmacology

Plasma samples for drug levels will be collected beginning at Week 2 through and including the last study visit at Week 81 (Cohort 1) and Week 85 (Cohort 2). These samples will collected from all participants, although PK testing may be limited to a subset of the samples.

Plasma will be processed and frozen locally for subsequent shipment to the HPTN LC following procedures outlined in the SSP Manual. Pharmacology testing will be performed at the HPTN LC or at an outside laboratory designated by the HPTN LC. The primary pharmacologic assessments will be performed using assays that have been validated and approved by the Clinical Pharmacology Quality Assurance (CPQA) Committee. Results will not be returned to the sites or study participants.

NOTE: Samples may be unblinded in the HPTN Pharmacology Laboratory (only), so the relevant assays are only performed on participants who received the study product.

Stored plasma may also be tested for the presence of other ARV drugs or other substances.

Pharmacogenomics

Blood samples collected for Pharmacogenomic testing will be analyzed for genetic polymorphisms associated with study drug exposure. Assays will be performed at the HPTN LC. Sites may choose to opt out of this testing. For sites that do allow this testing, results will not be returned to the sites or study participants.

GC/CT Rectal Swab Storage (Non US Sites Only)

Testing for GC/CT in men and women (rectal swab), will occur at scheduled visits approximately every 6 months. At US sites, testing will be performed at the local laboratory. At non-US sites, samples will be stored for non-real-time, batched testing at the HPTN LC or designated laboratory.

9.3 Quality Control and Quality Assurance Procedures

Study sites will document that their laboratories are certified under the Continuous Laboratory Improvement Act of 1988 (CLIA-certified) and/or participate in DAIDS-sponsored External Quality Assurance (EQA) programs, or other QA methods as deemed appropriate by the HPTN LC. HPTN LC staff will conduct periodic visits to each site to assess the implementation of on-site laboratory quality control (QC) procedures, including proper maintenance of laboratory testing equipment and use of appropriate reagents. HPTN LC staff will follow up directly with site staff to resolve any QC or QA problems identified through proficiency testing and/or on-site assessments.

9.3.1 QC for HIV Diagnostic Testing

Before performing HIV diagnostic testing, all sites must validate their HIV testing procedures, and the validation studies must be approved by the HPTN LC. Local laboratories will perform testing for HIV diagnosis at screening, enrollment, and all follow-up visits. Algorithms for HIV diagnostic testing are provided in the SSP Manual.

Throughout the course of the study, the HPTN LC will select a random sample of stored specimens to test for QA purposes. The total number of specimens undergoing QA testing will be determined by the HPTN LC.

The HPTN LC will inform site staff of the samples selected for QA testing, and site staff will ship the selected specimens to the HPTN LC. The HPTN LC will test the specimens for evidence of HIV infection and compare the results of their tests with the results obtained by the local labs. HPTN LC staff will follow-up directly with site staff to resolve any QA problems identified through this process.

9.3.2 Quality Assurance for General Laboratory Testing

Local laboratories will perform chemistry, LFT, lipid profile, hematology, urinalysis and pregnancy testing as indicated in Appendices I-VIII. Non-US laboratories performing these tests must demonstrate successful participation in the relevant EQA programs as deemed appropriate by the HPTN LC. US sites should send these tests to CLIA-certified laboratories and must participate in EQA programs.

9.3.3 Quality Assurance for CD4 Cell Count Testing

Local laboratories may also perform CD4 cell count testing as indicated in Appendix IX. Non-US laboratories performing these tests will be monitored by the DAIDS Immunology Quality Assurance (IQA) program and United Kingdom National External Quality Assessment Service (UKNEQAS) program and must demonstrate successful participation in these programs. US sites must use CLIA-certified laboratories; participation in the IQA program is recommended.

9.3.4 Quality Assurance for HIV RNA Testing

Local laboratories may also perform HIV RNA/viral load testing as indicated in Appendix IX or for evaluation of possible acute HIV infection. Non-US laboratories performing these tests will

be monitored by the DAIDS Virology Quality Assurance (VQA) program and must demonstrate successful participation in this program. Alternately HIV RNA/viral load testing for clinical purposes can be done per local standard of care. US sites must use CLIA-certified laboratories; participation in the VQA program is recommended. For participants who become HIV infected, HIV RNA/viral load analysis will be performed retrospectively at the HPTN LC; results from this testing will not be returned to study sites or participants.

9.4 Specimen Storage and Possible Future Research Testing

Study sites will store specimens collected in this study at least through the end of the study. In addition, at sites that allow this type of storage, study participants will be asked to provide written informed consent for these samples to be stored after the end of the study for possible future non-protocol listed testing. The specimens of participants who do not consent to long-term storage and additional testing will be destroyed after all of the protocol specified testing (including assessments at the HPTN LC) has been completed and the primary research paper has been published.

9.5 Biohazard Containment

As the transmission of HIV and other blood-borne pathogens can occur through contact with contaminated needles, blood, and blood products, appropriate blood and secretion precautions will be employed by all personnel in the drawing of blood and shipping and handling of all specimens for this study, as currently recommended by the US CDC. All infectious specimens will be transported in accordance with United States regulations (42 CFR 72).

10.0 ADMINISTRATIVE PROCEDURES

10.1 Protocol Registration

Initial Registration of the protocol by the DAIDS Protocol Registration Office (PRO) is required prior to implementation of this protocol. As part of this process, each site must have the protocol and protocol informed consent form(s) approved, as appropriate, by their local institutional review board (IRB)/ethics committee (EC) and any other applicable regulatory entity (RE). Upon receiving final approval, sites will submit all required protocol registration documents to the DAIDS PRO at the Regulatory Support Center (RSC). The DAIDS PRO will review the submitted protocol registration packet to ensure that all of the required documents have been received. In the case of Initial Registration, site-specific informed consent forms (ICFs) *WILL* be reviewed and approved by the DAIDS PRO. Sites will receive an Initial Registration Notification from the DAIDS PRO that indicates successful completion of the protocol registration process. A copy of the Initial Registration Notification should be retained in the site's regulatory files.

Following Initial Registration, any full protocol amendments require submission of a protocol registration packet to the DAIDS PRO as described above; however, the DAIDS PRO *WILL NOT* review and approve site-specific ICFs. Upon receiving final IRB/EC and any other applicable RE approval(s) for an amendment, sites should implement the amendment immediately. Sites will receive a Registration Notification when the DAIDS PRO receives a complete registration packet.

There may be cases where a site submits as their Initial Registration a protocol amendment (Version 2.0 or higher of a protocol); in such cases, the instructions for Initial Registration will be followed.

For additional information on the protocol registration process and specific documents required for initial and amendment registrations, refer to the current version of the DAIDS Protocol Registration Manual, which can be found at http://rsc.tech-res.com/protocolregistration/.

10.2 Study Activation

Pending successful protocol registration and submission of all required documents, the HPTN LOC staff will "activate" a site. Study implementation may not be initiated until a study activation notice is provided to the site by the HPTN LOC. (In some cases the Division of AIDS has provided activation approval via email, which is also acceptable documentation of activation.) In addition, if study "activation" is determined to be necessary for any subsequent amendments, study implementation may not be initiated until a study activation notice is provided to the site by the HPTN LOC.

10.3 Study Coordination

DAIDS holds the Investigational New Drug (IND) application for this study. Copies of all regulatory documents submitted to this IND by DAIDS will be forwarded to GlaxoSmithKline and ViiV Healthcare for cross-referencing with the company's other INDs for the study product(s). Assignment of all sponsor responsibilities for this study will be specified in a Clinical Trials Agreement executed by DAIDS and ViiV Healthcare.

Study implementation will be directed by this protocol as well as the SSP Manual. The SSP Manual, which will include links to the DAIDS SOPs for Source Documentation and Essential Documents, as well as links to the Manual for Expedited Reporting of Adverse Events to DAIDS and the DAIDS Toxicity Tables, will outline procedures for conducting study visits; data and forms processing; AE assessment, management and reporting; dispensing study products and documenting product accountability; and other study operations.

Study case report forms will be developed by the study team and HPTN SDMC. Data will be transferred to the HPTN SDMC, entered, and cleaned using the HPTN SDMC DataFax data management system. Quality control reports and queries routinely will be generated and distributed to the study sites for verification and resolution.

Close coordination between protocol team members will be necessary to track study progress, respond to queries about proper study implementation, and address other issues in a timely manner. Rates of accrual, adherence, follow-up, and AE incidence will be monitored closely by the team as well as the HPTN Study Monitoring Committee. The CMC (as described in Section 6.1) will address issues related to study eligibility and AE management and reporting as needed to assure consistent case management, documentation, and information-sharing across sites.

10.4 Study Monitoring

On-site study monitoring will be performed in accordance with DAIDS policies. Study monitors will visit the site to:

- verify compliance with human subjects and other research regulations and guidelines;
- assess adherence to the study protocol, study-specific procedures manual, and local counseling practices; and
- confirm the quality and accuracy of information collected at the study site and entered into the study database.

Site investigators will allow study monitors to inspect study facilities and documentation (e.g., informed consent forms, clinic and laboratory records, other source documents, case report forms), as well as observe the performance of study procedures. Investigators also will allow inspection of all study-related documentation by authorized representatives of the HPTN LOC, HPTN SDMC, HPTN LC, NIAID, ViiV Healthcare, site IRBs/ECs, and US regulatory authorities (OHRP and US FDA). A site visit log will be maintained at each study site to document all visits.

10.5 Protocol Compliance

The study will be conducted in full compliance with the protocol. The protocol will not be amended without prior written approval by the Protocol Chair and DAIDS Medical Officer. All protocol amendments must be submitted to and approved by the relevant IRB(s) and the DAIDS Regulatory Support Center (RSC) prior to implementing the amendment.

10.6 Investigator's Records

The Investigator will maintain, and store in a secure manner, complete, accurate, and current study records throughout the study. In accordance with Federal regulations, for each of the investigational products tested, the Investigator will retain all study records for at least two years following the date of marketing approval for the study product for the indication in which it was studied. If no marketing application is filed, or if the application is not approved, the records must be retained for two years after the US FDA is notified that the IND is discontinued. Study records include administrative documentation — including protocol registration documents and all reports and correspondence relating to the study — as well as documentation related to each participant screened and/or enrolled in the study — including informed consent forms, locator forms, case report forms, notations of all contacts with the participant, and all other source documents.

10.7 Use of Information and Publications

Publication of the results of this study will be governed by HPTN policies. Any presentation, abstract, or manuscript will be submitted by the Investigator to the HPTN Manuscript Review Committee, DAIDS, and ViiV Healthcare, for review prior to submission.

11.0 REFERENCES

1. Fauci AS, Folkers GK, Dieffenbach CW. HIV-AIDS: much accomplished, much to do. Nat Immunol. 2013;14(11):1104-7.
2. Grant RM, Lama JR, Anderson PL, McMahan V, Liu AY, Vargas L, et al. Preexposure Chemoprophylaxis for HIV Prevention in Men Who Have Sex with Men. The New England Journal of Medicine. 2010;363(27):2587-99.
3. Baeten JM, Donnell D, Ndase P, Mugo NR, Campbell JD, Wangisi J, et al. Antiretroviral Prophylaxis for HIV Prevention in Heterosexual Men and Women. New England Journal of Medicine. 2012;367(5):399-410.
4. Thigpen MC. Daily oral antiretroviral use for the prevention of HIV infection in heterosexually active young adults in Botswana: results from the TDF2 study. 6th IAS, Abstract WELBC01, 2011.
5. Gulick RM, Mellors JW, Havlir D, Eron JJ, Gonzalez C, McMahon D, et al. Treatment with indinavir, zidovudine, and lamivudine in adults with human immunodeficiency virus infection and prior antiretroviral therapy. N Engl J Med. 1997;337(11):734-9.
6. Gulick RM, Ribaudo HJ, Shikuma CM, Lustgarten S, Squires KE, Meyer WA, 3rd, et al. Triple-nucleoside regimens versus efavirenz-containing regimens for the initial treatment of HIV-1 infection. N Engl J Med. 2004;350(18):1850-61.
7. Riddler SA, Haubrich R, DiRienzo AG, Peeples L, Powderly WG, Klingman KL, et al. Class-sparing regimens for initial treatment of HIV-1 infection. N Engl J Med. 2008;358(20):2095-106.
8. Daar ES, Tierney C, Fischl MA, Sax PE, Mollan K, Budhathoki C, et al. Atazanavir plus ritonavir or efavirenz as part of a 3-drug regimen for initial treatment of HIV-1. Ann Intern Med. 2011;154(7):445-56.
9. van der Straten A, Van Damme L, Haberer JE, Bangsberg DR. Unraveling the divergent results of pre-exposure prophylaxis trials for HIV prevention. AIDS. 2012;26(7):F13-9.
10. Amico KR, Mansoor LE, Corneli A, Torjesen K, van der Straten A. Adherence support approaches in biomedical HIV prevention trials: experiences, insights and future directions from four multisite prevention trials. AIDS Behav. 2013;17(6):2143-55.
11. Woodsong C, MacQueen K, Amico KR, Friedland B, Gafos M, Mansoor L, et al. Microbicide clinical trial adherence: insights for introduction. J Int AIDS Soc. 2013;16:18505.
12. Wainberg MA, Friedland G. Public health implications of antiretroviral therapy and HIV drug resistance. JAMA. 1998;279(24):1977-83.
13. Carrieri MP, Chesney MA, Spire B, Loundou A, Sobel A, Lepeu G, et al. Failure to maintain adherence to HAART in a cohort of French HIV-positive injecting drug users. Int J Behav Med. 2003;10(1):1-14.
14. Bassetti S, Battegay M, Furrer H, Rickenbach M, Flepp M, Kaiser L, et al. Why is highly active antiretroviral therapy (HAART) not prescribed or discontinued? Swiss HIV Cohort Study. J Acquir Immune Defic Syndr. 1999;21(2):114-9.
15. Hogg RS, Heath K, Bangsberg D, Yip B, Press N, O'Shaughnessy MV, et al. Intermittent use of triple-combination therapy is predictive of mortality at baseline and after 1 year of follow-up. AIDS. 2002;16(7):1051-8
16. Spreen WR, Margolis DA, Pottage JC, Jr. Long-acting injectable antiretrovirals for HIV treatment and prevention. Curr Opin HIV AIDS. 2013;8(6):565-71.
17. World Health Organization (WHO) Fact Sheet, Updated October 2013 http://www.who.int/mediacentre/factsheets/fs360/en/index.html
18. Interim Guidance for Clinicians Considering the Use of Preexposure Prophylaxis for the Prevention of HIV Infection in Heterosexually Active Adults. *MMWR. Morbidity and mortality weekly report.* Aug 10 2012;61:586-589.

19. World Health Organization (WHO). Guidance on oral pre-exposure prophylaxis (PrEP) for serodiscordant couples, men and transgender women who have sex with men at high risk of HIV. 2012; http://www.who.int/hiv/pub/guidance_prep/en/index.html. Accessed Aug 14, 2012.

20. Gallant JE, DeJesus E, Arribas JR, Pozniak AL, Gazzard B, Campo RE, et al. Tenofovir DF, emtricitabine, and efavirenz vs. zidovudine, lamivudine, and efavirenz for HIV. N Engl J Med. 2006;354(3):251–60.

21. Sax PE et al. Co-Formulated Elvitegravir, Cobicistat, Emtricitabine, and Tenofovir Versus Co-Formulated Efavirenz, Emtricitabine, and Tenofovir for Initial Treatment of HIV-1 Infection: A Randomised, Double-Blind, Phase 3 Trial, Analysis of Results After 48 Weeks. Lancet 379, no. 9835 (June 30, 2012): 2439-2448, doi:10.1016/S0140-6736(12)60917-9.

22. Sax PE et al. Abacavir/lamivudine versus tenofovir DF/emtricitabine as part of combination regimens for initial treatment of HIV: Final results. J Infec Dis 2011.

23. Lennox JL, DeJesus E, Lazzarin A, Pollard RB, Madruga JV, Berger DS, et al. Safety and efficacy of raltegravir-based versus efavirenz-based combination therapy in treatment-naive patients with HIV-1 infection: a multicentre, double-blind randomised controlled trial. Lancet. 2009 ed. 2009 Sep 5;374(9692):796–806.

24. Molina JM, Molina J-M, Cahn P, et al. Rilpivirine versus efavirenz with tenofovir and emtricitabine in treatment-naive adults infected with HIV-1 (ECHO): a phase 3 randomised double-blind active-controlled trial. The Lancet 2011;378(9787):238–46.

25. Subbarao S, Otten RA, Ramos A, Kim C, Jackson E, Monsour M, et al. Chemoprophylaxis with tenofovir disoproxil fumarate provided partial protection against infection with simian human immunodeficiency virus in macaques given multiple virus challenges. J INFECT DIS. Oxford University Press; 2006 Oct 1;194(7):904–11.

26. Garcia-Lerma JG, Cong ME, Mitchell J, Youngpairoj AS, Zheng Q, Masciotra S, et al. Intermittent prophylaxis with oral truvada protects macaques from rectal SHIV infection. Science Translational Medicine. 2010 ed. American Association for the Advancement of Science; 2010 Jan 13;2(14):14ra4–14ra4.

27. Baeten JM, Donnell D, Ndase P, et al. Antiretroviral prophylaxis for HIV prevention in heterosexual men and women. *The New England journal of medicine.* Aug 2 2012;367(5):399-410. PMCID: Available free on-line.

28. Van Damme L, Corneli A, Ahmed K, et al. Preexposure prophylaxis for HIV infection among African women. *The New England journal of medicine.* Aug 2 2012;367(5):411-422.

29. Microbicide Trials Network (MTN). Microbicide Trials Network statement on decision to discontinue use of tenofovir gel in VOICE, a major HIV prevention study in women. 2011; http://www.mtnstopshiv.org/sites/default/files/attachments/MTNStatementNov17DSMB_final.pdf. Accessed Aug 30, 2012.

30. Marrazzo J, et al (2013). "Pre-exposure Prophylaxis for HIV in Women : Daily Oral Tenofovir, Oral tenofovir/Emtricitabine, or Vaginal Tenofovir Gel in the VOICE Study (MTN-003)." 20th Conference on Retroviruses and Opportunistic Infections; Atlanta, GA; 2013..

31. Haberer JE, Kahane J, Kigozi I, et al. Real-time adherence monitoring for HIV antiretroviral therapy. *AIDS Behav.* Dec 2010;14(6):1340-1346.

32. Kashuba AD, Patterson KB, Dumond JB, Cohen MS. Pre-exposure prophylaxis for HIV prevention: how to predict success. *Lancet.* Jun 30 2012;379(9835):2409-2411. PMCID: No federal funding (review).

33. Lui A et al. Bone Mineral Density in HIV-Negative Men Participating in a Tenofovir Pre-Exposure Prophylaxis Randomized Clinical Trial in San Francisco. PLoS ONE 2011;6(8):e23688.

34. Stellbrink H-J, Orkin C, Arribas JR, et al. Comparison of changes in bone density and turnover with abacavir-lamivudine versus tenofovir-emtricitabine in HIV-infected adults: 48-week results from the ASSERT study. Clinical Infectious Diseases 2010;51(8):963–72.

35. Hall AM, Hendry BM, Nitsch D, Connolly JO. HallAJKD2011. YAJKD 2011;57(5):773–80.

36. Patterson KB, Prince HA, Kraft E, et al. Penetration of tenofovir and emtricitabine in mucosal tissues: implications for prevention of HIV-1 transmission. *Sci Transl Med.* Dec 7 2011;3(112):112re114.

37. AIDS Info: DHHS guidelines: ref is Panel on Antiretroviral Guidelines for Adults and Adolescents. Guidelines for the use of antiretroviral agents in HIV-1-infected adults and adolescents. Department of Health and Human Services. Available at http://aidsinfo.nih.gov/contentfiles/lvguidelines/AdultandAdolescentGL.pdf.)

38. Reece M, Herbenick D, Schick V, Sanders SA, Dodge B, Fortenberry JD. Condom Use Rates in a National Probability Sample of Males and Females Ages 14 to 94 in the United States. The Journal of Sexual Medicine 2010;7:266–76.

39. GlaxoSmithKline Document Number RH2009/00003/03: GSK1265744 Investigator's Brochure. Effective Date 03 September 2013.

40. Andrews C, Gettie A, Russell-Lodrigue K, et al. Long-acting parenteral formulation of GSK1265744 protects macaques against repeated intrarectal challenges with SHIV [Abstract no. 24LB]; 20th Conference on Retroviruses and Opportunistic Infections; Atlanta, GA; 2013.

41. Andrews C, Spreen W, Yueh YL, Gettie A, Russell-Lodrigue Kasi Russell-Lodrigue3, Mohri H, Cheng-Mayer C, Hong Z, Markowitz M, Ho D. Correlating GSK1265744 Plasma Levels To Prevention of Rectal SHIV Transmission in Macaques. 21st Conference on Retrovirus and Opportunistic Infections, Abstract 39, March 2014, Boston, MA.

42. Andrews CD, Spreen WR, Mohri H, Moss L, Ford S, Gettie A, Russell-Lodrigue K, Bohm RP, Cheng-Mayer C, Hong Z, Markowitz M, Ho DD. Long-acting integrase inhibitor protects macaques from intrarectal simian/human immunodeficiency virus. Science. 2014 Mar 7;343(6175):1151-4)

43. Radzio J, Spreen W, Yueh YL, Mitchell J, Jenkins L, Garcia-Lerma JG, Heneine W. Monthly GSK744 Long-Acting Injections Protect Macaques Against Repeated Vaginal SHIV Exposures. 21st Conference on Retrovirus and Opportunistic Infections, Abstract 39, March 2014, Boston, MA.

44. Min S, DeJesus E, McCurdy L, et al. Pharmacokinetics (PK) and safety in healthy and HIV-infected subjects and short-term antiviral efficacy of S/GSK1265744, a next generation once daily HIV integrase inhibitor [Abstract no. H-1228]; 49th Interscience Conference on Antimicrobial Agents and Chemotherapy; San Francisco, CA; 2009.

45. Spreen W, Ford S, Chen S, et al. Pharmacokinetics, safety and tolerability of the HIV integrase inhibitor S/GSK1265744 long acting parenteral nanosuspension following single dose administration to healthy adults [Abstract no. TUPE040]; 19th International AIDS Conference; Washington, DC; 2012.

46. M Lou Y, Gould E, Chen S, Wilfret D, Spreen W, Piscitelli S, Margolis D. Meta-Analysis of Safety Data from 8 Clinical Studies with GSK1265744, an HIV Integrase Inhibitor, Dosed Orally or as Injection of Long-Acting Parenteral Nanosuspension. Poster Presentation #1752. 53rd ICAAC, Denver, USA, September 2013.

47. Margolis D, Bhatti B, Smith G, Weinberg W et al. Once-Daily Oral GSK1265744 (GSK744) As Part Of Combination Therapy in Antiretroviral Naïve Adults: 24-Week Safety and Efficacy Results from the LATTE Study (LAI116482). 14th European Aids Conference; Brussels, Belgium; 2013.

48. Margolis D, Brinson C, Eron J, Richmond G, LeBlanc R, Griffith S, St. Clair M, Stevens M, Williams P, Spreen W. 744 and Rilpivirine as Two-Drug Oral Maintenance Therapy: LAI116482 (LATTE) Week 48 Results. 21st Conference on Retrovirus and Opportunistic Infections, Abstract 91LB, March 2014, Boston, MA.

49. Coggins C, Elias CJ, Atisook R, et al. Women's preferences regarding the formulation of over-the-counter vaginal spermicides. *AIDS.* Jul 30 1998;12(11):1389-1391.

50. Severy LJ, Newcomer S. Critical Issues in Contraceptive and STI Acceptability Research. *Journal of Social Issues.* 2005;61(1):45-65.

51. Morrow KM, Tolley EE, Carballo-Dieguez A. Topical Microbicide Acceptability. *Sexually Transmitted Diseases*. 2nd ed 2013

52. Woodcock A, et al. Validation of the HIV treatment satisfaction questionnaire (HIVTSQ). Qual Life Res. 2001;10(6):517-31.

53. Woodcock A, et al. Validation of the revised 10-item HIV Treatment Satisfaction Questionnaire status version and new change version. Value Health 9: 320–333. doi: 10.1111/j.1524-4733.2006.00121.x

54. Malotte CK. Brief Risk-Reduction Counseling in Clinical Settings for HIV Pre-Exposure Prophylaxis. American Journal of Preventive Medicine 2013;44(1, Supplement 2):S112–8.

55. Mugwanya KK,et al. Sexual behaviour of heterosexual men and women receiving antiretroviral pre-exposure prophylaxis for HIV prevention: a longitudinal analysis. Lancet Infect Dis. 2013 Dec;13(12):1021-8. doi: 10.1016/S1473-3099(13)70226-3. Epub 2013 Oct 17.

56. Abbas UL, Anderson RM, Mellors JW. Potential Impact of Antiretroviral Chemoprophylaxis on HIV-1 Transmission in Resource-Limited Settings. PLoS ONE 2007;2(9):e875.

57. HPTN Ethics Guidance Document, 23-24). http://www.hptn.org/web%20documents/EWG/HPTNEthicsGuidanceV10Jun2009.pdf

58. Millum J, et al. The ethics of placebo-controlled trials: Methodological justifications. Contemp Clin Trials. 2013 Nov;36(2):510-4. doi: 10.1016/j.cct.2013.09.003. Epub 2013 Sep 12.

59. Agresti A, Coull BA. Approximate is better than "exact" for interval estimation of binomial proportions. Am Stat 1998;52:119-26).

APPENDICES I-XIII

Appendix I: Schedule of Procedures and Evaluations – For Participants Who Complete All Three Injections in Cohort 1

		Oral Phase				Injection And Tail Phase Follow-up								
	Screen	Day 0 Enr	Week 2 Safety	Week 4 Post Oral Drug	1-WEEK WASHOUT	Week 5 First Injection	Week 6, 9, 13 Safety	Week 17 Second Injection	Week 18, 23 Safety	Week 29 Third Injection	Week 30, 35 Safety	Week 41 Primary Endpoint	Week 53, 65, 77 Tail Phase	Week 81 Final Visit
**ADMINISTRATIVE, BEHAVIORAL, REGULATORY**														
**Informed consent**	X													
**Locator information**	X	X	X	X		X	X	X	X	X	X	X	X	
**Demographic information**		X												
**Randomization**		X												
**HIV counseling**	X	X	X	X		X	X	X	X	X	X	X	X	X
**Offer condoms and lubricant**	X	X	X	X		X	X	X	X	X	X	X	X	X
**Acceptability assessments (Weeks 0, 6, 18, 30 only)[15]**		X					X		X		X			
**Behavioral assessment**		X				X		X		X		X	X	
**Adherence counseling/pill count[1]**		X	X	X										
**AUDIT questionnaire**	X													
**CLINICAL EVALUATIONS & PROCEDURES**														
**History (including bleeding history at Screening), con meds, physical exam[2]**	X	X	X	X		X	X	X	X	X	X	X	X	X
**CNS symptom assessment**			X	X		X	X	X	X	X	X	X	X	X
**ECG[3]**	X	X		X			X		X		X			
**Blood collection**	X	X	X	X		X	X	X	X	X	X	X	X	X
**Urine collection for GC/CT testing men and women[5]**	X									X			X	
**Urine collection for urinalysis**		X										X		
**Urine pregnancy testing[4]**	X	X	X	X		X		X		X		X	X	X
**Rectal swab for GC/CT testing men and women[5]**		X								X			X	
**Vaginal swab for GC/CT testing[5] (Optional alternate collection for women if urine not used)**	X									X			X	
**Provide oral study drug**		X												
**Administer injection[6]**						X		X		X				
**ISR evaluation**							X		X		X			
**LOCAL LABORATORY EVALUATIONS & PROCEDURES**														
**HIV testing[7]**	X	X	X	X		X	X	X	X	X	X	X	X	X
**HBV and HCV testing[8]**	X													
**CBC with differential**	X	X	X	X		X	X	X	X	X	X	X		

	Screen	Oral Phase			1-WEEK WASHOUT	Injection And Tail Phase Follow-up								
		Day 0 Enr	Week 2 Safety	Week 4 Post Oral Drug		Week 5 First Injection	Week 6, 9, 13 Safety	Week 17 Second Injection	Week 18, 23 Safety	Week 29 Third Injection	Week 30, 35 Safety	Week 41 Primary Endpoint	Week 53, 65, 77 Tail Phase	Week 81 Final Visit
**Chemistry testing[9]**	X	X	X	X		X	X	X	X	X	X	X		
**LFTs (AST, ALT, total bilirubin, [at screening only, direct bilirubin in addition to total] alkaline phosphatase)**	X	X	X	X		X	X	X	X	X	X	X	X	X
**Fasting lipid profile[10]**		X										X		
**Syphilis serologic testing men and women[5]**	X									X			X	
**Urine/ vaginal swab GC/CT testing men and women[5]**	X									X			X	
**Rectal swab GC/CT testing[5]**		X								X			X	
**Urinalysis[11]**		X										X		
**Plasma storage[12]**	X	X	X	X		X	X	X	X	X	X	X	X	X
**Plasma storage for Pharmacology testing[13]**			X	X		X	X	X	X	X	X	X	X	X
**Sample storage for Pharmacogenomic testing[14]**		X												

FOOTNOTES FOR APPENDIX I

[1] Adherence counseling will be performed at Day 0 and Week 2; pill counts will be performed at Week 2 and 4.

[2] Targeted history and physical exam for ascertainment of eligibility at Screening, including a bleeding history at Screening; complete history and physical exam at Enrollment only; targeted history and physical exam at all other follow-up visits. Refer to the SSP Manual for procedures and evaluations included in the targeted and full physical exams. Sites may perform the complete physical exam and history required at Enrollment during Screening per the discretion of the IoR or designee.

[3] A 12-lead ECG will be performed at Screening, Enrollment, and Weeks 4, 6, 9, 23, and 35.

[4] Urine pregnancy testing is required for women of reproductive potential only. The assay used must have sensitivity of ≤25 mIU/mL βHCG. At the oral dosing visits (Day 0, Week 2 and Week 4) and injection visits (Weeks 5, 17, and 29), a urine pregnancy test must be performed and pregnancy must be ruled out PRIOR to administering the study product. Urine pregnancy testing may be performed in the clinic or the laboratory. Pregnancy testing is not required if a positive result was obtained at a prior visit and the participant is still pregnant. Refer to the SSP Manual for instructions regarding follow-up of participants with a positive pregnancy test result at Weeks 2, 4, or 5; also see Section 5.23 of the protocol.

[5] Refer to the SSP Manual for details regarding STI testing. STI testing (urine and/or vaginal swab for GC/CT, syphilis testing), will be performed at Screening and Weeks 29 and 53. Rectal swabs for GC/CT will be performed at enrollment (or for US sites, at screening per IoR discretion) and weeks 29 and 53. For non-US sites, rectal swabs will be batched and shipped to the HPTN LC for testing (see SSP Manual); for US sites, testing will be performed locally.

[6] Administer injection with counseling, including review of prohibited medications, after confirmation of negative pregnancy and HIV testing per SSP.

[7] The HIV testing algorithm is provided in the SSP Manual. If HIV rapid testing is included in the HIV testing algorithm, this testing may be performed in the clinic or the laboratory. Participants who have one or more reactive or positive HIV test result (any assay) at Screening or Enrollment are not eligible to participate in the study, even if they are confirmed to be HIV-uninfected. Additional testing is required for participants who have a reactive or positive HIV test after Enrollment (see Appendix 1X and the SSP Manual). In all cases, HIV acquisition after Enrollment must be confirmed using two specimens collected on different dates (see SSP Manual). HIV testing does not need to be performed after confirmation of HIV infection (based on results from samples collected on two separate dates). Refer to SSP Manual for instructions regarding follow-up for participants who have a reactive or positive HIV test at Weeks 2, 4 or 5; also see Section 5.21 of the protocol.

[8] Testing for hepatitis B virus (HBV) and hepatitis C virus (HCV) includes hepatitis B surface antigen (HBsAg), hepatitis B surface antibody (HBsAb), hepatitis B core antibody (HBcAb), and hepatitis C antibody (HCAb).

[9] Chemistry testing includes: BUN, urea, Na, K, Cl, CO_2, CPK, creatinine, total protein, glucose, calcium, phosphorous, amylase, lipase, and magnesium.

[10] The fasting lipid profile includes total cholesterol, HDL, triglycerides, and LDL (either calculated or measured). Participants should have fasted for at least 8 hours, preferably 12 hours, prior to sample collection.

[11] Urinalysis includes protein and glucose; this testing may be performed in the clinic or the laboratory. Results from urinalysis are not needed prior to enrollment.

[12] Stored plasma will be used for Quality Assurance testing at the HPTN LC and for other assessments described in Section 9.2. These assessments will be performed retrospectively; results will not be returned to study sites or participants, except as noted in Section 9.2.

[13] Plasma samples collected for Pharmacology testing at Weeks 5, 17, and 29 must be drawn PRIOR to injection of study product. Refer to the SSP Manual).

[14] Specimens will be stored at Enrollment for Pharmacogenomic testing only at sites that opt to include this testing (See SSP Manual for further details).

[15] All acceptability assessments may not be performed at all sites. The assessments are listed below. Also, refer to Section 9 and 11 of the SSP Manual.

- Baseline Acceptability Questionnaire – Enrollment
- Follow-Up Acceptability Questionnaire – Weeks 6, 18, and 30
- Study Medication Satisfaction Questionnaire – Weeks 6, 18, and 30
- Study Medication Satisfaction Change Questionnaire – Week 18 only
- Behavioral Questionnaire – Enrollment and Weeks 5, 17, 29, 41, 53, 65, and 77

Appendix II: Schedule of Procedures and Evaluations – For Participants Who Complete Two Injections Only in Cohort 1

	Injection And Tail Phase Follow-up								
	Week 5 First Injection	Week 6, 9, 13 Safety	Week 17 Second Injection	Week 18, 23 Safety	Week 29 Follow Up	Week 41 Follow Up	Week 53 Follow Up	Week 65 Follow Up	Week 69 Final
**ADMINISTRATIVE, BEHAVIORAL, REGULATORY**									
**Locator information**	X	X	X	X	X	X	X	X	
**HIV counseling**	X	X	X	X	X	X	X	X	X
**Offer condoms and lubricant**	X	X	X	X	X	X	X	X	X
**Acceptability assessment (Week 6 and 18 only)[15]**		X		X					
**Behavioral assessment**	X		X		X	X	X	X	
**CLINICAL EVALUATIONS & PROCEDURES**									
**History, con meds, physical exam[2]**	X	X	X	X	X	X	X	X	X
**CNS symptom assessment**	X	X	X	X	X	X	X	X	X
**ECG[3]**		X		X					
**Blood collection**	X	X	X	X	X	X	X	X	X
**Urine collection for GC/CT testing men and women[5]**					X		X		
**Urine collection for urinalysis[11]**						X			
**Urine pregnancy testing [4]**	X		X		X	X	X	X	X
**Rectal swab for GC/CT testing men and women[5]**					X		X		
**Vaginal swab for GC/CT testing[5] (Optional alternate collection for women if urine not used)**					X		X		
**Administer injection[6]**	X		X						
**ISR evaluation**		X		X					
**LOCAL LABORATORY EVALUATIONS**									
**HIV testing[7]**	X	X	X	X	X	X	X	X	X
**CBC with differential**	X	X	X	X	X	X			
**Chemistry testing[9]**	X	X	X	X	X	X			
**LFTs (AST, ALT, total bilirubin, alkaline phosphatase)**	X	X	X	X	X	X	X	X	X
**Fasting lipid profile[10]**						X			

	Injection And Tail Phase Follow-up								
	**Week 5 First Injection**	**Week 6, 9, 13 Safety**	**Week 17 Second Injection**	**Week 18, 23 Safety**	**Week 29 Follow Up**	**Week 41 Follow Up**	**Week 53 Follow Up**	**Week 65 Follow Up**	**Week 69 Final**
**Syphilis serologic testing men and women[5]**					X		X		
**Urine/vaginal swab GC/CT testing men and women[5]**					X		X		
**Urinalysis[11]**						X			
**Plasma storage[12]**	X	X	X	X	X	X	X	X	X
**Plasma storage for Pharmacology testing[13]**	X	X	X	X	X	X	X	X	X

SEE FOOTNOTES FOR APPENDIX I

Appendix III: Schedule of Procedures and Evaluations – For Participants Who Complete One Injection Only in Cohort 1

	Injection And Tail Phase Follow-up						
	**Week 5 First Injection**	**Week 6, 9, 13 Safety**	**Week 17 Follow Up**	**Week 29 Follow Up**	**Week 41 Follow Up**	**Week 53 Follow Up**	**Week 57 Final Visit**
**ADMINISTRATIVE, BEHAVIORAL, REGULATORY**							
**Locator information**	X	X	X	X	X	X	X
**HIV counseling**	X	X	X	X	X	X	X
**Offer condoms and lubricant**	X	X	X	X	X	X	X
**Tolerability/Acceptability Assessment (Week 6 only)**[15]		X					
**Behavioral assessment**	X		X	X	X	X	
**CLINICAL EVALUATIONS & PROCEDURES**							
**History, con meds, physical exam**[2]	X	X	X	X	X	X	X
**CNS symptom assessment**	X	X	X	X	X	X	X
**ECG**[3]		X					
**Blood collection**	X	X	X	X	X	X	X
**Urine collection for GC/CT testing men and women**[5]				X		X	
**Urine collection for urinalysis**[11]					X		
**Urine pregnancy testing**[4]	X		X	X	X	X	X
**Rectal swab for GC/CT testing men and women**[5]				X		X	
**Vaginal swab for GC/CT testing**[5] **(Optional alternate collection for women if urine not used)**				X		X	
**Administer injection**[6]	X						
**ISR Evaluation**		X					
**LOCAL LABORATORY EVALUATIONS**							
**HIV testing**[7]	X	X	X	X	X	X	X
**CBC with differential**	X	X	X	X	X		
**Chemistry testing**[9]	X	X	X	X	X		
**LFTs (AST, ALT, total bilirubin, alkaline phosphatase)**	X	X	X	X	X	X	X
**Fasting lipid profile**[10]					X		

	Injection And Tail Phase Follow-up						
	**Week 5 First Injection**	**Week 6, 9, 13 Safety**	**Week 17 Follow Up**	**Week 29 Follow Up**	**Week 41 Follow Up**	**Week 53 Follow Up**	**Week 57 Final Visit**
**Syphilis serologic testing men and women[5]**				X		X	
**Urine/vaginal swab GC/CT testing men and women[5]**				X		X	
**Rectal swab GC/CT testing[5]**				X		X	
**Urinalysis[11]**					X		
**Plasma storage[12]**	X	X	X	X	X	X	X
**Plasma storage for Pharmacology testing[13]**	X	X	X	X	X	X	X

SEE FOOTNOTES FOR APPENDIX I

Appendix IV: Schedule of Procedures and Evaluations – For Participants Who Complete All Five Injections in Cohort 2

		Oral Phase				Injection And Tail Phase Follow-up												
	Screen	Day 0 Enr	Wk 2 Safety	Week 4 Post Oral Drug	1-WEEK WASHOUT	Wk 5 1st Inj	Wk 6 Safety	Wk 9 2nd Inj	Wk 10, 13 Safety	Wk 17 3rd Inj	Wk 18, 21 Safety	Wk 25 4th Inj	Wk 26, 29 Safety	Wk 33 5th Inj	Wk 34, 37 Safety	Wk 41 1º Endpt	Wk 53, 65, 77 Tail	Wk 85 Final Visit
**ADMINISTRATIVE, BEHAVIORAL, REGULATORY**																		
**Informed consent**	X																	
**Locator information**	X	X	X	X		X	X	X	X	X	X	X	X	X	X	X	X	
**Demographic information**		X																
**Randomization**		X																
**HIV counseling**	X	X	X	X		X	X	X	X	X	X	X	X	X	X	X	X	X
**Offer condoms and lubricant**	X	X	X	X		X	X	X	X	X	X	X	X	X	X	X	X	X
**Acceptability assessments (Weeks 0, 6, 10, 18, 26, and 34 only)[15]**		X					X		X		X		X		X			
**Behavioral assessment**		X				X		X		X		X		X		X	X	
**Adherence counseling/pill count[1]**		X	X	X														
**AUDIT questionnaire**	X																	
**CLINICAL EVALUATIONS & PROCEDURES**																		
**History (including bleeding history at Screening), con meds, physical exam[2]**	X	X	X	X		X	X	X	X	X	X	X	X	X	X	X	X	X
**CNS symptom assessment**			X	X		X	X	X	X	X	X	X	X	X	X	X	X	X
**ECG[3]**	X	X		X			X		X		X		X		X			
**Blood collection**	X	X	X	X		X	X	X	X	X	X	X	X	X	X	X	X	X
**Urine collection for GC/CT testing men and women[5]**	X											X					X	
**Urine collection for urinalysis**		X														X		
**Urine pregnancy testing[4]**	X	X	X	X		X		X		X		X		X		X	X	X
**Rectal swab for GC/CT testing men and women[5]**		X										X					X	
**Vaginal swab for GC/CT testing[5] (Optional alternate collection for women if urine not used)**	X											X					X	
**Provide oral study drug**		X																
**Administer injection[6]**						X		X		X		X		X				
**ISR evaluation**							X		X		X		X		X			

		Oral Phase			1-WEEK WASHOUT	Injection And Tail Phase Follow-up												
	Screen	Day 0 Enr	Wk 2 Safety	Week 4 Post Oral Drug		Wk 5 1st Inj	Wk 6 Safety	Wk 9 2nd Inj	Wk 10, 13 Safety	Wk 17 3rd Inj	Wk 18, 21 Safety	Wk 25 4th Inj	Wk 26, 29 Safety	Wk 33 5th Inj	Wk 34, 37 Safety	Wk 41 1º Endpt	Wk 53, 65, 77 Tail	Wk 85 Final Visit
**LOCAL LABORATORY EVALUATIONS & PROCEDURES**																		
**HIV testing[7]**	X	X	X	X		X	X	X	X	X	X	X	X	X	X	X	X	X
**HBV and HCV testing[8]**	X																	
**CBC with differential**	X	X	X	X		X	X	X	X	X	X	X	X	X	X	X		
**Chemistry testing[9]**	X	X	X	X		X	X	X	X	X	X	X	X	X	X	X		
**LFTs (AST, ALT, total bilirubin, [at screening only, direct bilirubin in addition to total] alkaline phosphatase)**	X	X	X	X		X	X	X	X	X	X	X	X	X	X	X	X	X
**Fasting lipid profile[10]**		X														X		
**Syphilis serologic testing men and women[5]**	X											X					X	
**Urine/ vaginal swab GC/CT testing men and women[5]**	X											X					X	
**Rectal swab GC/CT testing[5]**		X										X					X	
**Urinalysis[11]**		X														X		
**Plasma storage[12]**	X	X	X	X		X	X	X	X	X	X	X	X	X	X	X	X	X
**Plasma storage for Pharmacology testing[13]**			X	X		X	X	X	X	X	X	X	X	X	X	X	X	X
**Sample storage for Pharmacogenomic testing[14]**		X																

FOOTNOTES FOR APPENDIX IV

[1] Adherence counseling will be performed at Day 0 and Week 2; pill counts will be performed at Week 2 and 4.

[2] Targeted history and physical exam for ascertainment of eligibility at Screening, including a bleeding history at Screening; complete history and physical exam at Enrollment only; targeted history and physical exam at all other follow-up visits. Refer to the SSP Manual for procedures and evaluations included in the targeted and full physical exams. Sites may perform the complete physical exam and history required at Enrollment during Screening per the discretion of the IoR or designee.

[3] A 12-lead ECG will be performed at Screening, Enrollment, and Weeks 4, 6, 13, 21, 29, and 37

[4] Urine pregnancy testing is required for women of reproductive potential only. The assay used must have sensitivity of ≤25 mIU/mL βHCG. At the oral dosing visits (Day 0, Week 2 and Week 4) and injection visits (Weeks 5, 9, 17, 25, and 33), a urine pregnancy test must be performed and pregnancy must be ruled out PRIOR to administering the study product. Urine pregnancy testing may be performed in the clinic or the laboratory. Pregnancy testing is not required if a positive result was obtained at a prior visit and the participant is still pregnant. Refer to the SSP Manual for instructions regarding follow-up of participants with a positive pregnancy test result at Weeks 2, 4, or 5; also see Section 5.23 of the protocol.

[5] Refer to the SSP Manual for details regarding STI testing. STI testing (urine and/or vaginal swab for GC/CT, syphilis testing), will be performed at Screening and Weeks 25 and 53. Rectal swabs for GC/CT will be performed at enrollment (or for US sites, at screening per IoR discretion) and weeks 25 and 53. For non-US sites, rectal swabs will be batched and shipped to the HPTN LC for testing (see SSP Manual); for US sites, testing will be performed locally.

[6] Administer injection with counseling, including review of prohibited medications, after confirmation of negative pregnancy and HIV testing per SSP Manual.

[7] The HIV testing algorithm is provided in the SSP Manual. If HIV rapid testing is included in the HIV testing algorithm, this testing may be performed in the clinic or the laboratory. Participants who have one or more reactive or positive HIV test result (any assay) at Screening or Enrollment are not eligible to participate in the study, even if they are confirmed to be HIV-uninfected. Additional testing is required for participants who have a reactive or positive HIV test after Enrollment (see Appendix IX and the SSP Manual). In all cases, HIV acquisition after Enrollment must be confirmed using two specimens collected on different dates (see SSP Manual). HIV testing does not need to be performed after confirmation of HIV infection (based on results from samples collected on two separate dates). Refer to SSP Manual for instructions regarding follow-up for participants who have a reactive or positive HIV test at Weeks 2, 4 or 5; also see Section 5.21 of the protocol.

[8]Testing for hepatitis B virus (HBV) and hepatitis C virus (HCV) includes hepatitis B surface antigen (HBsAg), hepatitis B surface antibody (HbsAb), hepatitis B core antibody (HbcAb), and hepatitis C antibody (HCAb).

[9] Chemistry testing includes: BUN, urea, Na, K, Cl, CO_2, CPK, creatinine, total protein, glucose, calcium, phosphorous, amylase, lipase, and magnesium.

[10] The fasting lipid profile includes total cholesterol, HDL, triglycerides, and LDL (either calculated or measured). Participants should have fasted for at least 8 hours, preferably 12 hours, prior to sample collection.

[11] Urinalysis includes protein and glucose; this testing may be performed in the clinic or the laboratory. Results from urinalysis are not needed prior to enrollment.

[12] Stored plasma will be used for Quality Assurance testing at the HPTN LC and for other assessments described in Section 9.2. These assessments will be performed retrospectively; results will not be returned to study sites or participants, except as noted in Section 9.2.

[13] Plasma samples collected for Pharmacology testing at Weeks 5, 9, 17, 25, and 33 must be drawn PRIOR to injection of study product. Refer to the SSP Manual).

[14] Specimens will be stored at Enrollment for Pharmacogenomic testing only at sites that opt to include this testing (See SSP Manual for further details).

[15] All acceptability assessments may not be performed at all sites. The assessments are listed below. Also, refer to Section 9 and 11 of the SSP Manual.

- Baseline Acceptability Questionnaire – Enrollment
- Follow-Up Acceptability Questionnaire – Weeks 6, 10, 18, 26, and 34
- Study Medication Satisfaction Questionnaire – Weeks 6, 10, 18, 26, and 34
- Study Medication Satisfaction Change Questionnaire – Week 10 only
- Behavioral Questionnaire – Enrollment and Weeks 5, 9, 17, 25, 33, 41, 53, 65, and 77

Appendix V: Schedule of Procedures and Evaluations – For Participants Who Complete Four Injections Only in Cohort 2

	Injection And Tail Phase Follow-up									
	**Week 5 First Injection**	**Week 6 Safety**	**Week 9 Second Injection**	**Week 10, 13 Safety**	**Week 17 Third Injection**	**Week 18, 21 Safety**	**Week 25 Fourth Injection**	**Week 26, 29 Safety**	**Week 41, 53, 65 Follow Up**	**Week 77 Final**
**ADMINISTRATIVE, BEHAVIORAL, REGULATORY**										
**Locator information**	X	X	X	X	X	X	X	X	X	
**HIV counseling**	X	X	X	X	X	X	X	X	X	X
**Offer condoms and lubricant**	X	X	X	X	X	X	X	X	X	X
**Acceptability assessment (Week 6, 10, 18, and 26 only)[1]**		X		X		X		X		
**Behavioral assessment**	X		X		X		X		X	
**CLINICAL EVALUATIONS & PROCEDURES**										
**History, con meds, physical exam**	X	X	X	X	X	X	X	X	X	X
**CNS symptom assessment**	X	X	X	X	X	X	X	X	X	X
**ECG (Week 6, 13, 21, and 29 only)**		X		X		X		X		
**Blood collection**	X	X	X	X	X	X	X	X	X	X
**Urine collection for GC/CT testing men and women[2]**							X		X	
**Urine collection for urinalysis[3]**									X	
**Urine pregnancy testing[4]**	X		X		X		X		X	X
**Rectal swab for GC/CT testing men and women[2]**							X		X	
**Vaginal swab for GC/CT testing (Optional alternate collection for women if urine not used)[2]**							X		X	
**Administer injection[5]**	X		X		X		X			
**ISR evaluation**		X		X		X		X		
**LOCAL LABORATORY EVALUATIONS**										
**HIV testing[6]**	X	X	X	X	X	X	X	X	X	X
**CBC with differential (Week 41 only)**	X	X	X	X	X	X	X	X	X	
**Chemistry testing[7] (Week 41 only)**	X	X	X	X	X	X	X	X	X	
**LFTs (AST, ALT, total bilirubin, alkaline phosphatase)**	X	X	X	X	X	X	X	X	X	X

	Injection And Tail Phase Follow-up									
	**Week 5 First Injection**	**Week 6 Safety**	**Week 9 Second Injection**	**Week 10, 13 Safety**	**Week 17 Third Injection**	**Week 18, 21 Safety**	**Week 25 Fourth Injection**	**Week 26, 29 Safety**	**Week 41, 53, 65 Follow Up**	**Week 77 Final**
**Fasting lipid profile[8]**									X	
**Syphilis serologic testing men and women[2]**							X		X	
**Urine/vaginal swab GC/CT testing men and women[2]**							X		X	
**Rectal swab GC/CT testing[2]**							X		X	
**Urinalysis[3]**									X	
**Plasma storage[9]**	X	X	X	X	X	X	X	X	X	X
**Plasma storage for Pharmacology testing[10]**	X	X	X	X	X	X	X	X	X	X

FOOTNOTES FOR APPENDIX V

1 All assessments may not be performed at all sites. The assessments for this visit schedule are listed below. Also, refer to Section 9 and 11 of the SSP Manual.

•Follow-Up Acceptability Questionnaire – Weeks 6, 10, 18, 26

•Study Medication Satisfaction Questionnaire – Weeks 6, 10, 18, 26

•Study Medication Satisfaction Change Questionnaire – Week 10 only

•Behavioral Questionnaire – Weeks 5, 9, 17, 25, 41, 53, and 65

2 Refer to the SSP Manual for details regarding STI testing. STI testing (urine and/or vaginal swab for GC/CT, syphilis testing), will be performed at Weeks 25 and 53. Rectal swabs for GC/CT will be performed at 25 and 53. For non-US sites, rectal swabs will be batched and shipped to the HPTN LC for testing (see SSP Manual); for US sites, testing will be performed locally.

3 Week 41 only. Urinalysis includes protein and glucose; this testing may be performed in the clinic or the laboratory.

4 Urine pregnancy testing is required for women of reproductive potential only. The assay used must have sensitivity of ≤25 mIU/mL βHCG. At the injection visits (Weeks 5, 9, 17, 25), a urine pregnancy test must be performed and pregnancy must be ruled out PRIOR to administering the study product. Urine pregnancy testing may be performed in the clinic or the laboratory. Pregnancy testing is not required if a positive result was obtained at a prior visit and the participant is still pregnant. Refer to the SSP Manual for instructions regarding follow-up of participants with a positive pregnancy test result at Week 5; also see Section 5.23 of the protocol.

5 Administer injection with counseling, including review of prohibited medications, after confirmation of negative pregnancy and HIV testing per SSP Manual.

6 The HIV testing algorithm is provided in the SSP Manual. If HIV rapid testing is included in the HIV testing algorithm, this testing may be performed in the clinic or the laboratory. Additional testing is required for participants who have a reactive or positive HIV test after Enrollment (see Appendix IX and the SSP Manual). In all cases, HIV acquisition after Enrollment must be confirmed using two specimens collected on different dates (see SSP Manual). HIV testing does not need to be performed after confirmation of HIV infection (based on results from samples collected on two separate dates). Refer to SSP Manual for instructions regarding follow-up for participants who have a reactive or positive HIV test at Week 5; also see Section 5.21 of the protocol.

7 Chemistry testing includes: BUN, urea, Na, K, Cl, CO2, CPK, creatinine, total protein, glucose, calcium, phosphorous, amylase, lipase, and magnesium.

8 Week 41 only. The fasting lipid profile includes total cholesterol, HDL, triglycerides, and LDL (either calculated or measured). Participants should have fasted for at least 8 hours, preferably 12 hours, prior to sample collection.

9 Stored plasma will be used for Quality Assurance testing at the HPTN LC and for other assessments described in Section 9.2. These assessments will be performed retrospectively; results will not be returned to study sites or participants, except as noted in Section 9.2.

10 Plasma samples collected for Pharmacology testing at Weeks 5, 9, 17, and 25 must be drawn PRIOR to injection of study product. Refer to the SSP Manual.

Appendix VI: Schedule of Procedures and Evaluations – For Participants Who Complete Three Injections Only in Cohort 2

	Injection And Tail Phase Follow-up							
	Week 5 First Injection	Week 6 Safety	Week 9 Second Injection	Week 10, 13 Safety	Week 17 Third Injection	Week 18, 21 Safety	Week 29, 41, 53 Follow Up	Week 69 Final
**ADMINISTRATIVE, BEHAVIORAL, REGULATORY**								
**Locator information**	X	X	X	X	X	X	X	
**HIV counseling**	X	X	X	X	X	X	X	X
**Offer condoms and lubricant**	X	X	X	X	X	X	X	X
**Acceptability assessment (Week 6, 10, and 18 only)[1]**		X		X		X		
**Behavioral assessment**	X		X		X		X	
**CLINICAL EVALUATIONS & PROCEDURES**								
**History, con meds, physical exam**	X	X	X	X	X	X	X	X
**CNS symptom assessment**	X	X	X	X	X	X	X	X
**ECG (Week 6, 13, 21 only)**		X		X		X		
**Blood collection**	X	X	X	X	X	X	X	X
**Urine collection for GC/CT testing men and women[2]**							X	
**Urine collection for urinalysis[3]**						X	X	
**Urine pregnancy testing[4]**	X		X		X		X	X
**Rectal swab for GC/CT testing men and women[2]**						X	X	
**Vaginal swab for GC/CT testing[5] (Optional alternate collection for women if urine not used)[2]**						X	X	
**Administer injection[5]**	X		X		X			
**ISR evaluation**		X		X		X		
**LOCAL LABORATORY EVALUATIONS**								
**HIV testing[6]**	X	X	X	X	X	X	X	X
**CBC with differential (Week 29 and 41 only)**	X	X	X	X	X	X	X	
**Chemistry testing[7] (Week 29 and 41 only)**	X	X	X	X	X	X	X	
**LFTs (AST, ALT, total bilirubin, alkaline phosphatase)**	X	X	X	X	X	X	X	X
**Fasting lipid profile[8]**							X	

	Injection And Tail Phase Follow-up							
	Week 5 First Injection	Week 6 Safety	Week 9 Second Injection	Week 10, 13 Safety	Week 17 Third Injection	Week 18, 21 Safety	Week 29, 41, 53 Follow Up	Week 69 Final
**Syphilis serologic testing men and women[2]**						X	X	
**Urine/vaginal swab GC/CT testing men and women[2]**						X	X	
**Rectal swab GC/CT testing[2]**						X	X	
**Urinalysis[3]**							X	
**Plasma storage[9]**	X	X	X	X	X	X	X	X
**Plasma storage for Pharmacology testing[10]**	X	X	X	X	X	X	X	X

FOOTNOTES FOR APPENDIX VI

1 All assessments may not be performed at all sites. The assessments for this visit schedule are listed below. Also, refer to Section 9 and 11 of the SSP Manual.

•Follow-Up Acceptability Questionnaire – Weeks 6, 10, 18,

•Study Medication Satisfaction Questionnaire – Weeks 6, 10, 18,

•Study Medication Satisfaction Change Questionnaire – Week 10 only

•Behavioral Questionnaire – Weeks 5, 9, 17, 29, 41, and 53

2 Refer to the SSP Manual for details regarding STI testing. STI testing (urine and/or vaginal swab for GC/CT, syphilis testing), will be performed at Weeks 21 and 53. Rectal swabs for GC/CT will be performed at 21 and 53. For non-US sites, rectal swabs will be batched and shipped to the HPTN LC for testing (see SSP Manual); for US sites, testing will be performed locally.

3 Week 41 only. Urinalysis includes protein and glucose; this testing may be performed in the clinic or the laboratory.

4 Urine pregnancy testing is required for women of reproductive potential only. The assay used must have sensitivity of ≤25 mIU/mL βHCG. At the injection visits (Weeks 5, 9, 17), a urine pregnancy test must be performed and pregnancy must be ruled out PRIOR to administering the study product. Urine pregnancy testing may be performed in the clinic or the laboratory. Pregnancy testing is not required if a positive result was obtained at a prior visit and the participant is still pregnant. Refer to the SSP Manual for instructions regarding follow-up of participants with a positive pregnancy test result at Week 5; also see Section 5.23 of the protocol.

5 Administer injection with counseling, including review of prohibited medications, after confirmation of negative pregnancy and HIV testing per SSP Manual.

6 The HIV testing algorithm is provided in the SSP Manual. If HIV rapid testing is included in the HIV testing algorithm, this testing may be performed in the clinic or the laboratory. Additional testing is required for participants who have a reactive or positive HIV test after Enrollment (see Appendix IX and the SSP Manual). In all cases, HIV acquisition after Enrollment must be confirmed using two specimens collected on different dates (see SSP Manual). HIV testing does not need to be performed after confirmation of HIV infection (based on results from samples collected on two separate dates). Refer to SSP Manual for instructions regarding follow-up for participants who have a reactive or positive HIV test at Week 5; also see Section 5.21 of the protocol.

7 Chemistry testing includes: BUN, urea, Na, K, Cl, CO2, CPK, creatinine, total protein, glucose, calcium, phosphorous, amylase, lipase, and magnesium.

8 Week 41 only. The fasting lipid profile includes total cholesterol, HDL, triglycerides, and LDL (either calculated or measured). Participants should have fasted for at least 8 hours, preferably 12 hours, prior to sample collection.

9 Stored plasma will be used for Quality Assurance testing at the HPTN LC and for other assessments described in Section 9.2. These assessments will be performed retrospectively; results will not be returned to study sites or participants, except as noted in Section 9.2.

10 Plasma samples collected for Pharmacology testing at Weeks 5, 9, and 17 must be drawn PRIOR to injection of study product. Refer to the SSP Manual.

Appendix VII: Schedule of Procedures and Evaluations – For Participants Who Complete Two Injections Only in Cohort 2

	Injection And Tail Phase Follow-up							
	Week 5 First Injection	Week 6 Safety	Week 9 Second Injection	Week 10, 13 Safety	Week 27 Follow Up	Week 41 Follow Up	Week 53 Follow Up	Week 61 Final Visit
**ADMINISTRATIVE, BEHAVIORAL, REGULATORY**								
**Locator information**	X	X	X	X	X	X	X	
**HIV counseling**	X	X	X	X	X	X	X	X
**Offer condoms and lubricant**	X	X	X	X	X	X	X	X
**Acceptability Assessment (Week 6 and 10 only)[1]**		X		X				
**Behavioral assessment**	X		X		X	X	X	
**CLINICAL EVALUATIONS & PROCEDURES**								
**History, con meds, physical exam**	X	X	X	X	X	X	X	X
**CNS symptom assessment**	X	X	X	X	X	X	X	X
**ECG (Week 6 and 13 only)**		X		X				
**Blood collection**	X	X	X	X	X	X	X	X
**Urine collection for GC/CT testing men and women[2]**					X		X	
**Urine collection for urinalysis[3]**						X		
**Urine pregnancy testing[4]**	X		X	X	X	X	X	X
**Rectal swab for GC/CT testing men and women[2]**					X		X	
**Vaginal swab for GC/CT testing (Optional alternate collection for women if urine not used)[2]**					X		X	
**Administer injection[5]**	X		X					
**ISR Evaluation**		X		X				
**LOCAL LABORATORY EVALUATIONS**								
**HIV testing[6]**	X	X	X	X	X	X	X	X
**CBC with differential**	X	X	X	X	X	X		
**Chemistry testing[7]**	X	X	X	X	X	X		
**LFTs (AST, ALT, total bilirubin, alkaline phosphatase)**	X	X	X	X	X	X	X	X

	Injection And Tail Phase Follow-up							
	Week 5 First Injection	Week 6 Safety	Week 9 Second Injection	Week 10, 13 Safety	Week 27 Follow Up	Week 41 Follow Up	Week 53 Follow Up	Week 61 Final Visit
Fasting lipid profile[8]						X		
Syphilis serologic testing men and women[2]					X		X	
Urine/vaginal swab GC/CT testing men and women					X		X	
Rectal swab GC/CT testing[2]					X		X	
Urinalysis[3]						X		
Plasma storage[9]	X	X	X	X	X	X	X	X
Plasma storage for Pharmacology testing[10]	X	X	X	X	X	X	X	X

FOOTNOTES FOR APPENDIX VII

1 All assessments may not be performed at all sites. The assessments for this visit schedule are listed below. Also, refer to Section 9 and 11 of the SSP Manual.

•Follow-Up Acceptability Questionnaire – Weeks 6, 10

•Study Medication Satisfaction Questionnaire – Weeks 6, 10

•Study Medication Satisfaction Change Questionnaire – Week 10 only

•Behavioral Questionnaire – Weeks 5, 9, 27, 41, 53

2 Refer to the SSP Manual for details regarding STI testing. STI testing (urine and/or vaginal swab for GC/CT, syphilis testing), will be performed at Weeks 27 and 53. Rectal swabs for GC/CT will be performed at 27 and 53. For non-US sites, rectal swabs will be batched and shipped to the HPTN LC for testing (see SSP Manual); for US sites, testing will be performed locally.

3 Urinalysis includes protein and glucose; this testing may be performed in the clinic or the laboratory.

4 Urine pregnancy testing is required for women of reproductive potential only. The assay used must have sensitivity of ≤25 mIU/mL βHCG. At the injection visits (Weeks 5, 9, a urine pregnancy test must be performed and pregnancy must be ruled out PRIOR to administering the study product. Urine pregnancy testing may be performed in the clinic or the laboratory. Pregnancy testing is not required if a positive result was obtained at a prior visit and the participant is still pregnant. Refer to the SSP Manual for instructions regarding follow-up of participants with a positive pregnancy test result at Week 5; also see Section 5.23 of the protocol.

5 Administer injection with counseling, including review of prohibited medications, after confirmation of negative pregnancy and HIV testing per SSP Manual.

6 The HIV testing algorithm is provided in the SSP Manual. If HIV rapid testing is included in the HIV testing algorithm, this testing may be performed in the clinic or the laboratory. Additional testing is required for participants who have a reactive or positive HIV test after Enrollment (see Appendix IX and the SSP Manual). In all cases, HIV acquisition after Enrollment must be confirmed using two specimens collected on different dates (see SSP Manual). HIV testing does not need to be performed after confirmation of HIV infection (based on results from samples collected on two separate dates). Refer to SSP Manual for instructions regarding follow-up for participants who have a reactive or positive HIV test at Week 5; also see Section 5.21 of the protocol.

7 Chemistry testing includes: BUN, urea, Na, K, Cl, CO2, CPK, creatinine, total protein, glucose, calcium, phosphorous, amylase, lipase, and magnesium.

8 Week 41 only. The fasting lipid profile includes total cholesterol, HDL, triglycerides, and LDL (either calculated or measured). Participants should have fasted for at least 8 hours, preferably 12 hours, prior to sample collection.

9 Stored plasma will be used for Quality Assurance testing at the HPTN LC and for other assessments described in Section 9.2. These assessments will be performed retrospectively; results will not be returned to study sites or participants, except as noted in Section 9.2.

10 Plasma samples collected for Pharmacology testing at Weeks 5 and 9, must be drawn PRIOR to injection of study product. Refer to the SSP Manual.

Appendix VIII: Schedule of Procedures and Evaluations – For Participants Who Complete One Injection Only in Cohort 2

	Injection And Tail Phase Follow-up							
	Week 5 First Injection	Week 6 Safety	Week 9 Follow Up	Week 13 Follow Up	Week 27 Follow Up	Week 41 Follow Up	Week 53 Follow Up	Week 57 Final Visit
**ADMINISTRATIVE, BEHAVIORAL, REGULATORY**								
**Locator information**	X	X	X	X	X	X	X	X
**HIV counseling**	X	X	X	X	X	X	X	X
**Offer condoms and lubricant**	X	X	X	X	X	X	X	X
**Acceptability Assessment (Week 6 only)[1]**		X						
**Behavioral assessment**	X			X	X	X	X	
**CLINICAL EVALUATIONS & PROCEDURES**								
**History, con meds, physical exam**	X	X	X	X	X	X	X	X
**CNS symptom assessment**	X	X	X	X	X	X	X	X
**ECG (Week 6 only)**		X						
**Blood collection**	X	X	X	X	X	X	X	X
**Urine collection for GC/CT testing men and women[2]**					X		X	
**Urine collection for urinalysis[3]**						X		
**Urine pregnancy testing[4]**	X		X	X	X	X	X	X
**Rectal swab for GC/CT testing men and women[2]**					X		X	
**Vaginal swab for GC/CT testing (Optional alternate collection for women if urine not used)[2]**					X		X	
**Administer injection[5]**	X							
**ISR Evaluation**		X						
**LOCAL LABORATORY EVALUATIONS**								
**HIV testing[6]**	X	X	X	X	X	X	X	X
**CBC with differential**	X	X	X	X	X	X		
**Chemistry testing[7]**	X	X	X	X	X	X		
**LFTs (AST, ALT, total bilirubin, alkaline phosphatase)**	X	X	X	X	X	X	X	X
**Fasting lipid profile[8]**						X		

	Injection And Tail Phase Follow-up							
	Week 5 First Injection	Week 6 Safety	Week 9 Follow Up	Week 13 Follow Up	Week 27 Follow Up	Week 41 Follow Up	Week 53 Follow Up	Week 57 Final Visit
**Syphilis serologic testing men and women[2]**					X		X	
**Urine/vaginal swab GC/CT testing men and women[2]**					X		X	
**Rectal swab GC/CT testing[2]**					X		X	
**Urinalysis[3]**						X		
**Plasma storage[9]**	X	X	X	X	X	X	X	X
**Plasma storage for Pharmacology testing[10]**	X	X	X	X	X	X	X	X

FOOTNOTES FOR APPENDIX VIII

1 All assessments may not be performed at all sites. The assessments for this visit schedule are listed below. Also, refer to Section 9 and 11 of the SSP Manual.

•Follow-Up Acceptability Questionnaire – Weeks 6 only

•Study Medication Satisfaction Questionnaire – Weeks 6 only

•Study Medication Satisfaction Change Questionnaire – Week 9 only

•Behavioral Questionnaire – Weeks 5, 9, 27, 41, 53

2 Refer to the SSP Manual for details regarding STI testing. STI testing (urine and/or vaginal swab for GC/CT, syphilis testing), will be performed at Weeks 27 and 53. Rectal swabs for GC/CT will be performed at Weeks 27 and 53. For non-US sites, rectal swabs will be batched and shipped to the HPTN LC for testing (see SSP Manual); for US sites, testing will be performed locally.

3 Urinalysis includes protein and glucose; this testing may be performed in the clinic or the laboratory.

4 Urine pregnancy testing is required for women of reproductive potential only. The assay used must have sensitivity of ≤25 mIU/mL βHCG. At the injection visits (Weeks 5, 9, a urine pregnancy test must be performed and pregnancy must be ruled out PRIOR to administering the study product. Urine pregnancy testing may be performed in the clinic or the laboratory. Pregnancy testing is not required if a positive result was obtained at a prior visit and the participant is still pregnant. Refer to the SSP Manual for instructions regarding follow-up of participants with a positive pregnancy test result at Week 5; also see Section 5.23 of the protocol.

5 Administer injection with counseling, including review of prohibited medications, after confirmation of negative pregnancy and HIV testing per SSP Manual.

6 The HIV testing algorithm is provided in the SSP Manual. If HIV rapid testing is included in the HIV testing algorithm, this testing may be performed in the clinic or the laboratory. Additional testing is required for participants who have a reactive or positive HIV test after Enrollment (see Appendix IX and the SSP Manual). In all cases, HIV acquisition after Enrollment must be confirmed using two specimens collected on different dates (see SSP Manual). HIV testing does not need to be performed after confirmation of HIV infection (based on results from samples collected on two separate dates). Refer to SSP Manual for instructions regarding follow-up for participants who have a reactive or positive HIV test at Week 5; also see Section 5.21 of the protocol.

7 Chemistry testing includes: BUN, urea, Na, K, Cl, CO2, CPK, creatinine, total protein, glucose, calcium, phosphorous, amylase, lipase, and magnesium.

8 Week 41 only. The fasting lipid profile includes total cholesterol, HDL, triglycerides, and LDL (either calculated or measured). Participants should have fasted for at least 8 hours, preferably 12 hours, prior to sample collection.

9 Stored plasma will be used for Quality Assurance testing at the HPTN LC and for other assessments described in Section 9.2. These assessments will be performed retrospectively; results will not be returned to study sites or participants, except as noted in Section 9.2.

10 Plasma samples collected for Pharmacology testing at Week 5 must be drawn PRIOR to injection of study product. Refer to the SSP Manual.

Appendix IX: Schedule for Additional Laboratory Procedures for Enrolled Participants who have a Reactive Positive HIV Test Result (HIV Confirmation Visit – to be performed on a different day from the initial reactive/positive sample)

	**HIV Confirmation Visit**
**ADMININISTRATIVE, BEHAVIORAL, REGULATORY**	
Locator information	X
Offer condoms and lubricant	X
HIV counseling	X
**CLINICAL EVALUATIONS AND PROCEDURES**	
Blood collection	X
**LOCAL LABORATORY EVALUATIONS**	
HIV testing[1]	X
CD4 cell count	X
HIV viral load testing	X
HIV resistance testing[2]	X[2]
Plasma storage[3]	X

[1] The HIV testing algorithm for the HIV Confirmation Visit is provided in the SSP Manual. If HIV rapid testing is included in the HIV testing algorithm, this testing may be performed in the clinic or the laboratory.
[2] Sites may collect specimens for resistance testing at a local laboratory to assist with clinical management; results from resistance testing performed at local laboratories will not be reported to the SDMC. Stored plasma may not be used real-time/local resistance testing.
[3] Stored plasma will be used for Quality Assurance testing at the HPTN LC and for other assessments described in Section 9.2. These assessments will be performed retrospectively; results will not be returned to study sites or participants, except as noted in Section 9.2.

Appendix X: Risk Assessment and Mitigation Strategy

Potential Risk of Clinical Significance	Data/Rationale for Risk	Mitigation Strategy
Elevated Liver Transaminases (ALT/AST)	See section 1.4 on Hepatic Safety.	Liver transaminases will be closely monitored throughout this study, and liver chemistry stopping criteria will be adopted. All instances of liver transaminase elevations of Grade 1 and above will be followed to resolution. This risk will also be mitigated by exclusion criteria. In addition, an oral lead-in period is being implemented in this study, where all subjects will receive oral 744 for 4 weeks, to determine individual safety and tolerability, prior to the administration of 744LA. For management of Grade 1 or higher AST/ALT, refer to Section 5.3 and Appendix XI.
Injection Site Reactions (ISRs)	The occurrence of injection site reactions (ISRs) was identified in rats and monkeys at all dose levels of 744LA and associated with both the IM and SC route of administration. In humans, experience to date has demonstrated that ISRs occur in the majority of exposed subjects, but are generally mild (Grade 1) and include tenderness, erythema, or nodule formation of several days duration. Reactions to date have been well tolerated and have not to date been associated with subject withdrawal.	Subjects will be closely monitored for ISRs, particularly for signs of pain, tenderness, infections, erythema, swelling, induration, or nodules (granulomas or cysts) throughout the study. Specialist dermatology consultation will be sought if warranted for individual subjects.
Creatine Phosphokinase (CPK) Elevations	Occurrences of asymptomatic, transient instances of elevations of CPK levels have been observed in Phase I studies and an ongoing Phase 2b study with oral 744 at dose levels of 10, 30 and 60 mg (LAI116482). These generally appeared to be related to physical activity, were not associated with clinical symptoms and returned to pre-treatment levels in all cases. No subject has required a discontinuation of GSK1265744 as a result of a CPK elevation. Rhabdomyolysis of uncertain cause has been included in labeling for a currently available integrase inhibitor	Standard laboratory monitoring.

Potential Risk of Clinical Significance	Data/Rationale for Risk	Mitigation Strategy
	(raltegravir), but has not been seen in any subject receiving GSK1265744 to date.	
Bone Marrow Depletion	This risk was demonstrated in high dose (1,000 mg/kg/day) oral 744 monkey study, but was not apparent from studies conducted in rats or at lower dose levels in monkeys. Blood disorders such as anemia and leucopenia are labeled for other drugs of the integrase inhibitor class. No signal for bone marrow depletion has been identified to date through Phase 2b.	Doses used within this study will result in many fold lower level exposure compared to the effect level in primates. Careful monitoring of adverse hematological events will occur during study conduct. AEs will be managed appropriately including, but not limited to, withdrawal of 744 (oral or LA), and will be followed to resolution, as per Sponsor's standard medical monitoring practices.
Gastrointestinal (GI) Intolerability	The risk identified in a monkey toxicity study at the highest administered dose of oral 744 and considered related to local irritation (rather than a systemic effect) leading to morbidity associated with clinical signs of intolerance.	Careful monitoring of adverse GI events will occur throughout the study. AEs will be managed appropriately including, but not limited to, withdrawal of 744, and will be followed to resolution, as per Sponsor's standard medical monitoring practices.
Hypersensitivity Reactions (HSR)	While there have been no clinical cases of hypersensitivity to GSK1265744, there is a theoretical risk of systemic or severe hypersensitivity reactions with or without hepatic symptoms. The long exposures anticipated after a 744LA injection may complicate the management of a drug hypersensitivity reaction, were it to occur.	This risk of developing a hypersensitivity reaction post administration of 744LA will be minimized by the use of an oral 744 lead-in to determine individual safety and tolerability prior to the introduction of 744LA. Any reactions would be managed per standard clinical guidelines.
Drug-Drug Interactions (DDIs)	Residual concentrations of GSK1265744 would remain in the systemic circulation of subjects who stopped treatment (e.g., for tolerability issues or treatment failure) for prolonged periods (months). Subjects discontinuing a 744LA regimen may be at risk for developing DDIs many weeks after discontinuing injectable therapy. Of note, evidence to date indicates that significant DDIs with GSK1265744 and other antiretrovirals are unlikely to occur.	None needed at this time. All subjects will be informed of prohibited medications throughout the study and updates provided as needed to participants.

Potential Risk of Clinical Significance	Data/Rationale for Risk	Mitigation Strategy
Development of Resistance in the Setting of HIV Acquisition	Residual concentrations of GSK1265744 would remain in the systemic circulation of participants who stopped 744LA (e.g., for tolerability issues or treatment withdrawal) for prolonged periods (months). Participants discontinuing a 744LA regimen may be at risk for developing resistance to GSK1265744 many weeks after discontinuing injectable therapy were they to become newly infected with HIV.	The risk of HIV acquisition should be minimized in this study on the basis of inclusion of participants at lower risk for acquiring HIV. All participants will be provided HIV counseling and testing throughout the study, as well as provision of condoms and lubricant. Additionally, participants will be followed for 52 weeks from the time of the last injection with 744LA. Participants who acquire HIV on study will be referred for appropriate care.

APPENDIX XI: TOXICITY MANAGEMENT

Toxicity Management General Guidance

In general, the IoR has the discretion to hold study product at any time if she/he feels that continued product use would be harmful to the participant, or interfere with treatment deemed clinically necessary according to the judgment of the IoR. In addition, a Clinical Management Committee (CMC) will be established for this study. The CMC's responsibilities will include consultation and decision-making regarding management of toxicities and study product administration, including product resumption following the occurrence of certain types of toxicities and/or permanent discontinuation. Throughout this appendix are examples of AEs that require consultation with the CMC; in all such cases, the CMC should be notified as soon as possible and ideally within 72 hours of site awareness of the AE in question. Investigators also should consult the CMC for further guidance in restarting study product or progressing to permanent discontinuation. Revealing a participant's blinded status will occur only for individuals who become HIV infected and choose to initiate antiretroviral therapy. (A full description of the CMC's role and responsibilities is included in the SSP Manual).

The following general guidance refers to all AEs except for AST/ALT. Refer to the section below "Specific Guidance on Transitioning from Oral to Injectable Phase", as well as to the table below for specific guidance for AST/ALT.

<u>Grade 1 or 2</u>

In general, participants who develop a Grade 1 or 2 AE regardless of relatedness to study product, and that is not specifically addressed in the Table below may continue use of the study product per protocol. See an exception to this under "Specific Guidance on Transitioning from Oral to Injectable Phase".

<u>Grade 3</u>

Participants who develop a Grade 3 AE or toxicity that is not specifically addressed in the Table below and is judged to be related to study product by the Investigator, study product use should be temporarily discontinued in consultation with the CMC. In general, and unless otherwise decided in consultation with the CMC, the investigator should re-evaluate the participant until resolution of the toxicity. The study product should be permanently discontinued if improvement to severity ≤ Grade 2 cannot be documented within 4 weeks of receipt of the initial result. If study product is resumed and the same Grade 3 AE recurs at any time, the Investigator must consult the CMC for further guidance on holding of study product, frequency of reevaluation or progression to permanent discontinuation of the study product.

<u>Grade 4</u>

Participants who develop a Grade 4 AE or toxicity that is not specifically addressed below (regardless of relationship to study product) should have the study product temporarily discontinued. The Investigator must consult the CMC and continue the temporary study product hold until a recommendation is obtained from the CMC. In general, study product use will not be resumed if the Grade 4 AE is considered related to study product use. If, in consultation with the CMC, study product use is resumed and the same Grade 4 AE recurs at Grade 4 level at any time, study product must then be permanently discontinued.

Specific Guidance on Transitioning from Oral to Injectable Phase

A Grade 1 AST/ALT abnormality reported at Week 2, regardless of relatedness to the study product, should be confirmed within one week. Study drug may continue until the confirmatory results are available. If the repeat value is < Grade 1, study drug may continue to Week 4. If the repeat value is Grade 1 or higher, study product should be stopped, and the participant will be prohibited from entering the injection phase of the study, and will be terminated from the study. All such cases must be reported to the CMC. In addition, participants will be followed with weekly AST/ALT assessments until they return to < Grade 1.

A Grade 2 or higher AST/ALT abnormality reported at Week 2 of the oral phase, regardless of relatedness to the study product, will prohibit a participant from entering the injection phase of the study, and the participant will be terminated from the study. All such cases must be reported to the CMC. In addition, participants will be followed with weekly AST/ALT assessments until they return to < Grade 1.

Excluding AST and ALT, any grade 3 clinical or laboratory AE observed prior to Week 5 will prompt consultation with the CMC prior to any injectable dosing. Excluding AST and ALT, Grade 3 adverse events deemed after CMC consultation to clearly not be related to study product may be permissible to continue to injectable dosing provided

a) Documented resolution to ≤ Grade 1 prior to injectable dosing, and
b) Approval of the CMC.

Grade 3 adverse events deemed related to study product, or a Grade 3 AST or ALT, or any Grade 4 adverse event will lead to study discontinuation prior to injectable dosing. AEs will be followed until resolution in consultation with the CMC.

General Criteria for Discontinuation of Study Product

Participants may voluntarily discontinue the study products for any reason at any time. Investigators will permanently discontinue participants from study product per protocol for any of the specific criteria below, which may be further clarified in the SSP Manual. Investigators also may permanently discontinue participants for reasons not shown here or in the SSP Manual (e.g., to protect participants' safety and/or if participants are unable or unwilling to comply with study product use procedures). In such cases, the IoR or designee must first query the CMC for review. The CMC will provide a written response to the site indicating whether the CMC has recommended permanent discontinuation of study product based on careful review of all relevant data. It should be noted that the safety of the participant should always take precedence, and therefore, an IoR may determine that study product be permanently discontinued before the CMC has time to respond. This is acceptable, and in such cases, the CMC should be notified as soon as possible regarding the nature of the case and the course of action taken.

The criteria for permanent discontinuation of study product use for an individual participant are:

- Study product-related toxicity requiring permanent discontinuation of study product per the guidelines above and below
- Completion of regimen as defined in the protocol
- Request by participant to terminate study product
- Clinical reasons determined by the IoR
- Acquires HIV infection or hepatitis B virus (HBV) infection

- Pregnancy or expresses a desire to become pregnant

Participants in the injection phase who prematurely and permanently discontinue study product should be asked to continue to be followed according to the applicable Schedule of Evaluations and Procedures (Appendices I - VIII).

Study product will be temporarily withheld from a participant for any of the following reasons:

- Report of use of prohibited concomitant medications as described in the SSP Manual. Study product use may resume upon consultation with the CMC and when the participant reports that he/she is no longer taking the prohibited medication, provided other reasons for temporary study product hold/permanent discontinuation do not apply. The CMC should be consulted in all cases where a participant reports taking a prohibited product during the course of the study.

- The participant is unable or unwilling to comply with required study procedures such as HIV testing and routine laboratory assessments, or otherwise might be put at undue risk to their safety and well-being by continuing study product use, according to the judgment of the IoR. The IoR must consult the CMC on all temporary study product holds instituted for this reason for further guidance on resuming study product use, continuing the temporary hold, or progressing to permanent discontinuation.

- The participant has one or more reactive HIV test results, or expresses a concern about having acute HIV infection. Criteria for resuming use of study product in these circumstances are defined in the SSP Manual and Section 5.21 of the protocol.

Participants who temporarily or permanently discontinue study product during the oral phase will be instructed to return all study products as soon as possible.

Guidance on Toxicity Management for Specified Toxicities:

Nausea, Vomiting, and Diarrhea

CONDITION AND SEVERITY[1]	IMMEDIATE ACTION	FOLLOW-UP AND MANAGEMENT[2]
**Nausea, Vomiting, and Diarrhea**		
Grade 1 and 2	Continue study product (reminder to take study product with food)	Treat symptomatically with hydration, oral antiemetic therapies or antiemetic suppositories at the discretion of the Investigator. The Investigator should order any clinically relevant laboratory analyses (per judgment of the Investigator).
Grade ≥ 3	Discontinue study product temporarily	Participants with Grade ≥ 3 nausea, vomiting, or diarrhea, for which an alternative etiology is not established, must discontinue the study product temporarily until Grade 2 or lower and be treated symptomatically. Should condition(s) not improve to Grade ≤ 2 within 7 days, the Investigator should consult the CMC for guidance on continuing the temporary discontinuation or progressing to permanent discontinuation of study product.

Guidance on Toxicity Management for Specified Toxicities:

AST/ALT

<table>
<tr><th>CONDITION AND SEVERITY[1]</th><th>STUDY PRODUCT USE</th><th>FOLLOW-UP AND MANAGEMENT[2]</th></tr>
<tr><td colspan="3">ELEVATIONS in AST/ALT
(New clinical finding or increase from baseline clinical finding only)</td></tr>
<tr><td>Grade 1 and higher</td><td colspan="2"><u>Oral phase</u>: A Grade 1 AST/ALT abnormality reported at Week 2, regardless of relatedness to the study product, should be confirmed within one week. Study drug may continue until the confirmatory results are available. If the repeat value is < Grade 1, study drug may continue to Week 4. If the repeat value is Grade 1 or higher, study product should be stopped, and the participant will be prohibited from entering the injection phase of the study, and will be terminated from the study. All such cases must be reported to the CMC. In addition, participants will be followed with weekly AST/ALT assessments until they return to < Grade 1.

A Grade 2 or higher AST/ALT abnormality reported at Week 2 of the oral phase, regardless of relatedness to the study product, will prohibit a participant from entering the injection phase of the study, and the participant will be terminated from the study. All such cases must be reported to the CMC. In addition, participants will be followed with weekly AST/ALT assessments until they return to < Grade 1

<u>Injection phase</u>: The CMC should be notified as soon as possible. For a Grade 1 AST/ALT, the CMC will determine whether further injections may be given in cases where levels are < Grade 1 prior to the next scheduled injection. Unless otherwise specified by the CMC, for Grade 1 and 2 AST/ALT, repeat testing should be performed weekly until levels are < Grade 1. For Grade 2 and higher AST/ALT, study product will be permanently discontinued. For Grade 3 and 4 AST/ALT, repeat testing should be performed as soon as possible, and participants should be followed weekly until levels are < Grade 1. Participants who are permanently discontinued from stud product should continue to be followed on study/off study product, following the appropriate Schedule of Evaluations (based on number of injections received).

Note the following for cases of exercise-induced CK abnormalities: Cases of CK abnormality, presumed attributable to exercise, at the Grade 3 or higher level accompanied by ALT/AST abnormalities of Grade 3 or lower should be brought to the attention of the CMC for adjudication of further management and further administration of study product. Grade 4 elevations in ALT or AST, regardless of CK elevation will prompt permanent discontinuation of study product. All such cases must be reported to the CMC.</td></tr>
</table>

Guidance on Toxicity Management for Specified Toxicities:

Note for all grades: All study participants will be negative for HBsAg at study entry, and participants who enter the study without evidence of immunity to HBV will be referred for HBV vaccination. Therefore, pre-existing HBV infection is not likely to be a cause of AST/ALT elevations.

Careful assessments should be done to rule out the use of alcohol, lactic acidosis syndrome, non-study medication-related product toxicity, herbal medications/supplements, or viral hepatitis as the cause of elevation in AST or ALT of any grade. The participant must be carefully assessed for any symptoms or signs of hepatotoxicity, including fatigue, malaise, anorexia and nausea, jaundice, acholic stools, right upper quadrant pain, or hepatomegaly. If the AST/ALT elevation is considered most likely to be due to concomitant illness or medication, standard management, including discontinuation of the likely causative agent, if possible, should be undertaken. If symptoms or signs of clinical hepatitis are present, study product must be held or discontinued. In addition, all participants with elevated values should be considered for testing for Hepatitis A, B, and C infection.

Guidance on Toxicity Management for Specified Toxicities:

Creatinine Clearance

CONDITION AND SEVERITY[1]	STUDY PRODUCT USE	FOLLOW-UP AND MANAGEMENT[2]
**CREATININE CLEARANCE**		
Estimated CrCl< 50 mL/min	Discontinue study product temporarily	If the calculated creatinine clearance is <50mL/min, it should be confirmed within 1 week of the receipt of the results, and the CMC should be consulted
Confirmed CrCl< 50 mL/min	Permanently discontinue study product	If the calculated creatinine clearance is confirmed to be <50 mL/min, the study product must be permanently discontinued and the CMC notified. Participants who fail to have a confirmed test within 2 weeks of receiving the initial result, should be permanently discontinued from use of the study product and the CMC is notified.
Re-testing result is ≥50 mL/min	Consult CMC for guidance	If re-testing yields a result ≥ 50 mL/min, the Investigator must consult the CMC for further guidance on resuming study product use, continuing the hold temporarily, or progressing to permanent discontinuation.<br>If the investigator in consultation with the CMC has determined that the case has stabilized, it may be possible to decrease the frequency of follow-up laboratory testing in addition to resumption of study product.

Guidance on Toxicity Management for Specified Toxicities:

Creatine Phosphokinase (CK or CPK)

CONDITION AND SEVERITY[1]	STUDY PRODUCT USE	FOLLOW-UP AND MANAGEMENT[2]
**Creatine Phosphokinase**		
Grade 3	Continue study product until repeat test results are available	A Grade 3 or higher elevation in CPK should result in a repeat assessment within 2-4 weeks to ensure the result is transient or due to exercise and will not require a change in study treatment. A history regarding use of products known to cause increase of CPK (such as statins) physical activity or exercise preceding the CPK evaluation should be obtained.
Grade 4	Continue study product until repeat test results are available if the elevation is thought to be possibly or probably related to study product.	Grade 4 elevations in CPK should have a repeat assessment after the subject has abstained from exercise for >24 hours. For persistent Grade 4 CPK elevations that are considered possibly or probably related to the study product, study product should be discontinued and the participant should be followed on study/off study product.<br><br>(See section on ALT/AST abnormalities for important notes on ALT/AST abnormalities accompanying CK elevations)

Guidance on Toxicity Management for Specified Toxicities:

QTc – Criteria for Permanent Discontinuation of Study Product

A participant that meets either criterion below will have study product stopped, but will remain in study follow-up. The QT correction formula used to determine study product discontinuation should be the same one used throughout the study.

- QTcB > 500 msec, OR
- Change from baseline: QTcB >60 msec

Study product discontinuation decisions are to be determined by the Investigator of Record or designee in consultation with the CMC. A decision to permanently discontinue study product will be based on an average QTc value of triplicate ECGs. If an ECG demonstrates a prolonged QT interval, obtain two more ECGs within one hour, and then use the averaged QTc values of the three ECGs to determine whether the participant should permanently discontinue study product.

Guidance for Injection Site Reactions (ISRs)

The CMC must be informed of all Grade 3 or 4 ISRs to determine etiology and assess appropriate continued study participation. ISR discomfort can be managed symptomatically (e.g. cold/warm compress, acetaminophen, ibuprofen) if the reaction is interfering with the participant's ability to perform activities of daily living.

Guidance for Allergic Reactions

Participants may continue to receive oral or injectable study product for Grade 1 or 2 allergic reactions at the discretion of the IoR or designee. The participant should be advised to contact the study site staff immediately if there is any worsening of symptoms or if further systemic signs or symptoms develop. Antihistamines, topical corticosteroids, or antipruritic agents may be prescribed.

Participants with Grade ≥3 allergic reactions that are considered to be related to study product should permanently discontinue study product and continue to be followed on study/off study product. Participants should be treated as clinically appropriate and followed until resolution of the AE.

APPENDIX XII: SAMPLE SCREENING AND ENROLLMENT INFORMED CONSENT FORM FOR PARTICIPANTS ENROLLING UNDER VERSION 3.0

A Phase IIa Study to Evaluate the Safety, Tolerability and Pharmacokinetics of the Investigational Injectable HIV Integrase Inhibitor, GSK1265744, in HIV-uninfected Men and Women

(HPTN 077)

Version 3.0
October 13, 2015
DAIDS Document ID: 11964

Sponsored by: Division of AIDS, United States (US) National Institute of Allergy and Infectious Diseases, US National Institutes of Health. Study products are provided by ViiV Healthcare

PRINCIPAL INVESTIGATOR: *[Insert Name]*

PHONE: [Insert Number**]**

Introduction

You are being asked to take part in an investigational research study related to the safety of a new drug that is hoped to be used one day to treat or prevent Human Immunodeficiency Virus, or HIV. HIV is the virus that causes Acquired Immunodeficiency Syndrome, or AIDS.

This study is being offered to approximately 194 men and women in Africa, and North and South America. Before you decide whether to join the study, we would like to explain the purpose of the study, the risks and benefits to you, and what is expected of you.

A description of this study will be available on www.ClinicalTrials.gov, as required by US law. This web site will not include information that can identify you. At most, the web site will include a summary of the results. You can search this web site at any time.

There may be no direct benefits for you if you participate in this study. There also may be some risks with taking part in the study. Before you can make an informed decision about whether to take part in this study, you should understand the possible risks and potential benefits of being in this study. This informed consent form gives information about the study that will be discussed with you. Once you understand the study, and if you agree to take part, you will be asked to sign your name on this form.

Your participation is voluntary

This consent form gives information about the study that will be discussed with you. Once you understand the study, and if you agree to take part, you will be asked to sign your name or make your mark on this form. You will be offered a copy of this form to keep.

Before you learn about the study, it is important that you know the following:

- Your participation is voluntary. You do not have to take part in any of the tests or procedures in the study.

- You may decide not to take part in the study, or you may decide to leave the study at any time. You will continue to receive the same services that you can get at [insert clinic name].
- If you decide not to take part in the study, you can still join another study at a later time if there is one available and you qualify.
- You cannot join this study if you are taking part in another study of drugs or medical devices. You are asked to tell the study staff about any other studies you are taking part in or thinking of taking part in. This is very important for your safety.
- If you are a woman and are pregnant or breastfeeding, or wish to become pregnant during the course of the study, you are not eligible to be in this study.

Purpose of the study

The purpose of this study is to find out whether a new drug is safe. The drug is being developed for both the treatment and prevention of HIV. The drug being used in this study has not been approved for use by the US Food and Drug Administration (FDA), which is the part of the US government that approves medicines. The drug is called GSK 1265744 (GSK 744 for short), and it is a type of drug called an antiretroviral drug. Antiretroviral drugs usually come in the form of pills, but newer kinds are becoming available as an injection, or a shot.

In this study, we want to find out whether the pill and injection form of the drug is safe, as well as how people who get the drug respond to it (meaning, does it make you feel sick). Another reason we are doing this study is to find out whether people in the study are willing to receive the injections (shots) of the new drug and how they feel about it. .

You are being asked to participate in this study because you are at very low risk of getting HIV. We do not know whether the drug being used in this study will prevent a person from getting HIV, and that is why we need volunteers who have little to no risk of getting HIV. [*If required or desired, sites to include here or other section as appropriate the protocol and site-specific risk criteria*].

Even though you are at low risk, we have to remind you every time we see you for your study visits that one of the best things that you can do to protect yourself against getting HIV during sex is to use a condom every time you have sex.

We will also ask you to let us know if your risk for getting HIV has changed while you are in the study, and also to let us know whether you think that you may have been exposed to HIV through sex or intravenous drugs.

If you think you are at risk for getting HIV, you should not participate in this study.

STUDY GROUPS

There are two study groups in this study. Each group has two arms. The first group (Group 1) has already been enrolled in the study. If you decide to take part in the study, you will be enrolled into the second study group (Group 2).

The groups and arms look like this:

- Group 1
  - Arm 1: People in this arm get pills and shots that have the GSK 744 drug in it, and people get shots every 12 weeks, at 3 time points.

- Arm 2: People in this arm get pills and shots at the same time points as above, but they do not have the GSK 744 drug in it. This is called a placebo. The placebo forms of the drug typically look like the active or "real" drug, but they do not have the drug or any other medicine in them.

  There are approximately 106 people enrolled into Group 1.

- Group 2

  - Arm 1: People in this arm get pills and shots that have the GSK 744 drug in it. People get a shot at 5 time points – the first two are 4 weeks apart and the next 3 are every 8 weeks. It is given as one shot in the buttock.

  - Arm 2: This arm gets the placebo pills and shots at the same time points as above.

  There will be approximately 88 people in Group 2.

Reason For the Two Groups

Recently, the ÉCLAIR study, a very similar study to this one, got some results. ÉCLAIR is studying over 100 men in the United States. These men got GSK 744 the same way as Group 1 in this study. So these men got the drug every 12 weeks, at 3 time points. ÉCLAIR showed that the drug did not last in the blood for the full 12 weeks. Since we want to see if this drug can protect people from getting HIV, we need to find out how long this rug lasts in between shots. We need this information to see if people can stay protected. That is why we have Group 2 in HPTN 077. We want to study a different dose of the drug and give it at a different length of time between shots. The group that we are asking you to enroll in will get a shot of 600 mg of GSK 744 at two time points 4 weeks apart, and then at 3 time points 8 weeks apart (Week 5 ,9, 17, 25, ands 33). The amount of drug is smaller in Group 2 than Group 1 (600 mg instead of 800 mg). The amount is smaller since the shots occur closer together in time in Group 1 than they do in Group 2. Whether you get the real drug or the placebo is chosen randomly, like flipping a coin. You cannot choose which one you get and the study staff cannot choose it for you.

No matter what group you are in, three out of four people (or 75%) will get pills and shots that have the GSK 744 drug in it. One out of four people (or 25%) will get the placebo, which does not have the GSK 744 drug in it. [*Sites may insert another way to describe the 3:1 randomization that may be more appropriate to the local population]*.

The study staff and you will not know whether you got the real drug or placebo until a planned time later during the study. When that time is reached, we will let everyone know which one they got. Your participation in the study may have ended by the time, but we will contact you to let you know.

If you did not receive the GSK 744 drug and you are still in the study when we let everyone know what they got, that will be the time that your participation in the study will end. Even if you did not get the GSK 744 drug, your participation is very important because it gives us a chance to compare what happens to you with a person that did get the drug.

STUDY PROCEDURES

If you decide to join the study, you will be asked to come to this clinic up to 22 times over the course of up to two years.

Screening Visit

Your screening visit may occur after you read, discuss, understand, and sign this form, or will we schedule it for you at another time. We will help you understand the form and answer your questions before you sign this form. The procedures done for the screening visit will take about [*site to fill in time required*].

At this visit, the study staff will:

- Ask you where you live and other questions about you, your medical health and if you are taking any medicines, your sexual practices, and whether you use alcohol or drugs for eligibility purposes.
- Give you a brief physical exam to make sure you are healthy.
- Talk with you about HIV and ways to protect yourself from getting it and offer condoms and lubricant.
- We will ask you to have an electrocardiogram (ECG) scan, which is a test to monitor your heart.
- Test for three sexually transmitted infections called gonorrhea, chlamydia, and syphilis. For gonorrhea and chlamydia, this will be done with urine if you are a man, or urine or a swab of your vagina or cervix if you are a woman. Blood will be collected for syphilis testing.
- For women of childbearing potential: Collect XX mL of urine for pregnancy testing.
- Collect ~XX mL (about x teaspoons) of blood for HIV testing, hepatitis B testing, hepatitis C testing, syphilis, to check your general health, to check the health of your liver, and for storage for study-related testing and long-term storage (if you provide consent).

The results of the HIV test will be available [*site to insert timeframe of RNA testing, and also EIA testing if being used*]. You will be contacted about the results of your other tests when they are available. If you have gonorrhea, chlamydia, or syphilis, you will not be eligible for the study and you will be referred for treatment. A small amount of blood will be stored from this visit. No other samples collected at the time of screening will be kept or used for any other tests other than those listed above.

Confirmation of Eligibility:

Once all the results of the screening tests are known, the following will happen within 45 days after screening:

- You will be told your test results and what they mean.
- If you have a positive HIV test or a positive test for hepatitis B infection, you will not be eligible for the study, and you will be referred for the appropriate medical care (*sites to add specifics about this here as necessary*).
- If you are negative for both HIV, hepatitis B infection, but the results from the other blood or urine tests show that you might have some health problems, or that you may be pregnant, you may not be eligible for the study. Study staff will refer you to available sources of medical care and other services you may need. Later, if these problems resolve, you can come back to find out if you are eligible at that time.
- You will be given referrals for other health services if you need them.

Enrollment Visit (Week 0)

If you are eligible for this study and decide to take part in the study, you will be asked to return for the enrollment visit. This visit will last about xx hours. During the visit, the study staff will:

- Confirm where you live and how to contact you.
- Ask you some questions about yourself, like your age, and your ethnic group.
- Talk with you about HIV and ways to protect yourself from getting it.
- Give you a complete physical exam, to include measuring your height, weight, temperature, blood pressure, and ask you about any other medicines you are taking.
- Perform an ECG scan.
- For women of childbearing potential: Collect ~XX mL of urine for pregnancy testing.
- Test for gonorrhea and chlamydia by using a swab of your rectum. *[US sites opting to perform this testing at screening to remove this from here and place in screening section]*.
- Collect a urine sample to see if there is sugar or protein in your urine.
    - Collect ~XX mL (about x teaspoons) of blood for: HIV testing, to check how much cholesterol is in your blood (a fatty substance in your blood), to check your general health, to check the health of your liver, and for storage for study-related testing and long-term storage (if you provide consent). [*Sites that opt to collect samples for Pharmcogenomic testing to add this language here:* Additionally, if you provide consent, we will use a sample of your blood to see how the drugs work in your body by looking at your genes. Information regarding the testing related to your genes is found later in this consent form.] For the cholesterol test, you will be instructed to not eat or drink anything other than what the study staff tell you is acceptable for 8-12 hours before your blood is drawn.
- Ask you questions about your sexual behavior.
- Ask you questions about your opinions about taking pills and getting injections, using other products, as well as your opinions about being in a study.
- Randomize you into one of the two study groups.
- Give you your study pills, and explain how to take them, and any side effects they may cause.
- Give you the results of your blood tests when they are available.
- Give you condoms and lubricants.

Weeks 2 and 4 Visits

This visit will last about xx hours. During this visit, the study staff will**:**

- Confirm where you live and how to contact you.
- Give you a brief physical exam, ask you if you have experienced any side effects from the study product, and ask you about any other medicines you are taking. During the Week 4 visit, we will also remind you about certain drugs that you should not take a week before your injection.

- Ask you to have an ECG scan (Week 4 only).
- Collect ~XX mL (about x teaspoons) of blood for HIV testing, to check your general health, the health of your liver, the amount of study product in you, and for storage for study-related testing and long-term storage (if you provide consent).
- For women of childbearing potential: Collect ~XX mL of urine for pregnancy testing.
- Talk with you about HIV and ways to protect yourself from getting it and count your pills.
- Ask you whether you have questions about taking your study pills. (Week 2 only)
- Give you the results of your blood tests when they are available.
- Give you condoms and lubricant.

Week 5 Visit

This visit will last xx hours. During this visit, the study staff will**:**

- Confirm where you live and how to contact you.
- Give you a brief physical exam, ask you if you have experienced any side effects from the study product, and ask you about any other medicines you are taking. We will also remind you about certain drugs that you should not take for one week after the injection.
- Talk with you about HIV and ways to protect yourself from getting it.
- For women of childbearing potential: Collect ~XX mL of urine for pregnancy testing.
- Collect ~XX mL (about x teaspoons) of blood for HIV testing, to check your general health, the health of your liver, the amount of study product in you, and for storage for study-related testing and long-term storage (if you provide consent).
- Ask you questions about your sexual behavior.
- Administer the a shot (your clinician will discuss the injection site with you). Give you the results of your blood tests when they are available.
- Give you condoms and lubricants.

Week 6 Visit

This visit will last up to xx hours. During this visit, the study staff will:

- Confirm where you live and how to contact you.
- Talk with you about HIV and ways to protect yourself from getting it.
- Give you a brief physical exam, ask you if you have experienced any side effects from the shots you received, and ask you about any other medicines you are taking. At the Week 13 visit, we will remind you about certain drugs that you should not take one week before your injection.
- Ask you to have an ECG scan.

- Collect ~XX mL (about x teaspoons) of blood for HIV testing, to check your general health, the health of your liver, the amount of study product in you, and for storage for study-related testing and long-term storage (if you provide consent).
- Ask you questions about the shots you received at the previous visit.
- Give you the results of your blood tests when they are available.
- Give you condoms and lubricant.

Week 9 Visit

This visit will last about xx hours. During this visit, the study staff will:

- Confirm where you live and how to contact you.
- Talk with you about HIV and ways to protect yourself from getting it.
- Give you a brief physical exam, ask you if you have experienced any side effects from the shots you received, and ask you about any other medicines you are taking. We will also remind you about certain drugs that you should not take for one week after the injection.
- For women of childbearing potential: Collect ~XX mL of urine for pregnancy testing.
- Collect ~XX mL (about x teaspoons) of blood for HIV testing, to check your general health, the health of your liver, the amount of study product in you, and for storage for study-related testing and long-term storage (if you provide consent).
- Ask you questions about your sexual behavior.
- Administer the shot (your clinician will discuss the injection site with you).
- Give you the results of your blood tests when they are available.
- Give you condoms and lubricant.

Weeks 10 and 13 Visits

These visits will last up to xx hours. During these visits, the study staff will:

- Confirm where you live and how to contact you.
- Talk with you about HIV and ways to protect yourself from getting it.
- Give you a brief physical exam, ask you if you have experienced any side effects from the shots you received, and ask you about any other medicines you are taking. At the Week 23 visit, we will also remind you about certain drugs that you should not take one week before your next injection.
- Ask you to have an ECG scan (Week 13 only).
- Collect ~XX mL (about x teaspoons) of blood for HIV testing, to check your general health, the health of your liver, the amount of study product in you, and for storage for study-related testing and long-term storage (if you provide consent).

- Ask you questions about the shot you received at the previous visit (Week 10 only).
- Give you condoms and lubricant.

Week 17 Visit

This visit will last about xx hour. During this visit, the study staff will**:**

- Confirm where you live and how to contact you.
- Give you a brief physical exam, ask you if you have experienced any side effects from the shot you received, and ask you about any other medicines you are taking. We will also remind you about certain drugs that you should not take one week after the injection.
- Talk with you about HIV and ways to protect yourself from getting it.
- For women of childbearing potential: Collect ~XX mL of urine for pregnancy testing.
- Collect ~XX mL (about x teaspoons) of blood for HIV testing, to check your general health, the health of your liver, the amount of study product in you, and for storage for study-related testing and long-term storage (if you provide consent).
- Ask you questions about your sexual behavior.
- Administer a shot (your clinician will discuss the injection site with you). Give you the results of your blood tests when they are available.
- Give you condoms and lubricant.

Weeks 18 and 21 Visits

These visits will last up to xx hours. During these visits, the study staff will:

- Confirm where you live and how to contact you.
- Talk with you about HIV and ways to protect yourself from getting it.
- Give you a brief physical exam, ask you if you have experienced any side effects from the shot you received, and ask you about any other medicines you are taking.
- Ask you to have an ECG scan (Week 21 only).
- Collect ~XX mL (about x teaspoons) of blood for HIV testing, to check your general health, the health of your liver, the amount of study product in you, and for storage for study-related testing and long-term storage (if you provide consent).
- Ask you about the shot you received at the previous visit (Week 18 only).
- Give you condoms and lubricant.

Week 25 Visit

This visit will last xx hours. During this visit, the study staff will**:**

- Confirm where you live and how to contact you.
- Give you a brief physical exam, ask you if you have experienced any side effects from the study product, and ask you about any other medicines you are taking. We will also remind you about certain drugs that you should not take for one week after the injection.
- Talk with you about HIV and ways to protect yourself from getting it.
- For women of childbearing potential: Collect ~XX mL of urine for pregnancy testing.
- Collect ~XX mL (about x teaspoons) of blood for HIV testing, to check your general health, the health of your liver, the amount of study product in you, and for storage for study-related testing and long-term storage (if you provide consent).
- Test you for three sexually transmitted infections called gonorrhea, chlamydia, and syphilis. For gonorrhea and chlamydia, this will be done with urine if you are a man.   If you are a woman, this will be done with urine or a swab of your vagina or cervix. For men and women we will also test using a swab of your rectum. Blood will be collected for syphilis testing.
- Ask you questions about your sexual behavior.
- Administer the shot (your clinician will discuss the injection site with you).
- Give you the results of your blood tests when they are available.
- Give you condoms and lubricants.

Weeks 26 and 29 Visits

These visits will last up to xx hours. During these visits, the study staff will:

- Confirm where you live and how to contact you.
- Talk with you about HIV and ways to protect yourself from getting it.
- Give you a brief physical exam, ask you if you have experienced any side effects from the shot you received, and ask you about any other medicines you are taking.
- Ask you to have an ECG scan (Week 29 only).
- Collect ~XX mL (about x teaspoons) of blood for HIV testing, to check your general health, the health of your liver, the amount of study product in you, and for storage for study-related testing and long-term storage (if you provide consent).
- Ask you about the shot you received at the previous visit (Week 26 only).
- Give you condoms and lubricant.

Week 33 Visit

This visit will last about xx hour. During this visit, the study staff will**:**

- Confirm where you live and how to contact you.
- Give you a brief physical exam, ask you if you have experienced any side effects from the shot you received, and ask you about any other medicines you are taking. We will also remind you about certain drugs that you should not take one week after the injection.
- Talk with you about HIV and ways to protect yourself from getting it.
- For women of childbearing potential: Collect ~XX mL of urine for pregnancy testing.
- Collect ~XX mL (about x teaspoons) of blood for HIV testing, to check your general health, the health of your liver, the amount of study product in you, and for storage for study-related testing and long-term storage (if you provide consent).
- Ask you questions about your sexual behavior.
- Administer a shot (your clinician will discuss the injection site with you). Give you the results of your blood tests when they are available.
- Give you condoms and lubricant.

Weeks 34 and 37 Visits

These visits will last up to xx hours. During these visits, the study staff will:

- Confirm where you live and how to contact you.
- Talk with you about HIV and ways to protect yourself from getting it.
- Give you a brief physical exam, ask you if you have experienced any side effects from the shot you received, and ask you about any other medicines you are taking.
- Ask you to have an ECG scan (Week 37 only).
- Collect ~XX mL (about x teaspoons) of blood for HIV testing, to check your general health, the health of your liver, the amount of study product in you, and for storage for study-related testing and long-term storage (if you provide consent).
- Ask you about the shot you received at the previous visit (Week 34 only).
- Give you condoms and lubricant.

Week 41 Visit

These visits will last up to xx hours. During these visits, the study staff will:

- Confirm where you live and how to contact you.
- Talk with you about HIV and ways to protect yourself from getting it.
- Give you a brief physical exam, ask you if you have experienced any side effects from the shots you received, and ask you about any other medicines you are taking.
- For women of childbearing potential: Collect ~XX mL of urine for pregnancy testing.
- Collect a urine sample to see if there is sugar or protein in your urine.
- Collect ~XX mL (about x teaspoons) of blood for HIV testing, to check your general health, to check how much cholesterol is in your blood (a fatty substance in your blood), the health of your liver, the amount of study drug is in your blood, and for storage for study-related testing and long-term storage (if you provide consent).
- Ask you questions about your sexual behavior.
- Give you condoms and lubricant.

Weeks 53, 65, and 77 Visits

These visits will last up to xx hours. During these visits, the study staff will:

- Confirm where you live and how to contact you.
- Talk with you about HIV and ways to protect yourself from getting it.
- Give you a brief physical exam, ask you if you have experienced any side effects from the shots you received, and ask you about any other medicines you are taking.
- For women of childbearing potential: Collect ~XX mL of urine for pregnancy testing.
- Collect ~XX mL (about x teaspoons) of blood for HIV testing, to check the health of your liver, the amount of study drug in you, and for storage for study-related testing and long-term storage (if you provide consent).
- At Week 53 only, we will test for three sexually transmitted infections called gonorrhea, chlamydia, and syphilis. For gonorrhea and chlamydia, this will be done with urine if you are a man. If you are a woman, this will be done with urine or a swab of your vagina or cervix. For men and women we will also test using a swab of your rectum. Blood will be collected for syphilis testing.
- Ask you questions about your sexual behavior.
- Give you condoms and lubricant.

Week 85, Final Visit

These visits will last up to xx hours. During this visit, the study staff will:

- Talk with you about HIV and ways to protect yourself from getting it.
- Give you a brief physical exam, ask you if you have experienced any side effects from the study shots you received, and ask you about any other medicines you are taking.
- For women of childbearing potential: Collect ~XX mL of urine for pregnancy testing.
- Collect ~XX mL (about x teaspoons) of blood for HIV testing, to check the health of your liver, the amount of product in you, and for storage for study-related testing and long-term storage (if you provide consent).
- Give you condoms and lubricants.

Permanently Stopping Your Study Product If you permanently stop being on the study drug during the study for any reason, we may ask you to continue to come for your visits, but on a modified schedule. We will fully explain to you what will be expected if you permanently stop getting the study drug.

Sample Storage

Each time blood is taken during a study visit, we will test some of it here in our laboratory at the site, and some of it will be stored for testing at designated laboratories in the United States. [*Non-US sites to add*: Swabs or other samples may also be sent for STI testing at designated laboratories in the United States].

POSSIBLE FUTURE TESTS [*Sites that are able to do this per country or IRB regulations to keep this included; otherwise, this should be removed, as well as the signature line for it on the signature page]*

Some of the blood that you give during this study may be left over after all of the study tests are completed. We would like to keep this blood for an indefinite amount of time for future testing that may be unrelated to this study, but still related to HIV. You will be asked to sign at the end of this consent form to give permission for this. This blood will be stored and tested at designated laboratories in the United States. Even if you do not give permission to store your blood after the study, you can still be in this study. You may also withdraw your consent for specimen storage at any time.

RISKS AND/OR DISCOMFORTS

Study Medications

The drug being used in this study is currently being used in other people participating in similar studies. Those studies have reported side effects of the study drug, such as headaches, dizziness, upset stomach, rash, liver problems, fatigue, or injection site reaction (pain, irritation, skin redness, bumps, swelling, itching, bruising). Other reported side effects were nausea, stomach cramps, constipation, and right hand pain.

There have been some people who were taking this medicine in other studies who have had liver side effects. All of these people were HIV-infected (HIV positive) and they all had some damage to their liver before taking the GSK 744 study medication. In those studies, while taking the study medication, their blood tests showed that their liver was irritated, although they felt well. The medications were stopped, and the liver blood tests are normal. In HPTN 077, anyone with HIV-infection, Hepatitis C (or B), or any liver irritation will not be allowed to be in the study.

Some people who have had a prior history of seizures (epilepsy) have had seizures (spells) while taking GSK 744. One person who did not have a previous history of seizures died after prolonged seizures. If you have ever had a seizure you will not be allowed in the study. We will ask you about any neurologic symptoms during the study.

In studies done in pregnant rats and their newborns, using very high amounts of GSK 744, there were more baby rats that died when they were born or right after they were born. This did not happen to the baby rats when the mother got lower amounts of the drug, or no drug. The amount of GSK 744 that we are giving in this study is expected to be more than 7-times lower than the amount given to the pregnant rats. Birth defects have not been found in any animal studies of GSK 744 so far. We do not know whether what happened to the baby rats has any impact on what will happen in pregnant humans taking this drug. That is why if you are female and participating in this study, you cannot be pregnant, and we want you to take birth control the entire time you are in the study and also for a year after. If you become pregnant, we will not give you any more study medication and we will ask you to stay in the study so that we can follow you carefully.

We will update you on any new side effects that we see in this study and other on-going studies, if those side effects appear to have come from the drug. If you have questions concerning the additional study drug side effects, please ask the medical staff at your site. As stated above, some of these risks are seen in HIV infected people taking these medications. It is not known if these side effects will occur as often and it could be that some of these side effects might be more or less serious in HIV uninfected people.

There is a risk of local pain, bruising, swelling and rarely, an infection may occur at the injection site (where you got the shot).

The shots you receive in this study are long acting, meaning they stay in your body for a long time. One single shot can stay in your body for up to one year. If you develop a side effect to the study drug after the shot, there will be no way to remove the drug from your body. You will be in the study for up to 85 weeks. This is so that we can monitor your health for a year after your last injection.

If you develop a symptom from these drugs while the drugs are still in your body, every effort will be made to treat the side effects. The amount of drug will decrease overtime and will eventually disappear.

HIV Infection

If you test positive for HIV during the study you will be asked to stop taking your study medication, but will continue to be followed in the study. Because the study medication is itself being studied to be an HIV treatment medication, if you become HIV infected while taking the study medication, there is a chance that other drugs used to treat HIV infection might not work. This is called drug resistance.

To reduce the possibility of developing drug resistance, you will be asked to work with your local study clinic team to begin HIV treatment for at least one year after your last study medication injection. The

study will not provide this treatment but may be able to help you find and/or pay for that treatment for the one-year treatment period.

Pregnancy

As mentioned above, there are no studies of pregnant women taking the drug being used in this study. If you are pregnant, you are not eligible to be in this study.

If you can get pregnant and are engaging in sexual activity that could lead to pregnancy, you must agree to use a form of contraception (an IUD or hormone-based contraceptive) during the trial. If you stop being in the study during or right after the first part of the study, which is the part where you take the pills only, then you have to be on contraception for 30 days after taking your last pill. If you stay in the study and get injections, then you have to be on contraception for one year after your last shot.

If you express desire to become or become pregnant after joining the study, you will no longer receive the study product.

If you become pregnant during the first part of the study, which is the part where you take pills only, you will not get any shots, and we will follow you until your pregnancy is over, and then your participation in the study will end. If you become pregnant after you have had at least one shot, you will continue to be followed on study, but you will not get any more shots.

If you are still pregnant when the study ends, we will still contact you and/or review your medical records until the outcome of your pregnancy is known by site staff.

Not all contraceptive choices can prevent HIV transmission, and some may actually increase the risk of getting HIV. We will talk with you throughout the study about ways to protect yourself from getting HIV. You should also discuss with your health care provider and the study clinic staff ways to maintain effective contraception during your participation in the study.

Blood Draws

Taking blood samples may cause some pain, bruise your arm, or make you feel lightheaded. In rare cases you may faint. There is also a slight chance of infection when blood is drawn. You may be nervous while you are waiting for your HIV test result. If the tests show that you have HIV, you may worry about your health and future. You will receive counseling before and after the test to help address your concerns. We will make every effort to protect your confidentiality during the study. However, it is possible that others may learn that you are part of this study and they may think that you are infected with HIV or are at high risk for infection with HIV. Because of this you could have trouble finding or keeping a job. You could also have problems with your family, friends and community.

Rectal Swabs, and Vaginal/Cervical Swabs (for women only)

You may experience pain or discomfort form the rectal or vaginal/cervical swab. In some cases, you may have some bleeding.

Other Testing and Genetic Testing

[*Sites that are able to do this per country or IRB/EC regulations to keep this section included; otherwise, this should be removed, as well as the signature lines for it on the signature page*]

We want to look at your genes that affect how your body changes and removes the drug used in this study. Gene differences between people can lead to different amounts of drug in the body. This may affect how well a drug protects people from HIV infection. If you consent, we will test your blood to get information about how your genes may have affected the drug levels in your body. The tests we will use to look at your genes are research tests and will be performed in a research laboratory. All of the samples will be identified with a coded number. The laboratory doing the testing will not know who you are. The results obtained for individual study participants (like you) will not be reported to the study sites or back to you. However, the combined results of the testing for all of the study participants will be available to the study sites and to the study participants at their request, once the analysis has been completed.

We also may also use your samples and information for more complete genetic testing. For example, researchers may do "genome-wide association studies," also known as GWAS. A genome is all of your genes in total. Genes are made of even smaller "building blocks" that are arranged in a specific order. Many diseases can result from changes to this order or "sequence." These changes may somewhat explain why some people get diseases like cancer or diabetes while others do not. These changes may partly explain the body's response to disease and treatment. We are interested in understanding if the way the study medication interacts with your body has anything to do with your particular genes.

We may find information as we do this study that raises important other questions as to the safety of the study medication - - this new information may require that we do additional testing of left-over study samples. We will let you know right away if we find any new information or any new concerns develop about the medication as the study continues.

Your genetic information may also be shared for future research purposes and may be stored in a central genetic database, but your personal information (like your name or anything else about you) will not be shared.

ECG

ECG patches may cause a skin reaction such as redness or itching. You may also experience localized skin discomforts and/or hair loss associated with the placement of ECG leads.

Social

There may also be some social risks to participating in this study. You may feel embarrassed or uncomfortable with some of the questions you will be asked, some of the procedures that will be done, or some of the test results that you will receive. The questions we will ask you about your sexual behavior may make you feel uneasy. However, you do not have to answer any question that you do not want to and you can stop answering the questions at any time. You may also experience stigma as a result of being involved in a study about HIV because people may assume that you are HIV-infected. In addition, there may be uncommon or previously unknown risks that might occur. You should report any problems to the researchers immediately.

BENEFITS

We will test you for HIV and hepatitis B and C during this study. The counseling you get during this study may help you to avoid HIV and other sexually transmitted infections. If you have or become infected with HIV, this counseling may help you to learn how to better care for yourself and avoid passing HIV to your sexual partners. If you become HIV infected during the study, or have another

sexually transmitted infection, we will refer you for care and/or treatment as described earlier, and you will continue to be followed in the study. At the screening visit we will also check if you have hepatitis B infection. If needed, we will refer you for hepatitis B vaccination. During the study you will have tests to check on the health of your blood, and liver. If any health problems are found, you will be referred for care. At every visit you will receive condoms and lubricant free of charge.

You may not receive any other direct benefit from being in this study; however, you or others in your community may benefit from this study later. The information gathered during this study may help to prevent the spread of HIV. This may be beneficial to you and your community.

NEW INFORMATION

You will be told any new information learned during this study that might affect your willingness to stay in the study. For example, if information becomes available that shows that the medication may be causing bad effects, you will be told about this. You will also be told when the results of the study may be available, and how to learn about them.

WHY YOU MAY BE WITHDRAWN FROM THE STUDY WITHOUT YOUR CONSENT

You may be withdrawn from the study without your consent if any of the following occur:

- You are unable or unwilling to follow all of the study procedures or instructions.
- You could be harmed by continuing to take the pill or injection.
- The study is stopped or canceled.
- The study staff feels that staying in the study would be harmful to you.
- You are not able to attend clinic visits or complete all of the study procedures.
- Other reasons, as decided by the study staff.

ALTERNATIVES TO PARTICIPATION

[*Sites to include/amend the following if applicable:* There may be other studies going on here or in the community that you may be eligible for. If you wish, we will tell you about other studies that we know about. There also may be other places where you can go for HIV counseling and testing. We will tell you about those places if you wish.]

COSTS TO YOU

There will be no cost to you for study related visits, study products, physical examinations, laboratory tests, or other procedures.

REIMBURSEMENT

You will receive [$xx] for your time, effort, and travel to and from the clinic at each scheduled visit.

CONFIDENTIALITY

To keep your information private, your samples will be labeled with a code that can only be traced back to your study clinic. Your name, where you live, and other personal information will be protected by the study clinic. The results of any tests done on these samples will not be included in your health records. Every effort will be made to keep your personal information confidential, but we cannot guarantee absolute confidentiality. Your personal information may be disclosed if required by law.

Efforts will be made to keep your study records and test results confidential to the extent permitted by law. However, we cannot guarantee absolute confidentiality. You will be identified by a code, and personal information from your records will not be released without your written permission. Any publication of this study will not use your name or identify you personally. However, your records may be reviewed, under guidelines of the United States Federal Privacy Act, by the United States Food and Drug Administration (FDA); the sponsor of the study (United States National Institutes of Health [NIH]), the [*insert name of site*] Institutional Review Board (IRB), study staff, study monitors, the companies that make the drugs used in this study, other government and regulatory authorities, and (*insert applicable local authorities*].

[*For US sites only to include*] In addition to the efforts made by the study staff to help keep your personal information confidential, we have obtained a Certificate of Confidentiality from the U.S. Federal Government. This Certificate protects researchers from being forced to tell people who are not connected with this study, such as the court system, about your participation. The Certificate of Confidentiality does not prevent you from releasing information about yourself and your participation in the study. The Certificate cannot be used to resist a demand for information from personnel of the United States Government that is used for auditing or evaluation of federally funded projects or for information that must be disclosed in order to meet the requirements of the federal Food and Drug Administration (FDA). Even with the Certificate of Confidentiality, if the study staff learns of possible child abuse and/or neglect or a risk of harm to you or others, we will tell the proper authorities.

The study staff will also use your personal information, if needed, to verify that you are not taking part in any other research studies. This includes other studies conducted by [site name] and studies conducted by other researchers that study staff know about. Any publication of this study will not use your name or identify you personally.

Your records may be reviewed by:

- US FDA
- US NIH
- US Department of Heath and Human Services (DHHS), Office of Human Research Protection (OHRP)
- Other government and regulatory authorities
- [insert names of applicable IRBs/ECs/other local eview bodies as applicable]
- Study staff
- Study monitors
- The company that makes the study drug (ViiV Healthcare)

[Sites to include/amend the following if applicable:] [*Local/state/national*] regulations require study staff to report the names of people who test positive for [*HIV and other infections*] passed during sex to the [*local health authority*]. Outreach workers from the [*health authority*] may then contact you about

informing your partners, since they also should be tested. If you do not want to inform your partners yourself, the outreach workers will contact them, according to the confidentiality guidelines of the [*health authority*].

RESEARCH-RELATED INJURY

[Sites to specify institutional policy:] It is unlikely that you will be injured as a result of study participation. If you are injured, the [*institution*] will give you immediate necessary treatment for your injuries. You [*will/will not*] have to pay for this treatment. You will be told where you can get additional treatment for your injuries. There is no program to pay money or give other forms of compensation for such injuries either through this institution or the NIH. You do not give up any legal rights by signing this consent form.

PROBLEMS OR QUESTIONS

If you ever have any questions about the study, or if you have a research-related injury, you should contact [*insert name of the investigator or other study staff*] at [*insert telephone number and/or physical address*].

If you have questions about your rights as a research participant, you should contact [insert name or title of person on the IRB or other organization appropriate for the site] at [insert physical address and telephone number].

If you have questions about who to contact at the research site, you should contact [insert name of the investigator or community educator or CAB member] at [insert physical address and telephone number].

SIGNATURE PAGE

A Phase IIa Study to Evaluate the Safety, Tolerability and Pharmacokinetics of the Investigational Injectable HIV Integrase Inhibitor, GSK1265744, in HIV-uninfected Men and Women

(HPTN 077)

Version 3.0

October 13, 2015

DAIDS Document ID: 11964

SCREENING AND ENROLLMENT CONSENT

Insert signature blocks as required by the local IRB:] If you have read this consent form, or had it read and explained to you, and you understand the information, and you voluntarily agree to join the study, please sign your name or make your mark below. [*Sites to add and edit the following sentence as it pertains to your site, e.g., if you are not allowing genetic testing, then do not include the words "genetic testing" in the sentence:* Also, please indicate by providing your initials in the spaces below the additional sample collection, genetic testing, or long-term storage that you agree to.]

________ I agree to take part in this study.

________ I agree to have samples of my blood stored and used for future testing related to HIV infection.

________ I do not agree to have samples of my blood stored and used for future testing related to HIV infection.

________ I agree to allow my blood to be tested to see how my genes make the drug work in my body.

________ I do not agree to allow my blood to be tested to see how my genes make the drug work in my body.

________ I agree to allow my genetic information to be shared for future research purposes and for this information to be stored in a central genetic database.

________ I do not agree to allow my genetic information to be shared for future research purposes or for this information to be stored in a central genetic database.

________________________	____________________________________
Participant Name (print)	Participant Signature and Date
________________________	____________________________________
Study Staff Conducting Consent Discussion (print)	Study Staff Signature and Date
________________________	____________________________________
Witness Name (print) (As appropriate)	Witness Signature and Date

APPENDIX XIII: SAMPLE HPTN 077 PARTICIPANT LETTER/INFORMATION SHEET FOR PARTICIPANTS ENROLLED UNDER VERSION 2.0

A Phase IIa Study to Evaluate the Safety, Tolerability and Pharmacokinetics of the Investigational Injectable HIV Integrase Inhibitor, GSK1265744, in HIV-uninfected Men and Women

(HPTN 077)

Version 3.0
October 13, 2015
DAIDS Document ID: 11964

Sponsored by: Division of AIDS, United States (US) National Institute of Allergy and Infectious Diseases, US National Institutes of Health. Study products are provided by ViiV Healthcare

Dear HPTN 077 Participant:

We told you when you joined the study that we would tell you if we had any new information about this study or others that may impact you. The purpose of this letter is to share with you five pieces of information from this study, from another study called ÉCLAIR, and also from other studies in animals.

1. ÉCLAIR is very similar to this study, and is being conducted in the United States in men only. It is fully enrolled and is much farther along in progress than this study. It uses the same dose (30 mg) of the pill-version of GSK 1265744 for 4-weeks, the and the same injectable form of the drug in the same amount (800 mg), at the same time in between shots (every 12 weeks) at three time points, and for the same purpose, to test whether it is safe and how people respond to it.

In HPTN 077 and the ÉCLAIR study, the shots are given every 12 weeks because that is how long earlier studies in animals and humans showed that it lasted at high levels in the blood. But, those studies did not involve a large number of animals or people. In the ÉCLAIR study that enrolled over 100 men, they recently did a planned check on the levels of drug in the blood of the participants, and it showed in some participants that the drug did not last at high levels for the entire 12 weeks. Since it is hoped that this drug can eventually be used to protect people from getting HIV, it is important to find out how long the drug lasts in between shots so that people can remain protected.

Because of these findings, in HPTN 077, we have decided to enroll a second group of participants that will test a slightly different dose of the same drug (600 mg) GSK1265744, given as a shot every 8 weeks. We plan to enroll about 88 participants in this new second group, in addition to the approximately 105 participants we currently have in the first group. This will allow us to know whether this dose every 8 weeks is safe and whether it lasts in the blood at high levels between shots. A lower dose was chosen since the shots are closer together in time (every 8 weeks instead of every 12 weeks).

Your participation in the HPTN 077 study is very important because we still need to know that the 800 mg dose every 12 weeks that what was found in the ÉCLAIR study in men was correct, and also to see if the same is true in women.

2. Some people who have had a prior history of seizures (epilepsy) have had seizures (spells) while taking GSK 744. One person who did not have a previous history of seizures died after prolonged seizures. You may have already been contacted by us to see if you have ever had a seizure. If you have,

and you have not finished getting all of your shots, you will not get any more shots, and we will ask you to continue in the study. If you have gotten all of your shots, we will continue to follow you in the study. Either way, we will ask you about any neurologic symptoms during the study.

3. In studies done in pregnant rats and their newborns, using very high amounts of GSK 744, there were more baby rats that died when they were born or right after they were born. This did not happen to the baby rats when the mother got lower amounts of the drug, or no drug. The amount of GSK 744 that we are giving in this study is expected to be more than 7-times lower than the amount given to the pregnant rats. Birth defects have not been found in any animal studies of GSK 744 so far. We do not know whether what happened to the baby rats has any impact on what will happen in pregnant humans taking this drug. That is why if you are female and participating in this study, you cannot be pregnant, and we want you to take birth control the entire time you are in the study and also for a year after. If you become pregnant, we will not give you any more study medication and we will ask you to stay in the study so that we can follow you carefully.

4. We told you when you started the study that there have been some people who were taking this medicine in other studies who have had liver side effects. All of these people were HIV-infected (HIV positive) and they all had some damage to their liver before taking the GSK 744 study medication. In those studies, while taking the study medication, their blood tests showed that their liver was irritated, although they felt well. The medications were stopped, and the liver blood tests are normal. That is why anyone in HPTN 077 will not be able to participate if they have HIV-infection, Hepatitis C (or B), or any liver irritation.

5. We told you that the study drug was being provided by GlaxoSmithKline and ViiV Healthcare. Only ViiV Healthcare is providing the study drug now.

We will continue to update you on any new information, including side effects that we see in this study and other on-going studies, and if those side effects appear to have come from the drug.

The HPTN 077 study team wants you to continue in the study. Your continued participation in HPTN 077 is, as it has always been, entirely voluntary.

If you have any questions now or later about the information provided in this letter, you may ask the study staff or contact me directly. We will do our best to answer any questions or concerns that you may have.

Your participation in the HPTN 077 will lead to important discoveries that will hopefully lead to better prevention methods for protection against HIV infection. Thank you for participating in HPTN 077.

Sincerely,

[Insert name and contact information of Investigator of Record]

If you have read this letter, or have had it read and explained to you, and understand the information, please sign your name below.

________ I agree to continue to take part in this study.

________ I do not agree to continue to take part in this study.

____________________	____________________
Participant Name (print)	Participant Signature and Date
____________________	____________________
Study Staff Conducting<br>Consent Discussion (print)	Study Staff Signature and Date
____________________	____________________
Witness Name (print)<br>(As appropriate)	Witness Signature and Date
