# Supplementary material for: Safety, tolerability, and pharmacokinetics of long-acting injectable cabotegravir in low-risk HIV-uninfected individuals: HPTN 077, a phase 2a randomized controlled trial
Source: PLoS Med. 2018 Nov 8;15(11):e1002690. doi: 10.1371/journal.pmed.1002690 (PMC6224042; doi:10.1371/journal.pmed.1002690)
Supplement: S2 Text — (DOCX) [file pmed.1002690.s008.docx]

S2 Text. **Next generation sequencing methods**

Methods used for next generation sequencing (NGS) were modified from a previously reported protocol (Sivay et al., 2017).

HIV RNA was extracted from plasma using the ViroSeq HIV-1 Genotyping System (Abbott Molecular, Des Plaines, IL). Viral RNA was reverse transcribed using Superscript IV Reverse Transcriptase (Invitrogen, Carlsbad, CA) with the HS55 primer (Berg et al., 2016). The resulting cDNA was amplified in a separate PCR reaction using High Fidelity Platinum Taq Polymerase (Invitrogen, Carlsbad, CA) according to manufacturer instructions. PCR was performed using the GAG2 forward primer (Cousins et al., 2013) and the HIV5073-R reverse primer (Dudley et al., 2014).

Reverse transcription conditions were as follows: Viral RNA was denatured at 65°C for 30 seconds and then cooled at 50°C for 5 minutes. Reverse transcription was performed at 50°C for 60 minutes; the enzyme was then denatured at 80°C for 10 minutes, and the sample was cooled at 4°C for 10 minutes. Viral RNA was degraded by adding 1 µl RNase H to each sample and incubating the samples at 37°C for 20 minutes; the RNase H was then inactivated at 65°C for 10 minutes.

PCR conditions were as follows: The polymerase was activated at 94°C for 2 minutes, followed with three cycles of 94°C for 30 seconds, 61°C for 30 seconds, and 68°C for 4 minutes and 45 seconds. This was followed by three cycles of 94°C for 30 seconds, 58°C for 30 seconds, and 68°C for 4 minutes and 45 seconds. This was followed by 40 cycles at 94°C for 30 seconds, 55°C for 30 seconds, and 68°C for 4 minutes and 45 seconds. Five percent dimethyl sulfoxide (DMSO) was added at the end of the reaction. The resulting PCR products were purified by gel extraction (MinElute PCR purification kit, Qiagen, Hilden, Germany), and were quantified using picogreen dye and a Qubit fluorometer (Invitrogen).

NGS was performed using the MiSeq System. One nanogram of each sample was fragmented using Nextera XT reaction mixtures (Illumina, San Diego, CA). Compatible barcodes were selected, and the manufacturer's protocol was followed. The bead-based normalization method from Illumina library prep kit was used. The final diluted pool was denatured using sodium hydroxide according to the Illumina MiSeq protocol with 5% denatured PhiX control libraries. Samples were loaded into a MiSeq cartridge and sequenced on a MiSeq instrument (2*251 cycles) using a 600-cycle MiSeq reagent kit v3 (Illumina).

Data was first screened using CLC Genomics Workbench v10.0 software (Qiagen, Aarhus, Denmark). Paired-end reads were trimmed for quality (limit=0.05) and ambiguity (2-nt maximum). PCR primer sequences were removed*.* Reads *<*50 bases in length were discarded. Two bioinformatics tools were used to detect low-frequency HIV drug resistance mutations: (1) Trimmed reads from each sample were aligned to a reference sequence (HXB2 #NC_001802) using the CLC Genomics Workbench tool. The following alignment settings were applied: mismatch=2, insertion=3, deletion=3, length fraction=0.7, and similarity fraction=0.8. Default settings were used for error rate calculations. The frequency of drug resistance mutations was determined using the low frequency variant detection tool (Berg et al., 2016) (cut-off for significance: 2%; minimum frequency for variants: 1,000 reads). (2) Trimmed reads from each sample were also analyzed using the MinVar tool (Huber et al., 2017) under Ubuntu 16.04 with a 2% cut-off for significance. Data from each of the triplicate NGS reactions was analyzed using both analysis tools.

**Next generation sequencing results**

The read numbers for the samples ranged from 437,064 to 2,128,546. The minimum average sequencing depth was 26,480X.

|  | Analysis tool | Sample 1  (first HIV-pos visit) | Sample 2  (+1 days) | Sample 3  (+41 days) |
| --- | --- | --- | --- | --- |
| NRTI mutations | CLC tool | None | None | K65R (2.1%)^a,b^ |
|  | MinVar tool | None | None | K65R (2.9%, 2.2%)^c^  L210W (2.3%)  D67E (2.8%) |
| NNRTI mutations | CLC tool | None | None | None |
|  | MinVar tool | None | None | None |
| PI mutations | CLC tool | I84V (3.1%) | None | None |
|  | MinVar tool | I84V (2.9%) | None | None |
| INSTI mutations | CLC tool | None | None | None |
|  | MinVar tool | None | None | None |

Footnote for table:

A cutoff of 2% was used for both analysis tools for variant detection. Minvar is reported to provide reliable detection of mutations at levels ≥5% (Huber et al., 2017).

^a^ The K65R mutation was detected using the CLC genomic workbench tool in two of three triplicate runs; in one of those runs, the mutation was detected below the assay cutoff (1.7%, not shown).

^b^ The L210W was detected in one of three triplicate runs using the CLC genomic workbench tool, but was below the cutoff (1.3%, not shown).

^c^ The K65R mutation was detected using MinVar in two of three triplicate runs.

**References:**

Berg, M.G., Yamaguchi, J., Alessandri-Gradt, E., Tell, R.W., Plantier, J.C., Brennan, C.A., 2016. A pan-HIV strategy for complete genome sequencing. J Clin Microbiol 54, 868-882.

Cousins, M.M., Donnell, D., Eshleman, S.H., 2013. Impact of mutation type and amplicon characteristics on genetic diversity measures generated using a high-resolution melting diversity assay. J Mol Diagn 15, 130-137.

Dudley, D.M., Bailey, A.L., Mehta, S.H., Hughes, A.L., Kirk, G.D., Westergaard, R.P., O'Connor, D.H., 2014. Cross-clade simultaneous HIV drug resistance genotyping for reverse transcriptase, protease, and integrase inhibitor mutations by Illumina MiSeq. Retrovirology 11, 122.

Huber, M., Metzner, K.J., Geissberger, F.D., Shah, C., Leemann, C., Klimkait, T., Boni, J., Trkola, A., Zagordi, O., 2017. MinVar: A rapid and versatile tool for HIV-1 drug resistance genotyping by deep sequencing. J Virol Methods 240, 7-13.

Sivay, M.V., Li, M., Piwowar-Manning, E., Zhang, Y., Hudelson, S.E., Marzinke, M.A., Amico, R.K., Redd, A., Hendrix, C.W., Anderson, P.L., Bokoch, K., Bekker, L.G., van Griensven, F., Mannheimer, S., Hughes, J.P., Grant, R., Eshleman, S.H., 2017. Characterization of HIV seroconverters in a TDF/FTC PrEP study: HPTN 067/ADAPT. J Acquir Immune Defic Syndr 75, 271-279.

**Figure S1: Injection site reaction severity over time categorized by the initial injection site reaction severity stratum.**

ISR = injection site reaction

**Figure S2: Distributions relative to PA-IC_90_ between cohorts by sex at birth and BMI (≥median vs <median)**

IM = intramuscular injection; BMI = body mass index; PA-IC_90_ = protein-adjusted ninety percent inhibitor concentration
